# Electric Dipole Moments from Stark Effect in Supersonic Expansion: *n*-Propanol, *n*-Butanol, and *n*-Butyl Cyanide

**DOI:** 10.3390/molecules28041692

**Published:** 2023-02-10

**Authors:** Zbigniew Kisiel, Krzysztof Habdas

**Affiliations:** 1 Institute of Physics, Polish Academy of Sciences, Al. Lotników 32/46, 02-668 Warszawa, Poland; 2 Department of Chemistry, University College London, 20 Gordon Street, London WC1H 0AJ, UK

**Keywords:** electric dipole moments, supersonic expansion, rotational spectroscopy, molecular structure, hyperfine splitting

## Abstract

The orientation and magnitude of the molecular electric dipole moment are key properties relevant to topics ranging from the nature of intermolecular interactions to the quantitative analysis of complex gas-phase mixtures, such as chemistry in astrophysical environments. Stark effect measurements on rotational spectra have been the method of choice for isolated molecules but have become less common with the practical disappearance of Stark modulation spectrometers. Their role has been taken over by supersonic expansion measurements within a Fabry-Perot resonator cavity, which introduces specific technical problems that need to be overcome. Several of the adopted solutions are described and compared. Presently, we report precise electric dipole moment determinations for the two most stable conformers of the selected molecules of confirmed or potential astrophysical relevance: *n*-propanol, *n*-butanol, and *n*-butyl cyanide. All dipole moment components have been precisely determined at supersonic expansion conditions by employing specially designed Stark electrodes and a computer program for fitting the measured Stark shifts, inclusive of cases with resolved nuclear quadrupole hyperfine structure. The experimental values are compared with suitable quantum chemistry computations. It is found that, among the tested levels of computation, vibrationally averaged dipole moments are the closest to the observation and the molecular values are, as in the lighter molecules in the series, largely determined by the hydroxyl or nitrile groups.

## 1. Introduction

The inventory of interstellar, circumstellar, extragalactic, protoplanetary disks and exoplanetary molecules is considerable and in 2018 numbered 204 distinct species [1], which have been identified largely by rotational spectroscopy. At the typical 100 K temperatures of such objects, the maximum intensities of the rotational transitions in most target molecules fall in the millimetre wave (MMW) and submillimetre wave (SMM) regions of the electromagnetic spectrum. The advent of powerful radio telescopes, in particular the Atacama Large Millimeter Array (ALMA) operating at such frequencies [2,3], allows for the recording of broadband surveys of astrophysical objects of interest. These have proven to be a treasure trove for the detection of hitherto unknown species, which is carried out by comparison with the simulation of all known species from the data held in the spectroscopy databases [4,5]. This approach allowed, for example, the confident detection of molecules such as urea in Sgr-B2 [6] or CH_3_CN in Orion [7]. The size of the identified species is systematically increasing, with molecules such as benzonitrile [8] or cyanonaphtalene [9] recently detected in TMC-1, so that the accuracy of data in databases is critical. The increasing sensitivity of radio telescopes also requires, for example, spectroscopic coverage in the simulations of low-lying excited vibrational states or isotopic species. This provides continuous challenges to laboratory spectroscopy, and the improved spectral analysis allowed, for example, the detection of vibrationally-excited propyl cyanide on Titan [10], or two conformers of *n*-propanol in an interstellar molecular cloud [11].

Such detections are critically dependent on the reliable simulation of the absolute and relative intensities of the transitions of the various known astrophysical species, as well as of the target species. The electric dipole moment, *µ*, of a molecule is the property directly responsible for the intensity of the rotational transitions, which depend on *µ*^2^. The dipole moment is, therefore, of immediate relevance in astrophysical simulations unravelling extra-terrestrial chemistry. It does, however, appear to be a somewhat neglected quantity. The most common experimental method of determining the dipole moments of isolated molecules has been through the measurement of the Stark effect observed upon the application of an external electric field to gas phase samples. Stark effect measurements on rotational spectra were quite common at the time of the instrumental domination of the experimental rotational spectroscopy by Stark modulation spectrometers. Such spectrometers depended on the Stark effect for their operation since the spectra were observed by applying a fast-changing high voltage applied to the molecules contained in long rectangular waveguide cells [12,13]. The most prominent such spectrometer was a commercial design by the Hewlett-Packard company [14], which was purchased by a large number of university departments. Unfortunately, the majority of these spectrometers are no longer operational, and dipole moment determinations became much less common. The role of Stark modulation spectrometers has been taken over by supersonic expansion Fourier transform microwave (FTMW) spectrometers, which are capable of high sensitivity and also of Stark measurements, providing some technical challenges are mitigated. In this work a description of some of the adopted solutions is given, followed by the dipole moment determinations for three selected molecules, which have either been just detected in astrophysical environments or are promising candidates for such detection.

**Figure 1:**
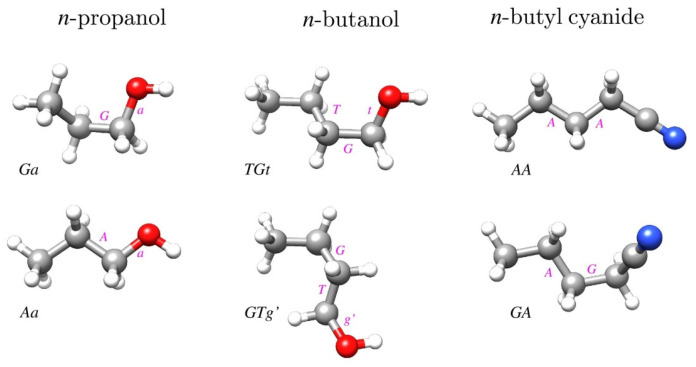
Geometries of two conformers of each of the three molecules studied in this work. The most stable conformer for each molecule is in the top row and conformer names are consistent with those used in the most recent rotational spectroscopy studies of these molecules [15,16,17]. The makeup of the conformer names is denoted by the magenta letters placed next to the bonds defining a given torsional orientation, leading to the conformer name by reading from left to right (or top to bottom for *GTg*’).

The geometries of the relevant conformers of the three studied molecules are displayed in Figure 1. In order to avoid confusion with the immediately preceding papers on these molecules, we retained the conformer notation used therein. We note, however, that the earlier used *T* designation used for *trans* has more recently been superseded by *A* for *anti* when referring to non-planar molecules. Thus, the two species of *n*-propanol have previously been denoted as *GT* and *TT*, while the two *n*-butanol species would in this scheme be denoted by *AGa* and *Gag*’.

## 2. Dipole Moment Measurements in Supersonic Expansion

**Figure 2:**
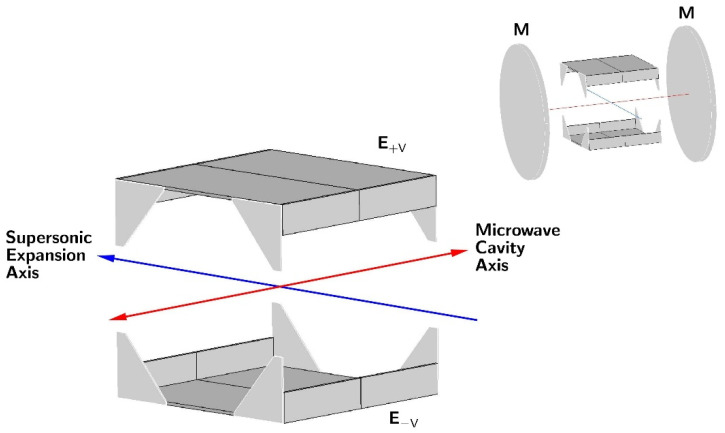
The electrodes (**E**) developed in Warsaw for ensuring improved electric field uniformity for Stark measurements on supersonically expanded samples and (upper right) their placement between the 50 cm diameter mirrors (**M**) of the Fabry-Perot resonator used for microwave measurements. The electrodes are in the form of 28 × 28 cm parallel plates, placed 27 cm apart, with additional side plates designed by numerical computations to focus the electric field in the interaction volume between the supersonic expansion plume and the microwave resonator mode. The main and side plates can be split into two parts for insertion and assembly through the easily accessible (15 cm and 10 cm diameter) central ports of the spectrometer chamber.

The most established approach for Stark measurements in rotational spectroscopy was based on the use of a long (2 m or longer) rectangular waveguide cell with an isolated central metal electrode [13]. The cell walls were at the ground voltage while the high voltage for Stark measurements was applied to the central electrode. The spacing between this electrode and the long walls of the cell was typically 0.5 cm, so that a central voltage of 1000 V corresponded to the application of an electric field of 2000 V/cm at the sample. The voltage of this order was deemed to be sufficiently safe from the point of view of avoiding discharge through the sample, while the cell geometry ensured electric field uniformity. Contemporary supersonic expansion measurements are made in a significantly different geometry, within a high vacuum chamber containing a sizable Fabry-Perot resonator [18]. The molecular expansion plume is directed either perpendicular or parallel to the resonator axis. The simplest way of applying an external electric field to the molecules is to use two parallel plate Stark electrodes inserted between the resonator mirrors, such that their resulting electric field is oriented perpendicular to the resonator axis; see the inset in Figure 2. The water dimer investigation [19] is a good example of the results obtainable with this straightforward design. Several deficiencies of this approach were noted and subsequent various attempts at providing technical and software solutions to the identified problems were published [20,21,22,23], as detailed below.

*Electric field uniformity*: One of the main problems associated with supersonic expansion measurements in cavity spectrometers derived from the Balle and Flygare design [18] is electric field uniformity. The underlying reason is that the microwave Fabry-Perot resonator should be as large as possible, since this determines the low frequency limit of good performance. At the same time, the electrodes inserted into the resonator should be separated by a significant distance if the microwave mode of the resonator is to remain unperturbed, while the resonator and the electrodes are contained within a metal high vacuum chamber that is subject to practical limits on its size. As an example, the design used in the present work has a cylindrical vacuum chamber with a diameter of 60 cm, containing 50 cm diameter mirrors, typically separated along the cylinder axis by 55–70 cm, depending on the resonator tuning. The optimum Stark electrode configuration in such a spectrometer is in the form of two square parallel metal plates, which are typically constrained to an edge size comparable to the plate separation. Such dimensions are, unfortunately, associated with poor electric field uniformity, due to significant ‘leaking’ of the field from the inter electrode volume. This has been investigated by numerical electric field calculations [20,23], and it is also directly reflected by effective cell separation determined during the calibrations performed with molecules of precisely known dipole moments. For infinite-size parallel plates, the electric field is given by ∆*V*/*d*, where ∆*V* is the voltage difference between the electrodes and *d* is their physical separation. In practice, effective fields were found to be significantly lower, in Ref. [19], for example, they were equal to 95% of that given by *V*/*d*. This effect is satisfactorily accounted for by the effective cell constant but is also associated with shorter decay times of the free induction decay (FID) signal, lowering the line intensity obtained on its Fourier transformation [24]. One natural solution for improving the field uniformity has been to increase the size of the Stark plates, while preserving their separation [20]. The resulting design had 70 × 50 cm plates separated by 25 cm, and was demonstrated to offer an improvement, although it was only facilitated by the availability of a particularly large high-vacuum chamber. An alternative approach has been to prevent the electric field from ‘leaking’ from the inter-electrode volume by means of a wire Stark cage [21]. In this design, the faces between the electrodes utilised equally spaced wires parallel to the electrodes. The neighbouring wires were connected by high resistance (5 MΩ) resistors and served as guard electrodes distributing the inter-electrode voltage in a uniform way. Improvement in the resulting field uniformity was demonstrated on the basis of the recorded line shapes. Another design, specific to the spectrometer configuration with parallel resonator and molecular expansion axes, has been to use the resonator mirrors as the Stark electrodes and to improve the electric field homogeneity by means of ring-shaped guard electrodes positioned between the mirrors [22]. The design was found to offer useful performance, but with a disadvantage arising from practical considerations, in that one of the resonator mirrors had to be at a high voltage of up to 15 kV.

The solution to the field uniformity problem used in the present work was arrived at on the basis of the numerical electric field calculations for the actual geometry of the experiment, inclusive of the effect of the chamber walls and resonator mirrors [23]. It turned out that significant improvement in the field uniformity could be obtained by attaching additional field-focusing metal plates to the basic parallel plate electrodes, as visible in the final design shown in Figure 2. The dimensions of the electrode attachments were adjusted so that the effective electrode separation determined from a molecular calibration was close to the physical separation. The performance of this electrode design has previously been described in some detail [23,24].

*Stark electrode calibration*: This is a compulsory step in Stark effect measurements, whereby the effective electrode separation is first determined by measuring the Stark shifts for a molecule with an accurately known dipole moment. In this way, any effects that may be outside the direct design control are accounted for. The relatively low electric fields attainable between the electrodes in supersonic expansion experiments have the result that the most common calibration with the *J* = 1←0 transition of the OCS molecule at 12,162.97 MHz is less than optimal. The dipole moment of OCS is relatively low at 0.715196(10) D [25], resulting, typically, as in our spectrometer, in a relatively small Stark frequency shift of less than 0.8 MHz for an already sizable voltage difference of 10 kV between the electrodes. For this reason, the possibility of using molecules with significantly larger dipole moments was investigated. Methyl iodide (CH_3_I, *µ* = 1.6406(4) D, [26]) and methyl cyanide (CH_3_CN, *µ* = 3.92197(13) D, [27]) turned out to be very useful in this respect. Their *J* = 1←0 transitions are near 15 GHz (CH_3_I) and 18.4 GHz (CH_3_CN), and each such transition is, furthermore, split into several hyperfine components, resulting from the presence of an atom with a quadrupolar nucleus. There is, therefore, plentiful data that can be acquired concerning the cell calibration, and the only reason hindering its previous use was the lack of a suitable computer program. Once this became available (see below), much more precise electrode separation calibrations could be performed. The results of the presently carried out calibrations for the Ne carrier gas-based expansions for both molecules are included in Supplementary Materials File S1. In these calibrations, the measured Stark shifts for CH_3_I were up to 4.4 MHz for applied voltages up to 12.3 kV, and for CH_3_CN Stark shifts were up to 12 MHz for voltages up 8.5 kV. The effective cell separations determined from these two calibrations were 26.972(3) cm from CH_3_I and 26.974(2) cm from CH_3_CN, in excellent agreement with each other, even in view of the very small statistical errors. In practice, it is considered safer to increase the estimated calibration uncertainty in order to account for various possible experimental effects, such as the possible changes in the shape of the supersonic expansion plume during measurements. For this reason, the calibration uncertainty is increased to 0.02 cm [24,28] and used in error propagation in all our dipole moment determinations.

*Analysis*: More careful analysis of the acquired Stark measurements is also necessary, due to the fact that Stark lobe shifts are often neither pure first- or second-order functions of the applied electric field. Often, pure second-order behaviour was assumed and the dipole moment was determined from the gradient of a simple straight line fit of the Stark frequency shift as a function of the square of the applied voltage. This was useful as a first approximation, but the recommended approach is to calculate Stark shifts on the basis of the complete Hamiltonian for the problem. This numerical treatment, using an irreducible tensor formalism, has been known for a long time [29]. It has been updated and made available by means of the contemporary computer code QSTARK [23], downloadable from the PROSPE website [30,31]. Thus, for the important H_2_SO_4_ molecule, the initially determined electric dipole moment value of 2.725(15) D [32] was significantly revised to 2.964(7) D [33] through a combination of new measurements and fitting. Similarly, the remeasurement of the dipole moment of acrylonitrile [34] led to revision of the previously anomalously high value of the *µ_b_* dipole moment component. This is much smaller than the *µ_a_* component, but at astrophysical temperatures it determines the intensities of the strongest rotational transitions of this molecule above 500 GHz [2,35], and the revision corresponded to a change in predicted intensities by a factor of 1.67 [34].

*Other technical issues*: These are less well defined but also need to be considered. In some of the previous studies [19,21], it was noted that there was some diffusion pump oil condensation on the electrodes, leading to a change in their effective Stark cell constant. In our spectrometer, there is a suitable oil baffle on top of the diffusion pump and the resonator chamber is connected to it by a large (40 cm diameter) right-angle valve, so that oil migration was never noticed. Another related problem arises from the use of insulating dielectric materials, to act as separators between the electrodes [21,23]. All of these (perspex, teflon, quartz, ceramics [23]) tended to acquire static charge induced by high operating voltages, and this distorted the electric field. The solution adopted in the Stark cell design shown in Figure 2 has been to dispense with the electrode separators altogether by moving all supporting elements to the back sides of the main plates [23]. An additional practical requirement arises from the need to minimise the possibility of the electric discharge between the electrodes and the chamber, or the supersonic expansion valve. A simple precaution, doubling the range of applicable safe Stark voltages, is to supply the electrodes with symmetrically opposed voltages, as indicated in Figure 2.

## 3. Results

Stark effect measurements are made on the lowest quantum number transitions, since, for those, the frequency shifts induced by an external electric field are the largest. The relevant transitions fall in the centimetre wave (CMW) microwave radiation region and can be measured with considerable precision under supersonic expansion conditions. On the other hand, the thrust of the contemporary analysis of rotational spectra is directed towards much higher frequencies of the MMW and SMM regions, since, at astrophysical or room temperatures transition intensities in those regions are maximised. It is, therefore, not surprising that these regions have proven to be the most useful for applied uses, such as astrophysical investigations of chemistry in star-forming regions. The downside is that the studied transitions are for considerable values of pertinent quantum numbers, and the reproduction of their frequencies necessitates the use of many parameters in often rather complex Hamiltonians. For this reason, the available data were refitted with the formulation of Watson’s asymmetric rotor Hamiltonian used by QSTARK, in order to ensure a good reproduction of the lowest frequency transitions. The resulting parameter values are summarised in Table 1, with further details specified in the section for each molecule. The complete results of all fits are also reported in Supplementary Materials File S2.

### 3.1. Electric Dipole Moments of Ga and Aa Conformers of n-Propanol

The rotational spectrum of *n*-propanol has been extensively measured at 8–550 GHz [37] and, more recently, also at 18–505 GHz [15]. The supersonic expansion measurement of the dipole moment of the most stable *Ga* conformer has already been reported [37] and was an update on an earlier room temperature value determined in [38]. The analysis of the rotational spectrum of the less stable *C_s_* symmetry *Aa* conformer proved to be quite challenging. The early measurement of its dipole moment was also published [38], while a reasonably comprehensive set of rotational transitions was later reported [39]. The transition measurements were considerably expanded on the basis of the MMW measurements [40], although only a limited number of the lines could be fitted at that time. The most recent independent measurements of the rotational spectrum of *n*-propanol [15] were made with the aim of deriving rotational transition frequencies for use in astrophysical investigations. The analysis for the *Aa* conformer turned out to be non-trivial and, while line lists for astrophysical use were generated, the frequencies of the measured transitions were not made available (as in fact, has also been the case in Ref. [40]). For this reason, we measured the lowest-*J*, lowest-*Ka* subset of rotational transitions of the *Aa* conformer by using the several spectrometers available in the Warsaw laboratory, as described in the Materials and Methods section. It was found that limiting the data to transitions with *J* ≤ 20 and *K_a_* ≤ 3 allowed a fit of the measured frequencies to the experimental accuracy with the use of only a moderate number of spectroscopic parameters. In the fit, the data acquired in this work were combined with those from Ref. [39], and, as carried out therein, the observed small internal rotation splitting was averaged for each line for which it was observed. The resulting spectroscopic parameters derived for use as fixed parameters in dipole moment determination are listed in Table 1, and the complete fit is included in Supplementary Materials File S2.

**Figure 3:**
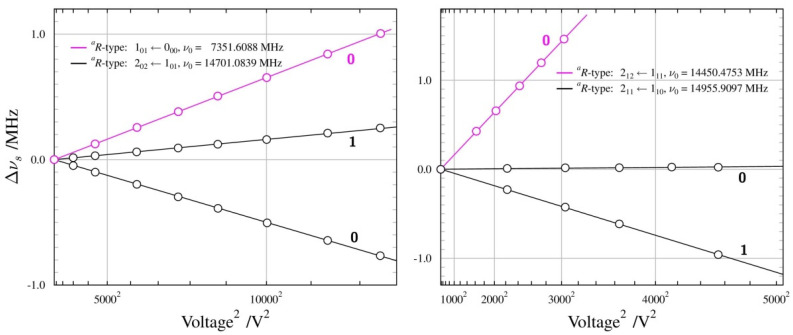
Summary of Stark measurements for the *Aa* conformer of *n*-propanol, plotted in the form of Stark frequency shift ∆*v*_s_ from zero-field transition frequency *v*_0_, as a function of the square of the applied voltage. Circles mark experimental points, while lines are drawn on the basis of the fitted dipole moment values. The calibrated Stark electrode separation is 26.97 cm, so that applied voltage of 10,000 V, for example, corresponds to electric field of 371 V/cm. All measured transitions are for the ∆*M* = 0 selection rule and values of the *M* quantum number are indicated for each Stark lobe. The apparent linear frequency shift dependence of the measured Stark lobes demonstrates their second-order behaviour.

Supersonic expansion measurements made possible the first observation of the lowest *J* rotational transitions allowed by the small, but non-zero, value of the *µ_a_* dipole moment component. None of these transitions displayed internal rotation splitting and, in fact, those proved to be the most useful for determining the dipole moment for the *Aa* conformer. The summary of the Stark measurements performed on the four different rotational transitions of this conformer is made diagrammatically in Figure 3. The measurements were made mostly with Ne carrier gas for the expansion, rather than the usual Ar carrier gas, since we found a considerable increase in the observed transition intensities with Ne. This is similar to the situation previously encountered for urethane, where the observation of the less stable conformer was, in fact, only possible in the Ne expansion [41].

Figure 3 shows that, for *Aa n*-propanol (unlike in the *n*-butyl cyanide further below), the Stark shift behaviour is fairly straightforward. It is also found that the Stark shifts of the transitions allowed by the *µ_a_* component were actually more sensitive to the much larger *µ_b_* component. The determination of both non-zero components (*µ_c_* is zero by symmetry) has, therefore, been possible entirely on the basis of *a*-type transitions, and is summarised in Table 2. The overall deviation of fit is comparable to 2 kHz, which is routinely taken to be the measurement precision of the used spectrometer. Table 2 also compares the obtained results with those from previous experimental work and with quantum chemistry computations. The current value of the *µ_a_* component is a significant improvement in precision and corresponds to a factor of almost three increases in the calculated intensity of *a*-type transitions, rationalising their relatively high experimental intensity. As for the computed results, the MP2 values are somewhat closer to the experiment than the DFT values, although both overestimate the experimental value. It should be remembered that the standard calculations deliver equilibrium values. The computation of vibrationally averaged dipole moments compatible with ground-state values from the experiment is possible with anharmonic force field calculations, and Table 2 shows that experiment and computation are, in this case, in closest agreement. It is also noted that the computations return practically identical total dipole moments for both conformers, while the experimental values are significantly different.

**Figure 4:**
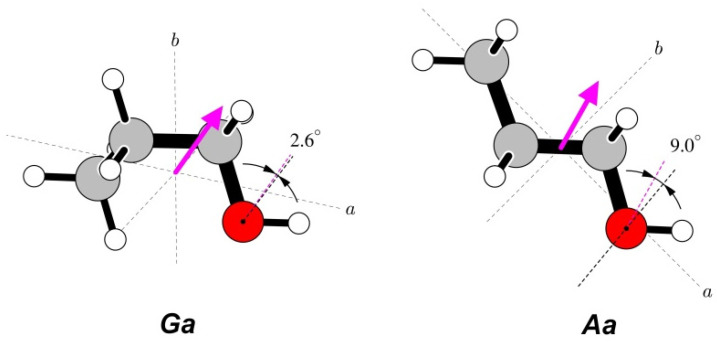
Orientations of the measured dipole moments in the two studied conformers of *n*-propanol. The dipole moment arrows are drawn from the centres of mass and are in the direction from the notional negative to the notional positive charge. Both molecules have been rotated such that the COH segments are oriented in a similar way in the plane of the paper. Dipole moment directions are compared at the oxygen atoms with orientations of the ∠COH bisectors. There is a rather close correspondence between these directions for the most stable *Ga* conformer, while for the *Aa* conformer, there is a significant distortion of the positive dipole moment direction towards the aliphatic group.

The total dipole moment does not carry direct information concerning its origin in a given molecule and is conventionally drawn from the centre of mass of the molecule, as in Figure 4. The direction of this vector quantity is not experimentally available but can be assigned on the basis of quantum chemistry computations. The actual origin of the dipole moment is expected to be in the small imbalance of electron charge distribution in the chemically active group terminating the aliphatic chain, in this case, of the hydroxyl group. While such information is, again, not directly available from the dipole moment measurement, it is possible to compare its angular orientation with that of its suspected carrier. Figure 4 shows that both the direction and dipole moment orientation for the *Ga* conformer are very close to that of the ∠COH bisector, while, for the *Aa* conformer, there is a significant deviation of the dipole moment direction towards the aliphatic chain.

### 3.2. Electric Dipole Moments of TGt and GTg’ Conformers of n-Butanol

The *n*-butanol molecule is the next step up from *n*-propanol. It has recently been extensively studied in supersonic expansion and rotational transitions in seven conformers have been assigned and fitted [16]. It was established that the global minimum conformer is *TGt*, with *GTg’* being the easiest to observe from among the less stable conformers (see Figure 1 for their structures). Data from Ref. [16] for both of these conformers were fitted therein with a Hamiltonian, accounting for the internal rotation splitting of some lines. In the present work, we used, as for the *Aa* conformer of *n*-propanol, the average frequencies of the A and E internal rotor component lines, in cases when such splitting was reported. For the *TGt* conformer, it was found, again as in *n*-propanol, that the measured data needed to be truncated in *K_a_* in order to avoid using too many, generally unphysical, parameters in the rotational Hamiltonian. Limiting the data to *K_a_* ≤ 2 transitions encompassed the experimental transition frequencies to within their uncertainty, at the cost of excluding 20 of the reported transitions that involved *K_a_* = 3. This was not necessary for the *GTg’* conformer since the reported transitions were all for *K_a_* ≤ 2.

None of the transitions used for the dipole moment measurement showed splitting of the zero-field line or of its Stark components. The frequency measurement precision was not reported in Ref. [16] and, for this reason, the deviations of fit reported in Table 3 of that paper (2.3 kHz for the *TGt* conformer, 2.8 kHz for the *GTg’* conformer) were used in deriving the deviation of fit, *σ*_rms_, values in Table 1.

**Figure 5:**
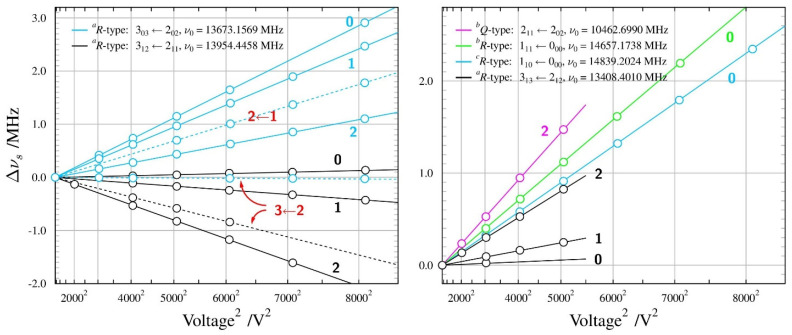
Summary of Stark measurements for the most stable, *TGt*, conformer of *n*-butanol (see the caption to Figure 3 for details). There are three non-zero electric dipole moment components in this molecule and it was possible to measure Stark lobes for rotational transitions allowed by all three components. In addition, the small non-degeneracy between the two nominally degenerate modes of the cylindrical Fabry-Perot resonator (see Ref. [24]) allowed for measurement also of several Δ*M* = +1 Stark lobes, with *M* values marked in red.

**Figure 6:**
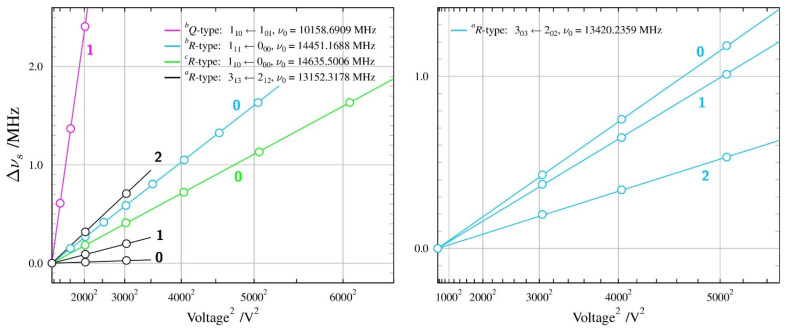
Summary of Stark measurements for the less stable *GTg’* conformer of *n*-butanol (see the caption to Figure 3 for details). As in the most stable *TGt* conformer, it was possible to measure Stark lobes of transitions allowed by non-zero values of all three non-zero dipole moment components.

The Stark measurements on the *TGt* conformer were made on supersonic expansion using the standard Ar carrier gas. The transitions of the *GTg’* conformer were too weak under these conditions, so the supersonic expansion was generated by diluting the sample in Ne carrier gas, as for the *Aa* conformer of *n*-propanol. The obtained results are summarised in Figure 5 and Figure 6 and in Table 3. The only previous dipole moment values available for *n*-butanol were the calculated ones [16] and it turns out that the experimental values are closer to the calculations performed at the computational levels adopted in this work. The computations still overestimate the experimental values, although vibrational averaging leads to the best agreement between the experiment and computation, especially for the lowest energy *TGt* conformer.

**Figure 7:**
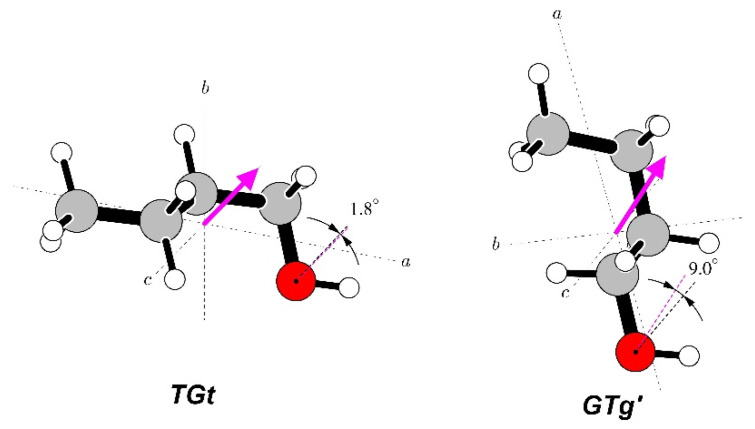
Orientations of the measured electric dipole moments in the two studied conformers of *n*-butanol. Both molecules, as in the case of *n*-propanol, have been rotated such that the COH atom chains are in the plane of the diagram at a comparable orientation. Dipole moment directions are compared at the oxygen atoms with ∠COH bisectors and, as in *n*-propanol, are seen to be almost coincident for the most stable *TGt* conformer, while in the *GTg’* conformer, the positive direction of the dipole moment is significantly distorted in the direction of the aliphatic chain.

A comparison of the determined dipole moments with the molecular structures of *n*-butanol is made in Figure 7. There is a marked similarity with a corresponding comparison for *n*-propanol in Figure 4, as the molecular dipole moment for the most stable conformer is aligned much more closely with the ∠COH bisector than is the case in the less stable conformer. The distortion of the dipole moment direction in relation to the ∠COH bisector for the less stable *GTg’* conformer is also in the same direction and magnitude as for *Aa n*-propanol, i.e., towards the aliphatic chain.

### 3.3. Electric Dipole Moments of AA and GA Conformers of n-Butyl Cyanide

The *n*-butyl cyanide (valeronitrile) molecule was found to exhibit lines of three conformers, *AA*, *GA,* and *AG*, in the rotational spectrum [42]. The *AA* conformer was identified to be the most stable one, while of the two less stable conformers it was *GA* that showed a significantly stronger rotational spectrum in supersonic expansion. For this reason, only the *AA* and *GA* conformers (see Figure 1) were studied in the present work. Both of these conformers do not display internal rotation splitting, although, in the supersonic expansion, all low-*J* transitions were split into several resolvable components arising from nuclear quadrupole splitting, due to the *I* = 1 spin of the ^14^N nucleus. The most recently reported extensive data for *n*-butyl cyanide [17] came from room temperature MMW spectra and did not have resolved hyperfine splitting, but its analysis was combined in a global fit with the hyperfine resolved data set [42]. The spectroscopic constants to be fixed in the dipole moment fits, and reported in Table 1, have presently been redetermined on the basis of the combined data set [17]. The reason for the redetermination was that the fits in Table 1 of Ref. [17] are for the *S*-reduced form of Watson’s asymmetric rotor Hamiltonian, while QSTARK requires *A*-reduction constants.

**Figure 8:**
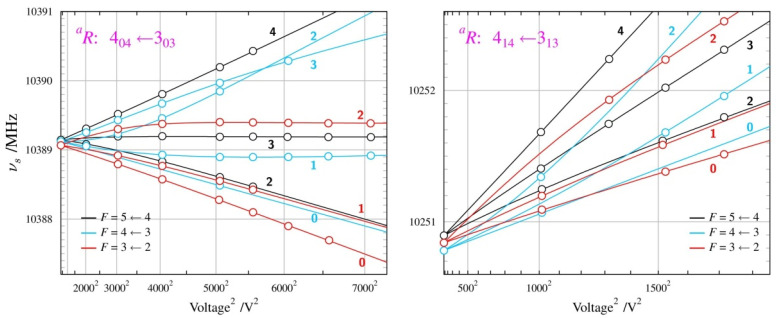
Summary of Stark measurements for the most stable, *AA* conformer, of *n*-butyl cyanide. In this case, actual frequencies, rather than Stark shifts, are plotted as a function of the square of the applied voltage. The molecule has nuclear quadrupole hyperfine structure so that each rotational transition is split into several components characterised by values of the *F* quantum number, and each such component becomes further split into Stark components for distinct values of *M_F_* (marked next to each Stark curve). Significant departures from the straight-line second-order behaviour are visible (see text) and allow determination of both non-zero dipole moment components from measurements only on transitions allowed by the *µ_a_* dipole moment component.

**Figure 9:**
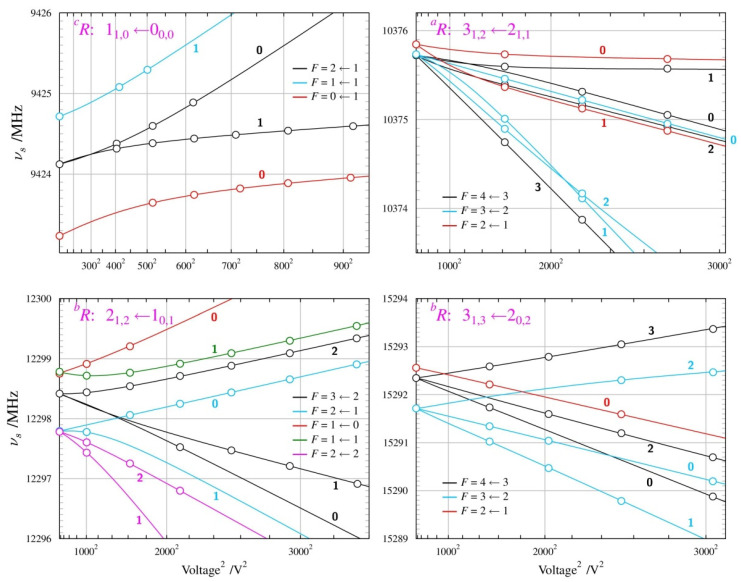
Summary of Stark measurements for the less stable *GA* conformer of *n*-butyl cyanide (see the caption of Figure 8 for details). In this case, it was possible to measure Stark components of rotational transitions allowed by all three non-zero dipole moment components, which are the only parameters of fit reproducing the complex behaviour in the four panels.

The Stark behaviour of the two studied conformers (Figure 8 and Figure 9) was measured with Ar carrier gas and was significantly different from each other, and also different from those for the alcohol molecules above. The results of the dipole moment determinations are summarised in Table 4. The presence of nuclear quadrupole hyperfine coupling leads to hyperfine splitting already at the zero Stark field. There are several frequency components characterised by the total angular moment quantum number *F*, which accounts for the sum of the rotational and nuclear spin angular momenta. Distinct Stark components then arise from the projection of this total angular momentum onto the external electric field axis, described by the quantum number *M_F_* (instead of M for the spinless case). Due to the multiple allowed values of the *F* and *M_F_* quantum numbers and the small values of the nuclear quadrupole splitting constants for the nitrogen nucleus (see Supplementary Materials Files S1 and S2), there is considerable energy level density, providing scope for perturbations between these levels when subjected to an external electric field. This leads to unusual curvatures in the field behaviour of the many possible Stark components, which is, fortunately, accounted for satisfactorily by the QSTARK program [23]. These curvatures are apparent for both conformers, but are more pronounced for the *GA* conformer, as visible in Figure 9.

The *AA* conformer has *C_s_* molecular symmetry, so that it has only two non-zero dipole moment components. In fact, only the *R*-branch (∆*J* = +1) transitions associated with the *µ_a_* dipole moment component turned out to be usable for the Stark measurements. The *µ_b_* allowed transitions in the available frequency region turned out to be too weak. Nevertheless, the Stark shifts for the chosen transitions were found to be sufficiently sensitive to both dipole moment components, allowing for their precise determination; see Table 4. This is similar to the behaviour found above for the *Aa* conformer of *n*-propanol.

The less stable *GA* conformer has all three non-zero dipole moment components, although the *µ_c_* component is very small and previously has not even been considered [42]. Presently, it was possible to measure the Stark shifts for rotational transitions associated with all three dipole moment components, including the 1_10_←0_00_ transition allowed by the non-zero value of *µ_c_*. Unfortunately, the Stark shifts for this transition turned out to be much more sensitive to the other two, *µ_a_* and *µ_b_*, components. For this reason, the precision in the determination of *µ_c_* for the *GA* conformer is the poorest. The deviations of the dipole moment fit for both conformers are still reasonable, but somewhat above the nominal 2 kHz precision of the spectrometer. Comparisons in Table 4 highlight the relatively poor performance of the estimates made in Ref. [42]. These are significantly inferior to the quantum chemistry values computed presently. The correspondence between the experiment and vibrationally averaged values for both conformers turns out to be very good and is the best out of all molecules considered in this work. This concerns both the magnitude and the angular orientation of the dipole moment.

**Figure 10:**
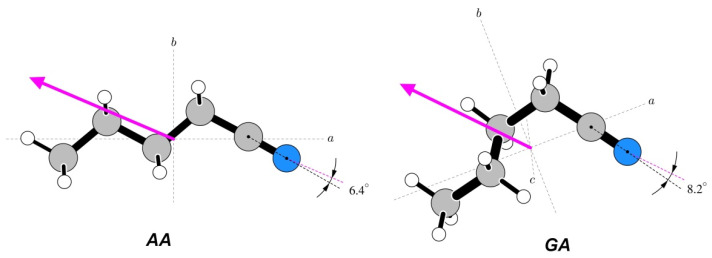
Orientations of the measured dipole moments in the two studied conformers of *n*-butyl cyanide. Both molecules are rotated, such that the CCN atom segment is in the plane of the diagram and at a comparable orientation. The marked angles are between the CN axis and the dipole moment direction.

For *n*-butyl cyanide, it is the nitrile group that is expected to be the carrier of the dipole moment. Comparison of the orientations relevant to this point is made in Figure 10. It can be seen that, for both conformers, the positive dipole moment axis is shifted significantly towards the alkyl group. There is a greater angular shift for the *GA* conformer, in which the alkyl chain is more folded and thus closer to the nitrile group.

## 4. Materials and Methods

Commercially available samples were used in all cases. The Stark effect measurements were carried out with the supersonic expansion Fabry-Perot cavity Fourier transform spectrometer in Warsaw [43,44]. This spectrometer is a coaxial waveguide version of the ground-breaking design of Balle and Flygare [18] and has been equipped with specially designed Stark electrodes (see Figure 2). The electrodes have been optimised for the generation of a uniform electric field in the interaction volume between the molecular expansion plume and the microwave mode in the Fabry-Perot resonator [23]. Electric field calibrations were performed on the basis of the Stark effect measurements on CH_3_I and CH_3_CN molecules. In all cases, the samples were diluted to 1% or less in Ar or Ne carrier gases, and were expanded into the microwave cavity of the spectrometer from a backing pressure of *ca* 1 atm for Ar and *ca* 2 atm for Ne. The sample input was performed by pulsing an electromagnetic expansion valve at a rate of 2 Hz, with 8–20 microwave response signals collected over 1 ms duration for each gas pulse. The stark measurements were made for the parallel configuration of the electric field vectors of the microwave radiation and external electric field. This configuration generated the smallest number of Stark components, limited to those allowed by the ∆*M* = 0 selection rule. The alternative perpendicular configuration for the measurement of components allowed by the ∆*M*= ±1 selection rule is possible by means of the simple expedient of rotating by 90° the L-type coupling antennas in the centres of the resonator mirrors [24]. All Stark measurements, for both calibrations and dipole moment determinations, were fitted with the QSTARK program [23] available on the PROSPE website [30,31]. In all dipole moment fits, the only adjustable parameters were the two or three allowed non-zero dipole moment components. The output files of all fits are collected in Supplementary Materials File S1.

Additional measurements for determining the fixed spectroscopic constants to use for *n*-propanol were made at room temperature in the CMW and in the MMW regions with two other spectrometers, also available in the Warsaw laboratory. The CMW measurements were made at 8.2–16.9 GHz with the waveguide FTMW spectrometer originally constructed in Kiel, Germany [45,46], with modifications described in the recent study of the phenol molecule [47]. The MMW measurements were made at 89.7–137.4 GHz with the broadband spectrometer constructed in Warsaw [48], operated in this case with probing radiation from a 12× harmonic generation source [49].

The fits of the spectroscopic constants and the resulting spectroscopic predictions were carried out either with the ASFIT/ASROT package [50], also available on the PROSPE website, or with the SPFIT/SPCAT package of H.M. Pickett [51,52]. The estimated frequency precision of the supersonic expansion measurements is 2 kHz, of WG-FTMW measurements 10 kHz, and of MMW measurements 50 kHz, and these values were used when weighted fits were necessary.

Quantum mechanical computations were carried out with Gaussian 16 [53] and CFOUR 2 [54] packages, both of which facilitate vibrationally averaged dipole moment calculations with numerically identical results.

## 5. Conclusions

The aim of this paper has been to provide an overview of the techniques employed in electric dipole moment measurements performed on rotational transitions recorded in supersonic expansion, to determine the dipole moments for several molecules of astrophysical relevance, and to benchmark dipole moment estimates made when experimental values are not available. The present results show (fortunately) that the direct experimental measurement of molecular electric dipole moments is still superior to quantum chemistry computations, and, even more so, to estimates from similar molecules. It is noted, however, that a suitably chosen level of computation can be quite reliable. This is the case for both conformers of *n*-butyl cyanide measured presently, and as found previously for acrylonitrile and propionitrile [34]. In all of the three title molecules, the vibrational averaging of the computed values improves the correspondence with the experimental (ground state) values. For the two alcohol molecules, the computed values are significantly closer to the experiment for the most stable conformers, showing that the vibrational effects in their less stable conformers are accounted for less successfully. The diagrams in Figure 4, Figure 7 and Figure 10 are useful illustrations of the correlations between the experimental dipole moments and molecular structure and can be made without the need for any additional computations.

## Figures and Tables

**Table 1:** Redetermined rotational and centrifugal distortion constants for use as fixed parameters in the evaluation of electric dipole moments.

	*n*-Propanol	*n*-Butanol		*n*-Butyl Cyanide	
Parameter ^a^	*Aa*	*TGt*	*GTg’*	*AA*	*GA*
*A*/MHz	26,401.4765(25)	12,467.74156(60)	12,305.00007(57)	15,028.68713(43)	7635.62481(21)
*B*/MHz	3802.15318(36)	2371.51771(10)	2330.57664(34)	1334.107310(22)	1788.636927(33)
*C*/MHz	3549.45833(49)	2189.47940(11)	2146.23454(32)	1263.856699(21)	1554.218399(37)
∆*_J_*/kHz	0.9788(27)	0.75626(66)	0.7131(16)	0.1458342(27)	0.878123(12)
∆*_JK_*/kHz	−0.658(58)	−6.180(10)	−2.543(16)	−7.54678(13)	−10.00176(14)
∆*_K_*/kHz	151.28(54)	60.25(11)	73.01(23)	214.3805(81)	52.4857(12)
*δ_J_*/kHz	0.12547(94)	0.12484(13)	0.13705(59)	0.0220207(19)	0.2333427(65)
*δ_K_*/kHz	−6.32(16)	2.063(35)	2.45(15)	0.48160(39)	1.72956(61)
*N_l_*_ines_	103	72	53	1481	1464
*σ*_rms_	1.060 ^b^	0.944	0.697	0.939 ^b^	0.937 ^b^

^a^ *A*, *B*, *C* are rotational constants; ∆*_J_* to *δ_K_* are quartic centrifugal distortion constants (from a fit of Watson’s A-reduced asymmetric rotor Hamiltonian in representation I*^r^* [36]); *N*_lines_ is the number of fitted distinct frequency measured lines; and *σ*_rms_ is the unitless deviation of the weighted fit with assumed frequency uncertainties given in the text. ^b^ Additional higher level centrifugal distortion constants have been used (Supplementary Materials Files S1 and S2), although those do not have an effect on the dipole moment determination.

**Table 2:** Electric dipole moments of the *Ga* and *Aa* conformers of *n*-propanol.

	*n*-Propanol *Ga*					*n*-Propanol *Aa*				
Parameter ^a^	Ref. [38] ^b^	Ref. [37] ^c^	Calc. ^d^	Calc. ^e^	Calc. ^f^	Ref. [38] ^b^	This work ^c^	Calc. ^d^	Calc. ^e^	Calc. ^f^
|*µ_a_*|/D	0.32(6)	0.4914(4)	0.529	0.580	0.570	0.21(7)	0.3589(7)	0.319	0.321	0.282
|*µ_b_*|/D	1.23(2)	0.9705(13)	1.020	1.079	1.071	1.54(2)	1.2820(13)	1.422	1.500	1.549
|*µ_c_*|/D	0.94(2)	0.9042(12)	0.894	0.929	1.009	0	0	0	0	0
*µ*_tot_/D	1.58(3)	1.4145(17)	1.456	1.538	1.578	1.55(3)	1.3312(13)	1.458	1.534	1.575
*N*_lines_		43					37			
*σ*_fit_/kHz		2.08					2.29			

^a^ *µ_a_*, *µ_b_*, *µ_c_* are electric dipole moment components along the *a*, *b*, *c* principal axes, respectively; *N*_trans_ is the number of fitted Stark measurements; and *σ*_fit_ is the deviation of the fit made with the QSTARK program by assuming the values of rotational and centrifugal distortion constants, as discussed in the text. ^b^ Experimental value from Stark measurements on room-temperature rotational spectrum. ^c^ Experimental value from Stark measurements of rotational spectrum in supersonic expansion. ^d^ Vibrationally averaged value computed at the MP2/aug-cc-pVDZ level. ^e^ Equilibrium value computed at the MP2/aug-cc-pVDZ level. ^f^ Equilibrium value computed at the B3LYP/6-311+G(2d,p) level.

**Table 3:** Electric dipole moments of the *TGt* and *GTg’* conformers of *n*-butanol.

	*n*-Butanol *TGt*					*n*-Butanol *GTg’*				
Parameter ^a^	Ref. [16] ^b^	This Work	Calc. ^c^	Calc. ^d^	Calc. ^e^	Ref. [16] ^b^	This Work	Calc. ^c^	Calc. ^d^	Calc. ^e^
|*µ_a_*|/D	0.89	0.7137(10)	0.752	0.813	0.805	1.26	1.0881(11)	1.175	1.215	1.168
|*µ_b_*|/D	1.05	0.8989(8)	0.934	0.988	0.990	1.35	1.0304(12)	1.164	1.203	1.315
|*µ_c_*|/D	0.94	0.8071(8)	0.801	0.829	0.910	0.80	0.6980(16)	0.719	0.764	0.730
*µ*_tot_/D	1.67	1.4032(11)	1.442	1.525	1.567	2.01	1.6532(16)	1.804	1.873	1.904
*N_l_*_ines_		68 + 6					31 + 5			
*σ*_fit_/kHz		3.15					3.12			

^a^ See the corresponding footnote to Table 2 for explanation of the parameters. ^b^ Equilibrium values computed at the *cam*-B3LYP/6-311++G(d,p) level. ^c^ Vibrationally averaged values computed at the MP2/aug-cc-pVDZ level. ^d^ Equilibrium values computed at the MP2/aug-cc-pVDZ level. ^e^ Equilibrium values computed at the B3LYP/6-311+G(2d,p) level.

**Table 4:** Electric dipole moments of the *AA* and *GA* conformers of *n*-butyl cyanide.

	*n*-Butyl Cyanide *AA*					*n*-Butyl Cyanide GA				
Parameter ^a^	Ref. [42] ^b^	This Work	Calc. ^c^	Calc. ^d^	Calc. ^e^	Ref. [42]	This Work	Calc. ^c^	Calc. ^d^	Calc. ^e^
|*µ_a_*|/D	3.40	3.9047(32)	3.885	3.914	4.076	2.3	2.6856(15)	2.656	2.669	2.894
|*µ_b_*|/D	1.90	1.6662(16)	1.665	1.688	1.714	3.1	2.8521(36)	2.841	2.882	2.919
|*µ_c_*|/D	0	0	0	0	0	0	0.390(17)	0.417	0.419	0.442
*µ*_tot_/D	3.89	4.2454(36)	4.226	4.262	4.421	3.86	3.9368(36)	3.912	3.950	4.134
*N_l_*_ines_		70 + 7					85 + 14			
*σ*_fit_/kHz		3.02					3.47			

^a^ See the corresponding footnote to Table 2 for explanation of the parameters. ^b^ Estimated by rotating the dipole moment of methyl cyanide to the orientation of the CN group in each *n*-butyl cyanide conformer. ^c^ Vibrationally averaged value computed at the MP2/aug-cc-pVDZ level. ^d^ Equilibrium value computed at the MP2/aug-cc-pVDZ level. ^e^ Equilibrium value computed at the B3LYP/6-311+G(2d,p) level.

## Data Availability

Most of the relevant data are contained within the article or supplementary material. In addition, primary input and output files for the performed fits are available at http://info.ifpan.edu.pl/~kisiel/data.htm (accessed on 8 February 2023).

## Supplementary Materials

The following are available online at https://www.mdpi.com/article/10.3390/molecules28041692/s1, File S1: collected results of dipole moment fits for the title molecules; File S2: collected results of spectroscopic fits for determining spectroscopic constants to be fixed in dipole moment fits.

## Abbreviations

The following abbreviations are used in this manuscript:

FTMW	Fourier Transform Microwave
CMW	Centimetre-wave
MMW	Millimetre-wave
SMM	Submillimetre-wave
