# Supplementary material for: Electric Dipole Moments from Stark Effect in Supersonic Expansion: n-Propanol, n-Butanol, and n-Butyl Cyanide
Source: Molecules. 2023 Feb 10;28(4):1692. doi: 10.3390/molecules28041692 (PMC9961461; doi:10.3390/molecules28041692)
Supplement: Supplementary file 1 [file molecules-28-01692-s001.zip › File_S1.pdf]

Supplementary file for the paper:

Krzysztof Habdas and Zbigniew Kisiel,
"Electric Dipole Moments from Stark Effect in Supersonic Expansion:
n-propanol, n-butanol, and n-butyl cyanide"
Molecules 2023, 28, 1962. https://doi.org/10.3390/molecules28041692

//

Table S1. This file concatenates output files resulting from fitting
frequencies of measured Stark components and electrode calibrations
made with the QSTARK program.

1/ Electric dipole moment fit for the Aa conformer of n-propanol
2/ Electric dipole moment fit for the TGt conformer of n-butanol
3/ Electric dipole moment fit for the GTg' conformer of n-butanol
4/ Electric dipole moment fit for the AA conformer of n-propyl cyanide
5/ Electric dipole moment fit for the GA conformer of n-propyl cyanide
6/ Example Stark electrode distance calibration using CH3CN in Ne expansion
7/ Example Stark electrode distance calibration using CH3I in Ne expansion

NOTE: the various outputs are separated by three lines as below, so that the
text 'cut here' can be used to rapidly locate the different sections.

```
////////////////////////////////////////////////////////////////////////////////////////////
//////////////// cut here - please display and print using a fixed width font ///////////////
////////////////////////////////////////////////////////////////////////////////////////////

  ___________________________________________________________________________
 |                                                                           |
 |   QSTARK - Fully diagonalizing fit of Stark shifts in a rotor             |
 |            with zero or one quadrupolar nuclei                            |
 |___________________________________________________________________________|
  version 14.XI.2022                                          Zbigniew KISIEL


 -----------------------------------------------------------------------------
 trans-trans n-propanol, ground state
 -----------------------------------------------------------------------------

 Calculation for J and F exceeding the value in data by at least 2
 The fit will be made to TRANSITION FREQUENCIES

    41 Lines read in
    41 Lines fitted    --->    4 field free and   37 at non-zero field
     2 Constants fitted


 TRANSITIONS (F and MF in units of 1/2):


      J K  K  <- J K  K     F MF<- F MF    Volts        Obs          Obs-Calc       Calc         Field      Field**2
         -1 +1      -1 +1
!
! 2006 measurements, Ar carrier d=26.950
! 2022 measurements, Ne carrier d=26.9727 and the actually applied voltages
!                       have been corrected to d=26.950 by
!                       V_old =  V_new *  ( d_old/d_new ) = V_new * 0.999158
!                       where 0.999158 = 26.950/26.9727
!
! Rotational and cd constants are from the ASFIT fit of
! Dreizler+Scappini 1981 data and IFPAN BF-FTMW + WG-FTMW + MMW
! lines (to average frequencies in cases of resolved A,E splitting)
!
!-------------------------------------------------
!  101 <- 000
!-------------------------------------------------
!
  1.   1  0  1   0  0  0    2  0   0  0       0.0     7351.60880      0.00121      7351.6076      0.0000         0.00
!
  2.   1  0  1   0  0  0    2  0   0  0    4391.3     7351.73360     -0.00022      7351.7338    162.9425     26550.25
  3.   1  0  1   0  0  0    2  0   0  0    6244.8     7351.86470      0.00184      7351.8629    231.7180     53693.23
  4.   1  0  1   0  0  0    2  0   0  0    7645.7     7351.99050      0.00026      7351.9902    283.6994     80485.37
  5.   1  0  1   0  0  0    2  0   0  0    8777.4     7352.11480      0.00291      7352.1119    325.6920    106075.29
  6.   1  0  1   0  0  0    2  0   0  0   10005.6     7352.26220     -0.00068      7352.2629    371.2653    137837.93
  7.   1  0  1   0  0  0    2  0   0  0   11349.8     7352.44990     -0.00086      7352.4508    421.1429    177361.31
  8.   1  0  1   0  0  0    2  0   0  0   12400.3     7352.61340     -0.00065      7352.6140    460.1224    211712.67
!
!-------------------------------------------------
!  202 <- 101
!-------------------------------------------------
!
```

```
  9.   2  0  2   1  0  1   4  0   2  0       0.0    14701.08390      0.00055     14701.0834      0.0000          0.00
!
 10.   2  0  2   1  0  1   4  2   2  2    2991.3    14701.09890      0.00111     14701.0978    110.9944      12319.76
 11.   2  0  2   1  0  1   4  2   2  2    4390.5    14701.11460      0.00014     14701.1145    162.9128      26540.58
 12.   2  0  2   1  0  1   4  2   2  2    6232.3    14701.14510     -0.00094     14701.1460    231.2542      53478.49
 13.   2  0  2   1  0  1   4  2   2  2    7641.1    14701.17630     -0.00128     14701.1776    283.5288      80388.56
 14.   2  0  2   1  0  1   4  2   2  2    8778.3    14701.20630     -0.00142     14701.2077    325.7254     106097.05
 15.   2  0  2   1  0  1   4  2   2  2   10003.9    14701.24360     -0.00127     14701.2449    371.2022     137791.09
 16.   2  0  2   1  0  1   4  2   2  2   11349.8    14701.29470      0.00344     14701.2913    421.1429     177361.31
 17.   2  0  2   1  0  1   4  2   2  2   12393.4    14701.33590      0.00465     14701.3313    459.8664     211477.12
!
 18.   2  0  2   1  0  1   4  0   2  0    2994.2    14701.03580     -0.00276     14701.0386    111.1020      12343.66
 19.   2  0  2   1  0  1   4  0   2  0    4395.3    14700.98350     -0.00334     14700.9868    163.0909      26598.64
 20.   2  0  2   1  0  1   4  0   2  0    6239.3    14700.88660     -0.00227     14700.8889    231.5139      53598.69
 21.   2  0  2   1  0  1   4  0   2  0    7641.1    14700.78710     -0.00458     14700.7917    283.5288      80388.56
 22.   2  0  2   1  0  1   4  0   2  0    8783.8    14700.69510     -0.00284     14700.6979    325.9295     106230.04
 23.   2  0  2   1  0  1   4  0   2  0   10014.6    14700.57950     -0.00289     14700.5824    371.5993     138086.01
 24.   2  0  2   1  0  1   4  0   2  0   11349.8    14700.43950     -0.00044     14700.4399    421.1429     177361.31
 25.   2  0  2   1  0  1   4  0   2  0   12393.4    14700.31770      0.00147     14700.3162    459.8664     211477.12
!
!
!-------------------------------------------------
!  212 <- 111
!-------------------------------------------------
!
 26.   2  1  2   1  1  1   4  0   2  0       0.0    14450.47530     -0.00103     14450.4763      0.0000          0.00
!
 27.   2  1  2   1  1  1   4  0   2  0    1628.4    14450.90260      0.00216     14450.9004     60.4230       3650.94
 28.   2  1  2   1  1  1   4  0   2  0    2027.9    14451.13190     -0.00160     14451.1335     75.2468       5662.07
 29.   2  1  2   1  1  1   4  0   2  0    2423.6    14451.41220     -0.00182     14451.4140     89.9295       8087.31
 30.   2  1  2   1  1  1   4  0   2  0    2736.4    14451.67130      0.00075     14451.6706    101.5362      10309.60
 31.   2  1  2   1  1  1   4  0   2  0    3030.3    14451.93740     -0.00201     14451.9394    112.4416      12643.10
!
!-------------------------------------------------
!  211 <- 110
!-------------------------------------------------
!
 32.   2  1  1   1  1  0   4  0   2  0       0.0    14955.90970     -0.00154     14955.9112      0.0000          0.00
!
 33.   2  1  1   1  1  0   4  0   2  0    2224.7    14955.91930      0.00203     14955.9173     82.5492       6814.36
 34.   2  1  1   1  1  0   4  0   2  0    3045.1    14955.92650      0.00396     14955.9225    112.9907      12766.90
 35.   2  1  1   1  1  0   4  0   2  0    3646.7    14955.92770      0.00026     14955.9274    135.3135      18309.76
 36.   2  1  1   1  1  0   4  0   2  0    4147.8    14955.93460      0.00240     14955.9322    153.9072      23687.44
 37.   2  1  1   1  1  0   4  0   2  0    4546.5    14955.93300     -0.00342     14955.9364    168.7013      28460.13
!
 38.   2  1  1   1  1  0   4  2   2  2    2224.7    14955.68150      0.00038     14955.6811     82.5492       6814.36
 39.   2  1  1   1  1  0   4  2   2  2    3045.1    14955.48480      0.00435     14955.4804    112.9907      12766.90
 40.   2  1  1   1  1  0   4  2   2  2    3646.7    14955.29630      0.00244     14955.2939    135.3135      18309.76
 41.   2  1  1   1  1  0   4  2   2  2    4546.5    14954.95090     -0.00196     14954.9529    168.7013      28460.13

 Standard deviation =        0.002291


 ITERATION NO = 3 CONSTANTS, deviations and changes:

     Mu.a      =         0.358858474 +-      0.000303856           0.000000000
     Mu.b      =         1.281948710 +-      0.000593686           0.000000000


 FINAL RESULTS OF LEAST SQUARES FITTING PROCEDURE
 ================================================

 FITTED CONSTANTS:

 A     /MHz       26401.4764974393      1:Xab /MHz           0.
 B     /MHz        3802.15317891442     1:XJ.a/kHz           0.
 C     /MHz        3549.45832788319     1:XK.a/kHz           0.
 DJ    /kHz           0.97882481121886  1:XJbc/kHz           0.
 DJK   /kHz          -0.65770733393743  1:Ma  /MHz           0.
 DK    /kHz         151.280382690001    1:Mb-c/MHz           0.
 dJ    /kHz           0.12547510773005  1:Tr  /MHz           0.
 dK    /kHz          -6.31899120921345  1:Xd  /kHz           0.
 HJ    / Hz          -0.0273188744555   1:Xbc /MHz           0.
 HJK   / Hz           0.                1:Xac /MHz           0.
 HKJ   / Hz        -132.685651099522
 HK    / Hz           0.
 hJ    / Hz          -0.0075759732997
 hJK   / Hz           0.                Mu.a  /D             0.35885(30)
 hK    / Hz         336.244125489832    Mu.b  /D             1.28194(59)
 LKKJ  /mHz           0.                Mu.c  /D             0.
 1:Xa  /MHz           0.                 d    /cm           26.95
 1:Xb-c/MHz           0.                 k    /cm            0.
```

```
 CORRELATION COEFFICIENTS:

          Mu.a     Mu.b

 Mu.a     1.0000
 Mu.b    -0.2953   1.0000


 WORST FITTED LINES:

      J K  K  <- J K  K     F MF<- F MF    Volts        Obs          Obs-Calc       Calc          Field      Field**2
         -1 +1      -1 +1

 17.   2  0  2   1  0  1    4  2   2  2   12393.4    14701.33590      0.00465     14701.3313    459.8664   211477.12
 21.   2  0  2   1  0  1    4  0   2  0    7641.1    14700.78710     -0.00458     14700.7917    283.5288    80388.56
 39.   2  1  1   1  1  0    4  2   2  2    3045.1    14955.48480      0.00435     14955.4804    112.9907    12766.90
 34.   2  1  1   1  1  0    4  0   2  0    3045.1    14955.92650      0.00396     14955.9225    112.9907    12766.90
 16.   2  0  2   1  0  1    4  2   2  2   11349.8    14701.29470      0.00344     14701.2913    421.1429   177361.31


 CONTRIBUTIONS (from gradients) OF INDIVIDUAL CONSTANTS TO FREQUENCY:

                                                                          Mu.a     Mu.b

!
! 2006 measurements, Ar carrier d=26.950
! 2022 measurements, Ne carrier d=26.9727 and the actually applied voltages
!                        have been corrected to d=26.950 by
!                        V_old =  V_new *  ( d_old/d_new ) = V_new * 0.999158
!                        where 0.999158 = 26.950/26.9727
!
! Rotational and cd constants are from the ASFIT fit of
! Dreizler+Scappini 1981 data and IFPAN BF-FTMW + WG-FTMW + MMW
! lines (to average frequencies in cases of resolved A,E splitting)
!
!--------------------------------------------------
!  101 <- 000
!--------------------------------------------------
!
  1.   1  0  1   0  0  0    2  0   0  0       0.0     7351.6088     0.00000  0.00000
!
  2.   1  0  1   0  0  0    2  0   0  0    4391.3     7351.7336     0.06286  0.06337
  3.   1  0  1   0  0  0    2  0   0  0    6244.8     7351.8647     0.12711  0.12815
  4.   1  0  1   0  0  0    2  0   0  0    7645.7     7351.9905     0.19053  0.19209
  5.   1  0  1   0  0  0    2  0   0  0    8777.4     7352.1148     0.25111  0.25316
  6.   1  0  1   0  0  0    2  0   0  0   10005.6     7352.2622     0.32628  0.32896
  7.   1  0  1   0  0  0    2  0   0  0   11349.8     7352.4499     0.41982  0.42326
  8.   1  0  1   0  0  0    2  0   0  0   12400.3     7352.6134     0.50111  0.50522
!
!--------------------------------------------------
!  202 <- 101
!--------------------------------------------------
!
  9.   2  0  2   1  0  1    4  0   2  0       0.0    14701.0839     0.00000  0.00000
!
 10.   2  0  2   1  0  1    4  2   2  2    2991.3    14701.0989     0.00677  0.00767
 11.   2  0  2   1  0  1    4  2   2  2    4390.5    14701.1146     0.01459  0.01652
 12.   2  0  2   1  0  1    4  2   2  2    6232.3    14701.1451     0.02939  0.03329
 13.   2  0  2   1  0  1    4  2   2  2    7641.1    14701.1763     0.04418  0.05005
 14.   2  0  2   1  0  1    4  2   2  2    8778.3    14701.2063     0.05831  0.06606
 15.   2  0  2   1  0  1    4  2   2  2   10003.9    14701.2436     0.07572  0.08580
 16.   2  0  2   1  0  1    4  2   2  2   11349.8    14701.2947     0.09747  0.11045
 17.   2  0  2   1  0  1    4  2   2  2   12393.4    14701.3359     0.11621  0.13171
!
 18.   2  0  2   1  0  1    4  0   2  0    2994.2    14701.0358    -0.00835 -0.03644
 19.   2  0  2   1  0  1    4  0   2  0    4395.3    14700.9835    -0.01799 -0.07852
 20.   2  0  2   1  0  1    4  0   2  0    6239.3    14700.8866    -0.03625 -0.15821
 21.   2  0  2   1  0  1    4  0   2  0    7641.1    14700.7871    -0.05437 -0.23726
 22.   2  0  2   1  0  1    4  0   2  0    8783.8    14700.6951    -0.07184 -0.31350
 23.   2  0  2   1  0  1    4  0   2  0   10014.6    14700.5795    -0.09338 -0.40747
 24.   2  0  2   1  0  1    4  0   2  0   11349.8    14700.4395    -0.11993 -0.52329
 25.   2  0  2   1  0  1    4  0   2  0   12393.4    14700.3177    -0.14299 -0.62387
!
!
!--------------------------------------------------
!  212 <- 111
!--------------------------------------------------
!
 26.   2  1  2   1  1  1    4  0   2  0       0.0    14450.4753     0.00000  0.00000
!
 27.   2  1  2   1  1  1    4  0   2  0    1628.4    14450.9026     0.00204  0.42141
 28.   2  1  2   1  1  1    4  0   2  0    2027.9    14451.1319     0.00317  0.65242
```

```
 29.   2  1  2   1  1  1    4  0   2  0    2423.6    14451.4122     0.00452  0.92994
 30.   2  1  2   1  1  1    4  0   2  0    2736.4    14451.6713     0.00576  1.18324
 31.   2  1  2   1  1  1    4  0   2  0    3030.3    14451.9374     0.00707  1.44819
!
!-------------------------------------------------
!  211 <- 110
!-------------------------------------------------
!
 32.   2  1  1   1  1  0    4  0   2  0       0.0    14955.9097     0.00000  0.00000
!
 33.   2  1  1   1  1  0    4  0   2  0    2224.7    14955.9193     0.00368  0.00235
 34.   2  1  1   1  1  0    4  0   2  0    3045.1    14955.9265     0.00690  0.00440
 35.   2  1  1   1  1  0    4  0   2  0    3646.7    14955.9277     0.00989  0.00631
 36.   2  1  1   1  1  0    4  0   2  0    4147.8    14955.9346     0.01280  0.00816
 37.   2  1  1   1  1  0    4  0   2  0    4546.5    14955.9330     0.01538  0.00980
!
 38.   2  1  1   1  1  0    4  2   2  2    2224.7    14955.6815    -0.20901 -0.02090
 39.   2  1  1   1  1  0    4  2   2  2    3045.1    14955.4848    -0.39094 -0.03914
 40.   2  1  1   1  1  0    4  2   2  2    3646.7    14955.2963    -0.55981 -0.05611
 41.   2  1  1   1  1  0    4  2   2  2    4546.5    14954.9509    -0.86772 -0.08713

 ----------------------------------------------------------------------------



////////////////////////////////////////////////////////////////////////////////////////////
//////////////// cut here - please display and print using a fixed width font ///////////////
////////////////////////////////////////////////////////////////////////////////////////////

  ___________________________________________________________________________
 |                                                                           |
 |   QSTARK - Fully diagonalizing fit of Stark shifts in a rotor             |
 |            with zero or one quadrupolar nuclei                            |
 |___________________________________________________________________________|
  version 14.XI.2022                                        Zbigniew KISIEL


 ----------------------------------------------------------------------------
  n-butanol, TGt conformer
 ----------------------------------------------------------------------------

 Calculation for J and F exceeding the value in data by at least 2
 The fit will be made to TRANSITION FREQUENCIES

    74 Lines read in
    74 Lines fitted    --->    6 field free and   68 at non-zero field
     3 Constants fitted

 TRANSITIONS (F and MF in units of 1/2):

      J K  K  <- J K  K      F MF<- F MF    Volts        Obs          Obs-Calc       Calc         Field      Field**2
         -1 +1      -1 +1

!
!  Zero field frequencies are AE averaged state frequencies from
!  the Kawashima et al. JPCA 2022 paper.  Assumed spectroscopic parameters are
!  from refit of the AE-averaged subset from that paper limited to Ka=2.
!
!-----------------------------------------------------------
!  1_11 <- 0_00  bR
!-----------------------------------------------------------
  1.   1  1  1   0  0  0    2  0   0  0       0.0    14657.17380     -0.00087     14657.1747      0.0000         0.00

  2.   1  1  1   0  0  0    2  0   0  0    3031.1    14657.58000      0.00077     14657.5792    112.3562     12623.91
  3.   1  1  1   0  0  0    2  0   0  0    4053.0    14657.89930      0.00170     14657.8976    150.2358     22570.78
  4.   1  1  1   0  0  0    2  0   0  0    5056.4    14658.30090      0.00184     14658.2991    187.4296     35129.84
  5.   1  1  1   0  0  0    2  0   0  0    6068.8    14658.79610      0.00313     14658.7930    224.9570     50605.65
  6.   1  1  1   0  0  0    2  0   0  0    7081.5    14659.37390     -0.00194     14659.3758    262.4956     68903.91
  7.   1  1  1   0  0  0    2  0   0  0    8085.2    14660.03800     -0.00262     14660.0406    299.7005     89820.39

!-----------------------------------------------------------
!  1_10 <- 0_00  cR
!-----------------------------------------------------------
  8.   1  1  0   0  0  0    2  0   0  0       0.0    14839.20240     -0.00133     14839.2037      0.0000         0.00

  9.   1  1  0   0  0  0    2  0   0  0    3027.5    14839.53140     -0.00123     14839.5326    112.2227     12593.94
 10.   1  1  0   0  0  0    2  0   0  0    4041.6    14839.79080      0.00068     14839.7901    149.8132     22443.99
 11.   1  1  0   0  0  0    2  0   0  0    5054.9    14840.12180      0.00031     14840.1215    187.3740     35109.00
 12.   1  1  0   0  0  0    2  0   0  0    6079.3    14840.53150     -0.00051     14840.5320    225.3462     50780.92
 13.   1  1  0   0  0  0    2  0   0  0    7067.6    14841.00210      0.00180     14841.0003    261.9803     68633.68
 14.   1  1  0   0  0  0    2  0   0  0    8086.1    14841.55600     -0.00144     14841.5574    299.7339     89840.38

!-----------------------------------------------------------
```

```
!   2_11<-2_02  bQ
!------------------------------------------------------------
 15.   2  1  1   2  0  2   4  0   4  0       0.0    10462.69900    -0.00047    10462.6995     0.0000        0.00

 16.   2  1  1   2  0  2   4  4   4  4    2023.2    10462.94000     0.00404    10462.9360    74.9956     5624.33
 17.   2  1  1   2  0  2   4  4   4  4    3031.3    10463.23270     0.00247    10463.2302   112.3636    12625.58
 18.   2  1  1   2  0  2   4  4   4  4    4046.1    10463.65350     0.00870    10463.6448   149.9800    22494.00
 19.   2  1  1   2  0  2   4  4   4  4    5054.2    10464.17790     0.00393    10464.1740   187.3480    35099.28

!------------------------------------------------------------
!  3_03 <- 2_02  aR
!------------------------------------------------------------
 20.   3  0  3   2  0  2   6  0   4  0       0.0    13673.15690     0.00006    13673.1568     0.0000        0.00

 21.   3  0  3   2  0  2   6  0   4  0    3038.4    13673.57420     0.00443    13673.5698   112.6268    12684.79
 22.   3  0  3   2  0  2   6  0   4  0    4048.6    13673.89240     0.00256    13673.8898   150.0727    22521.80
 23.   3  0  3   2  0  2   6  0   4  0    5060.5    13674.30500     0.00327    13674.3017   187.5815    35186.84
 24.   3  0  3   2  0  2   6  0   4  0    6066.6    13674.80290     0.00121    13674.8017   224.8755    50568.97
 25.   3  0  3   2  0  2   6  0   4  0    8076.8    13676.06620    -0.00371    13676.0699   299.3891    89633.85

 26.   3  0  3   2  0  2   6  2   4  2    3038.4    13673.51020     0.00435    13673.5058   112.6268    12684.79
 27.   3  0  3   2  0  2   6  2   4  2    4048.6    13673.77590    -0.00062    13673.7765   150.0727    22521.80
 28.   3  0  3   2  0  2   6  2   4  2    5060.5    13674.12420    -0.00081    13674.1250   187.5815    35186.84
 29.   3  0  3   2  0  2   6  2   4  2    6076.2    13674.55290     0.00020    13674.5527   225.2313    50729.14
 30.   3  0  3   2  0  2   6  2   4  2    7077.1    13675.05290     0.00239    13675.0505   262.3325    68818.32
 31.   3  0  3   2  0  2   6  2   4  2    8076.8    13675.62570     0.00231    13675.6234   299.3891    89633.85

 32.   3  0  3   2  0  2   6  4   4  4    3038.4    13673.31930     0.00559    13673.3137   112.6268    12684.79
 33.   3  0  3   2  0  2   6  4   4  4    4048.6    13673.43590     0.00054    13673.4354   150.0727    22521.80
 34.   3  0  3   2  0  2   6  4   4  4    5060.5    13673.59220     0.00021    13673.5920   187.5815    35186.84
 35.   3  0  3   2  0  2   6  4   4  4    6076.2    13673.78430     0.00011    13673.7842   225.2313    50729.14
 36.   3  0  3   2  0  2   6  4   4  4    7076.8    13674.01080     0.00299    13674.0078   262.3213    68812.48
 37.   3  0  3   2  0  2   6  4   4  4    8076.8    13674.25940    -0.00589    13674.2653   299.3891    89633.85

! Delta M = +1
 38.   3  0  3   2  0  2   6  4   4  2    5060.5    13673.85510    -0.00153    13673.8566   187.5815    35186.84
 39.   3  0  3   2  0  2   6  4   4  2    6076.2    13674.16390    -0.00188    13674.1658   225.2313    50729.14
 40.   3  0  3   2  0  2   6  4   4  2    7076.8    13674.52610     0.00058    13674.5255   262.3213    68812.48
 41.   3  0  3   2  0  2   6  4   4  2    8076.8    13674.93510    -0.00469    13674.9398   299.3891    89633.85

 42.   3  0  3   2  0  2   6  6   4  4    3038.4    13673.15220    -0.00025    13673.1524   112.6268    12684.79
 43.   3  0  3   2  0  2   6  6   4  4    4048.6    13673.14400    -0.00504    13673.1490   150.0727    22521.80
 44.   3  0  3   2  0  2   6  6   4  4    6066.2    13673.13890    -0.00044    13673.1393   224.8606    50562.30
 45.   3  0  3   2  0  2   6  6   4  4    7074.9    13673.13620     0.00317    13673.1330   262.2509    68775.54
 46.   3  0  3   2  0  2   6  6   4  4    8076.8    13673.13020     0.00438    13673.1258   299.3891    89633.85

!------------------------------------------------------------
!  3_12 <- 2_11  aR
!------------------------------------------------------------
 47.   3  1  2   2  1  1   6  0   4  0       0.0    13954.44580     0.00179    13954.4440     0.0000        0.00

 48.   3  1  2   2  1  1   6  0   4  0    4043.2    13954.47500    -0.00197    13954.4770   149.8725    22461.76
 49.   3  1  2   2  1  1   6  0   4  0    5060.8    13954.49240    -0.00324    13954.4956   187.5927    35191.01
 50.   3  1  2   2  1  1   6  0   4  0    6057.1    13954.52400     0.00605    13954.5180   224.5233    50410.72
 51.   3  1  2   2  1  1   6  0   4  0    7071.1    13954.54380    -0.00096    13954.5448   262.1100    68701.68
 52.   3  1  2   2  1  1   6  0   4  0    8085.6    13954.58060     0.00490    13954.5757   299.7153    89829.27

 53.   3  1  2   2  1  1   6  2   4  2    4043.2    13954.33530    -0.00188    13954.3372   149.8725    22461.76
 54.   3  1  2   2  1  1   6  2   4  2    5060.8    13954.27590    -0.00077    13954.2767   187.5927    35191.01
 55.   3  1  2   2  1  1   6  2   4  2    6057.1    13954.20150    -0.00286    13954.2044   224.5233    50410.72
 56.   3  1  2   2  1  1   6  2   4  2    7071.1    13954.11820     0.00070    13954.1175   262.1100    68701.68
 57.   3  1  2   2  1  1   6  2   4  2    8085.6    13954.01610    -0.00113    13954.0172   299.7153    89829.27

 58.   3  1  2   2  1  1   6  4   4  4    2020.0    13954.31470     0.00203    13954.3127    74.8769     5606.56
 59.   3  1  2   2  1  1   6  4   4  4    4043.2    13953.91440    -0.00388    13953.9183   149.8725    22461.76
 60.   3  1  2   2  1  1   6  4   4  4    5060.8    13953.61880    -0.00211    13953.6209   187.5927    35191.01
 61.   3  1  2   2  1  1   6  4   4  4    6057.1    13953.27370     0.00779    13953.2659   224.5233    50410.72
 62.   3  1  2   2  1  1   6  4   4  4    7075.6    13952.83780    -0.00020    13952.8380   262.2769    68789.15

! Delta M = +1
 63.   3  1  2   2  1  1   6  6   4  4    4043.2    13954.06350    -0.00472    13954.0682   149.8725    22461.76
 64.   3  1  2   2  1  1   6  6   4  4    5060.8    13953.86270     0.00688    13953.8558   187.5927    35191.01
 65.   3  1  2   2  1  1   6  6   4  4    6064.7    13953.60510     0.00481    13953.6003   224.8050    50537.30

!------------------------------------------------------------
!   3_13<-2_12  aR
!------------------------------------------------------------
 66.   3  1  3   2  1  2   6  0   4  0       0.0    13408.40100    -0.00023    13408.4012     0.0000        0.00

 67.   3  1  3   2  1  2   6  0   4  0    3038.8    13408.42560     0.00388    13408.4217   112.6416    12688.13

 68.   3  1  3   2  1  2   6  2   4  2    3038.8    13408.49490     0.00369    13408.4912   112.6416    12688.13
 69.   3  1  3   2  1  2   6  2   4  2    4043.7    13408.56290     0.00235    13408.5606   149.8910    22467.32
```

```
70.   3  1  3   2  1  2    6  2   4  2    5054.9    13408.64900     -0.00116     13408.6502    187.3740    35109.00

71.   3  1  3   2  1  2    6  4   4  4    2028.4    13408.53570      0.00150     13408.5342     75.1883     5653.28
72.   3  1  3   2  1  2    6  4   4  4    3038.8    13408.70180      0.00224     13408.6996    112.6416    12688.13
73.   3  1  3   2  1  2    6  4   4  4    4043.7    13408.92900     -0.00022     13408.9292    149.8910    22467.32
74.   3  1  3   2  1  2    6  4   4  4    5054.9    13409.22540     -0.00034     13409.2257    187.3740    35109.00

Standard deviation =       0.003153


ITERATION NO = 3 CONSTANTS, deviations and changes:

    Mu.a     =         0.713680449 +-      0.000365989           0.000000001
    Mu.b     =         0.898876976 +-      0.000442430           0.000000000
    Mu.c     =         0.807144715 +-      0.000547181           0.000000000


FINAL RESULTS OF LEAST SQUARES FITTING PROCEDURE
================================================

FITTED CONSTANTS:

A     /MHz       12467.7415597117      1:Xab /MHz           0.
B     /MHz        2371.5177110614      1:XJ.a/kHz           0.
C     /MHz        2189.4793994294      1:XK.a/kHz           0.
DJ    /kHz           0.756259417       1:XJbc/kHz           0.
DJK   /kHz          -6.180283871       1:Ma  /MHz           0.
DK    /kHz          60.254623227       1:Mb-c/MHz           0.
dJ    /kHz           0.124839613       1:Tr  /MHz           0.
dK    /kHz           2.063149976       1:Xd  /kHz           0.
HJ    / Hz           0.                1:Xbc /MHz           0.
HJK   / Hz           0.                1:Xac /MHz           0.
HKJ   / Hz           0.
HK    / Hz           0.
hJ    / Hz           0.
hJK   / Hz           0.                Mu.a  /D             0.71368(36)
hK    / Hz           0.                Mu.b  /D             0.89887(44)
LKKJ  /mHz           0.                Mu.c  /D             0.80714(54)
1:Xa  /MHz           0.                 d    /cm           26.9776
1:Xb-c/MHz           0.                 k    /cm            0.


CORRELATION COEFFICIENTS:

         Mu.a     Mu.b     Mu.c

Mu.a     1.0000
Mu.b    -0.2588   1.0000
Mu.c    -0.0867   0.7003   1.0000


WORST FITTED LINES:

     J K  K  <- J K  K     F MF<-  F MF    Volts        Obs          Obs-Calc       Calc          Field      Field**2
        -1 +1      -1 +1

18.   2  1  1   2  0  2    4  4   4  4    4046.1    10463.65350      0.00870     10463.6448    149.9800    22494.00
61.   3  1  2   2  1  1    6  4   4  4    6057.1    13953.27370      0.00779     13953.2659    224.5233    50410.72
64.   3  1  2   2  1  1    6  6   4  4    5060.8    13953.86270      0.00688     13953.8558    187.5927    35191.01
50.   3  1  2   2  1  1    6  0   4  0    6057.1    13954.52400      0.00605     13954.5180    224.5233    50410.72
37.   3  0  3   2  0  2    6  4   4  4    8076.8    13674.25940     -0.00589     13674.2653    299.3891    89633.85


CONTRIBUTIONS (from gradients) OF INDIVIDUAL CONSTANTS TO FREQUENCY:

                                                                          Mu.a     Mu.b     Mu.c


!
!  Zero field frequencies are AE averaged state frequencies from
!  the Kawashima et al. JPCA 2022 paper.  Assumed spectroscopic parameters are
!  from refit of the AE-averaged subset from that paper limited to Ka=2.
!
!--------------------------------------------------------------
!  1_11 <- 0_00  bR
!--------------------------------------------------------------
  1.   1  1  1   0  0  0    2  0   0  0       0.0    14657.1738     0.00000  0.00000  0.00000

  2.   1  1  1   0  0  0    2  0   0  0    3031.1    14657.5800     0.08264  0.28555 -0.03609
  3.   1  1  1   0  0  0    2  0   0  0    4053.0    14657.8993     0.14775  0.51008 -0.06418
  4.   1  1  1   0  0  0    2  0   0  0    5056.4    14658.3009     0.22996  0.79298 -0.09921
  5.   1  1  1   0  0  0    2  0   0  0    6068.8    14658.7961     0.33127  1.14067 -0.14171
  6.   1  1  1   0  0  0    2  0   0  0    7081.5    14659.3739     0.45106  1.55048 -0.19102
```

```
  7.   1  1  1   0  0  0    2  0   0  0    8085.2    14660.0380     0.58800  2.01721 -0.24613

!-----------------------------------------------------------
!  1_10 <- 0_00  cR
!-----------------------------------------------------------
  8.   1  1  0   0  0  0    2  0   0  0       0.0    14839.2024     0.00000  0.00000  0.00000

  9.   1  1  0   0  0  0    2  0   0  0    3027.5    14839.5314     0.08386  0.04581 -0.19942
 10.   1  1  0   0  0  0    2  0   0  0    4041.6    14839.7908     0.14944  0.08189 -0.35560
 11.   1  1  0   0  0  0    2  0   0  0    5054.9    14840.1218     0.23376  0.12862 -0.55669
 12.   1  1  0   0  0  0    2  0   0  0    6079.3    14840.5315     0.33809  0.18698 -0.80596
 13.   1  1  0   0  0  0    2  0   0  0    7067.6    14841.0021     0.45693  0.25417 -1.09050
 14.   1  1  0   0  0  0    2  0   0  0    8086.1    14841.5560     0.59808  0.33496 -1.42928

!-----------------------------------------------------------
!   2_11<-2_02  bQ
!-----------------------------------------------------------
 15.   2  1  1   2  0  2    4  0   4  0       0.0    10462.6990     0.00000  0.00000  0.00000

 16.   2  1  1   2  0  2    4  4   4  4    2023.2    10462.9400     0.14861  0.07102 -0.01682
 17.   2  1  1   2  0  2    4  4   4  4    3031.3    10463.2327     0.33336  0.15939 -0.03781
 18.   2  1  1   2  0  2    4  4   4  4    4046.1    10463.6535     0.59334  0.28388 -0.06746
 19.   2  1  1   2  0  2    4  4   4  4    5054.2    10464.1779     0.92465  0.44280 -0.10546

!-----------------------------------------------------------
!  3_03 <- 2_02  aR
!-----------------------------------------------------------
 20.   3  0  3   2  0  2    6  0   4  0       0.0    13673.1569     0.00000  0.00000  0.00000

 21.   3  0  3   2  0  2    6  0   4  0    3038.4    13673.5742    -0.00911  0.24784 -0.17408
 22.   3  0  3   2  0  2    6  0   4  0    4048.6    13673.8924    -0.01615  0.43984 -0.30896
 23.   3  0  3   2  0  2    6  0   4  0    5060.5    13674.3050    -0.02520  0.68678 -0.48245
 24.   3  0  3   2  0  2    6  0   4  0    6066.6    13674.8029    -0.03616  0.98631 -0.69292
 25.   3  0  3   2  0  2    6  0   4  0    8076.8    13676.0662    -0.06385  1.74511 -1.22625

 26.   3  0  3   2  0  2    6  2   4  2    3038.4    13673.5102    -0.00249  0.20503 -0.14648
 27.   3  0  3   2  0  2    6  2   4  2    4048.6    13673.7759    -0.00433  0.36398 -0.26005
 28.   3  0  3   2  0  2    6  2   4  2    5060.5    13674.1242    -0.00656  0.56858 -0.40623
 29.   3  0  3   2  0  2    6  2   4  2    6076.2    13674.5529    -0.00911  0.81956 -0.58556
 30.   3  0  3   2  0  2    6  2   4  2    7077.1    13675.0529    -0.01181  1.11154 -0.79419
 31.   3  0  3   2  0  2    6  2   4  2    8076.8    13675.6257    -0.01455  1.44736 -1.03416

 32.   3  0  3   2  0  2    6  4   4  4    3038.4    13673.3193     0.01710  0.07629 -0.06349
 33.   3  0  3   2  0  2    6  4   4  4    4048.6    13673.4359     0.03035  0.13545 -0.11272
 34.   3  0  3   2  0  2    6  4   4  4    5060.5    13673.5922     0.04743  0.21161 -0.17610
 35.   3  0  3   2  0  2    6  4   4  4    6076.2    13673.7843     0.06838  0.30507 -0.25388
 36.   3  0  3   2  0  2    6  4   4  4    7076.8    13674.0108     0.09277  0.41381 -0.34437
 37.   3  0  3   2  0  2    6  4   4  4    8076.8    13674.2594     0.12085  0.53900 -0.44855

! Delta M = +1
 38.   3  0  3   2  0  2    6  4   4  2    5060.5    13673.8551    -0.02316  0.43729 -0.28574
 39.   3  0  3   2  0  2    6  4   4  2    6076.2    13674.1639    -0.03304  0.63029 -0.41187
 40.   3  0  3   2  0  2    6  4   4  2    7076.8    13674.5261    -0.04425  0.85472 -0.55854
 41.   3  0  3   2  0  2    6  4   4  2    8076.8    13674.9351    -0.05681  1.11298 -0.72731

 42.   3  0  3   2  0  2    6  6   4  4    3038.4    13673.1522     0.00712 -0.00260  0.00891
 43.   3  0  3   2  0  2    6  6   4  4    4048.6    13673.1440     0.01264 -0.00462  0.01582
 44.   3  0  3   2  0  2    6  6   4  4    6066.2    13673.1389     0.02838 -0.01037  0.03551
 45.   3  0  3   2  0  2    6  6   4  4    7074.9    13673.1362     0.03860 -0.01410  0.04829
 46.   3  0  3   2  0  2    6  6   4  4    8076.8    13673.1302     0.05031 -0.01838  0.06293

!-----------------------------------------------------------
!  3_12 <- 2_11  aR
!-----------------------------------------------------------
 47.   3  1  2   2  1  1    6  0   4  0       0.0    13954.4458     0.00000  0.00000  0.00000

 48.   3  1  2   2  1  1    6  0   4  0    4043.2    13954.4750    -0.00445 -0.00060 -0.03799
 49.   3  1  2   2  1  1    6  0   4  0    5060.8    13954.4924    -0.00697 -0.00094 -0.05952
 50.   3  1  2   2  1  1    6  0   4  0    6057.1    13954.5240    -0.00999 -0.00136 -0.08524
 51.   3  1  2   2  1  1    6  0   4  0    7071.1    13954.5438    -0.01361 -0.00187 -0.11614
 52.   3  1  2   2  1  1    6  0   4  0    8085.6    13954.5806    -0.01780 -0.00247 -0.15181

 53.   3  1  2   2  1  1    6  2   4  2    4043.2    13954.3353    -0.12606 -0.01838 -0.03764
 54.   3  1  2   2  1  1    6  2   4  2    5060.8    13954.2759    -0.19741 -0.02878 -0.05894
 55.   3  1  2   2  1  1    6  2   4  2    6057.1    13954.2015    -0.28265 -0.04119 -0.08438
 56.   3  1  2   2  1  1    6  2   4  2    7071.1    13954.1182    -0.38498 -0.05609 -0.11492
 57.   3  1  2   2  1  1    6  2   4  2    8085.6    13954.0161    -0.50303 -0.07328 -0.15013

 58.   3  1  2   2  1  1    6  4   4  4    2020.0    13954.3147    -0.12253 -0.01791 -0.00914
 59.   3  1  2   2  1  1    6  4   4  4    4043.2    13953.9144    -0.48991 -0.07164 -0.03645
 60.   3  1  2   2  1  1    6  4   4  4    5060.8    13953.6188    -0.76636 -0.11207 -0.05691
 61.   3  1  2   2  1  1    6  4   4  4    6057.1    13953.2737    -1.09579 -0.16027 -0.08118
 62.   3  1  2   2  1  1    6  4   4  4    7075.6    13952.8378    -1.49199 -0.21824 -0.11021
```

```
! Delta M = +1
 63.   3  1  2   2  1  1    6  6   4  4    4043.2    13954.0635    -0.41255  0.00748 -0.02991
 64.   3  1  2   2  1  1    6  6   4  4    5060.8    13953.8627    -0.64521  0.01188 -0.04670
 65.   3  1  2   2  1  1    6  6   4  4    6064.7    13953.6051    -0.92462  0.01732 -0.06677

!------------------------------------------------------------
!   3_13<-2_12  aR
!------------------------------------------------------------
 66.   3  1  3   2  1  2    6  0   4  0       0.0    13408.4010     0.00000  0.00000  0.00000

 67.   3  1  3   2  1  2    6  0   4  0    3038.8    13408.4256    -0.00262  0.02309 -0.00002

 68.   3  1  3   2  1  2    6  2   4  2    3038.8    13408.4949     0.07457  0.02342  0.00801
 69.   3  1  3   2  1  2    6  2   4  2    4043.7    13408.5629     0.13200  0.04146  0.01417
 70.   3  1  3   2  1  2    6  2   4  2    5054.9    13408.6490     0.20619  0.06477  0.02212

 71.   3  1  3   2  1  2    6  4   4  4    2028.4    13408.5357     0.13636  0.01088  0.01431
 72.   3  1  3   2  1  2    6  4   4  4    3038.8    13408.7018     0.30581  0.02439  0.03207
 73.   3  1  3   2  1  2    6  4   4  4    4043.7    13408.9290     0.54093  0.04311  0.05668
 74.   3  1  3   2  1  2    6  4   4  4    5054.9    13409.2254     0.84413  0.06721  0.08837

 ------------------------------------------------------------------------------




//////////////////////////////////////////////////////////////////////////////////////////////
//////////////// cut here - please display and print using a fixed width font ///////////////
//////////////////////////////////////////////////////////////////////////////////////////////

  ____________________________________________________________________________
 |                                                                            |
 |   QSTARK - Fully diagonalizing fit of Stark shifts in a rotor              |
 |            with zero or one quadrupolar nuclei                             |
 |____________________________________________________________________________|
  version 14.XI.2022                                           Zbigniew KISIEL


 ------------------------------------------------------------------------------
 GTgp final fit, Ne carrier
 ------------------------------------------------------------------------------

 Calculation for J and F exceeding the value in data by at least 2
 The fit will be made to TRANSITION FREQUENCIES

    36 Lines read in
    36 Lines fitted    --->    5 field free and   31 at non-zero field
     3 Constants fitted

 TRANSITIONS (F and MF in units of 1/2):


      J K  K  <- J K  K     F MF<- F MF    Volts        Obs          Obs-Calc       Calc         Field      Field**2
         -1 +1      -1 +1

!
!  Zero field frequencies are frequencies from the Kawashima et al.
!  JPCA 2022 paper, assumed spectroscopic parameters are from refit of
!  the AE-state averaged frequencies from that paper
!
!------------------------------------------------------------
!  1_10 <- 0_00 c.R
!------------------------------------------------------------
  1.   1  1  0   0  0  0    2  0   0  0       0.0    14635.50060      0.00011     14635.5005      0.0000         0.00

  2.   1  1  0   0  0  0    2  0   0  0    2025.7    14635.68520      0.00296     14635.6822     75.1019      5640.29
  3.   1  1  0   0  0  0    2  0   0  0    3025.5    14635.91110      0.00509     14635.9060    112.1690     12581.88
  4.   1  1  0   0  0  0    2  0   0  0    4038.1    14636.22250     -0.00060     14636.2231    149.7106     22413.27
  5.   1  1  0   0  0  0    2  0   0  0    5063.5    14636.63230     -0.00481     14636.6371    187.7268     35241.37
  6.   1  1  0   0  0  0    2  0   0  0    6068.2    14637.13520      0.00153     14637.1337    224.9756     50614.03

!------------------------------------------------------------
!  1_11 <- 0_00 b.R
!------------------------------------------------------------
  7.   1  1  1   0  0  0    2  0   0  0       0.0    14451.16880     -0.00048     14451.1693      0.0000         0.00

  8.   1  1  1   0  0  0    2  0   0  0    1512.4    14451.31920      0.00295     14451.3162     56.0715      3144.01
  9.   1  1  1   0  0  0    2  0   0  0    2031.5    14451.43920      0.00478     14451.4344     75.3169      5672.63
 10.   1  1  1   0  0  0    2  0   0  0    2530.7    14451.58670      0.00602     14451.5807     93.8245      8803.04
 11.   1  1  1   0  0  0    2  0   0  0    3025.8    14451.75620     -0.00111     14451.7573    112.1801     12584.37
 12.   1  1  1   0  0  0    2  0   0  0    3529.5    14451.97380      0.00456     14451.9692    130.8545     17122.91
 13.   1  1  1   0  0  0    2  0   0  0    4044.7    14452.21990      0.00030     14452.2196    149.9553     22486.60
```

```
 14.   1  1  1   0  0  0   2  0   0  0    4548.8    14452.49460     -0.00281     14452.4974    168.6446    28441.00
 15.   1  1  1   0  0  0   2  0   0  0    5048.9    14452.80220     -0.00286     14452.8051    187.1856    35038.43

!------------------------------------------------------------
!  1_10 <- 1_01 b.Q
!------------------------------------------------------------
 16.   1  1  0   1  0  1   2  0   2  0       0.0    10158.69090     -0.00126     10158.6922      0.0000        0.00

 17.   1  1  0   1  0  1   2  2   2  2    1010.0    10159.30100      0.00535     10159.2957     37.4453     1402.15
 18.   1  1  0   1  0  1   2  2   2  2    1523.1    10160.06030      0.00071     10160.0596     56.4682     3188.66
 19.   1  1  0   1  0  1   2  2   2  2    2025.8    10161.09830     -0.00100     10161.0993     75.1056     5640.85

!------------------------------------------------------------
!  3_03 <- 2_02 a.R
!------------------------------------------------------------
 20.   3  0  3   2  0  2   6  0   4  0       0.0    13420.23590      0.00001     13420.2359      0.0000        0.00

 21.   3  0  3   2  0  2   6  0   4  0    3044.0    13420.66420      0.00172     13420.6625    112.8548    12736.22
 22.   3  0  3   2  0  2   6  0   4  0    4034.5    13420.98680      0.00167     13420.9851    149.5772    22373.33
 23.   3  0  3   2  0  2   6  0   4  0    5057.7    13421.41490      0.00185     13421.4131    187.5118    35160.68

 24.   3  0  3   2  0  2   6  2   4  2    3044.0    13420.60870      0.00456     13420.6041    112.8548    12736.22
 25.   3  0  3   2  0  2   6  2   4  2    4034.5    13420.88040     -0.00251     13420.8829    149.5772    22373.33
 26.   3  0  3   2  0  2   6  2   4  2    5057.7    13421.24820     -0.00477     13421.2530    187.5118    35160.68

 27.   3  0  3   2  0  2   6  4   4  4    3044.0    13420.43360      0.00517     13420.4284    112.8548    12736.22
 28.   3  0  3   2  0  2   6  4   4  4    4034.5    13420.57670      0.00257     13420.5741    149.5772    22373.33
 29.   3  0  3   2  0  2   6  4   4  4    5057.7    13420.76700     -0.00045     13420.7674    187.5118    35160.68

!------------------------------------------------------------
!  3_13 <- 2_12 a.R
!------------------------------------------------------------
 30.   3  1  3   2  1  2   6  0   4  0       0.0    13152.31780      0.00037     13152.3174      0.0000        0.00

 31.   3  1  3   2  1  2   6  0   4  0    2032.9    13152.32820     -0.00084     13152.3290     75.3688     5680.46
 32.   3  1  3   2  1  2   6  0   4  0    3035.8    13152.34580      0.00247     13152.3433    112.5508    12667.69

 33.   3  1  3   2  1  2   6  2   4  2    2032.9    13152.40940      0.00347     13152.4059     75.3688     5680.46
 34.   3  1  3   2  1  2   6  2   4  2    3035.8    13152.51680      0.00206     13152.5147    112.5508    12667.69

 35.   3  1  3   2  1  2   6  4   4  4    2032.9    13152.63650      0.00009     13152.6364     75.3688     5680.46
 36.   3  1  3   2  1  2   6  4   4  4    3035.8    13153.02550     -0.00264     13153.0281    112.5508    12667.69

 Standard deviation =       0.003120


 ITERATION NO = 3 CONSTANTS, deviations and changes:

     Mu.a     =         1.088135266 +-      0.000587385           0.000000000
     Mu.b     =         1.030426294 +-      0.000920619           0.000000000
     Mu.c     =         0.697987849 +-      0.001407785           0.000000000


 FINAL RESULTS OF LEAST SQUARES FITTING PROCEDURE
 ================================================

 FITTED CONSTANTS:

 A     /MHz       12305.00007          1:Xab /MHz           0.
 B     /MHz        2330.57664          1:XJ.a/kHz           0.
 C     /MHz        2146.23454          1:XK.a/kHz           0.
 DJ    /kHz           0.7131           1:XJbc/kHz           0.
 DJK   /kHz          -2.543            1:Ma  /MHz           0.
 DK    /kHz          73.01             1:Mb-c/MHz           0.
 dJ    /kHz           0.13705          1:Tr  /MHz           0.
 dK    /kHz           2.45             1:Xd  /kHz           0.
 HJ    / Hz           0.               1:Xbc /MHz           0.
 HJK   / Hz           0.               1:Xac /MHz           0.
 HKJ   / Hz           0.
 HK    / Hz           0.
 hJ    / Hz           0.
 hJK   / Hz           0.               Mu.a  /D             1.08813(58)
 hK    / Hz           0.               Mu.b  /D             1.03042(92)
 LKKJ  /mHz           0.               Mu.c  /D             0.6979(14)
 1:Xa  /MHz           0.                d    /cm           26.9727
 1:Xb-c/MHz           0.                k    /cm            0.


 CORRELATION COEFFICIENTS:

          Mu.a     Mu.b     Mu.c

 Mu.a     1.0000
```

```
 Mu.b    -0.1140   1.0000
 Mu.c     0.2136   0.5636   1.0000


 WORST FITTED LINES:

      J K  K  <- J K  K      F MF<- F MF    Volts        Obs          Obs-Calc       Calc         Field      Field**2
         -1 +1      -1 +1

 10.   1  1  1   0  0  0    2  0   0  0    2530.7    14451.58670      0.00602     14451.5807     93.8245     8803.04
 17.   1  1  0   1  0  1    2  2   2  2    1010.0    10159.30100      0.00535     10159.2957     37.4453     1402.15
 27.   3  0  3   2  0  2    6  4   4  4    3044.0    13420.43360      0.00517     13420.4284    112.8548    12736.22
  3.   1  1  0   0  0  0    2  0   0  0    3025.5    14635.91110      0.00509     14635.9060    112.1690    12581.88
  5.   1  1  0   0  0  0    2  0   0  0    5063.5    14636.63230     -0.00481     14636.6371    187.7268    35241.37


 CONTRIBUTIONS (from gradients) OF INDIVIDUAL CONSTANTS TO FREQUENCY:

                                                                         Mu.a     Mu.b     Mu.c


!
!  Zero field frequencies are frequencies from the Kawashima et al.
!  JPCA 2022 paper, assumed spectroscopic parameters are from refit of
!  the AE-state subset from that paper
!
!------------------------------------------------------------
!  1_10 <- 0_00 c.R
!------------------------------------------------------------
  1.   1  1  0   0  0  0    2  0   0  0       0.0    14635.5006     0.00000  0.00000  0.00000

  2.   1  1  0   0  0  0    2  0   0  0    2025.7    14635.6852     0.08897  0.02727 -0.06555
  3.   1  1  0   0  0  0    2  0   0  0    3025.5    14635.9111     0.19845  0.06092 -0.14631
  4.   1  1  0   0  0  0    2  0   0  0    4038.1    14636.2225     0.35348  0.10876 -0.26085
  5.   1  1  0   0  0  0    2  0   0  0    5063.5    14636.6323     0.55572  0.17150 -0.41060
  6.   1  1  0   0  0  0    2  0   0  0    6068.2    14637.1352     0.79801  0.24714 -0.59049

!------------------------------------------------------------
!  1_11 <- 0_00 b.R
!------------------------------------------------------------
  7.   1  1  1   0  0  0    2  0   0  0       0.0    14451.1688     0.00000  0.00000  0.00000

  8.   1  1  1   0  0  0    2  0   0  0    1512.4    14451.3192     0.04873  0.09137 -0.00685
  9.   1  1  1   0  0  0    2  0   0  0    2031.5    14451.4392     0.08792  0.16482 -0.01234
 10.   1  1  1   0  0  0    2  0   0  0    2530.7    14451.5867     0.13643  0.25572 -0.01910
 11.   1  1  1   0  0  0    2  0   0  0    3025.8    14451.7562     0.19503  0.36546 -0.02724
 12.   1  1  1   0  0  0    2  0   0  0    3529.5    14451.9738     0.26536  0.49709 -0.03696
 13.   1  1  1   0  0  0    2  0   0  0    4044.7    14452.2199     0.34848  0.65254 -0.04836
 14.   1  1  1   0  0  0    2  0   0  0    4548.8    14452.4946     0.44075  0.82496 -0.06093
 15.   1  1  1   0  0  0    2  0   0  0    5048.9    14452.8022     0.54297  1.01581 -0.07474

!------------------------------------------------------------
!  1_10 <- 1_01 b.Q
!------------------------------------------------------------
 16.   1  1  0   1  0  1    2  0   2  0       0.0    10158.6909     0.00000  0.00000  0.00000

 17.   1  1  0   1  0  1    2  2   2  2    1010.0    10159.3010     0.56962  0.02016 -0.01197
 18.   1  1  0   1  0  1    2  2   2  2    1523.1    10160.0603     1.28548  0.04592 -0.02720
 19.   1  1  0   1  0  1    2  2   2  2    2025.8    10161.0983     2.25064  0.08138 -0.04805

!------------------------------------------------------------
!  3_03 <- 2_02 a.R
!------------------------------------------------------------
 20.   3  0  3   2  0  2    6  0   4  0       0.0    13420.2359     0.00000  0.00000  0.00000

 21.   3  0  3   2  0  2    6  0   4  0    3044.0    13420.6642    -0.02165  0.31929 -0.12885
 22.   3  0  3   2  0  2    6  0   4  0    4034.5    13420.9868    -0.03800  0.56065 -0.22626
 23.   3  0  3   2  0  2    6  0   4  0    5057.7    13421.4149    -0.05965  0.88060 -0.35540

 24.   3  0  3   2  0  2    6  2   4  2    3044.0    13420.6087    -0.00594  0.26536 -0.10892
 25.   3  0  3   2  0  2    6  2   4  2    4034.5    13420.8804    -0.01021  0.46618 -0.19135
 26.   3  0  3   2  0  2    6  2   4  2    5057.7    13421.2482    -0.01560  0.73267 -0.30073

 27.   3  0  3   2  0  2    6  4   4  4    3044.0    13420.4336     0.04065  0.10297 -0.04893
 28.   3  0  3   2  0  2    6  4   4  4    4034.5    13420.5767     0.07142  0.18088 -0.08595
 29.   3  0  3   2  0  2    6  4   4  4    5057.7    13420.7670     0.11225  0.28425 -0.13506

!------------------------------------------------------------
!  3_13 <- 2_12 a.R
!------------------------------------------------------------
 30.   3  1  3   2  1  2    6  0   4  0       0.0    13152.3178     0.00000  0.00000  0.00000

 31.   3  1  3   2  1  2    6  0   4  0    2032.9    13152.3282    -0.00278  0.01438 -0.00001
```

```
 32.   3  1  3   2  1  2    6  0    4  0    3035.8    13152.3458    -0.00619  0.03207 -0.00003

 33.   3  1  3   2  1  2    6  2    4  2    2032.9    13152.4094     0.07669  0.01450  0.00271
 34.   3  1  3   2  1  2    6  2    4  2    3035.8    13152.5168     0.17095  0.03232  0.00604

 35.   3  1  3   2  1  2    6  4    4  4    2032.9    13152.6365     0.31477  0.01484  0.01086
 36.   3  1  3   2  1  2    6  4    4  4    3035.8    13153.0255     0.70073  0.03299  0.02416

 ------------------------------------------------------------------------------




////////////////////////////////////////////////////////////////////////////////////////////
//////////////// cut here - please display and print using a fixed width font //////////////
////////////////////////////////////////////////////////////////////////////////////////////


  ____________________________________________________________________________
 |                                                                            |
 |   QSTARK - Fully diagonalizing fit of Stark shifts in a rotor              |
 |            with zero or one quadrupolar nuclei                             |
 |____________________________________________________________________________|
  version 14.XI.2022                                           Zbigniew KISIEL


 ------------------------------------------------------------------------------
 AA conformer of valeronitrile
 ------------------------------------------------------------------------------

 Calculation for J and F exceeding the value in data by at least 2
 The fit will be made to TRANSITION FREQUENCIES

    77 Lines read in
    77 Lines fitted    --->    7 field free and   70 at non-zero field
     2 Constants fitted

 TRANSITIONS (F and MF in units of 1/2):


      J K  K  <- J K  K     F MF<- F MF    Volts        Obs          Obs-Calc       Calc         Field      Field**2
         -1 +1      -1 +1

!
! Rotational and centrifugal parameters are fixed at values from
! A-reduction refit of data from Ordu et al., A&A 541 (2012) A121:1-8
!
!---------------------------------------------------------------------
!  4_14 <- 3_13 (a.R)
!---------------------------------------------------------------------
  1.   4  1  4   3  1  3    8  0    6  0       0.0    10250.78160     -0.00003     10250.7816      0.0000        0.00
  2.   4  1  4   3  1  3    6  0    4  0       0.0    10250.84060      0.00002     10250.8406      0.0000        0.00
  3.   4  1  4   3  1  3   10  0    8  0       0.0    10250.89590      0.00003     10250.8959      0.0000        0.00

  4.   4  1  4   3  1  3    6  0    4  0    1014.0    10251.09260      0.00411     10251.0885     37.5867     1412.76
  5.   4  1  4   3  1  3    6  0    4  0    1525.0    10251.38160     -0.00037     10251.3820     56.5284     3195.46
  6.   4  1  4   3  1  3    6  0    4  0    1715.0    10251.51390     -0.00177     10251.5157     63.5713     4041.30

  7.   4  1  4   3  1  3    6  2    4  2    1014.0    10251.19740      0.00167     10251.1957     37.5867     1412.76
  8.   4  1  4   3  1  3    6  2    4  2    1515.4    10251.58520      0.00365     10251.5816     56.1725     3155.35

  9.   4  1  4   3  1  3    6  4    4  4    1316.9    10251.92790     -0.00307     10251.9310     48.8146     2382.86
 10.   4  1  4   3  1  3    6  4    4  4    1525.0    10252.23410      0.00285     10252.2312     56.5284     3195.46
 11.   4  1  4   3  1  3    6  4    4  4    1715.0    10252.52560     -0.00069     10252.5263     63.5713     4041.30

 12.   4  1  4   3  1  3    8  0    6  0    1014.0    10251.06940      0.00377     10251.0656     37.5867     1412.76

 13.   4  1  4   3  1  3    8  2    6  2    1525.0    10251.68010     -0.00312     10251.6832     56.5284     3195.46
 14.   4  1  4   3  1  3    8  2    6  2    1715.0    10251.95680     -0.00261     10251.9594     63.5713     4041.30

 15.   4  1  4   3  1  3    8  4    6  4    1009.1    10251.34120     -0.00277     10251.3440     37.4051     1399.14

 16.   4  1  4   3  1  3   10  4    8  4    1014.0    10251.24780      0.00316     10251.2446     37.5867     1412.76
 17.   4  1  4   3  1  3   10  4    8  4    1515.4    10251.61680      0.00227     10251.6145     56.1725     3155.35
 18.   4  1  4   3  1  3   10  4    8  4    1715.0    10251.79620      0.00578     10251.7904     63.5713     4041.30

 19.   4  1  4   3  1  3   10  6    8  6    1009.1    10251.40530     -0.00246     10251.4078     37.4051     1399.14
 20.   4  1  4   3  1  3   10  6    8  6    1315.0    10251.74540      0.00116     10251.7442     48.7441     2375.99
 21.   4  1  4   3  1  3   10  6    8  6    1525.0    10252.02070     -0.00112     10252.0218     56.5284     3195.46
 22.   4  1  4   3  1  3   10  6    8  6    1715.0    10252.30940      0.00278     10252.3066     63.5713     4041.30

 23.   4  1  4   3  1  3   10  8    8  8    1009.1    10251.68270      0.00034     10251.6824     37.4051     1399.14
 24.   4  1  4   3  1  3   10  8    8  8    1316.9    10252.23840     -0.00231     10252.2407     48.8146     2382.86
```

```
!-----------------------------------------------------------------------
!  4_04 <- 3_03 (a.R)
!-----------------------------------------------------------------------
 25.   4  0  4   3  0  3    6  0   4  0       0.0    10389.06600     -0.00001      10389.0660     0.0000          0.00
 26.   4  0  4   3  0  3    8  0   6  0       0.0    10389.12170      0.00004      10389.1217     0.0000          0.00
 27.   4  0  4   3  0  3   10  0   8  0       0.0    10389.14480      0.00003      10389.1448     0.0000          0.00
 28.   4  0  4   3  0  3    6  0   6  0       0.0    10390.29700     -0.00002      10390.2970     0.0000          0.00

 29.   4  0  4   3  0  3    6  0   4  0    3033.8    10388.79360     -0.00032      10388.7939   112.4563      12646.41
 30.   4  0  4   3  0  3    6  0   4  0    4043.3    10388.57440      0.00290      10388.5715   149.8762      22462.87
 31.   4  0  4   3  0  3    6  0   4  0    5050.5    10388.27720      0.00122      10388.2760   187.2109      35047.91
 32.   4  0  4   3  0  3    6  0   4  0    5565.9    10388.09760      0.00171      10388.0959   206.3156      42566.13
 33.   4  0  4   3  0  3    6  0   4  0    6055.8    10387.89870     -0.00810      10387.9068   224.4751      50389.08
 34.   4  0  4   3  0  3    6  0   4  0    6576.5    10387.69360      0.00659      10387.6870   243.7763      59426.89

 35.   4  0  4   3  0  3    6  2   4  2    3033.8    10388.91940      0.00364      10388.9158   112.4563      12646.41
 36.   4  0  4   3  0  3    6  2   4  2    4043.3    10388.76770      0.00098      10388.7667   149.8762      22462.87
 37.   4  0  4   3  0  3    6  2   4  2    5062.4    10388.55130      0.00136      10388.5499   187.6520      35213.26
 38.   4  0  4   3  0  3    6  2   4  2    5565.0    10388.42390      0.00469      10388.4192   206.2822      42552.37

 39.   4  0  4   3  0  3    6  4   4  4    3033.8    10389.30040      0.00245      10389.2979   112.4563      12646.41
 40.   4  0  4   3  0  3    6  4   4  4    4043.3    10389.37540      0.00229      10389.3731   149.8762      22462.87
 41.   4  0  4   3  0  3    6  4   4  4    5062.4    10389.40180      0.00504      10389.3968   187.6520      35213.26
! same Stark lobe but QSTARK labelling switch
 42.   4  0  4   3  0  3    6  4   6  4    5565.0    10389.39680      0.00012      10389.3967   206.2822      42552.37
 43.   4  0  4   3  0  3    6  4   6  4    6055.8    10389.39080     -0.00282      10389.3936   224.4751      50389.08
 44.   4  0  4   3  0  3    6  4   6  4    6567.3    10389.38430     -0.00531      10389.3896   243.4353      59260.74
 45.   4  0  4   3  0  3    6  4   6  4    7066.9    10389.38360     -0.00271      10389.3863   261.9544      68620.09

 46.   4  0  4   3  0  3    8  0   6  0    5062.4    10388.48530      0.00295      10388.4823   187.6520      35213.26

 47.   4  0  4   3  0  3    8  2   6  2    2019.1    10389.05500      0.00260      10389.0524    74.8436       5601.56
 48.   4  0  4   3  0  3    8  2   6  2    4043.3    10388.92890     -0.00074      10388.9296   149.8762      22462.87
 49.   4  0  4   3  0  3    8  2   6  2    5062.4    10388.89630     -0.00237      10388.8987   187.6520      35213.26
 50.   4  0  4   3  0  3    8  2   6  2    5565.0    10388.89580      0.00127      10388.8945   206.2822      42552.37
 51.   4  0  4   3  0  3    8  2   6  2    6055.8    10388.89780      0.00112      10388.8967   224.4751      50389.08
 52.   4  0  4   3  0  3    8  2   6  2    6567.3    10388.90700      0.00271      10388.9043   243.4353      59260.74
 53.   4  0  4   3  0  3    8  2   6  2    7069.8    10388.91700      0.00111      10388.9159   262.0619      68676.42

 54.   4  0  4   3  0  3    8  4   6  4    3033.8    10389.23300      0.00336      10389.2296   112.4563      12646.41
 55.   4  0  4   3  0  3    8  4   6  4    4038.8    10389.45660      0.00145      10389.4552   149.7094      22412.90
 56.   4  0  4   3  0  3    8  4   6  4    5062.4    10389.84590     -0.00288      10389.8488   187.6520      35213.26

 57.   4  0  4   3  0  3    8  6   6  6    2019.1    10389.25170     -0.00014      10389.2518    74.8436       5601.56
 58.   4  0  4   3  0  3    8  6   6  6    3033.8    10389.42970      0.00261      10389.4271   112.4563      12646.41
 59.   4  0  4   3  0  3    8  6   6  6    4038.8    10389.66930     -0.00168      10389.6710   149.7094      22412.90
 60.   4  0  4   3  0  3    8  6   6  6    5062.4    10389.96870     -0.00145      10389.9701   187.6520      35213.26
 61.   4  0  4   3  0  3    8  6   6  6    6055.8    10390.28730      0.00329      10390.2840   224.4751      50389.08

 62.   4  0  4   3  0  3   10  4   8  4    2019.1    10389.08090     -0.00433      10389.0852    74.8436       5601.56
 63.   4  0  4   3  0  3   10  4   8  4    4043.3    10388.83110     -0.00161      10388.8327   149.8762      22462.87
 64.   4  0  4   3  0  3   10  4   8  4    5062.4    10388.60630      0.00198      10388.6043   187.6520      35213.26
 65.   4  0  4   3  0  3   10  4   8  4    5565.0    10388.46910      0.00155      10388.4675   206.2822      42552.37

 66.   4  0  4   3  0  3   10  6   8  6    3033.8    10389.19460      0.00723      10389.1874   112.4563      12646.41
 67.   4  0  4   3  0  3   10  6   8  6    4043.3    10389.19800      0.00456      10389.1934   149.8762      22462.87
 68.   4  0  4   3  0  3   10  6   8  6    5062.4    10389.18920     -0.00300      10389.1922   187.6520      35213.26
 69.   4  0  4   3  0  3   10  6   8  6    5565.0    10389.19190      0.00138      10389.1905   206.2822      42552.37
 70.   4  0  4   3  0  3   10  6   8  6    6044.8    10389.18740     -0.00157      10389.1890   224.0674      50206.19
 71.   4  0  4   3  0  3   10  6   8  6    6567.3    10389.18540     -0.00238      10389.1878   243.4353      59260.74
 72.   4  0  4   3  0  3   10  6   8  6    7069.8    10389.18400     -0.00344      10389.1874   262.0619      68676.42

 73.   4  0  4   3  0  3   10  8   8  8    2019.1    10389.30670      0.00064      10389.3061    74.8436       5601.56
 74.   4  0  4   3  0  3   10  8   8  8    3033.8    10389.51780      0.00481      10389.5130   112.4563      12646.41
 75.   4  0  4   3  0  3   10  8   8  8    4043.3    10389.80600     -0.00109      10389.8071   149.8762      22462.87
 76.   4  0  4   3  0  3   10  8   8  8    5062.4    10390.19580     -0.00036      10390.1962   187.6520      35213.26
 77.   4  0  4   3  0  3   10  8   8  8    5565.0    10390.42960      0.00694      10390.4227   206.2822      42552.37

 Standard deviation =       0.003023


 ITERATION NO = 3 CONSTANTS, deviations and changes:

     Mu.a      =          3.904682673 +-      0.001377092           0.000000000
     Mu.b      =          1.666240438 +-      0.000944196           0.000000000


 FINAL RESULTS OF LEAST SQUARES FITTING PROCEDURE
 ===============================================

 FITTED CONSTANTS:
```

A /MHz	15028.68713	1:Xab /MHz	0.
B /MHz	1334.10731	1:XJ.a/kHz	0.
C /MHz	1263.856699	1:XK.a/kHz	0.
DJ /kHz	0.1458342	1:XJbc/kHz	0.
DJK /kHz	-7.54678	1:Ma /MHz	0.
DK /kHz	214.3805	1:Mb-c/MHz	0.
dJ /kHz	0.0220207	1:Tr /MHz	0.
dK /kHz	0.4816	1:Xd /kHz	0.
HJ / Hz	0.00009962	1:Xbc /MHz	0.
HJK / Hz	-0.006464	1:Xac /MHz	0.
HKJ / Hz	-0.05301		
HK / Hz	4.77		
hJ / Hz	0.00003144		
hJK / Hz	0.	Mu.a /D	3.9046(13)
hK / Hz	0.	Mu.b /D	1.66624(94)
LKKJ /mHz	-0.02051	Mu.c /D	0.
1:Xa /MHz	-2.72667	d /cm	26.9776
1:Xb-c/MHz	-1.37612	k /cm	0.

1:X.bb /MHz 0.675275000000
1:X.cc /MHz 2.051395000000

CORRELATION COEFFICIENTS:

	Mu.a	Mu.b
Mu.a	1.0000	
Mu.b	-0.2177	1.0000

WORST FITTED LINES:

	J	K -1	K +1	<- J	K -1	K +1	F	MF	<- F	MF	Volts	Obs	Obs-Calc	Calc	Field	Field**2
33.	4	0	4	3	0	3	6	0	4	0	6055.8	10387.89870	-0.00810	10387.9068	224.4751	50389.08
66.	4	0	4	3	0	3	10	6	8	6	3033.8	10389.19460	0.00723	10389.1874	112.4563	12646.41
77.	4	0	4	3	0	3	10	8	8	8	5565.0	10390.42960	0.00694	10390.4227	206.2822	42552.37
34.	4	0	4	3	0	3	6	0	4	0	6576.5	10387.69360	0.00659	10387.6870	243.7763	59426.89
18.	4	1	4	3	1	3	10	4	8	4	1715.0	10251.79620	0.00578	10251.7904	63.5713	4041.30

CONTRIBUTIONS (from gradients) OF INDIVIDUAL CONSTANTS TO FREQUENCY:

!
!---
! 4_14 <- 3_13 (a.R)
!---

													Mu.a	Mu.b
1.	4	1	4	3	1	3	8	0	6	0	0.0	10250.7816	0.00000	0.00000
2.	4	1	4	3	1	3	6	0	4	0	0.0	10250.8406	0.00000	0.00000
3.	4	1	4	3	1	3	10	0	8	0	0.0	10250.8959	0.00000	0.00000
4.	4	1	4	3	1	3	6	0	4	0	1014.0	10251.0926	0.01773	0.22282
5.	4	1	4	3	1	3	6	0	4	0	1525.0	10251.3816	0.00672	0.50387
6.	4	1	4	3	1	3	6	0	4	0	1715.0	10251.5139	-0.00473	0.63732
7.	4	1	4	3	1	3	6	2	4	2	1014.0	10251.1974	0.12087	0.21161
8.	4	1	4	3	1	3	6	2	4	2	1515.4	10251.5852	0.19104	0.47401
9.	4	1	4	3	1	3	6	4	4	4	1316.9	10251.9279	0.61603	0.30109
10.	4	1	4	3	1	3	6	4	4	4	1525.0	10252.2341	0.73411	0.40668
11.	4	1	4	3	1	3	6	4	4	4	1715.0	10252.5256	0.86488	0.51922
12.	4	1	4	3	1	3	8	0	6	0	1014.0	10251.0694	0.06467	0.21916
13.	4	1	4	3	1	3	8	2	6	2	1525.0	10251.6801	0.54723	0.47614
14.	4	1	4	3	1	3	8	2	6	2	1715.0	10251.9568	0.74231	0.59968
15.	4	1	4	3	1	3	8	4	6	4	1009.1	10251.3412	0.46668	0.18747
16.	4	1	4	3	1	3	10	4	8	4	1014.0	10251.2478	0.11739	0.20374
17.	4	1	4	3	1	3	10	4	8	4	1515.4	10251.6168	0.17679	0.45911
18.	4	1	4	3	1	3	10	4	8	4	1715.0	10251.7962	0.20133	0.59115
19.	4	1	4	3	1	3	10	6	8	6	1009.1	10251.4053	0.31126	0.17817
20.	4	1	4	3	1	3	10	6	8	6	1315.0	10251.7454	0.50293	0.30615
21.	4	1	4	3	1	3	10	6	8	6	1525.0	10252.0207	0.66273	0.41543
22.	4	1	4	3	1	3	10	6	8	6	1715.0	10252.3094	0.82920	0.52958
23.	4	1	4	3	1	3	10	8	8	8	1009.1	10251.6827	0.64759	0.14399
24.	4	1	4	3	1	3	10	8	8	8	1316.9	10252.2384	1.10741	0.24884

```
!----------------------------------------------------------------------
!  4_04 <- 3_03 (a.R)
!----------------------------------------------------------------------
 25.   4  0  4   3  0  3    6  0   4  0       0.0    10389.0660     0.00000  0.00000
 26.   4  0  4   3  0  3    8  0   6  0       0.0    10389.1217     0.00000  0.00000
 27.   4  0  4   3  0  3   10  0   8  0       0.0    10389.1448     0.00000  0.00000
 28.   4  0  4   3  0  3    6  0   6  0       0.0    10390.2970     0.00000  0.00000

 29.   4  0  4   3  0  3    6  0   4  0    3033.8    10388.7936    -0.15012 -0.13056
 30.   4  0  4   3  0  3    6  0   4  0    4043.3    10388.5744    -0.28464 -0.23376
 31.   4  0  4   3  0  3    6  0   4  0    5050.5    10388.2772    -0.46726 -0.36738
 32.   4  0  4   3  0  3    6  0   4  0    5565.9    10388.0976    -0.57730 -0.44752
 33.   4  0  4   3  0  3    6  0   4  0    6055.8    10387.8987    -0.69106 -0.53100
 34.   4  0  4   3  0  3    6  0   4  0    6576.5    10387.6936    -0.82105 -0.62745

 35.   4  0  4   3  0  3    6  2   4  2    3033.8    10388.9194    -0.06207 -0.11475
 36.   4  0  4   3  0  3    6  2   4  2    4043.3    10388.7677    -0.15493 -0.20871
 37.   4  0  4   3  0  3    6  2   4  2    5062.4    10388.5513    -0.28684 -0.33310
 38.   4  0  4   3  0  3    6  2   4  2    5565.0    10388.4239    -0.36012 -0.40506

 39.   4  0  4   3  0  3    6  4   4  4    3033.8    10389.3004     0.21623 -0.06578
 40.   4  0  4   3  0  3    6  4   4  4    4043.3    10389.3754     0.22763 -0.13320
 41.   4  0  4   3  0  3    6  4   4  4    5062.4    10389.4018     0.23839 -0.22584
! same Stark lobe but QSTARK labelling switch
 42.   4  0  4   3  0  3    6  4   6  4    5565.0    10389.3968     0.26762 -0.27916
 43.   4  0  4   3  0  3    6  4   6  4    6055.8    10389.3908     0.31285 -0.33591
 44.   4  0  4   3  0  3    6  4   6  4    6567.3    10389.3843     0.37514 -0.39995
 45.   4  0  4   3  0  3    6  4   6  4    7066.9    10389.3836     0.44810 -0.46727

 46.   4  0  4   3  0  3    8  0   6  0    5062.4    10388.4853    -0.29431 -0.34444

 47.   4  0  4   3  0  3    8  2   6  2    2019.1    10389.0550    -0.01216 -0.04949
 48.   4  0  4   3  0  3    8  2   6  2    4043.3    10388.9289     0.09258 -0.18394
 49.   4  0  4   3  0  3    8  2   6  2    5062.4    10388.8963     0.23837 -0.27706
 50.   4  0  4   3  0  3    8  2   6  2    5565.0    10388.8958     0.32507 -0.32926
 51.   4  0  4   3  0  3    8  2   6  2    6055.8    10388.8978     0.41444 -0.38441
 52.   4  0  4   3  0  3    8  2   6  2    6567.3    10388.9070     0.51011 -0.44637
 53.   4  0  4   3  0  3    8  2   6  2    7069.8    10388.9170     0.60521 -0.51178

 54.   4  0  4   3  0  3    8  4   6  4    3033.8    10389.2330     0.28849 -0.06655
 55.   4  0  4   3  0  3    8  4   6  4    4038.8    10389.4566     0.71391 -0.09552
 56.   4  0  4   3  0  3    8  4   6  4    5062.4    10389.8459     1.28077 -0.12504

 57.   4  0  4   3  0  3    8  6   6  6    2019.1    10389.2517     0.15207 -0.01617
 58.   4  0  4   3  0  3    8  6   6  6    3033.8    10389.4297     0.35013 -0.03144
 59.   4  0  4   3  0  3    8  6   6  6    4038.8    10389.6693     0.59449 -0.04583
 60.   4  0  4   3  0  3    8  6   6  6    5062.4    10389.9687     0.83496 -0.05517
 61.   4  0  4   3  0  3    8  6   6  6    6055.8    10390.2873     1.03076 -0.05717

 62.   4  0  4   3  0  3   10  4   8  4    2019.1    10389.0809    -0.01949 -0.04759
 63.   4  0  4   3  0  3   10  4   8  4    4043.3    10388.8311    -0.18183 -0.20300
 64.   4  0  4   3  0  3   10  4   8  4    5062.4    10388.6063    -0.32444 -0.32577
 65.   4  0  4   3  0  3   10  4   8  4    5565.0    10388.4691    -0.40156 -0.39715

 66.   4  0  4   3  0  3   10  6   8  6    3033.8    10389.1946     0.09550 -0.07892
 67.   4  0  4   3  0  3   10  6   8  6    4043.3    10389.1980     0.14852 -0.14530
 68.   4  0  4   3  0  3   10  6   8  6    5062.4    10389.1892     0.22595 -0.23374
 69.   4  0  4   3  0  3   10  6   8  6    5565.0    10389.1919     0.27559 -0.28518
 70.   4  0  4   3  0  3   10  6   8  6    6044.8    10389.1874     0.33030 -0.33902
 71.   4  0  4   3  0  3   10  6   8  6    6567.3    10389.1854     0.39778 -0.40285
 72.   4  0  4   3  0  3   10  6   8  6    7069.8    10389.1840     0.47008 -0.46928

 73.   4  0  4   3  0  3   10  8   8  8    2019.1    10389.3067     0.17443 -0.01158
 74.   4  0  4   3  0  3   10  8   8  8    3033.8    10389.5178     0.40190 -0.02696
 75.   4  0  4   3  0  3   10  8   8  8    4043.3    10389.8060     0.72865 -0.04941
 76.   4  0  4   3  0  3   10  8   8  8    5062.4    10390.1958     1.16247 -0.07956
 77.   4  0  4   3  0  3   10  8   8  8    5565.0    10390.4296     1.41475 -0.09719

 -----------------------------------------------------------------------------


/////////////////////////////////////////////////////////////////////////////////////////////
/////////////// cut here - please display and print using a fixed width font //////////////
/////////////////////////////////////////////////////////////////////////////////////////////

  ____________________________________________________________________________
 |                                                                            |
 |   QSTARK - Fully diagonalizing fit of Stark shifts in a rotor              |
 |            with zero or one quadrupolar nuclei                             |
 |____________________________________________________________________________|
  version 14.XI.2022                                            Zbigniew KISIEL
```

```
---------------------------------------------------------------------------
GA conformer of valeronitrile
---------------------------------------------------------------------------

Calculation for J and F exceeding the value in data by at least 2
The fit will be made to TRANSITION FREQUENCIES

   99 Lines read in
   99 Lines fitted    --->   14 field free and   85 at non-zero field
    3 Constants fitted

TRANSITIONS (F and MF in units of 1/2):


     J K  K  <- J K  K     F MF<- F MF    Volts        Obs          Obs-Calc       Calc          Field      Field**2
        -1 +1      -1 +1

!
! Rotational and centrifugal parameters are fixed at values from
! A-reduction refit of data from Ordu et al., A&A 541 (2012) A121:1-8
!
!-------------------------------------------------------------
! 110 <- 000 cR
!-------------------------------------------------------------
  1.   1  1  0   0  0  0    0  0   2  0       0.0     9423.23330      0.00015      9423.2331      0.0000        0.00
  2.   1  1  0   0  0  0    4  0   2  0       0.0     9424.12270      0.00017      9424.1225      0.0000        0.00
  3.   1  1  0   0  0  0    2  0   2  0       0.0     9424.71560      0.00016      9424.7154      0.0000        0.00

  4.   1  1  0   0  0  0    0  0   2  0     515.6     9423.64480      0.00094      9423.6439     19.1122      365.27
  5.   1  1  0   0  0  0    0  0   2  0     619.9     9423.74270      0.00018      9423.7425     22.9783      528.00
  6.   1  1  0   0  0  0    0  0   2  0     718.0     9423.82150     -0.00003      9423.8215     26.6147      708.34
  7.   1  1  0   0  0  0    0  0   2  0     807.7     9423.88740      0.00158      9423.8858     29.9397      896.38
  8.   1  1  0   0  0  0    0  0   2  0     911.6     9423.95510     -0.00031      9423.9554     33.7910     1141.83

  9.   1  1  0   0  0  0    2  2   2  2     412.9     9425.07960     -0.00306      9425.0827     15.3053      234.25
 10.   1  1  0   0  0  0    2  2   2  2     500.8     9425.29360     -0.00092      9425.2945     18.5635      344.61

 11.   1  1  0   0  0  0    4  0   2  0     403.6     9424.37780      0.00251      9424.3753     14.9606      223.82
 12.   1  1  0   0  0  0    4  0   2  0     515.6     9424.59760      0.00279      9424.5948     19.1122      365.27
 13.   1  1  0   0  0  0    4  0   2  0     617.7     9424.88840      0.00754      9424.8809     22.8968      524.26

 14.   1  1  0   0  0  0    4  2   2  2     403.6     9424.31620      0.00115      9424.3151     14.9606      223.82
 15.   1  1  0   0  0  0    4  2   2  2     515.6     9424.38410      0.00051      9424.3836     19.1122      365.27
 16.   1  1  0   0  0  0    4  2   2  2     619.9     9424.44110      0.00002      9424.4411     22.9783      528.00
 17.   1  1  0   0  0  0    4  2   2  2     709.3     9424.48750     -0.00026      9424.4878     26.2922      691.28
 18.   1  1  0   0  0  0    4  2   2  2     807.7     9424.53880      0.00008      9424.5387     29.9397      896.38
 19.   1  1  0   0  0  0    4  2   2  2     915.4     9424.59420     -0.00185      9424.5961     33.9319     1151.37

!-------------------------------------------------------------
! 312 <- 211 aR
!-------------------------------------------------------------
 20.   3  1  2   2  1  1    8  0   6  0       0.0    10375.72220      0.00065     10375.7216      0.0000        0.00
 21.   3  1  2   2  1  1    6  0   4  0       0.0    10375.73360      0.00064     10375.7330      0.0000        0.00
 22.   3  1  2   2  1  1    4  0   2  0       0.0    10375.84600      0.00060     10375.8454      0.0000        0.00

 23.   3  1  2   2  1  1    4  0   2  0    1621.7    10375.73370     -0.00187     10375.7356     60.1128     3613.55
 24.   3  1  2   2  1  1    4  0   2  0    2730.2    10375.68100     -0.00216     10375.6832    101.2025    10241.94

 25.   3  1  2   2  1  1    4  2   2  2    1621.7    10375.36600     -0.00220     10375.3682     60.1128     3613.55
 26.   3  1  2   2  1  1    4  2   2  2    2219.5    10375.12400     -0.00027     10375.1243     82.2720     6768.68
! same Stark lobe but label switch
 27.   3  1  2   2  1  1    4  2   4  2    2730.5    10374.87380      0.00169     10374.8721    101.2136    10244.19

 28.   3  1  2   2  1  1    6  0   4  0    1621.7    10375.45850     -0.00065     10375.4591     60.1128     3613.55
 29.   3  1  2   2  1  1    6  0   4  0    2219.5    10375.22000     -0.00032     10375.2203     82.2720     6768.68
 30.   3  1  2   2  1  1    6  0   4  0    2730.5    10374.95170     -0.00581     10374.9575    101.2136    10244.19

 31.   3  1  2   2  1  1    6  2   4  2    1621.7    10375.00840      0.00215     10375.0062     60.1128     3613.55
 32.   3  1  2   2  1  1    6  2   4  2    2219.5    10374.11350     -0.00359     10374.1171     82.2720     6768.68

 33.   3  1  2   2  1  1    6  4   4  4    1621.7    10374.89570      0.00302     10374.8927     60.1128     3613.55
 34.   3  1  2   2  1  1    6  4   4  4    2219.5    10374.16840      0.00769     10374.1607     82.2720     6768.68

 35.   3  1  2   2  1  1    8  0   6  0    2219.5    10375.31300     -0.00141     10375.3144     82.2720     6768.68
 36.   3  1  2   2  1  1    8  0   6  0    2730.5    10375.05140     -0.00066     10375.0521    101.2136    10244.19

 37.   3  1  2   2  1  1    8  2   6  2    1621.7    10375.59540     -0.00152     10375.5969     60.1128     3613.55
 38.   3  1  2   2  1  1    8  2   6  2    2730.2    10375.57260      0.00444     10375.5682    101.2025    10241.94

 39.   3  1  2   2  1  1    8  4   6  4    1621.7    10375.39260      0.00297     10375.3896     60.1128     3613.55
 40.   3  1  2   2  1  1    8  4   6  4    2219.5    10375.16310     -0.00337     10375.1665     82.2720     6768.68
```

```
 41.   3  1  2   2  1  1   8  4   6  4   2730.5   10374.92130     0.00031     10374.9210   101.2136   10244.19

 42.   3  1  2   2  1  1   8  6   6  6   1621.7   10374.74450     0.00191     10374.7426    60.1128    3613.55
 43.   3  1  2   2  1  1   8  6   6  6   2219.5   10373.87230     0.00346     10373.8688    82.2720    6768.68

!-------------------------------------------------------------
! 212 <- 101 bR
!-------------------------------------------------------------
 44.   2  1  2   1  0  1   4  0   4  0      0.0   12297.78200    -0.00003     12297.7820     0.0000       0.00
 45.   2  1  2   1  0  1   4  0   2  0      0.0   12297.79480     0.00034     12297.7945     0.0000       0.00
 46.   2  1  2   1  0  1   6  0   4  0      0.0   12298.41770     0.00039     12298.4173     0.0000       0.00
 47.   2  1  2   1  0  1   2  0   0  0      0.0   12298.75200     0.00034     12298.7517     0.0000       0.00
 48.   2  1  2   1  0  1   2  0   2  0      0.0   12298.78270    -0.00004     12298.7827     0.0000       0.00

 49.   2  1  2   1  0  1   2  0   0  0   1006.5   12298.91290    -0.00507     12298.9180    37.3087    1391.94
 50.   2  1  2   1  0  1   2  0   0  0   1621.8   12299.20630    -0.00593     12299.2122    60.1165    3614.00

 51.   2  1  2   1  0  1   2  2   2  2   1006.5   12298.71670    -0.00064     12298.7173    37.3087    1391.94
 52.   2  1  2   1  0  1   2  2   2  2   1621.8   12298.76590    -0.00250     12298.7684    60.1165    3614.00
 53.   2  1  2   1  0  1   2  2   2  2   2121.5   12298.91610    -0.00028     12298.9164    78.6393    6184.14
 54.   2  1  2   1  0  1   2  2   2  2   2533.4   12299.09110    -0.00244     12299.0935    93.9075    8818.63
! same Stark lobe but label switch
 55.   2  1  2   1  0  1   4  2   2  2   2932.6   12299.29950    -0.00497     12299.3045   108.7050   11816.78
 56.   2  1  2   1  0  1   4  2   2  2   3331.2   12299.54560    -0.00484     12299.5504   123.4802   15247.36

 57.   2  1  2   1  0  1   4  0   2  0   1621.8   12298.06100     0.00296     12298.0580    60.1165    3614.00
 58.   2  1  2   1  0  1   4  0   2  0   2121.5   12298.24840     0.00275     12298.2457    78.6393    6184.14
 59.   2  1  2   1  0  1   4  0   2  0   2533.4   12298.43750    -0.00061     12298.4381    93.9075    8818.63
 60.   2  1  2   1  0  1   4  0   2  0   2932.6   12298.65580    -0.00151     12298.6573   108.7050   11816.78
 61.   2  1  2   1  0  1   4  0   2  0   3331.2   12298.90490    -0.00346     12298.9084   123.4802   15247.36

 62.   2  1  2   1  0  1   4  2   2  2   1006.5   12297.77760     0.00508     12297.7725    37.3087    1391.94

 63.   2  1  2   1  0  1   4  2   4  2   1006.5   12297.43390    -0.00174     12297.4356    37.3087    1391.94

 64.   2  1  2   1  0  1   4  4   4  4   1006.5   12297.60510    -0.00158     12297.6067    37.3087    1391.94
 65.   2  1  2   1  0  1   4  4   4  4   1621.8   12297.25320     0.00426     12297.2489    60.1165    3614.00
 66.   2  1  2   1  0  1   4  4   4  4   2121.5   12296.79940     0.00003     12296.7994    78.6393    6184.14

 67.   2  1  2   1  0  1   6  0   4  0   2121.5   12297.52550     0.00406     12297.5214    78.6393    6184.14

 68.   2  1  2   1  0  1   6  2   4  2   2533.4   12297.47170    -0.00003     12297.4717    93.9075    8818.63
 69.   2  1  2   1  0  1   6  2   4  2   2932.6   12297.21090    -0.00061     12297.2115   108.7050   11816.78
 70.   2  1  2   1  0  1   6  2   4  2   3335.6   12296.91310    -0.00387     12296.9170   123.6433   15287.67

 71.   2  1  2   1  0  1   6  4   4  4   1006.5   12298.43990     0.00497     12298.4349    37.3087    1391.94
 72.   2  1  2   1  0  1   6  4   4  4   1621.8   12298.54210    -0.00027     12298.5424    60.1165    3614.00
 73.   2  1  2   1  0  1   6  4   4  4   2121.5   12298.71180     0.00708     12298.7047    78.6393    6184.14
 74.   2  1  2   1  0  1   6  4   4  4   2533.4   12298.88220    -0.00223     12298.8844    93.9075    8818.63
 75.   2  1  2   1  0  1   6  4   4  4   2932.6   12299.09100    -0.00437     12299.0954   108.7050   11816.78
 76.   2  1  2   1  0  1   6  4   4  4   3331.2   12299.33860    -0.00206     12299.3407   123.4802   15247.36

!-------------------------------------------------------------
! 313 <- 202 bR
!-------------------------------------------------------------
 77.   3  1  3   2  0  2   6  0   4  0      0.0   15291.71500     0.00056     15291.7144     0.0000       0.00
 78.   3  1  3   2  0  2   8  0   6  0      0.0   15292.34710     0.00058     15292.3465     0.0000       0.00
 79.   3  1  3   2  0  2   4  0   2  0      0.0   15292.56050     0.00052     15292.5600     0.0000       0.00

 80.   3  1  3   2  0  2   4  0   2  0   1510.1   15292.21330    -0.00416     15292.2175    55.9761    3133.32
 81.   3  1  3   2  0  2   4  0   2  0   2524.2   15291.59180     0.00224     15291.5896    93.5665    8754.69

 82.   3  1  3   2  0  2   6  0   4  0   1510.1   15291.34250    -0.00132     15291.3438    55.9761    3133.32
 83.   3  1  3   2  0  2   6  0   4  0   2028.4   15291.04050    -0.00341     15291.0439    75.1883    5653.28
 84.   3  1  3   2  0  2   6  0   4  0   3035.4   15290.19590    -0.00549     15290.2014   112.5156   12659.75

 85.   3  1  3   2  0  2   6  2   4  2   1510.1   15291.02300    -0.00317     15291.0262    55.9761    3133.32
 86.   3  1  3   2  0  2   6  2   4  2   2028.4   15290.47050    -0.00904     15290.4795    75.1883    5653.28
 87.   3  1  3   2  0  2   6  2   4  2   2524.2   15289.78480    -0.00957     15289.7944    93.5665    8754.69

 88.   3  1  3   2  0  2   6  4   4  4   2524.2   15292.30170     0.00252     15292.2992    93.5665    8754.69
 89.   3  1  3   2  0  2   6  4   4  4   3036.6   15292.46670     0.00001     15292.4667   112.5600   12669.76

 90.   3  1  3   2  0  2   8  0   6  0   1510.1   15291.73500     0.00277     15291.7322    55.9761    3133.32
 91.   3  1  3   2  0  2   8  0   6  0   3035.4   15289.87780     0.00227     15289.8755   112.5156   12659.75

 92.   3  1  3   2  0  2   8  4   6  4   2028.4   15291.59610     0.00069     15291.5954    75.1883    5653.28
 93.   3  1  3   2  0  2   8  4   6  4   2524.6   15291.19760     0.00656     15291.1910    93.5813    8757.47
 94.   3  1  3   2  0  2   8  4   6  4   3035.4   15290.69640     0.00528     15290.6911   112.5156   12659.75

 95.   3  1  3   2  0  2   8  6   6  6   1510.1   15292.58870     0.00494     15292.5838    55.9761    3133.32
 96.   3  1  3   2  0  2   8  6   6  6   2028.4   15292.78980     0.00402     15292.7858    75.1883    5653.28
 97.   3  1  3   2  0  2   8  6   6  6   2524.2   15293.05000     0.00721     15293.0428    93.5665    8754.69
```

```
 98.   3  1  3    2  0  2    8  6    6  6    2524.2    15293.05000      0.00721     15293.0428     93.5665     8754.69
 99.   3  1  3    2  0  2    8  6    6  6    3036.6    15293.36860     -0.00657     15293.3752    112.5600    12669.76

 Standard deviation =       0.003466


 ITERATION NO = 3 CONSTANTS, deviations and changes:

     Mu.a     =         2.685545733 +-      0.001276761           0.000000000
     Mu.b     =         2.852087694 +-      0.003165046           0.000000000
     Mu.c     =         0.389738395 +-      0.015466209          -0.000000001


 FINAL RESULTS OF LEAST SQUARES FITTING PROCEDURE
 ================================================

 FITTED CONSTANTS:

 A     /MHz        7635.62481           1:Xab /MHz           0.
 B     /MHz        1788.636927          1:XJ.a/kHz           0.
 C     /MHz        1554.218399          1:XK.a/kHz           0.
 DJ    /kHz           0.878123          1:XJbc/kHz           0.
 DJK   /kHz         -10.00176           1:Ma  /MHz           0.
 DK    /kHz          52.4857            1:Mb-c/MHz           0.
 dJ    /kHz           0.2333427         1:Tr  /MHz           0.
 dK    /kHz           1.72956           1:Xd  /kHz           0.
 HJ    / Hz           0.00058741        1:Xbc /MHz           0.
 HJK   / Hz           0.01675           1:Xac /MHz           0.
 HKJ   / Hz          -0.36068
 HK    / Hz           1.4593
 hJ    / Hz           0.0002657
 hJK   / Hz          -0.0047            Mu.a  /D             2.6855(12)
 hK    / Hz           0.1004            Mu.b  /D             2.8520(31)
 LKKJ  /mHz           0.01863           Mu.c  /D             0.389(15)
 1:Xa  /MHz          -0.0414             d    /cm           26.9776
 1:Xb-c/MHz          -3.91148            k    /cm            0.

 1:X.bb /MHz         -1.935040000000
 1:X.cc /MHz          1.976440000000

 CORRELATION COEFFICIENTS:

         Mu.a     Mu.b     Mu.c

 Mu.a     1.0000
 Mu.b    -0.6375   1.0000
 Mu.c    -0.6625   0.9596   1.0000


 WORST FITTED LINES:

      J K  K  <- J K  K      F MF<- F MF    Volts        Obs          Obs-Calc       Calc          Field      Field**2
         -1 +1      -1 +1

 87.   3  1  3    2  0  2    6  2    4  2    2524.2    15289.78480     -0.00957     15289.7944     93.5665     8754.69
 86.   3  1  3    2  0  2    6  2    4  2    2028.4    15290.47050     -0.00904     15290.4795     75.1883     5653.28
 34.   3  1  2    2  1  1    6  4    4  4    2219.5    10374.16840      0.00769     10374.1607     82.2720     6768.68
 13.   1  1  0    0  0  0    4  0    2  0     617.7     9424.88840      0.00754      9424.8809     22.8968      524.26
 97.   3  1  3    2  0  2    8  6    6  6    2524.2    15293.05000      0.00721     15293.0428     93.5665     8754.69


 CONTRIBUTIONS (from gradients) OF INDIVIDUAL CONSTANTS TO FREQUENCY:

                                                                         Mu.a     Mu.b     Mu.c
!
!-------------------------------------------------------------
! 110 <- 000 cR
!-------------------------------------------------------------
  1.   1  1  0    0  0  0    0  0    2  0       0.0     9423.2333     0.00000  0.00000  0.00000
  2.   1  1  0    0  0  0    4  0    2  0       0.0     9424.1227     0.00000  0.00000  0.00000
  3.   1  1  0    0  0  0    2  0    2  0       0.0     9424.7156     0.00000  0.00000  0.00000

  4.   1  1  0    0  0  0    0  0    2  0     515.6     9423.6448     0.23524  0.02962  0.00062
  5.   1  1  0    0  0  0    0  0    2  0     619.9     9423.7427     0.23187  0.03792  0.00089
  6.   1  1  0    0  0  0    0  0    2  0     718.0     9423.8215     0.22399  0.04695  0.00118
  7.   1  1  0    0  0  0    0  0    2  0     807.7     9423.8874     0.22286  0.05668  0.00148
  8.   1  1  0    0  0  0    0  0    2  0     911.6     9423.9551     0.23153  0.06985  0.00188

  9.   1  1  0    0  0  0    2  2    2  2     412.9     9425.0796     0.40228  0.03043  0.00044
 10.   1  1  0    0  0  0    2  2    2  2     500.8     9425.2936     0.63661  0.04677  0.00065

 11.   1  1  0    0  0  0    4  0    2  0     403.6     9424.3778     0.28909  0.02474  0.00040
 12.   1  1  0    0  0  0    4  0    2  0     515.6     9424.5976     0.57210  0.04488  0.00067
```

```
 13.   1  1  0   0  0  0    4  0   2  0     617.7     9424.8884     0.92383  0.06899  0.00097

 14.   1  1  0   0  0  0    4  2   2  2     403.6     9424.3162     0.11409  0.01681  0.00038
 15.   1  1  0   0  0  0    4  2   2  2     515.6     9424.3841     0.12465  0.02465  0.00061
 16.   1  1  0   0  0  0    4  2   2  2     619.9     9424.4411     0.13203  0.03345  0.00088
 17.   1  1  0   0  0  0    4  2   2  2     709.3     9424.4875     0.14203  0.04240  0.00114
 18.   1  1  0   0  0  0    4  2   2  2     807.7     9424.5388     0.15852  0.05382  0.00148
 19.   1  1  0   0  0  0    4  2   2  2     915.4     9424.5942     0.18306  0.06820  0.00189

!-----------------------------------------------------------
! 312 <- 211 aR
!-----------------------------------------------------------
 20.   3  1  2   2  1  1    8  0   6  0       0.0    10375.7222     0.00000  0.00000  0.00000
 21.   3  1  2   2  1  1    6  0   4  0       0.0    10375.7336     0.00000  0.00000  0.00000
 22.   3  1  2   2  1  1    4  0   2  0       0.0    10375.8460     0.00000  0.00000  0.00000

 23.   3  1  2   2  1  1    4  0   2  0    1621.7    10375.7337    -0.05040 -0.00763 -0.00038
 24.   3  1  2   2  1  1    4  0   2  0    2730.2    10375.6810    -0.04780 -0.00297 -0.00097

 25.   3  1  2   2  1  1    4  2   2  2    1621.7    10375.3660    -0.26507 -0.04767 -0.00058
 26.   3  1  2   2  1  1    4  2   2  2    2219.5    10375.1240    -0.41925 -0.07668 -0.00101
! same Stark lobe but label switch
 27.   3  1  2   2  1  1    4  2   4  2    2730.5    10374.8738    -0.61761 -0.12737 -0.00155

 28.   3  1  2   2  1  1    6  0   4  0    1621.7    10375.4585    -0.21911 -0.05514 -0.00058
 29.   3  1  2   2  1  1    6  0   4  0    2219.5    10375.2200    -0.41004 -0.10315 -0.00109
 30.   3  1  2   2  1  1    6  0   4  0    2730.5    10374.9517    -0.61997 -0.15590 -0.00164

 31.   3  1  2   2  1  1    6  2   4  2    1621.7    10375.0084    -0.74622 -0.21704 -0.00125
 32.   3  1  2   2  1  1    6  2   4  2    2219.5    10374.1135    -1.53034 -0.43254 -0.00244

 33.   3  1  2   2  1  1    6  4   4  4    1621.7    10374.8957    -0.75233 -0.11492 -0.00095
 34.   3  1  2   2  1  1    6  4   4  4    2219.5    10374.1684    -1.38602 -0.12985 -0.00151

 35.   3  1  2   2  1  1    8  0   6  0    2219.5    10375.3130    -0.38983 -0.11342 -0.00111
 36.   3  1  2   2  1  1    8  0   6  0    2730.5    10375.0514    -0.61085 -0.17152 -0.00168

 37.   3  1  2   2  1  1    8  2   6  2    1621.7    10375.5954    -0.05484  0.00259 -0.00035
 38.   3  1  2   2  1  1    8  2   6  2    2730.2    10375.5726    -0.03729  0.02507 -0.00089

 39.   3  1  2   2  1  1    8  4   6  4    1621.7    10375.3926    -0.23314 -0.03078 -0.00052
 40.   3  1  2   2  1  1    8  4   6  4    2219.5    10375.1631    -0.40866 -0.06689 -0.00098
 41.   3  1  2   2  1  1    8  4   6  4    2730.5    10374.9213    -0.61272 -0.11881 -0.00153

 42.   3  1  2   2  1  1    8  6   6  6    1621.7    10374.7445    -0.81877 -0.17628 -0.00116
 43.   3  1  2   2  1  1    8  6   6  6    2219.5    10373.8723    -1.53854 -0.34743 -0.00219

!-----------------------------------------------------------
! 212 <- 101 bR
!-----------------------------------------------------------
 44.   2  1  2   1  0  1    4  0   4  0       0.0    12297.7820     0.00000  0.00000  0.00000
 45.   2  1  2   1  0  1    4  0   2  0       0.0    12297.7948     0.00000  0.00000  0.00000
 46.   2  1  2   1  0  1    6  0   4  0       0.0    12298.4177     0.00000  0.00000  0.00000
 47.   2  1  2   1  0  1    2  0   0  0       0.0    12298.7520     0.00000  0.00000  0.00000
 48.   2  1  2   1  0  1    2  0   2  0       0.0    12298.7827     0.00000  0.00000  0.00000

 49.   2  1  2   1  0  1    2  0   0  0    1006.5    12298.9129     0.04610  0.12093 -0.00296
 50.   2  1  2   1  0  1    2  0   0  0    1621.8    12299.2063     0.18353  0.31843 -0.00727

 51.   2  1  2   1  0  1    2  2   2  2    1006.5    12298.7167    -0.12935  0.10918 -0.00414
 52.   2  1  2   1  0  1    2  2   2  2    1621.8    12298.7659    -0.13773  0.29691 -0.00943
 53.   2  1  2   1  0  1    2  2   2  2    2121.5    12298.9161    -0.13247  0.51502 -0.01544
 54.   2  1  2   1  0  1    2  2   2  2    2533.4    12299.0911    -0.14842  0.73718 -0.02175
! same Stark lobe but label switch
 55.   2  1  2   1  0  1    4  2   2  2    2932.6    12299.2995    -0.17727  0.98933 -0.02900
 56.   2  1  2   1  0  1    4  2   2  2    3331.2    12299.5456    -0.21550  1.27753 -0.03733

 57.   2  1  2   1  0  1    4  0   2  0    1621.8    12298.0610    -0.04805  0.30293 -0.00883
 58.   2  1  2   1  0  1    4  0   2  0    2121.5    12298.2484    -0.08194  0.51842 -0.01511
 59.   2  1  2   1  0  1    4  0   2  0    2533.4    12298.4375    -0.11643  0.73936 -0.02154
 60.   2  1  2   1  0  1    4  0   2  0    2932.6    12298.6558    -0.15539  0.99085 -0.02886
 61.   2  1  2   1  0  1    4  0   2  0    3331.2    12298.9049    -0.19958  1.27869 -0.03723

 62.   2  1  2   1  0  1    4  2   2  2    1006.5    12297.7776    -0.18312  0.10554 -0.00451

 63.   2  1  2   1  0  1    4  2   4  2    1006.5    12297.4339    -0.41122 -0.00066 -0.00242

 64.   2  1  2   1  0  1    4  4   4  4    1006.5    12297.6051    -0.30570  0.09729 -0.00534
 65.   2  1  2   1  0  1    4  4   4  4    1621.8    12297.2532    -0.87785  0.24722 -0.01442
 66.   2  1  2   1  0  1    4  4   4  4    2121.5    12296.7994    -1.54504  0.42073 -0.02497

 67.   2  1  2   1  0  1    6  0   4  0    2121.5    12297.5255    -1.04541  0.04979 -0.00552
```

```
 68.   2  1  2   1  0  1    6  2   4  2    2533.4    12297.4717    -0.89343  0.11130 -0.00385
 69.   2  1  2   1  0  1    6  2   4  2    2932.6    12297.2109    -1.16849  0.15122 -0.00497
 70.   2  1  2   1  0  1    6  2   4  2    3335.6    12296.9131    -1.49101  0.19726 -0.00631

 71.   2  1  2   1  0  1    6  4   4  4    1006.5    12298.4399    -0.07116  0.11311 -0.00375
 72.   2  1  2   1  0  1    6  4   4  4    1621.8    12298.5421    -0.09810  0.29955 -0.00917
 73.   2  1  2   1  0  1    6  4   4  4    2121.5    12298.7118    -0.12001  0.51584 -0.01536
 74.   2  1  2   1  0  1    6  4   4  4    2533.4    12298.8822    -0.14614  0.73731 -0.02174
 75.   2  1  2   1  0  1    6  4   4  4    2932.6    12299.0910    -0.17895  0.98919 -0.02902
 76.   2  1  2   1  0  1    6  4   4  4    3331.2    12299.3386    -0.21855  1.27731 -0.03736

!------------------------------------------------------------
! 313 <- 202 bR
!------------------------------------------------------------
 77.   3  1  3   2  0  2    6  0   4  0       0.0    15291.7150     0.00000  0.00000  0.00000
 78.   3  1  3   2  0  2    8  0   6  0       0.0    15292.3471     0.00000  0.00000  0.00000
 79.   3  1  3   2  0  2    4  0   2  0       0.0    15292.5605     0.00000  0.00000  0.00000

 80.   3  1  3   2  0  2    4  0   2  0    1510.1    15292.2133    -0.03028 -0.31416  0.00679
 81.   3  1  3   2  0  2    4  0   2  0    2524.2    15291.5918    -0.07622 -0.87978  0.01945

 82.   3  1  3   2  0  2    6  0   4  0    1510.1    15291.3425    -0.04922 -0.31635  0.00631
 83.   3  1  3   2  0  2    6  0   4  0    2028.4    15291.0405    -0.09090 -0.57234  0.01142
 84.   3  1  3   2  0  2    6  0   4  0    3035.4    15290.1959    -0.21669 -1.29142  0.02576

 85.   3  1  3   2  0  2    6  2   4  2    1510.1    15291.0230    -0.12983 -0.53360  0.01004
 86.   3  1  3   2  0  2    6  2   4  2    2028.4    15290.4705    -0.25370 -0.96570  0.01760
 87.   3  1  3   2  0  2    6  2   4  2    2524.2    15289.7848    -0.42566 -1.49949  0.02636

 88.   3  1  3   2  0  2    6  4   4  4    2524.2    15292.3017    -0.40938  0.79649 -0.03902
 89.   3  1  3   2  0  2    6  4   4  4    3036.6    15292.4667    -0.71229  1.13302 -0.05955

 90.   3  1  3   2  0  2    8  0   6  0    1510.1    15291.7350    -0.07061 -0.52426  0.01156
 91.   3  1  3   2  0  2    8  0   6  0    3035.4    15289.8778    -0.31764 -2.11746  0.04560

 92.   3  1  3   2  0  2    8  4   6  4    2028.4    15291.5961    -0.15142 -0.58273  0.00984
 93.   3  1  3   2  0  2    8  4   6  4    2524.6    15291.1976    -0.21306 -0.90162  0.01596
 94.   3  1  3   2  0  2    8  4   6  4    3035.4    15290.6964    -0.28420 -1.30374  0.02398

 95.   3  1  3   2  0  2    8  6   6  6    1510.1    15292.5887    -0.06450  0.29871 -0.01186
 96.   3  1  3   2  0  2    8  6   6  6    2028.4    15292.7898    -0.10119  0.54143 -0.02100
 97.   3  1  3   2  0  2    8  6   6  6    2524.2    15293.0500    -0.13856  0.84142 -0.03206
 98.   3  1  3   2  0  2    8  6   6  6    2524.2    15293.0500    -0.13856  0.84142 -0.03206
 99.   3  1  3   2  0  2    8  6   6  6    3036.6    15293.3686    -0.18110  1.22081 -0.04589

 ------------------------------------------------------------------------------



///////////////////////////////////////////////////////////////////////////////
/////////////// cut here - please display and print using a fixed width font //////////////
///////////////////////////////////////////////////////////////////////////////


  ____________________________________________________________________________
 |                                                                            |
 |   QSTARK - Fully diagonalizing fit of Stark shifts in a rotor              |
 |            with zero or one quadrupolar nuclei                             |
 |____________________________________________________________________________|
  version 26.VIII.2022                                        Zbigniew KISIEL


 ------------------------------------------------------------------------------
 Stark electrode calibration
 methyl cyanide, ground state, 20.X.2022 Ne carrier
 ------------------------------------------------------------------------------

 Calculation for J and F exceeding the value in data by at least 1
 The fit will be made to TRANSITION FREQUENCIES

    28 Lines read in
    28 Lines fitted    --->    3 field free and   25 at non-zero field
     1 Constants fitted

 TRANSITIONS (F and MF in units of 1/2):


      J K  K  <- J K  K      F MF<-  F MF    Volts        Obs          Obs-Calc       Calc          Field      Field**2
         -1 +1      -1 +1
  1.   1  0  1   0  0  0    2  2   2  2       0.0    18396.72280     -0.00243     18396.7252      0.0000         0.00
  2.   1  0  1   0  0  0    2  0   2  0    3855.1    18397.74070      0.00508     18397.7356    142.9721     20441.02
  3.   1  0  1   0  0  0    2  2   2  2    3855.1    18398.11100     -0.00123     18398.1122    142.9721     20441.02
```

4.	1	0	1	0	0	0	2	0	2	0	5465.2	18398.75410	-0.00158	18398.7557	202.6851	41081.23
5.	1	0	1	0	0	0	2	2	2	2	5465.2	18399.23890	-0.00631	18399.2452	202.6851	41081.23
6.	1	0	1	0	0	0	2	0	2	0	6677.1	18399.74830	-0.00748	18399.7558	247.6302	61320.70
7.	1	0	1	0	0	0	2	2	2	2	6677.1	18400.28450	-0.00647	18400.2910	247.6302	61320.70
8.	1	0	1	0	0	0	2	0	2	0	7686.2	18400.74200	0.00131	18400.7407	285.0541	81255.87
9.	1	0	1	0	0	0	2	2	2	2	7686.2	18401.29420	-0.00582	18401.3000	285.0541	81255.87
10.	1	0	1	0	0	0	2	0	2	0	8487.6	18401.61790	-0.00345	18401.6213	314.7753	99083.46
11.	1	0	1	0	0	0	2	2	2	2	8487.6	18402.18900	-0.00541	18402.1944	314.7753	99083.46
!																
12.	1	0	1	0	0	0	4	0	2	0	0.0	18397.99550	-0.00101	18397.9965	0.0000	0.00
13.	1	0	1	0	0	0	4	0	2	0	3855.1	18399.65030	-0.00020	18399.6505	142.9721	20441.02
14.	1	0	1	0	0	0	4	2	2	2	3855.1	18399.93240	0.00249	18399.9299	142.9721	20441.02
15.	1	0	1	0	0	0	4	0	2	0	5465.2	18400.94270	-0.00436	18400.9471	202.6851	41081.23
16.	1	0	1	0	0	0	4	2	2	2	5465.2	18402.14970	0.00051	18402.1492	202.6851	41081.23
17.	1	0	1	0	0	0	4	0	2	0	6677.1	18402.05450	-0.00791	18402.0624	247.6302	61320.70
18.	1	0	1	0	0	0	4	2	2	2	6677.1	18404.38920	-0.00091	18404.3901	247.6302	61320.70
19.	1	0	1	0	0	0	4	0	2	0	7686.2	18403.10580	-0.00026	18403.1061	285.0541	81255.87
20.	1	0	1	0	0	0	4	2	2	2	7686.2	18406.61250	-0.00535	18406.6179	285.0541	81255.87
21.	1	0	1	0	0	0	0	0	2	0	8487.6	18404.01710	-0.00183	18404.0189	314.7753	99083.46
22.	1	0	1	0	0	0	4	2	2	2	8487.6	18408.60600	-0.01167	18408.6177	314.7753	99083.46
!																
23.	1	0	1	0	0	0	0	0	2	0	0.0	18399.89170	-0.00069	18399.8924	0.0000	0.00
24.	1	0	1	0	0	0	0	0	2	0	3855.1	18401.55770	-0.00055	18401.5583	142.9721	20441.02
25.	1	0	1	0	0	0	0	0	2	0	5465.2	18403.61480	0.00141	18403.6134	202.6851	41081.23
26.	1	0	1	0	0	0	0	0	2	0	6677.1	18405.78340	-0.00078	18405.7842	247.6302	61320.70
27.	1	0	1	0	0	0	0	0	2	0	7686.2	18407.97260	-0.00419	18407.9768	285.0541	81255.87
28.	1	0	1	0	0	0	4	0	2	0	8487.6	18409.94610	-0.01155	18409.9576	314.7753	99083.46

Standard deviation = 0.004954

ITERATION NO = 1 CONSTANTS, deviations and changes:

d = 26.973498052 +- 0.002488090 0.009498052

TRANSITIONS (F and MF in units of 1/2):

	J	K -1	K +1	<- J	K -1	K +1	F	MF<-	F	MF	Volts	Obs	Obs-Calc	Calc	Field	Field**2
1.	1	0	1	0	0	0	2	2	2	2	0.0	18396.72280	-0.00243	18396.7252	0.0000	0.00
2.	1	0	1	0	0	0	2	0	2	0	3855.1	18397.74070	0.00579	18397.7349	142.9218	20426.63
3.	1	0	1	0	0	0	2	2	2	2	3855.1	18398.11100	-0.00039	18398.1114	142.9218	20426.63
4.	1	0	1	0	0	0	2	0	2	0	5465.2	18398.75410	-0.00015	18398.7543	202.6137	41052.31
5.	1	0	1	0	0	0	2	2	2	2	5465.2	18399.23890	-0.00479	18399.2437	202.6137	41052.31
6.	1	0	1	0	0	0	2	0	2	0	6677.1	18399.74830	-0.00535	18399.7536	247.5430	61277.53
7.	1	0	1	0	0	0	2	2	2	2	6677.1	18400.28450	-0.00427	18400.2888	247.5430	61277.53
8.	1	0	1	0	0	0	2	0	2	0	7686.2	18400.74200	0.00413	18400.7379	284.9538	81198.65
9.	1	0	1	0	0	0	2	2	2	2	7686.2	18401.29420	-0.00294	18401.2971	284.9538	81198.65
10.	1	0	1	0	0	0	2	0	2	0	8487.6	18401.61790	0.00000	18401.6179	314.6644	99013.69
11.	1	0	1	0	0	0	2	2	2	2	8487.6	18402.18900	-0.00192	18402.1909	314.6644	99013.69
!																
12.	1	0	1	0	0	0	4	0	2	0	0.0	18397.99550	-0.00101	18397.9965	0.0000	0.00
13.	1	0	1	0	0	0	4	0	2	0	3855.1	18399.65030	0.00081	18399.6495	142.9218	20426.63
14.	1	0	1	0	0	0	4	2	2	2	3855.1	18399.93240	0.00398	18399.9284	142.9218	20426.63
15.	1	0	1	0	0	0	4	0	2	0	5465.2	18400.94270	-0.00269	18400.9454	202.6137	41052.31
16.	1	0	1	0	0	0	4	2	2	2	5465.2	18402.14970	0.00369	18402.1460	202.6137	41052.31
17.	1	0	1	0	0	0	4	0	2	0	6677.1	18402.05450	-0.00561	18402.0601	247.5430	61277.53
18.	1	0	1	0	0	0	4	2	2	2	6677.1	18404.38920	0.00390	18404.3853	247.5430	61277.53
19.	1	0	1	0	0	0	4	0	2	0	7686.2	18403.10580	0.00270	18403.1031	284.9538	81198.65
20.	1	0	1	0	0	0	4	2	2	2	7686.2	18406.61250	0.00106	18406.6114	284.9538	81198.65
21.	1	0	1	0	0	0	0	0	2	0	8487.6	18404.01710	0.00172	18404.0154	314.6644	99013.69
22.	1	0	1	0	0	0	4	2	2	2	8487.6	18408.60600	-0.00384	18408.6098	314.6644	99013.69
!																
23.	1	0	1	0	0	0	0	0	2	0	0.0	18399.89170	-0.00069	18399.8924	0.0000	0.00
24.	1	0	1	0	0	0	0	0	2	0	3855.1	18401.55770	0.00078	18401.5569	142.9218	20426.63
25.	1	0	1	0	0	0	0	0	2	0	5465.2	18403.61480	0.00444	18403.6104	202.6137	41052.31
26.	1	0	1	0	0	0	0	0	2	0	6677.1	18405.78340	0.00393	18405.7795	247.5430	61277.53
27.	1	0	1	0	0	0	0	0	2	0	7686.2	18407.97260	0.00214	18407.9705	284.9538	81198.65
28.	1	0	1	0	0	0	4	0	2	0	8487.6	18409.94610	-0.00377	18409.9499	314.6644	99013.69

Standard deviation = 0.003361

ITERATION NO = 2 CONSTANTS, deviations and changes:

d = 26.973503266 +- 0.001689791 0.000005214

TRANSITIONS (F and MF in units of 1/2):

	J	K -1	K +1	<- J	K -1	K +1	F	MF<-	F	MF	Volts	Obs	Obs-Calc	Calc	Field	Field**2

```
  1.   1  0  1   0  0  0    2  2   2  2      0.0    18396.72280    -0.00243     18396.7252     0.0000        0.00
  2.   1  0  1   0  0  0    2  0   2  0   3855.1    18397.74070     0.00580     18397.7349   142.9217    20426.62
  3.   1  0  1   0  0  0    2  2   2  2   3855.1    18398.11100    -0.00039     18398.1114   142.9217    20426.62
  4.   1  0  1   0  0  0    2  0   2  0   5465.2    18398.75410    -0.00015     18398.7543   202.6137    41052.29
  5.   1  0  1   0  0  0    2  2   2  2   5465.2    18399.23890    -0.00479     18399.2437   202.6137    41052.29
  6.   1  0  1   0  0  0    2  0   2  0   6677.1    18399.74830    -0.00535     18399.7536   247.5429    61277.50
  7.   1  0  1   0  0  0    2  2   2  2   6677.1    18400.28450    -0.00426     18400.2888   247.5429    61277.50
  8.   1  0  1   0  0  0    2  0   2  0   7686.2    18400.74200     0.00413     18400.7379   284.9537    81198.62
  9.   1  0  1   0  0  0    2  2   2  2   7686.2    18401.29420    -0.00294     18401.2971   284.9537    81198.62
 10.   1  0  1   0  0  0    2  0   2  0   8487.6    18401.61790     0.00000     18401.6179   314.6644    99013.66
 11.   1  0  1   0  0  0    2  2   2  2   8487.6    18402.18900    -0.00192     18402.1909   314.6644    99013.66
!
 12.   1  0  1   0  0  0    4  0   2  0      0.0    18397.99550    -0.00101     18397.9965     0.0000        0.00
 13.   1  0  1   0  0  0    4  0   2  0   3855.1    18399.65030     0.00081     18399.6495   142.9217    20426.62
 14.   1  0  1   0  0  0    4  2   2  2   3855.1    18399.93240     0.00398     18399.9284   142.9217    20426.62
 15.   1  0  1   0  0  0    4  0   2  0   5465.2    18400.94270    -0.00269     18400.9454   202.6137    41052.29
 16.   1  0  1   0  0  0    4  2   2  2   5465.2    18402.14970     0.00369     18402.1460   202.6137    41052.29
 17.   1  0  1   0  0  0    4  0   2  0   6677.1    18402.05450    -0.00561     18402.0601   247.5429    61277.50
 18.   1  0  1   0  0  0    4  2   2  2   6677.1    18404.38920     0.00390     18404.3853   247.5429    61277.50
 19.   1  0  1   0  0  0    4  0   2  0   7686.2    18403.10580     0.00270     18403.1031   284.9537    81198.62
 20.   1  0  1   0  0  0    4  2   2  2   7686.2    18406.61250     0.00106     18406.6114   284.9537    81198.62
 21.   1  0  1   0  0  0    0  0   2  0   8487.6    18404.01710     0.00172     18404.0154   314.6644    99013.66
 22.   1  0  1   0  0  0    4  2   2  2   8487.6    18408.60600    -0.00383     18408.6098   314.6644    99013.66
!
 23.   1  0  1   0  0  0    0  0   2  0      0.0    18399.89170    -0.00069     18399.8924     0.0000        0.00
 24.   1  0  1   0  0  0    0  0   2  0   3855.1    18401.55770     0.00078     18401.5569   142.9217    20426.62
 25.   1  0  1   0  0  0    0  0   2  0   5465.2    18403.61480     0.00445     18403.6104   202.6137    41052.29
 26.   1  0  1   0  0  0    0  0   2  0   6677.1    18405.78340     0.00393     18405.7795   247.5429    61277.50
 27.   1  0  1   0  0  0    0  0   2  0   7686.2    18407.97260     0.00214     18407.9705   284.9537    81198.62
 28.   1  0  1   0  0  0    4  0   2  0   8487.6    18409.94610    -0.00377     18409.9499   314.6644    99013.66

 Standard deviation =       0.003361


 ITERATION NO = 3 CONSTANTS, deviations and changes:

       d      =        26.973503266 +-      0.001689792           0.000000000


 FINAL RESULTS OF LEAST SQUARES FITTING PROCEDURE
 ================================================

 FITTED CONSTANTS:

 A     /MHz      148900.1236           1:Xab /MHz           0.
 B     /MHz        9198.899299         1:XJ.a/kHz           0.
 C     /MHz        9198.899299         1:XK.a/kHz           0.
 DJ    /kHz           3.804801256      1:XJbc/kHz           0.
 DJK   /kHz         177.417            1:Ma  /MHz          -0.0007
 DK    /kHz        2830.04             1:Mb-c/MHz           0.
 dJ    /kHz           0.               1:Tr  /MHz          -0.0044
 dK    /kHz           0.               1:Xd  /kHz           0.
 HJ    / Hz          -0.014            1:Xbc /MHz           0.
 HJK   / Hz           0.               1:Xac /MHz           0.
 HKJ   / Hz           0.
 HK    / Hz           0.
 hJ    / Hz           0.
 hJK   / Hz           0.               Mu.a  /D             3.92197
 hK    / Hz           0.               Mu.b  /D             0.
 LKKJ  /mHz           0.               Mu.c  /D             0.
 1:Xa  /MHz          -4.22534           d    /cm           26.9735(16)
 1:Xb-c/MHz           0.                k    /cm            0.

 1:X.bb /MHz          2.112670000000
 1:X.cc /MHz          2.112670000000


 WORST FITTED LINES:

      J K  K  <- J K  K      F MF<- F MF    Volts        Obs          Obs-Calc       Calc          Field      Field**2
         -1 +1      -1 +1

  2.   1  0  1   0  0  0    2  0   2  0   3855.1    18397.74070     0.00580     18397.7349   142.9217    20426.62
 17.   1  0  1   0  0  0    4  0   2  0   6677.1    18402.05450    -0.00561     18402.0601   247.5429    61277.50
  6.   1  0  1   0  0  0    2  0   2  0   6677.1    18399.74830    -0.00535     18399.7536   247.5429    61277.50
  5.   1  0  1   0  0  0    2  2   2  2   5465.2    18399.23890    -0.00479     18399.2437   202.6137    41052.29
 25.   1  0  1   0  0  0    0  0   2  0   5465.2    18403.61480     0.00445     18403.6104   202.6137    41052.29

 ----------------------------------------------------------------------------


/////////////////////////////////////////////////////////////////////////////////////////////
//////////////// cut here - please display and print using a fixed width font //////////////
```

//

```
 _____________________________________________________________________________
|                                                                             |
|   QSTARK - Fully diagonalizing fit of Stark shifts in a rotor               |
|            with zero or one quadrupolar nuclei                              |
|_____________________________________________________________________________|
 version 26.VIII.2022                                        Zbigniew KISIEL
```

Stark electrode calibration
methyl iodide, ground state, 20.X.2022, Ne carrier

Calculation for J and F exceeding the value in data by at least 1
The fit will be made to TRANSITION FREQUENCIES

31 Lines read in
30 Lines fitted ---> 3 field free and 27 at non-zero field
1 Constants fitted

TRANSITIONS (F and MF in units of 1/2):

	J	K -1	K +1	<- J	K -1	K +1	F	MF<-	F	MF	Volts	Obs	Obs-Calc	Calc	Field	Field**2
1.	1	0	1	0	0	0	5	1	5	1	0.0	14694.92870	-0.00108	14694.9298	0.0000	0.00
2.	1	0	1	0	0	0	5	3	5	3	6269.7	14695.70170	0.00220	14695.6995	232.4694	54042.03
3.	1	0	1	0	0	0	5	5	5	5	6269.7	14696.07190	-0.00007	14696.0720	232.4694	54042.03
4.	1	0	1	0	0	0	5	3	5	3	8695.4	14696.41330	0.00347	14696.4098	322.4101	103948.26
5.	1	0	1	0	0	0	5	5	5	5	8695.4	14697.12490	-0.00124	14697.1261	322.4101	103948.26
6.	1	0	1	0	0	0	5	3	5	3	10920.8	14697.26250	-0.00092	14697.2634	404.9240	163963.44
7.	1	0	1	0	0	0	5	5	5	5	10920.8	14698.39000	-0.00306	14698.3931	404.9240	163963.44
8.	1	0	1	0	0	0	5	3	5	3	12324.2	0.00000	-14697.90086--	14697.9009	456.9596	208812.06
9.	1	0	1	0	0	0	5	5	5	5	12324.2	14699.33230	-0.00697	14699.3393	456.9596	208812.06
10.	1	0	1	0	0	0	7	1	5	1	0.0	15100.74080	-0.00561	15100.7464	0.0000	0.00
11.	1	0	1	0	0	0	7	1	5	1	6269.7	15101.72590	0.00472	15101.7212	232.4694	54042.03
12.	1	0	1	0	0	0	7	3	5	3	6269.7	15101.66660	0.00409	15101.6625	232.4694	54042.03
13.	1	0	1	0	0	0	7	5	5	5	6269.7	15101.54880	0.00404	15101.5448	232.4694	54042.03
14.	1	0	1	0	0	0	7	1	5	1	8695.4	15102.62450	0.00438	15102.6201	322.4101	103948.26
15.	1	0	1	0	0	0	7	3	5	3	8695.4	15102.51060	0.00255	15102.5081	322.4101	103948.26
16.	1	0	1	0	0	0	7	5	5	5	8695.4	15102.28560	0.00316	15102.2824	322.4101	103948.26
17.	1	0	1	0	0	0	7	1	5	1	10920.8	15103.69970	0.00015	15103.6996	404.9240	163963.44
18.	1	0	1	0	0	0	7	3	5	3	10920.8	15103.52500	0.00071	15103.5243	404.9240	163963.44
19.	1	0	1	0	0	0	7	5	5	5	10920.8	15103.17040	0.00032	15103.1701	404.9240	163963.44
20.	1	0	1	0	0	0	7	1	5	1	12324.2	15104.50190	-0.00317	15104.5051	456.9596	208812.06
21.	1	0	1	0	0	0	7	3	5	3	12324.2	15104.28100	-0.00229	15104.2833	456.9596	208812.06
22.	1	0	1	0	0	0	7	5	5	5	12324.2	15103.83220	-0.00158	15103.8338	456.9596	208812.06
23.	1	0	1	0	0	0	3	1	5	1	0.0	15275.89340	0.00020	15275.8932	0.0000	0.00
24.	1	0	1	0	0	0	3	1	5	1	6269.7	15276.73940	0.00326	15276.7361	232.4694	54042.03
25.	1	0	1	0	0	0	3	3	5	3	6269.7	15276.65350	0.00356	15276.6499	232.4694	54042.03
26.	1	0	1	0	0	0	3	1	5	1	8695.4	15277.51940	0.00369	15277.5157	322.4101	103948.26
27.	1	0	1	0	0	0	3	3	5	3	8695.4	15277.35310	0.00362	15277.3495	322.4101	103948.26
28.	1	0	1	0	0	0	3	1	5	1	10920.8	15278.45450	-0.00014	15278.4546	404.9240	163963.44
29.	1	0	1	0	0	0	3	3	5	3	10920.8	15278.19200	0.00037	15278.1916	404.9240	163963.44
30.	1	0	1	0	0	0	3	1	5	1	12324.2	15279.15630	-0.00103	15279.1573	456.9596	208812.06
31.	1	0	1	0	0	0	3	3	5	3	12324.2	15278.82040	-0.00120	15278.8216	456.9596	208812.06

Standard deviation = 0.003062

ITERATION NO = 1 CONSTANTS, deviations and changes:

d = 26.971915388 +- 0.003341456 0.001915388

TRANSITIONS (F and MF in units of 1/2):

	J	K -1	K +1	<- J	K -1	K +1	F	MF<-	F	MF	Volts	Obs	Obs-Calc	Calc	Field	Field**2
1.	1	0	1	0	0	0	5	1	5	1	0.0	14694.92870	-0.00108	14694.9298	0.0000	0.00
2.	1	0	1	0	0	0	5	3	5	3	6269.7	14695.70170	0.00231	14695.6994	232.4529	54034.35
3.	1	0	1	0	0	0	5	5	5	5	6269.7	14696.07190	0.00009	14696.0718	232.4529	54034.35
4.	1	0	1	0	0	0	5	3	5	3	8695.4	14696.41330	0.00368	14696.4096	322.3872	103933.50
5.	1	0	1	0	0	0	5	5	5	5	8695.4	14697.12490	-0.00092	14697.1258	322.3872	103933.50
6.	1	0	1	0	0	0	5	3	5	3	10920.8	14697.26250	-0.00059	14697.2631	404.8952	163940.15
7.	1	0	1	0	0	0	5	5	5	5	10920.8	14698.39000	-0.00257	14698.3926	404.8952	163940.15
8.	1	0	1	0	0	0	5	3	5	3	12324.2	0.00000	-14697.90044--	14697.9004	456.9271	208782.41
9.	1	0	1	0	0	0	5	5	5	5	12324.2	14699.33230	-0.00635	14699.3386	456.9271	208782.41

10.	1	0	1	0	0	0	7	1	5	1	0.0	15100.74080	-0.00561	15100.7464	0.0000	0.00
11.	1	0	1	0	0	0	7	1	5	1	6269.7	15101.72590	0.00485	15101.7210	232.4529	54034.35
12.	1	0	1	0	0	0	7	3	5	3	6269.7	15101.66660	0.00422	15101.6624	232.4529	54034.35
13.	1	0	1	0	0	0	7	5	5	5	6269.7	15101.54880	0.00415	15101.5446	232.4529	54034.35
14.	1	0	1	0	0	0	7	1	5	1	8695.4	15102.62450	0.00465	15102.6199	322.3872	103933.50
15.	1	0	1	0	0	0	7	3	5	3	8695.4	15102.51060	0.00280	15102.5078	322.3872	103933.50
16.	1	0	1	0	0	0	7	5	5	5	8695.4	15102.28560	0.00338	15102.2822	322.3872	103933.50
17.	1	0	1	0	0	0	7	1	5	1	10920.8	15103.69970	0.00056	15103.6991	404.8952	163940.15
18.	1	0	1	0	0	0	7	3	5	3	10920.8	15103.52500	0.00111	15103.5239	404.8952	163940.15
19.	1	0	1	0	0	0	7	5	5	5	10920.8	15103.17040	0.00067	15103.1697	404.8952	163940.15
20.	1	0	1	0	0	0	7	1	5	1	12324.2	15104.50190	-0.00264	15104.5045	456.9271	208782.41
21.	1	0	1	0	0	0	7	3	5	3	12324.2	15104.28100	-0.00179	15104.2828	456.9271	208782.41
22.	1	0	1	0	0	0	7	5	5	5	12324.2	15103.83220	-0.00114	15103.8333	456.9271	208782.41
23.	1	0	1	0	0	0	3	1	5	1	0.0	15275.89340	0.00020	15275.8932	0.0000	0.00
24.	1	0	1	0	0	0	3	1	5	1	6269.7	15276.73940	0.00338	15276.7360	232.4529	54034.35
25.	1	0	1	0	0	0	3	3	5	3	6269.7	15276.65350	0.00366	15276.6498	232.4529	54034.35
26.	1	0	1	0	0	0	3	1	5	1	8695.4	15277.51940	0.00392	15277.5155	322.3872	103933.50
27.	1	0	1	0	0	0	3	3	5	3	8695.4	15277.35310	0.00382	15277.3493	322.3872	103933.50
28.	1	0	1	0	0	0	3	1	5	1	10920.8	15278.45450	0.00022	15278.4543	404.8952	163940.15
29.	1	0	1	0	0	0	3	3	5	3	10920.8	15278.19200	0.00070	15278.1913	404.8952	163940.15
30.	1	0	1	0	0	0	3	1	5	1	12324.2	15279.15630	-0.00057	15279.1569	456.9271	208782.41
31.	1	0	1	0	0	0	3	3	5	3	12324.2	15278.82040	-0.00079	15278.8212	456.9271	208782.41

Standard deviation = 0.003045

ITERATION NO = 2 CONSTANTS, deviations and changes:

d = 26.971915593 +- 0.003323180 0.000000205

TRANSITIONS (F and MF in units of 1/2):

	J	K -1	K +1	<- J	K -1	K +1	F	MF<-	F	MF	Volts	Obs	Obs-Calc	Calc	Field	Field**2
1.	1	0	1	0	0	0	5	1	5	1	0.0	14694.92870	-0.00108	14694.9298	0.0000	0.00
2.	1	0	1	0	0	0	5	3	5	3	6269.7	14695.70170	0.00231	14695.6994	232.4529	54034.35
3.	1	0	1	0	0	0	5	5	5	5	6269.7	14696.07190	0.00009	14696.0718	232.4529	54034.35
4.	1	0	1	0	0	0	5	3	5	3	8695.4	14696.41330	0.00368	14696.4096	322.3872	103933.50
5.	1	0	1	0	0	0	5	5	5	5	8695.4	14697.12490	-0.00092	14697.1258	322.3872	103933.50
6.	1	0	1	0	0	0	5	3	5	3	10920.8	14697.26250	-0.00059	14697.2631	404.8952	163940.15
7.	1	0	1	0	0	0	5	5	5	5	10920.8	14698.39000	-0.00257	14698.3926	404.8952	163940.15
8.	1	0	1	0	0	0	5	3	5	3	12324.2	0.00000	-14697.90044--	14697.9004	456.9271	208782.40
9.	1	0	1	0	0	0	5	5	5	5	12324.2	14699.33230	-0.00635	14699.3386	456.9271	208782.40
10.	1	0	1	0	0	0	7	1	5	1	0.0	15100.74080	-0.00561	15100.7464	0.0000	0.00
11.	1	0	1	0	0	0	7	1	5	1	6269.7	15101.72590	0.00485	15101.7210	232.4529	54034.35
12.	1	0	1	0	0	0	7	3	5	3	6269.7	15101.66660	0.00422	15101.6624	232.4529	54034.35
13.	1	0	1	0	0	0	7	5	5	5	6269.7	15101.54880	0.00415	15101.5446	232.4529	54034.35
14.	1	0	1	0	0	0	7	1	5	1	8695.4	15102.62450	0.00465	15102.6199	322.3872	103933.50
15.	1	0	1	0	0	0	7	3	5	3	8695.4	15102.51060	0.00280	15102.5078	322.3872	103933.50
16.	1	0	1	0	0	0	7	5	5	5	8695.4	15102.28560	0.00338	15102.2822	322.3872	103933.50
17.	1	0	1	0	0	0	7	1	5	1	10920.8	15103.69970	0.00056	15103.6991	404.8952	163940.15
18.	1	0	1	0	0	0	7	3	5	3	10920.8	15103.52500	0.00111	15103.5239	404.8952	163940.15
19.	1	0	1	0	0	0	7	5	5	5	10920.8	15103.17040	0.00067	15103.1697	404.8952	163940.15
20.	1	0	1	0	0	0	7	1	5	1	12324.2	15104.50190	-0.00264	15104.5045	456.9271	208782.40
21.	1	0	1	0	0	0	7	3	5	3	12324.2	15104.28100	-0.00179	15104.2828	456.9271	208782.40
22.	1	0	1	0	0	0	7	5	5	5	12324.2	15103.83220	-0.00114	15103.8333	456.9271	208782.40
23.	1	0	1	0	0	0	3	1	5	1	0.0	15275.89340	0.00020	15275.8932	0.0000	0.00
24.	1	0	1	0	0	0	3	1	5	1	6269.7	15276.73940	0.00338	15276.7360	232.4529	54034.35
25.	1	0	1	0	0	0	3	3	5	3	6269.7	15276.65350	0.00366	15276.6498	232.4529	54034.35
26.	1	0	1	0	0	0	3	1	5	1	8695.4	15277.51940	0.00392	15277.5155	322.3872	103933.50
27.	1	0	1	0	0	0	3	3	5	3	8695.4	15277.35310	0.00382	15277.3493	322.3872	103933.50
28.	1	0	1	0	0	0	3	1	5	1	10920.8	15278.45450	0.00022	15278.4543	404.8952	163940.15
29.	1	0	1	0	0	0	3	3	5	3	10920.8	15278.19200	0.00070	15278.1913	404.8952	163940.15
30.	1	0	1	0	0	0	3	1	5	1	12324.2	15279.15630	-0.00057	15279.1569	456.9271	208782.40
31.	1	0	1	0	0	0	3	3	5	3	12324.2	15278.82040	-0.00079	15278.8212	456.9271	208782.40

Standard deviation = 0.003045

ITERATION NO = 3 CONSTANTS, deviations and changes:

d = 26.971915593 +- 0.003323180 0.000000000

FINAL RESULTS OF LEAST SQUARES FITTING PROCEDURE
==

FITTED CONSTANTS:

```
A     /MHz      155094.63              1:Xab /MHz           0.
B     /MHz        7501.275744          1:XJ.a/kHz           0.
C     /MHz        7501.275744          1:XK.a/kHz           0.
DJ    /kHz           6.307543          1:XJbc/kHz           0.
DJK   /kHz          98.7688            1:Ma  /MHz          -0.017383
DK    /kHz        2689.14              1:Mb-c/MHz           0.
dJ    /kHz           0.                1:Tr  /MHz          -0.052223
dK    /kHz           0.                1:Xd  /kHz           0.
HJ    / Hz          -0.003452          1:Xbc /MHz           0.
HJK   / Hz           0.05847           1:Xac /MHz           0.
HKJ   / Hz           4.617
HK    / Hz           0.
hJ    / Hz           0.
hJK   / Hz           0.                Mu.a  /D             1.6406
hK    / Hz           0.                Mu.b  /D             0.
LKKJ  /mHz           0.                Mu.c  /D             0.
1:Xa  /MHz       -1934.1395             d    /cm           26.9719(33)
1:Xb-c/MHz           0.                 k    /cm            0.

1:X.bb /MHz        967.069750000000
1:X.cc /MHz        967.069750000000
```

WORST FITTED LINES:

	J	K_{-1}	K_{+1}	<- J	K_{-1}	K_{+1}	F	MF	<- F	MF	Volts	Obs	Obs-Calc	Calc	Field	Field**2
9.	1	0	1	0	0	0	5	5	5	5	12324.2	14699.33230	-0.00635	14699.3386	456.9271	208782.40
10.	1	0	1	0	0	0	7	1	5	1	0.0	15100.74080	-0.00561	15100.7464	0.0000	0.00
11.	1	0	1	0	0	0	7	1	5	1	6269.7	15101.72590	0.00485	15101.7210	232.4529	54034.35
14.	1	0	1	0	0	0	7	1	5	1	8695.4	15102.62450	0.00465	15102.6199	322.3872	103933.50
12.	1	0	1	0	0	0	7	3	5	3	6269.7	15101.66660	0.00422	15101.6624	232.4529	54034.35

```
///////////////////////////////////////////////////////////////////////////////////////////////
//////////////// cut here - please display and print using a fixed width font ///////////////
///////////////////////////////////////////////////////////////////////////////////////////////
```
