# Supplementary material for: Electric Dipole Moments from Stark Effect in Supersonic Expansion: n-Propanol, n-Butanol, and n-Butyl Cyanide
Source: Molecules. 2023 Feb 10;28(4):1692. doi: 10.3390/molecules28041692 (PMC9961461; doi:10.3390/molecules28041692)
Supplement: Supplementary file 1 [file molecules-28-01692-s001.zip › File_S2.pdf]

Supplementary file for the paper:

Krzysztof Habdas and Zbigniew Kisiel,
"Electric Dipole Moments from Stark Effect in Supersonic Expansion: n-propanol, n-butanol, and n-butyl cyanide"
Molecules 2023, 28, 1692. https://doi.org/10.3390/molecules28041692

//

Table S2. This file concatenates output files resulting from fitting rotational transition frequencies with constants in Watson's asymmetric rotor Hamiltonian for the use as fixed parameters in electric dipole moment determinations:

1/ The global fit of the literature Dreizler+Scappini, Z. Naturforsch. 36A, 1187 (1981) data and of current measurements for the Aa conformer of n-propanol (frequencies of A and E internal rotation components averaged when necessary).
1a/ Listing of frequencies measured for the A and E internal rotation components of the rotational transitions for Aa n-propanol.
2/ The refit of Kawashima et al. J.Phys.Chem.A 125, 1166 (2021) data for the TGt conformer of n-butanol
3/ The refit of Kawashima et al. J.Phys.Chem.A 125, 1166 (2021) data for the GTg' conformer of n-butanol
4/ The refit of Ordu et al. Astron. & Astrophys. 541, A121 (2012) data for the AA conformer of n-propyl cyanide
5/ The refit of Ordu et al. Astron. & Astrophys. 541, A121 (2012) data for the GA conformer of n-propyl cyanide

NOTE: the various outputs are separated by three lines as below, so that the text 'cut here' can be used to rapidly locate the different sections.

//
//////////////// cut here - please display and print using a fixed width font ///////////////
//

ASFIT version 19a.V.2020

n-propanol, Aa or TT conformer, ground vibrational state

Fit of Watson's Hamiltonian --> reduction-A, representation-I.r :

A	=	26401.4764974393219745252	+-	0.0025350265485648334
B	=	3802.1531789144214599219	+-	0.0003565902873607968
C	=	3549.4583278831873940362	+-	0.0004838164905461915
DJ	=	0.0009788248112188652	+-	0.0000026775694140950
DJK	=	-0.0006577073339374337	+-	0.0000579831830411016
DK	=	0.1512803826900017412	+-	0.0005428756800322036
dJ	=	0.0001254751077300517	+-	0.0000009388589714995
dK	=	-0.0063189912092134490	+-	0.0001570881489515189
HJ	=	-0.0000000273188744555	+-	0.0000000049569926021
HKJ	=	-0.0001326856510995219	+-	0.0000025061301897575
hJ	=	-0.0000000075759732997	+-	0.0000000017632298045
hK	=	0.0003362441254898326	+-	0.0000185816956150952

Deviation of fit = 0.051455
Weighted deviation of fit = 1.060267

	TRANSITION						Observed		Obs-Calc	Error	Note	Blend components

!
! H.Dreizler and F.Scappini, Z. Naturforsch. 36A, 1187-1191 (1981)
!

1.	1	1	1	0	0	0	29950.8000		0.0321	0.0500		
2.	1	1	0	1	0	1	22851.9100		0.0293	0.0500		
3.	2	1	2	1	0	1	37049.7700		0.1334	0.0500		
4.	3	1	3	2	1	2	21674.3200		-0.0297	0.0500		
5.B	4	3	1	3	3	0	29410.1700	B	-0.2074	0.1500	1.0E+00	29410.4104
6.B	4	3	2	3	3	1	29410.1700	B	-0.2074	0.1500	1.0E+00	29410.3444
7.	4	2	3	3	2	2	29404.6500		0.0955	0.0500		
8.	4	1	4	3	1	3	28896.6500		0.0491	0.0500		
9.	4	0	4	3	0	3	29385.1100		-0.0123	0.0500		
10.	5	1	4	4	1	3	37379.9400		-0.0255	0.0500		
11.	5	1	5	4	1	4	36116.6500		-0.0649	0.0500		
12.	5	0	5	4	1	4	15227.3900		0.0243	0.0500		
13.	5	0	5	4	0	4	36715.4400		-0.0106	0.0500		
14.	6	0	6	5	1	5	23145.8500		-0.0072	0.0500		
15.	7	0	7	6	1	6	31153.9700		-0.0680	0.0500		

```
  16.    10   1   9     9   2   8      12125.5900        0.0094   0.0500
  17.    11   1  10    10   2   9      20798.2800       -0.0281   0.0500
  18.    12   1  11    11   2  10      29577.2500       -0.0270   0.0500
  19.    13   1  12    12   2  11      38458.3100       -0.0375   0.0500
  20.    16   2  14    15   3  13       8490.9200       -0.0006   0.0500
  21.    17   2  15    16   3  14      16936.5700        0.0106   0.0500
!
  22.     3   2   2     4   1   3      37510.4600       -0.1045   0.0500
  23.     4   2   3     5   1   4      29535.1200       -0.0335   0.0500
  24.     5   2   4     6   1   5      21438.2500       -0.0833   0.0500
  25.     6   2   5     7   1   6      13221.7100       -0.0325   0.0500
  26.     8   0   8     7   1   7      39240.5600        0.0410   0.0500
  27.    10   3   8    11   2   9      31632.3400       -0.0815   0.0500
  28.    11   3   9    12   2  10      23850.2100       -0.0448   0.0500
  29.    12   3  10    13   2  11      15959.5900       -0.0109   0.0500
!
  30.     2   1   1     2   0   2      23106.7500        0.0414   0.0500
  31.     3   1   2     3   0   3      23492.9200        0.0320   0.0500
  32.     4   1   3     4   0   4      24015.1500        0.0278   0.0500
  33.     5   1   4     5   0   5      24679.6600        0.0229   0.0500
  34.     6   1   5     6   0   6      25494.1100       -0.0145   0.0500
  35.     7   1   6     7   0   7      26467.7200        0.0702   0.0500
  36.     8   1   7     8   0   8      27610.4800       -0.0302   0.0500
  37.     9   1   8     9   0   9      28933.9300       -0.1014   0.0500
  38.    10   1   9    10   0  10      30450.2400       -0.0489   0.0500
  39.    11   1  10    11   0  11      32171.7200       -0.0284   0.0500
  40.    12   1  11    12   0  12      34110.8400        0.0169   0.0500
  41.    13   1  12    13   0  13      36279.3400       -0.0186   0.0500
  42.E   14   1  13    14   0  14      38688.5600        0.4940   0.0500--excl
!
  43.E   15   1  14    15   1  15      30114.1000        4.3963   0.0500--excl   /?
  44.E   16   1  15    16   1  16      34062.0000        5.4972   0.0500--excl   /?
  45.E   17   1  16    17   1  17      38228.5300        5.7966   0.0500--excl   /?
!
!  IFPAN cavity FTMW
!
  46.     1   0   1     0   0   0       7351.6088        0.0012   0.0020
!
  47.     2   1   2     1   1   1      14450.4753       -0.0010   0.0020
  48.     2   0   2     1   0   1      14701.0839        0.0005   0.0020
  49.     2   1   1     1   1   0      14955.9097       -0.0015   0.0020
!
  50.     4   0   4     3   1   3       7408.5151       -0.0009   0.0020 /avg
!
  51.     1   1   1     2   0   2       7898.0796        0.0027   0.0020 /avg
!
  52.     5   0   5     4   1   4      15227.3674        0.0017   0.0020 /avg
!
!  IFPAN VDIx12 (all AE averaged, unless stated otherwise)
!
  53.    10   1  10     9   0   9      89742.7250       -0.0340   0.0500 /AE unresolved
  54.    11   1  11    10   0  10      95939.4927       -0.0415   0.0500
  55.    12   1  12    11   0  11     102089.6216       -0.0454   0.0500
  56.    13   1  13    12   0  12     108207.7733       -0.0305   0.0500
  57.    14   1  14    13   0  13     114308.7787        0.0019   0.0500
  58.    15   1  15    14   0  14     120407.1998        0.0487   0.0500
  59.    16   1  16    15   0  15     126516.7618        0.0068   0.0500
!
  60.    15   0  15    14   1  14      96872.2859       -0.0081   0.0500 /AE unresolved
  61.    17   0  17    16   1  16     113236.5554        0.0079   0.0500
  62.    18   0  18    17   1  17     121340.8504        0.0392   0.0500
  63.    19   0  19    18   1  18     129383.1961        0.0418   0.0500
  64.    20   0  20    19   1  19     137358.8425        0.0839   0.0500
!
  65.    19   1  18    18   2  17      93569.3769       -0.0722   0.0500
  66.    20   1  19    19   2  18     102988.0130       -0.0749   0.0500
  67.    21   1  20    20   2  19     112447.6029       -0.0384   0.0500
!
  68.     4   2   3     3   1   2      96822.4473       -0.0283   0.0500
  69.     5   2   4     4   1   3     103668.0949        0.1022   0.0500
  70.     6   2   5     5   1   4     110387.2890       -0.0518   0.0500
  71.     7   2   6     6   1   5     116981.1109       -0.0355   0.0500
!
  72.     6   2   4     5   1   5     114324.9306       -0.0418   0.0500
  73.     7   2   5     6   1   6     122551.8368        0.0133   0.0500
!
  74.    18   2  17    18   1  18      90373.1356        0.0071   0.0500
  75.    19   2  18    19   1  19      92873.9656        0.0436   0.0500
  76.    20   2  19    20   1  20      95506.5063        0.0343   0.0500
  77.    21   2  20    21   1  21      98268.9090        0.0060   0.0500
  78.    22   2  21    22   1  22     101158.9583        0.0318   0.0500
!
  79.E    6   3   3     6   2   4     113511.9896        0.3587   0.0500--excl
```

```
  80.E    7   3   4     7   2   5     113429.0557       0.4312   0.0500--excl
  81.E    8   3   5     8   2   6     113305.4259       0.2408   0.0500--excl
  82.E    9   3   6     9   2   7     113130.7757       0.1105   0.0500--excl
  83.    11   3   8    11   2   9     112582.8728       0.0204   0.0500
  84.    12   3   9    12   2  10     112186.5368       0.0414   0.0500
  85.    13   3  10    13   2  11     111693.9803       0.0830   0.0500
  86.    14   3  11    14   2  12     111095.5776       0.0645   0.0500
  87.    17   3  14    17   2  15     108601.1432       0.0099   0.0500
  88.    18   3  15    18   2  16     107529.1161       0.0351   0.0500
  89.    19   3  16    19   2  17     106341.3584       0.0052   0.0500
!
  90.E    7   3   5     7   2   6     113690.6943      -0.5153   0.0500--excl
  91.E    8   3   6     8   2   7     113740.5238      -0.2785   0.0500--excl
  92.E    9   3   7     9   2   8     113811.1442      -0.1405   0.0500--excl
  93.    10   3   8    10   2   9     113907.4469      -0.0853   0.0500
  94.    11   3   9    11   2  10     114034.7718      -0.0214   0.0500
  95.    12   3  10    12   2  11     114198.6216      -0.0175   0.0500
  96.    13   3  11    13   2  12     114404.8599      -0.0510   0.0500
  97.    14   3  12    14   2  13     114659.6612      -0.0020   0.0500
  98.    15   3  13    15   2  14     114969.0913      -0.0148   0.0500
  99.    16   3  14    16   2  15     115339.5135      -0.0329   0.0500
 100.    17   3  15    17   2  16     115777.2931      -0.0354   0.0500
 101.    18   3  16    18   2  17     116288.7361      -0.0387   0.0500
 102.    19   3  17    19   2  18     116880.0090      -0.1194   0.0500
!
!  IFPAN WG-FTMW (all AE averaged)
!
 103.     5   0   5     4   1   4      15227.3682       0.0025   0.0100
!
 104.     6   2   5     7   1   6      13221.7440       0.0015   0.0100
 105.     7   2   5     8   1   8      14243.7981      -0.0007   0.0100
 106.     8   2   6     9   1   9       8238.2854      -0.0062   0.0100
!
 107.    10   1   9     9   2   8      12125.5893       0.0087   0.0100
 108.    12   1  12    11   2   9       8441.1172       0.0012   0.0100
 109.    13   1  13    12   2  10      13489.7275       0.0082   0.0100
!
 110.    12   3  10    13   2  11      15959.5908      -0.0101   0.0100
 111.    13   3  10    14   2  13      11734.0068       0.0297   0.0100
!
 112.    16   2  14    15   3  13       8490.9161      -0.0044   0.0100
 113.    17   2  16    16   3  13       9046.0679      -0.0168   0.0100
 114.    17   2  15    16   3  14      16936.5736       0.0143   0.0100
 115.    18   2  17    17   3  14      15778.7901       0.0103   0.0100
----------------------------------------------------------------------------------------------------

 Sigma   =       0.051455                            103 Distinct lines in fit
 Sigma.w =       1.060267                             91 Degrees of freedom

 A    /MHz         26401.4765(25)          LJK /mHz           [ 0.]
 B    /MHz          3802.15318(36)         LKKJ/mHz           [ 0.]
 C    /MHz          3549.45833(48)         LK  /mHz           [ 0.]
  DJ  /kHz             0.9788(27)          lJ  /mHz           [ 0.]
  DJK /kHz            -0.658(58)           lJK /mHz           [ 0.]
  DK  /kHz           151.28(54)            lKJ /mHz           [ 0.]
  dJ  /kHz             0.12548(94)         lK  /mHz           [ 0.]
  dK  /kHz            -6.32(16)           PJ   /microHz       [ 0.]
 HJ   / Hz            -0.0273(50)         PJJK /microHz       [ 0.]
 HJK  / Hz           [ 0.]                PJK  /microHz       [ 0.]
 HKJ  / Hz         -132.7(25)             PKJ  /microHz       [ 0.]
 HK   / Hz           [ 0.]                PKKJ /microHz       [ 0.]
 hJ   / Hz            -0.0076(18)         PK   /microHz       [ 0.]
 hJK  / Hz           [ 0.]                pJ   /microHz       [ 0.]
 hK   / Hz           336.(19)             pJJK /microHz       [ 0.]
  LJ  /mHz           [ 0.]                pJK  /microHz       [ 0.]
  LJJK/mHz           [ 0.]                pKKJ /microHz       [ 0.]
                                          pK   /microHz       [ 0.]

 CORRELATION COEFFICIENTS, C.ij:

         A        B        C         DJ       DJK      DK       dJ       dK

 A        1.0000
 B        0.6099   1.0000
 C        0.3771  -0.1809   1.0000
  DJ      0.2747  -0.1186   0.7328   1.0000
  DJK     0.3038  -0.3219   0.5573   0.5557   1.0000
  DK      0.3406   0.6480  -0.3764  -0.1438  -0.5547   1.0000
  dJ      0.0722   0.2244  -0.3889  -0.0351  -0.1714   0.2029   1.0000
  dK     -0.0278   0.5997  -0.6538  -0.6282  -0.6887   0.6469  -0.1269   1.0000
 HJ       0.1542  -0.2145   0.6432   0.9577   0.4540  -0.1141   0.0612  -0.6549
 HKJ      0.4475   0.4324  -0.1908   0.0013   0.2350   0.5445  -0.1043   0.3843
 hJ       0.0596   0.2771  -0.4280  -0.0935  -0.2741   0.2793   0.9751  -0.0080
```

```
 hK      -0.0782   0.5758  -0.6799  -0.6365  -0.8567   0.7120   0.0934   0.9329

          HJ       HKJ      hJ       hK

 HJ       1.0000
 HKJ     -0.0544   1.0000
 hJ      -0.0080  -0.0874   1.0000
 hK      -0.6076   0.2211   0.2203   1.0000

 Mean value of |C.ij|, i.ne.j =   0.3684
 Mean value of  C.ij,  i.ne.j =   0.0803


 Distribution of values of |C.ij|, i.ne.j:

                 10        20        30        40        50        60
          ....,....|....,....|....,....|....,....|....,....|....,....|....,..
  5 <0.05 *****
  8 <0.10 ********
  5 <0.15 *****
  4 <0.20 ****
  6 <0.25 ******
  4 <0.30 ****
  3 <0.35 ***
  4 <0.40 ****
  3 <0.45 ***
  1 <0.50 *
  1 <0.55 *
  5 <0.60 *****
  7 <0.65 *******
  4 <0.70 ****
  2 <0.75 **
  0 <0.80
  0 <0.85
  1 <0.90 *
  1 <0.95 *
  2 <1.00 **
________________________________________________________________________________

 MOST INFLUENTIAL TRANSITIONS:
                                              Observed     Obs-Calc   hjj > 0.500

  115.    18   2  17    17   3  14      15778.7901        0.0103   0.6042
  114.    17   2  15    16   3  14      16936.5736        0.0143   0.5397
   51.     1   1   1     2   0   2       7898.0796        0.0027   0.5337
   78.    22   2  21    22   1  22     101158.9583        0.0318   0.5296

                                                  mean of all values = 0.1154

 WORST FITTING LINES (line number=(o-c)/error):
     111=  3.0      3=  2.7    102= -2.4     22= -2.1     69=  2.0     37= -2.0
________________________________________________________________________________


//////////////////////////////////////////////////////////////////////////////////////////////
/////////////// cut here - please display and print using a fixed width font ///////////////
//////////////////////////////////////////////////////////////////////////////////////////////
```

Internal rotation components of newly measured rotational transitions for
the Aa conformer of n-propanol and evaluation of their average frequencies.

n-propanol, Aa conformer						frequency /MHz A	E	(A+E)/2	E-A
IFPAN supersonic expansion Fabry-Perot cavity FTMW:									
4	0	4	3	1	3	7408.4275	7408.6026	7408.5151	0.1751
1	1	1	2	0	2	7897.9902	7898.1690	7898.0796	0.1788
5	0	5	4	1	4	15227.2807	15227.4543	15227.3675	0.1736
IFPAN room temperature MMW:									
19	1	18	18	2	17	93569.1358	93569.6180	93569.3769	0.4822
20	1	19	19	2	18	102987.8012	102988.2248	102988.0130	0.4236
21	1	20	20	2	19	112447.3806	112447.8251	112447.6029	0.4445
4	2	3	3	1	2	96822.7307	96822.1639	96822.4473	-0.5668
5	2	4	4	1	3	103668.3869	103667.8028	103668.0949	-0.5841
6	2	5	5	1	4	110387.5698	110387.0082	110387.2890	-0.5616
7	2	6	6	1	5	116981.4026	116980.8192	116981.1109	-0.5834

```
 6   2   4  5   1   5      114325.1758  114324.6854    114324.9306      -0.4904
 7   2   5  6   1   6      122552.1001  122551.5734    122551.8368      -0.5267

18   2  17 18   1  18       90373.4469   90372.8242     90373.1356      -0.6227
19   2  18 19   1  19       92874.2683   92873.6629     92873.9656      -0.6054
20   2  19 20   1  20       95506.8086   95506.2039     95506.5063      -0.6047
21   2  20 21   1  21       98269.2169   98268.6010     98268.9090      -0.6159
22   2  21 22   1  22      101159.2784  101158.6381    101158.9583      -0.6403

 8   3   5  8   2   6      113305.5472  113305.3045    113305.4259      -0.2427
 9   3   6  9   2   7      113131.0429  113130.5084    113130.7757      -0.5345
11   3   8 11   2   9      112583.2208  112582.5247    112582.8728      -0.6961
12   3   9 12   2  10      112186.9266  112186.1469    112186.5368      -0.7797
13   3  10 13   2  11      111694.3613  111693.5992    111693.9803      -0.7621
14   3  11 14   2  12      111095.9925  111095.1626    111095.5776      -0.8299
17   3  14 17   2  15      108601.5450  108600.7413    108601.1432      -0.8037
18   3  15 18   2  16      107529.4943  107528.7378    107529.1161      -0.7565
19   3  16 19   2  17      106341.7500  106340.9667    106341.3584      -0.7833

 7   3   5  7   2   6      113691.5818  113689.8067    113690.6943      -1.7751
 8   3   6  8   2   7      113741.1985  113739.8490    113740.5238      -1.3495
 9   3   7  9   2   8      113811.6992  113810.5892    113811.1442      -1.1100
10   3   8 10   2   9      113907.9304  113906.9634    113907.4469      -0.9670
11   3   9 11   2  10      114035.2530  114034.2905    114034.7718      -0.9625
12   3  10 12   2  11      114199.0373  114198.2059    114198.6216      -0.8314
13   3  11 13   2  12      114405.2590  114404.4607    114404.8599      -0.7983
14   3  12 14   2  13      114660.0728  114659.2495    114659.6612      -0.8233
15   3  13 15   2  14      114969.4997  114968.6829    114969.0913      -0.8168
16   3  14 16   2  15      115339.9161  115339.1109    115339.5135      -0.8052
17   3  15 17   2  16      115777.6934  115776.8928    115777.2931      -0.8006
18   3  16 18   2  17      116289.1346  116288.3375    116288.7361      -0.7971
19   3  17 19   2  18      116880.3882  116879.6297    116880.0090      -0.7585

IFPAN room temperature waveguide FTMW:

 5   0   5  4   1   4       15227.2830   15227.4530     15227.3680       0.1700
 6   2   5  7   1   6       13221.9807   13221.5072     13221.7440      -0.4735
 7   2   5  8   1   8       14244.0605   14243.5358     14243.7982      -0.5247
 8   2   6  9   1   9        8238.5519    8238.0189      8238.2854      -0.5330
10   1   9  9   2   8       12125.3492   12125.8294     12125.5893       0.4802
12   1  12 11   2   9        8440.8478    8441.3866      8441.1172       0.5388
13   1  13 12   2  10       13489.4549   13490.0000     13489.7275       0.5451
12   3  10 13   2  11       15960.0218   15959.1598     15959.5908      -0.8620
13   3  10 14   2  13       11734.3898   11733.6238     11734.0068      -0.7660
16   2  14 15   3  13        8490.5087    8491.3236      8490.9162       0.8149
17   2  16 16   3  13        9045.6787    9046.4570      9046.0679       0.7783
17   2  15 16   3  14       16936.1765   16936.9707     16936.5736       0.7942
18   2  17 17   3  14       15778.4059   15779.1743     15778.7901       0.7684
---------------------------------------------------------------------------------
---------------------------------------------------------------------------------


//////////////////////////////////////////////////////////////////////////////////////////////
//////////////// cut here - please display and print using a fixed width font ///////////////
//////////////////////////////////////////////////////////////////////////////////////////////

                                                     ASFIT version 19a.V.2020
________________________________________________________________________________

 n-butanol, TGt conformer
________________________________________________________________________________

 Fit of Watson's Hamiltonian --> reduction-A, representation-I.r  :


 A    =      12467.7415597117524157511   +-    0.0006046211760547080
 B    =       2371.5177110614044977410   +-    0.0001005589392009880
 C    =       2189.4793994294855110638   +-    0.0001106197995009905
  DJ  =          0.0007562594175625388   +-    0.0000006645587245262
  DJK =         -0.0061802838715994879   +-    0.0000101281817585809
  DK  =          0.0602546232279127791   +-    0.0001122876597563152
  dJ  =          0.0001248396139415317   +-    0.0000001311436928633
  dK  =          0.0020631499762923363   +-    0.0000346215608711245

          Deviation of fit =       0.002171
 Weighted deviation of fit =       0.943700

-----------------------------------------------------------------------------------------------------------
                 TRANSITION              Observed      Obs-Calc    Error  Note        Blend components
-----------------------------------------------------------------------------------------------------------
!
! AE-averaged transitions, Kawashima et al. J.Phys.Chem.A 125, 1166 (2021)
```

```
! 1/ only transitions with Ka <= 2 used in the fit
! 2/ measurement uncertainty set at that of the reported internal rotation fit
!
! mu_a
!
    1.     2   0   2     1   0   1       9119.5310        0.0000   0.0023
    2.     2   1   2     1   1   1       8939.9683       -0.0004   0.0023
    3.     2   1   1     1   1   0       9304.0213        0.0005   0.0023
    4.     3   0   3     2   0   2      13673.1569        0.0001   0.0023
    5.     3   1   3     2   1   2      13408.4010       -0.0002   0.0023
    6.     3   1   2     2   1   1      13954.4458        0.0018   0.0023
    7.     3   2   2     2   2   1      13683.0637        0.0057   0.0023
    8.     3   2   1     2   2   0      13692.8064       -0.0044   0.0023
    9.     4   0   4     3   0   3      18219.4341        0.0006   0.0023
   10.     4   1   4     3   1   3      17875.0009        0.0005   0.0023
   11.     4   1   3     3   1   2      18602.9337       -0.0005   0.0023
   12.     4   2   3     3   2   2      18242.0988        0.0020   0.0023
   13.     4   2   2     3   2   1      18266.4570       -0.0011   0.0023
   14.E    4   3   2     3   3   1      18249.0819        0.0264   0.0023--excl
   15.E    4   3   1     3   3   0      18249.1529       -0.0251   0.0023--excl
!
   16.     5   0   5     4   0   4      22755.9687        0.0006   0.0023
   17.     5   1   5     4   1   4      22339.2033        0.0007   0.0023
   18.     5   1   4     4   1   3      23248.8000        0.0006   0.0023
   19.     5   2   4     4   2   3      22799.4393        0.0004   0.0023
   20.     5   2   3     4   2   2      22848.0765       -0.0006   0.0023
   21.E    5   3   3     4   3   2      22813.3807        0.0469   0.0023--excl
   22.E    5   3   2     4   3   1      22813.7136       -0.0489   0.0023--excl
!
! mu_b
!
   23.     1   1   1     0   0   0      14657.1738       -0.0008   0.0023
   24.     1   1   0     1   0   1      10278.2083       -0.0013   0.0023
   25.     2   1   2     1   0   1      19036.1494        0.0001   0.0023
   26.     2   1   1     2   0   2      10462.6990       -0.0005   0.0023
   27.     3   1   3     2   0   2      23325.0204        0.0009   0.0023
   28.E    3   2   1     2   2   0      13692.8064       -0.0044   0.0023--excl   /ed mu_a
   29.     2   2   1     3   1   2      16333.4451       -0.0067   0.0023
   30.?    2   2   0     3   1   3      17428.0244        0.0095   0.0023 >3*Err
   31.     3   1   2     3   0   3      10743.9883        0.0017   0.0023
   32.     4   0   4     3   1   3       8567.5696       -0.0012   0.0023
   33.     3   2   2     4   1   3      11413.5767        0.0011   0.0023
   34.     4   1   3     4   0   4      11127.4884        0.0010   0.0023
   35.     5   0   5     4   1   4      13448.5388        0.0003   0.0023
   36.     4   2   2     5   1   5       9173.0778       -0.0029   0.0023
   37.     5   1   4     5   0   5      11620.3194        0.0007   0.0023
   38.     6   0   6     5   1   5      18389.8043        0.0000   0.0023
   39.E    5   3   3     6   2   4      23430.2820       -0.0462   0.0023--excl
   40.E    5   3   2     6   2   5      23601.0022        0.0553   0.0023--excl
   41.     6   1   5     6   0   6      12231.1567        0.0000   0.0023
   42.     7   0   7     6   1   6      23380.1483        0.0005   0.0023
   43.E    6   3   4     7   2   5      18766.7172       -0.0146   0.0023--excl
   44.E    6   3   3     7   2   6      19073.6149        0.0245   0.0023--excl
   45.     7   1   6     7   0   7      12969.9990       -0.0002   0.0023
   46.     8   1   7     7   2   6       9115.7017       -0.0014   0.0023
   47.E    7   3   5     8   2   6      14054.2466       -0.0038   0.0023--excl
   48.E    7   3   4     8   2   7      14564.8297        0.0122   0.0023--excl
   49.     8   1   7     8   0   8      13847.8012        0.0001   0.0023
   50.     9   1   8     8   2   7      14449.1419        0.0000   0.0023
   51.E    8   3   6     9   2   7       9280.5781        0.0002   0.0023--excl
   52.E    8   3   5     9   2   8      10080.5965        0.0070   0.0023--excl
   53.     9   1   8     9   0   9      14875.9622       -0.0009   0.0023
   54.    10   1   9     9   2   8      19856.7786       -0.0007   0.0023
   55.    10   1  10     9   2   7       9092.4042        0.0006   0.0023
   56.    10   1   9    10   0  10      16065.6680       -0.0002   0.0023
   57.    11   1  11    10   2   8      12209.6586       -0.0010   0.0023
   58.    11   1  10    11   0  11      17427.0968        0.0004   0.0023
   59.    12   1  11    12   0  12      18968.5752        0.0002   0.0023
!
! mu_c
!
   60.     1   1   0     0   0   0      14839.2024       -0.0013   0.0023
   61.     1   1   1     1   0   1      10096.1782       -0.0024   0.0023
   62.     2   1   1     1   0   1      19582.2288       -0.0016   0.0023
   63.     2   1   2     2   0   2       9916.6191        0.0008   0.0023
   64.     2   2   1     3   1   3      17425.5702       -0.0056   0.0023
   65.     2   2   0     3   1   2      16335.8954        0.0045   0.0023
   66.     3   1   3     3   0   3       9651.8631        0.0004   0.0023
   67.     4   0   4     3   1   2       7475.4460       -0.0008   0.0023
   68.     3   2   2     4   1   4      13233.6320       -0.0014   0.0023
   69.     3   2   1     4   1   3      11425.7686        0.0011   0.0023
   70.     4   1   4     4   0   4       9307.4301        0.0005   0.0023
   71.     5   0   5     4   1   3      11628.4800       -0.0007   0.0023
```

72.	4	2	3	5	1	5	9136.5272	-0.0004	0.0023
73.	5	1	5	5	0	5	8890.6639	-0.0002	0.0023
74.	6	0	6	5	1	4	15660.1486	-0.0011	0.0023
75.E	5	3	3	6	2	5	23600.3318	-0.0435	0.0023--excl
76.E	5	3	2	6	2	4	23430.9546	0.0547	0.0023--excl
77.	6	1	6	6	0	6	8410.6797	-0.0003	0.0023
78.	7	0	7	6	1	5	19559.6696	-0.0015	0.0023
79.E	6	3	4	7	2	6	19071.8617	-0.0145	0.0023--excl
80.E	6	3	3	7	2	5	18768.4715	0.0256	0.0023--excl
81.	7	1	7	7	0	7	7878.2219	-0.0011	0.0023
82.	8	0	8	7	1	6	23315.2418	-0.0001	0.0023
83.	8	1	7	7	2	5	8810.5580	-0.0007	0.0023
84.E	7	3	5	8	2	7	14560.5324	-0.0027	0.0023--excl
85.E	7	3	4	8	2	6	14058.5451	0.0122	0.0023--excl
86.	8	1	8	8	0	8	7305.4252	-0.0008	0.0023
87.	9	1	8	8	2	6	13942.8574	0.0002	0.0023
88.E	8	3	6	9	2	8	10071.1777	0.0006	0.0023--excl
89.E	8	3	5	9	2	7	9289.9977	0.0074	0.0023--excl
90.	10	1	10	9	2	8	9883.0040	0.0012	0.0023
91.	10	1	9	9	2	7	19066.1812	0.0010	0.0023
92.	11	1	11	10	2	9	13385.6161	-0.0008	0.0023
93.	12	1	12	11	2	10	16785.8375	0.0008	0.0023

Sigma = 0.002171 72 Distinct lines in fit
Sigma.w = 0.943700 64 Degrees of freedom

A /MHz	12467.74156(60)	LJK /mHz	[0.]
B /MHz	2371.51771(10)	LKKJ/mHz	[0.]
C /MHz	2189.47940(11)	LK /mHz	[0.]
DJ /kHz	0.75626(66)	lJ /mHz	[0.]
DJK /kHz	-6.180(10)	lJK /mHz	[0.]
DK /kHz	60.25(11)	lKJ /mHz	[0.]
dJ /kHz	0.12484(13)	lK /mHz	[0.]
dK /kHz	2.063(35)	PJ /microHz	[0.]
HJ / Hz	[0.]	PJJK /microHz	[0.]
HJK / Hz	[0.]	PJK /microHz	[0.]
HKJ / Hz	[0.]	PKJ /microHz	[0.]
HK / Hz	[0.]	PKKJ /microHz	[0.]
hJ / Hz	[0.]	PK /microHz	[0.]
hJK / Hz	[0.]	pJ /microHz	[0.]
hK / Hz	[0.]	pJJK /microHz	[0.]
LJ /mHz	[0.]	pJK /microHz	[0.]
LJJK/mHz	[0.]	pKKJ /microHz	[0.]
		pK /microHz	[0.]

CORRELATION COEFFICIENTS, C.ij:

	A	B	C	DJ	DJK	DK	dJ	dK
A	1.0000							
B	0.2500	1.0000						
C	0.3890	-0.1852	1.0000					
DJ	0.5509	0.5252	0.4221	1.0000				
DJK	0.5182	0.1622	0.4664	0.8309	1.0000			
DK	0.8180	0.0094	0.1296	0.2672	0.1756	1.0000		
dJ	-0.1393	0.4819	-0.4534	-0.0727	-0.3139	-0.0762	1.0000	
dK	-0.0684	0.6776	-0.7601	0.0569	-0.1758	-0.0412	0.3087	1.0000

Mean value of |C.ij|, i.ne.j = 0.3331
Mean value of C.ij, i.ne.j = 0.1698

Distribution of values of |C.ij|, i.ne.j:

```
                10        20        30        40        50        60
         ....,....|....,....|....,....|....,....|....,....|....,....|....,..
 2 <0.05 **
 4 <0.10 ****
 2 <0.15 **
 4 <0.20 ****
 1 <0.25 *
 1 <0.30 *
 2 <0.35 **
 1 <0.40 *
 1 <0.45 *
 3 <0.50 ***
 2 <0.55 **
 1 <0.60 *
 0 <0.65
 1 <0.70 *
 0 <0.75
 1 <0.80 *
```

```
  2 <0.85 **
  0 <0.90
  0 <0.95
  0 <1.00
________________________________________________________________________________

 MOST INFLUENTIAL TRANSITIONS:
                                               Observed     Obs-Calc   hjj > 0.500

   93.    12   1  12    11   2  10      16785.8375        0.0008   0.5636
   57.    11   1  11    10   2   8      12209.6586       -0.0010   0.5447
   59.    12   1  11    12   0  12      18968.5752        0.0002   0.5151

                                                   mean of all values = 0.1111

 WORST FITTING LINES (line number=(o-c)/error):
      30=  4.2     29= -2.9      7=  2.5     64= -2.4     65=  2.0      8= -1.9
________________________________________________________________________________


//////////////////////////////////////////////////////////////////////////////////////////////
/////////////// cut here - please display and print using a fixed width font //////////////
//////////////////////////////////////////////////////////////////////////////////////////////

                                                      ASFIT version 19a.V.2020
________________________________________________________________________________

 n-butanol, GTg' conformer
________________________________________________________________________________

 Fit of Watson's Hamiltonian --> reduction-A, representation-I.r  :


 A    =     12305.0000657480122754350   +-    0.0005715598920053754
 B    =      2330.5766385684919441701   +-    0.0003443007367714670
 C    =      2146.2345439343439466029   +-    0.0003176364174808935
  DJ  =         0.0007131166058370429   +-    0.0000015764864269479
  DJK =        -0.0025430610948640694   +-    0.0000160502414981471
  DK  =         0.0730058432919195727   +-    0.0002288265755256424
  dJ  =         0.0001370477996773269   +-    0.0000005840864793192
  dK  =         0.0024532564759884982   +-    0.0001494107941678149

          Deviation of fit =       0.001950
 Weighted deviation of fit =       0.696486

-----------------------------------------------------------------------------------------------------
                 TRANSITION              Observed      Obs-Calc     Error  Note        Blend components
-----------------------------------------------------------------------------------------------------
!
! Transitions from Kawaashima et al. J.Phys.Chem.A 125, 1166 (2021)
! 1/ in cases of resolved A,E splitting AE averaged transitions were used
! 2/ measurement uncertainty set at that of the reported internal rotation fit
!
! mu_a
!
   1.     1   0   1     0   0   0       4476.8080       -0.0003   0.0028
   2.     2   0   2     1   0   1       8951.0685        0.0000   0.0028
   3.     2   1   2     1   1   1       8769.2834        0.0016   0.0028
   4.     2   1   1     1   1   0       9137.9392        0.0016   0.0028
   5.     3   0   3     2   0   2      13420.2359        0.0000   0.0028
   6.     3   1   3     2   1   2      13152.3178        0.0003   0.0028
   7.     3   1   2     2   1   1      13705.2633        0.0003   0.0028
   8.     3   2   2     2   2   1      13430.4245        0.0069   0.0028
   9.     3   2   1     2   2   0      13440.5369       -0.0013   0.0028 /  avg
  10.     4   0   4     3   0   3      17881.7843        0.0012   0.0028
  11.     4   1   4     3   1   3      17533.4576        0.0002   0.0028
  12.     4   1   3     3   1   2      18270.5868        0.0008   0.0028
  13.     4   2   3     3   2   2      17905.1746       -0.0019   0.0028
  14.     4   2   2     3   2   1      17930.4531       -0.0023   0.0028
  15.     5   0   5     4   0   4      22333.2335        0.0016   0.0028
  16.     5   1   5     4   1   4      21912.1215        0.0013   0.0028
  17.     5   1   4     4   1   3      22833.1898        0.0011   0.0028
  18.     5   2   4     4   2   3      22378.1784       -0.0033   0.0028
  19.     5   2   3     4   2   2      22428.6454       -0.0025   0.0028
!
! mu_b
!
  20.     1   1   1     0   0   0      14451.1688       -0.0005   0.0028
  21.     1   1   0     1   0   1      10158.6909       -0.0012   0.0028
  22.     2   1   2     1   0   1      18743.6429        0.0001   0.0028
  23.     2   1   1     2   0   2      10345.5626        0.0013   0.0028
  24.     3   1   3     2   0   2      22944.8891       -0.0027   0.0028
```

```
  25.     3   1   2     3   0   3      10630.5896        0.0012   0.0028
  26.     4   0   4     3   1   3       8357.1264       -0.0008   0.0028
  27.     4   1   3     4   0   4      11019.3919        0.0006   0.0028
  28.     5   0   5     4   1   4      13156.9014       -0.0003   0.0028
  29.     5   1   4     5   0   5      11519.3459       -0.0022   0.0028
  30.     6   0   6     5   1   5      18016.9960        0.0009   0.0028
  31.     6   1   5     6   0   6      12139.4456        0.0020   0.0028 /  avg
  32.     7   0   7     6   1   6      22925.7899        0.0008   0.0028
  33.     7   1   6     7   0   7      12890.0134        0.0011   0.0028 /  avg
  34.     8   1   7     7   2   6       8843.0471        0.0010   0.0028
  35.     8   1   7     8   0   8      13782.3440       -0.0011   0.0028 /  avg
!
! mu_a
!
  36.     1   1   0     0   0   0      14635.5006        0.0001   0.0028
  37.     1   1   1     1   0   1       9974.3604       -0.0006   0.0028 /  avg
  38.     2   1   1     1   0   1      19296.6268       -0.0030   0.0028 /  avg
  39.     2   1   2     2   0   2       9792.5726       -0.0017   0.0028
  40.     3   1   2     2   0   2      24050.8256        0.0013   0.0028
  41.     3   1   3     3   0   3       9524.6563        0.0004   0.0028
  42.     4   0   4     3   1   2       7251.1930       -0.0017   0.0028
  43.     4   1   4     4   0   4       9176.3301       -0.0001   0.0028
  44.     5   0   5     4   1   3      11313.8418        0.0013   0.0028
  45.     5   1   5     5   0   5       8755.2186        0.0001   0.0028
  46.     6   0   6     5   1   4      15252.8670        0.0016   0.0028
  47.     6   1   6     6   0   6       8270.7724        0.0004   0.0028
  48.     7   0   7     6   1   5      19057.1179        0.0004   0.0028 /  avg
  49.     7   1   7     7   0   7       7734.1236        0.0018   0.0028
  50.     8   0   8     7   1   6      22714.4195       -0.0025   0.0028 /  avg
  51.     8   1   7     7   2   5       8526.4935       -0.0035   0.0028
  52.     8   1   8     8   0   8       7157.8148        0.0023   0.0028
  53.     9   1   8     8   2   6      13575.9004        0.0025   0.0028
--------------------------------------------------------------------------------------------------------

 Sigma   =       0.001950                              53 Distinct lines in fit
 Sigma.w =       0.696486                              45 Degrees of freedom

 A    /MHz         12305.00007(57)         LJK /mHz           [ 0.]
 B    /MHz          2330.57664(34)         LKKJ/mHz           [ 0.]
 C    /MHz          2146.23454(32)         LK  /mHz           [ 0.]
  DJ  /kHz             0.7131(16)          lJ  /mHz           [ 0.]
  DJK /kHz            -2.543(16)           lJK /mHz           [ 0.]
  DK  /kHz            73.01(23)            lKJ /mHz           [ 0.]
  dJ  /kHz             0.13705(58)         lK  /mHz           [ 0.]
  dK  /kHz             2.45(15)           PJ   /microHz       [ 0.]
 HJ   / Hz            [ 0.]               PJJK /microHz       [ 0.]
 HJK  / Hz            [ 0.]               PJK  /microHz       [ 0.]
 HKJ  / Hz            [ 0.]               PKJ  /microHz       [ 0.]
 HK   / Hz            [ 0.]               PKKJ /microHz       [ 0.]
 hJ   / Hz            [ 0.]               PK   /microHz       [ 0.]
 hJK  / Hz            [ 0.]               pJ   /microHz       [ 0.]
 hK   / Hz            [ 0.]               pJJK /microHz       [ 0.]
  LJ  /mHz            [ 0.]               pJK  /microHz       [ 0.]
  LJJK/mHz            [ 0.]               pKKJ /microHz       [ 0.]
                                          pK   /microHz       [ 0.]

 CORRELATION COEFFICIENTS, C.ij:

          A        B        C         DJ       DJK      DK       dJ       dK

 A        1.0000
 B        0.0367   1.0000
 C        0.1625  -0.8686   1.0000
  DJ      0.2089   0.7381  -0.3770   1.0000
  DJK     0.4899  -0.2311   0.4209   0.0814   1.0000
  DK      0.3471   0.1268  -0.0436   0.3075  -0.3699   1.0000
  dJ      0.1187   0.4364  -0.4563   0.1662  -0.1161   0.3666   1.0000
  dK     -0.0779   0.9539  -0.9409   0.6075  -0.3443   0.0560   0.2898   1.0000

 Mean value of |C.ij|, i.ne.j =   0.3479
 Mean value of  C.ij,  i.ne.j =   0.0746


 Distribution of values of |C.ij|, i.ne.j:

                  10        20        30        40        50        60
          ....,....|....,....|....,....|....,....|....,....|....,....|....,..
  2 <0.05 **
  3 <0.10 ***
  3 <0.15 ***
  2 <0.20 **
  2 <0.25 **
  1 <0.30 *
```

```
  3 <0.35 ***
  3 <0.40 ***
  2 <0.45 **
  2 <0.50 **
  0 <0.55
  0 <0.60
  1 <0.65 *
  0 <0.70
  1 <0.75 *
  0 <0.80
  0 <0.85
  1 <0.90 *
  1 <0.95 *
  1 <1.00 *
_______________________________________________________________________________

 MOST INFLUENTIAL TRANSITIONS:
                                         Observed     Obs-Calc   hjj > 0.500

   34.      8   1   7     7   2   6       8843.0471        0.0010   0.9529
   53.      9   1   8     8   2   6      13575.9004        0.0025   0.7138
   35.      8   1   7     8   0   8      13782.3440       -0.0011   0.5974
   50.      8   0   8     7   1   6      22714.4195       -0.0025   0.5412

                                             mean of all values = 0.1509

 WORST FITTING LINES (line number=(o-c)/error):
       8=  2.5     51= -1.3     18= -1.2     38= -1.1     24= -1.0     53=  0.9
_______________________________________________________________________________


/////////////////////////////////////////////////////////////////////////////////////////////
//////////////// cut here - please display and print using a fixed width font //////////////
/////////////////////////////////////////////////////////////////////////////////////////////


AA_valeronitrile, A-reduction                          Thu Dec 29 17:46:55 2022

-------------------------------------------------------------------------------------=========
                                             obs         o-c     error     blends     Notes
                                                                         o-c      wt
    / instead of : below denotes (o-c)>3*err
-------------------------------------------------------------------------------------=========
   1:  2  1  2  0  2    1  1  1  0  1         5125.0200  -0.0041  0.002
   2:  2  1  2  0  2    1  1  1  0  2         5125.2220  -0.0045  0.002
   3:  2  1  2  0  3    1  1  1  0  2         5125.8850  -0.0008  0.002
   4:  2  1  2  0  1    1  1  1  0  1         5126.0470  -0.0029  0.002
   5:  2  1  2  0  1    1  1  1  0  0         5126.5540  -0.0021  0.002
   6:  2  0  2  0  2    1  0  1  0  2         5194.8340  -0.0009  0.002
   7:  2  0  2  0  1    1  0  1  0  0         5194.9720  -0.0011  0.002
   8:  2  0  2  0  3    1  0  1  0  2         5195.7120  -0.0004  0.002
   9:  2  0  2  0  1    1  0  1  0  1         5197.0160  -0.0021  0.002
  10:  2  1  1  0  2    1  1  0  0  1         5265.5200   0.0000  0.002
  11:  2  1  1  0  3    1  1  0  0  2         5266.3520  -0.0003  0.002
  12:  2  1  1  0  1    1  1  0  0  0         5267.3950  -0.0013  0.002
  13:  2  1  2  0  3    3  0  3  0  4         5831.5480  -0.0058  0.002
  14:  2  1  2  0  1    3  0  3  0  2         5831.5980  -0.0031  0.002
  15/  2  1  2  0  2    3  0  3  0  3         5831.8000  -0.0062  0.002
  16:  3  1  3  0  3    2  1  2  0  2         7688.2110  -0.0011  0.002
  17:  3  1  3  0  4    2  1  2  0  3         7688.4640  -0.0001  0.002
  18:  3  0  3  0  2    2  0  2  0  1         7792.6590  -0.0035  0.002
  19:  3  0  3  0  3    2  0  2  0  2         7792.7960  -0.0007  0.002
  20:  3  0  3  0  4    2  0  2  0  3         7792.8300  -0.0010  0.002
  21:  3  1  2  0  3    2  1  1  0  2         7898.9520  -0.0007  0.002
  22:  3  1  2  0  4    2  1  1  0  3         7899.1860  -0.0023  0.002
  23:  1  1  1  0  0    2  0  2  0  1         8497.7040  -0.0035  0.003
  24:  1  1  1  0  1    2  0  2  0  1         8498.2160   0.0023  0.003
  25:  1  1  1  0  2    2  0  2  0  3         8498.4980  -0.0009  0.003
  26:  1  1  1  0  2    2  0  2  0  2         8499.3780   0.0015  0.003
  27:  1  1  1  0  1    2  0  2  0  2         8499.5750  -0.0039  0.003
  28:  4  1  4  0  4    3  1  3  0  3        10250.7850   0.0033  0.004
  29:  4  1  4  0  5    3  1  3  0  4        10250.8940  -0.0018  0.002
  30:  4  0  4  0  3    3  0  3  0  2        10389.0680   0.0019  0.003
  31:  4  0  4  0  4    3  0  3  0  3        10389.1170  -0.0046  0.003
  32:  4  0  4  0  5    3  0  3  0  4        10389.1440  -0.0007  0.003
  33:  4  1  3  0  4    3  1  2  0  4        10531.3080  -0.0005  0.002
  34:  4  1  3  0  4    3  1  2  0  3        10531.7610  -0.0002  0.002
  35:  4  1  3  0  3    3  1  2  0  2        10531.8450  -0.0046  0.004
  36:  4  1  3  0  5    3  1  2  0  4        10531.8630  -0.0020  0.002
  37:  5  1  5  0  5    4  1  4  0  4        12813.0040  -0.0008  0.002
  38:  5  1  5  0  6    4  1  4  0  5        12813.0670  -0.0015  0.002
  39:  5  1  4  0  5    4  1  3  0  4        13164.2130   0.0003  0.003
  40:  5  1  4  0  6    4  1  3  0  5        13164.2700   0.0008  0.003
```

```
  41:  1  1  0  0  1    1  0  1  0  0        13763.7780  -0.0014  0.002
  42:  1  1  0  0  0    1  0  1  0  1        13764.2830  -0.0030  0.002
  43:  1  1  0  0  2    1  0  1  0  2        13764.3855  -0.0056  0.002
  44:  1  1  0  0  1    1  0  1  0  2        13765.0050  -0.0015  0.002
  45:  1  1  0  0  2    1  0  1  0  1        13765.2085  -0.0006  0.002
  46:  1  1  0  0  1    1  0  1  0  1        13765.8220  -0.0024  0.002
  47:  1  1  1  0  0    0  0  0  0  1        16292.0040  -0.0034  0.003
  48/  1  1  1  0  2    0  0  0  0  1        16292.3020  -0.0092  0.003
  49:  1  1  1  0  1    0  0  0  0  1        16292.5090  -0.0046  0.003
  50:  2  1  2  0  1    1  0  1  0  0        18819.2380   0.0012  0.003
  51:  2  1  2  0  2    1  0  1  0  2        18819.4340  -0.0040  0.003
  52:  2  1  2  0  3    1  0  1  0  2        18820.0900  -0.0073  0.003
!
! hyperfine unresolved
!
  53:  7  1  7  1  7    6  1  6  1  6        17936.3110  -0.0058  0.003
  54:  7  0  7  1  7    6  0  6  1  6        18170.4650  -0.0032  0.003
  55:  7  2  6  1  7    6  2  5  1  6        18183.8750   0.0002  0.003
  56:  7  2  5  1  7    6  2  4  1  6        18198.9520  -0.0023  0.003
  57:  7  1  6  1  7    6  1  5  1  6        18427.9130  -0.0028  0.003
  58:  8  1  8  1  8    7  1  7  1  7        20497.2700  -0.0060  0.003
  59:  8  2  7  1  8    7  2  6  1  7        20780.6000  -0.0032  0.003
  60:  8  2  6  1  8    7  2  5  1  7        20803.2040  -0.0030  0.003
  61:  8  1  7  1  8    7  1  6  1  7        21059.0090  -0.0034  0.003
  62: 29  2 28  1 29   28  2 27  1 28        75150.3124   0.0044  0.020
  63: 29  7 23  1 29   28  7 22  1 28        75372.2122  -0.0056  0.020  -0.0057 0.50
  64: 29  7 22  1 29   28  7 21  1 28        75372.2122  -0.0056  0.020  -0.0057 0.50
  65: 29  8 22  1 29   28  8 21  1 28        75373.3514   0.0027  0.020   0.0027 0.50
  66: 29  8 21  1 29   28  8 20  1 28        75373.3514   0.0027  0.020   0.0027 0.50
  67: 29  6 23  1 29   28  6 22  1 28        75374.9918  -0.0087  0.020  -0.0082 0.50
  68: 29  6 24  1 29   28  6 23  1 28        75374.9918  -0.0076  0.020  -0.0082 0.50
  69: 29  9 21  1 29   28  9 20  1 28        75377.0851  -0.0011  0.020  -0.0011 0.50
  70: 29  9 20  1 29   28  9 19  1 28        75377.0851  -0.0011  0.020  -0.0011 0.50
  71: 29 10 19  1 29   28 10 18  1 28        75382.7668   0.0080  0.020   0.0080 0.50
  72: 29 10 20  1 29   28 10 19  1 28        75382.7668   0.0080  0.020   0.0080 0.50
  73: 29  5 24  1 29   28  5 23  1 28        75384.5534  -0.0290  0.020   0.0150 0.50
  74: 29  5 25  1 29   28  5 24  1 28        75384.5534   0.0591  0.020   0.0150 0.50
  75: 29 11 18  1 29   28 11 17  1 28        75389.9942   0.0034  0.020   0.0034 0.50
  76: 29 11 19  1 29   28 11 18  1 28        75389.9942   0.0034  0.020   0.0034 0.50
  77: 29 12 17  1 29   28 12 16  1 28        75398.5559  -0.0025  0.020  -0.0026 0.50
  78: 29 12 18  1 29   28 12 17  1 28        75398.5559  -0.0025  0.020  -0.0026 0.50
  79: 29  4 26  1 29   28  4 25  1 28        75406.0193  -0.0179  0.020
  80: 29 13 17  1 29   28 13 16  1 28        75408.3111  -0.0102  0.020  -0.0103 0.50
  81: 29 13 16  1 29   28 13 15  1 28        75408.3111  -0.0102  0.020  -0.0103 0.50
  82: 29  4 25  1 29   28  4 24  1 28        75410.2691  -0.0195  0.020
  83: 29  3 27  1 29   28  3 26  1 28        75418.3777  -0.0103  0.020
  84: 29 14 15  1 29   28 14 14  1 28        75419.1885   0.0011  0.020   0.0012 0.50
  85: 29 14 16  1 29   28 14 15  1 28        75419.1885   0.0011  0.020   0.0012 0.50
  86: 29 15 14  1 29   28 15 13  1 28        75431.1051   0.0114  0.020   0.0114 0.50
  87: 29 15 15  1 29   28 15 14  1 28        75431.1051   0.0114  0.020   0.0114 0.50
  88: 29 16 14  1 29   28 16 13  1 28        75443.9888  -0.0075  0.020  -0.0075 0.50
  89: 29 16 13  1 29   28 16 12  1 28        75443.9888  -0.0075  0.020  -0.0075 0.50
  90: 29 17 12  1 29   28 17 11  1 28        75457.8596  -0.0035  0.020  -0.0035 0.50
  91: 29 17 13  1 29   28 17 12  1 28        75457.8596  -0.0035  0.020  -0.0035 0.50
  92: 29 19 10  1 29   28 19  9  1 28        75488.3738  -0.0250  0.020  -0.0251 0.50
  93: 29 19 11  1 29   28 19 10  1 28        75488.3738  -0.0250  0.020  -0.0251 0.50
  94: 29 20  9  1 29   28 20  8  1 28        75505.0304  -0.0042  0.020  -0.0043 0.50
  95: 29 20 10  1 29   28 20  9  1 28        75505.0304  -0.0042  0.020  -0.0043 0.50
  96: 29 21  8  1 29   28 21  7  1 28        75522.5412  -0.0243  0.020  -0.0244 0.50
  97: 29 21  9  1 29   28 21  8  1 28        75522.5412  -0.0243  0.020  -0.0244 0.50
  98: 29  3 26  1 29   28  3 25  1 28        75523.4671   0.0183  0.020
  99: 29 22  7  1 29   28 22  6  1 28        75541.0000   0.0186  0.020   0.0186 0.50
 100: 29 22  8  1 29   28 22  7  1 28        75541.0000   0.0186  0.020   0.0186 0.50
 101: 29 25  4  1 29   28 25  3  1 28        75601.4450  -0.0096  0.020  -0.0096 0.50
 102: 29 25  5  1 29   28 25  4  1 28        75601.4450  -0.0096  0.020  -0.0096 0.50
 103: 40  3 38  1 40   40  2 39  1 40        75656.1573  -0.0573  0.060
 104: 29  1 28  1 29   28  1 27  1 28        76016.9392  -0.0001  0.020
 105: 29  2 27  1 29   28  2 26  1 28        76037.3632   0.0109  0.020
 106: 28  1 28  1 28   27  0 27  1 27        76461.3410  -0.0195  0.020
 107: 30  1 30  1 30   29  1 29  1 29        76630.4288   0.0018  0.020
 108: 76  3 73  1 76   76  2 74  1 76        76851.1940  -0.0576  0.060
 109: 42  3 40  1 42   42  2 41  1 42        76948.9910   0.0161  0.040
 110: 30  0 30  1 30   29  0 29  1 29        76959.1491  -0.0087  0.020
 111: 43  3 41  1 43   43  2 42  1 43        77648.3735  -0.0102  0.040
 112: 30  2 29  1 30   29  2 28  1 29        77727.9318   0.0039  0.020
 113: 30  7 24  1 30   29  7 23  1 29        77971.8829   0.0091  0.020   0.0092 0.50
 114: 30  7 23  1 30   29  7 22  1 29        77971.8829   0.0091  0.020   0.0092 0.50
 115: 30  8 22  1 30   29  8 21  1 29        77972.6515   0.0010  0.020   0.0010 0.50
 116: 30  8 23  1 30   29  8 22  1 29        77972.6515   0.0010  0.020   0.0010 0.50
 117: 30  6 25  1 30   29  6 24  1 29        77975.3583  -0.0068  0.020  -0.0076 0.50
 118: 30  6 24  1 30   29  6 23  1 29        77975.3583  -0.0084  0.020  -0.0076 0.50
 119: 30  9 22  1 30   29  9 21  1 29        77976.2598   0.0101  0.020   0.0101 0.50
 120: 30  9 21  1 30   29  9 20  1 29        77976.2598   0.0101  0.020   0.0101 0.50
```

121:	30	10	21	1	30	29	10	20	1	29	77981.9226	-0.0049	0.020	-0.0049	0.50
122:	30	10	20	1	30	29	10	19	1	29	77981.9226	-0.0049	0.020	-0.0049	0.50
123:	30	5	25	1	30	29	5	24	1	29	77986.2741	-0.0641	0.020	-0.0043	0.50
124:	30	5	26	1	30	29	5	25	1	29	77986.2741	0.0555	0.020	-0.0043	0.50
125:	30	11	19	1	30	29	11	18	1	29	77989.2570	-0.0114	0.020	-0.0114	0.50
126:	30	11	20	1	30	29	11	19	1	29	77989.2570	-0.0114	0.020	-0.0114	0.50
127:	30	12	19	1	30	29	12	18	1	29	77998.0198	-0.0049	0.020	-0.0049	0.50
128:	30	12	18	1	30	29	12	17	1	29	77998.0198	-0.0049	0.020	-0.0049	0.50
129:	30	13	18	1	30	29	13	17	1	29	78008.0374	-0.0036	0.020	-0.0036	0.50
130:	30	13	17	1	30	29	13	16	1	29	78008.0374	-0.0036	0.020	-0.0036	0.50
131:	30	4	27	1	30	29	4	26	1	29	78010.0763	0.0145	0.020		
132:	30	4	26	1	30	29	4	25	1	29	78015.4673	0.0213	0.020		
133:	30	14	17	1	30	29	14	16	1	29	78019.2182	0.0027	0.020	0.0027	0.50
134:	30	14	16	1	30	29	14	15	1	29	78019.2182	0.0027	0.020	0.0027	0.50
135:	30	3	28	1	30	29	3	27	1	29	78020.7911	0.0018	0.020		
136:	30	15	15	1	30	29	15	14	1	29	78031.4899	0.0112	0.020	0.0112	0.50
137:	30	15	16	1	30	29	15	15	1	29	78031.4899	0.0112	0.020	0.0112	0.50
138:	30	16	14	1	30	29	16	13	1	29	78044.7592	-0.0228	0.020	-0.0228	0.50
139:	30	16	15	1	30	29	16	14	1	29	78044.7592	-0.0228	0.020	-0.0228	0.50
140:	30	17	13	1	30	29	17	12	1	29	78059.0949	0.0048	0.020	0.0048	0.50
141:	30	17	14	1	30	29	17	13	1	29	78059.0949	0.0048	0.020	0.0048	0.50
142:	30	18	13	1	30	29	18	12	1	29	78074.3654	-0.0110	0.020	-0.0110	0.50
143:	30	18	12	1	30	29	18	11	1	29	78074.3654	-0.0110	0.020	-0.0110	0.50
144:	30	19	12	1	30	29	19	11	1	29	78090.6137	-0.0071	0.020	-0.0071	0.50
145:	30	19	11	1	30	29	19	10	1	29	78090.6137	-0.0071	0.020	-0.0071	0.50
146:	30	20	10	1	30	29	20	9	1	29	78107.8287	0.0215	0.020	0.0215	0.50
147:	30	20	11	1	30	29	20	10	1	29	78107.8287	0.0215	0.020	0.0215	0.50
148:	30	21	10	1	30	29	21	9	1	29	78125.9026	-0.0197	0.020	-0.0197	0.50
149:	30	21	9	1	30	29	21	8	1	29	78125.9026	-0.0197	0.020	-0.0197	0.50
150:	50	1	49	1	50	50	0	50	1	50	78138.1014	-0.0163	0.020		
151:	30	3	27	1	30	29	3	26	1	29	78144.4998	-0.0212	0.020		
152:	30	22	8	1	30	29	22	7	1	29	78145.0006	0.0452	0.040	0.0453	0.50
153:	30	22	9	1	30	29	22	8	1	29	78145.0006	0.0452	0.040	0.0453	0.50
154:	30	24	6	1	30	29	24	5	1	29	78185.7741	0.0363	0.020	0.0364	0.50
155:	30	24	7	1	30	29	24	6	1	29	78185.7741	0.0363	0.020	0.0364	0.50
156:	30	25	6	1	30	29	25	5	1	29	78207.4119	-0.0589	0.040	-0.0590	0.50
157:	30	25	5	1	30	29	25	4	1	29	78207.4119	-0.0589	0.040	-0.0590	0.50
158:	30	26	4	1	30	29	26	3	1	29	78229.9965	-0.0920	0.060	-0.0921	0.50
159:	30	26	5	1	30	29	26	4	1	29	78229.9965	-0.0920	0.060	-0.0921	0.50
160:	44	3	42	1	44	44	2	43	1	44	78383.7241	-0.0093	0.040		
161:	32	0	32	1	32	31	1	31	1	31	78437.2116	-0.0415	0.040		
162:	30	1	29	1	30	29	1	28	1	29	78609.2793	-0.0093	0.020		
163:	29	1	29	1	29	28	0	28	1	28	78640.4255	-0.0058	0.020		
164:	30	2	28	1	30	29	2	27	1	29	78686.5793	0.0104	0.020		
165:	63	2	61	1	63	63	1	62	1	63	78698.3818	-0.0100	0.040		
166:	45	3	43	1	45	45	2	44	1	45	79155.4120	0.0309	0.040		
167:	31	1	31	1	31	30	1	30	1	30	79170.7763	0.0405	0.040		
168:	31	0	31	1	31	30	0	30	1	30	79481.5232	0.0185	0.040		
169:	31	2	30	1	31	30	2	29	1	30	80304.2051	-0.0101	0.020		
170:	31	7	24	1	31	30	7	23	1	30	80571.5788	-0.0127	0.020	-0.0127	0.50
171:	31	7	25	1	31	30	7	24	1	30	80571.5788	-0.0126	0.020	-0.0127	0.50
172:	31	8	24	1	31	30	8	23	1	30	80571.9398	-0.0345	0.020	-0.0345	0.50
173:	31	8	23	1	31	30	8	22	1	30	80571.9398	-0.0345	0.020	-0.0345	0.50
174:	31	9	22	1	31	30	9	21	1	30	80575.3752	-0.0328	0.020	-0.0328	0.50
175:	31	9	23	1	31	30	9	22	1	30	80575.3752	-0.0328	0.020	-0.0328	0.50
176:	31	6	25	1	31	30	6	24	1	30	80575.9090	0.0515	0.040	0.0527	0.50
177:	31	6	26	1	31	30	6	25	1	30	80575.9090	0.0538	0.040	0.0527	0.50
178:	31	10	21	1	31	30	10	20	1	30	80581.0633	-0.0086	0.020	-0.0086	0.50
179:	31	10	22	1	31	30	10	21	1	30	80581.0633	-0.0086	0.020	-0.0086	0.50
180:	31	12	19	1	31	30	12	18	1	30	80597.4539	0.0123	0.020	0.0123	0.50
181:	31	12	20	1	31	30	12	19	1	30	80597.4539	0.0123	0.020	0.0123	0.50
182:	31	13	18	1	31	30	13	17	1	30	80607.7170	0.0141	0.020	0.0141	0.50
183:	31	13	19	1	31	30	13	18	1	30	80607.7170	0.0141	0.020	0.0141	0.50
184:	31	4	28	1	31	30	4	27	1	30	80614.3517	-0.0589	0.040		
185:	31	14	17	1	31	30	14	16	1	30	80619.1584	-0.0206	0.020	-0.0207	0.50
186:	31	14	18	1	31	30	14	17	1	30	80619.1584	-0.0206	0.020	-0.0207	0.50
187:	31	4	27	1	31	30	4	26	1	30	80621.2221	0.0475	0.040		
188:	31	3	29	1	31	30	3	28	1	30	80622.9631	0.0098	0.020		
189:	31	15	16	1	31	30	15	15	1	30	80631.8100	0.0162	0.020	0.0163	0.50
190:	31	15	17	1	31	30	15	16	1	30	80631.8100	0.0162	0.020	0.0163	0.50
191:	31	16	16	1	31	30	16	15	1	30	80645.4959	0.0025	0.020	0.0026	0.50
192:	31	16	15	1	31	30	16	14	1	30	80645.4959	0.0025	0.020	0.0026	0.50
193:	31	18	13	1	31	30	18	12	1	30	80676.0062	0.0047	0.020	0.0048	0.50
194:	31	18	14	1	31	30	18	13	1	30	80676.0062	0.0047	0.020	0.0048	0.50
195:	31	19	13	1	31	30	19	12	1	30	80692.8133	0.0545	0.040	0.0546	0.50
196:	31	19	12	1	31	30	19	11	1	30	80692.8133	0.0545	0.040	0.0546	0.50
197:	31	22	10	1	31	30	22	9	1	30	80748.8197	-0.0193	0.020	-0.0193	0.50
198:	31	22	9	1	31	30	22	8	1	30	80748.8197	-0.0193	0.020	-0.0193	0.50
199:	31	3	28	1	31	30	3	27	1	30	80767.7199	-0.0402	0.020		
200:	31	23	8	1	31	30	23	7	1	30	80769.4334	0.0052	0.020	0.0053	0.50
201:	31	23	9	1	31	30	23	8	1	30	80769.4334	0.0052	0.020	0.0053	0.50
202:	31	1	30	1	31	30	1	29	1	30	81198.2334	-0.0063	0.020		
203:	33	0	33	1	33	32	1	32	1	32	81250.2650	0.0276	0.020		

204:	31	2	29	1	31	30	2	28	1	30	81335.4597	-0.0077	0.020		
205:	47	2	46	1	47	47	1	47	1	47	81678.6884	-0.0162	0.040		
206:	48	3	46	1	48	48	2	47	1	48	81690.6465	-0.0035	0.040		
207:	32	1	32	1	32	31	1	31	1	31	81710.1163	0.0021	0.020		
208:	32	0	32	1	32	31	0	31	1	31	82002.7470	0.0070	0.020		
209:	49	3	47	1	49	49	2	48	1	49	82609.7096	0.0302	0.040		
210:	32	2	31	1	32	31	2	30	1	31	82879.1415	0.0008	0.020		
211:	31	1	31	1	31	30	0	30	1	30	83047.0009	0.0094	0.020		
212:	32	8	25	1	32	31	8	24	1	31	83171.3489	0.0281	0.020	0.0020	0.25
213:	32	8	24	1	32	31	8	23	1	31	83171.3489	0.0281	0.020	0.0020	0.25
214:	32	7	26	1	32	31	7	25	1	31	83171.3489	-0.0241	0.020	0.0020	0.25
215:	32	7	25	1	32	31	7	24	1	31	83171.3489	-0.0242	0.020	0.0020	0.25
216:	32	9	23	1	32	31	9	22	1	31	83174.5467	-0.0143	0.020	-0.0143	0.50
217:	32	9	24	1	32	31	9	23	1	31	83174.5467	-0.0143	0.020	-0.0143	0.50
218:	32	6	26	1	32	31	6	25	1	31	83176.4813	0.0044	0.020	0.0061	0.50
219:	32	6	27	1	32	31	6	26	1	31	83176.4813	0.0077	0.020	0.0061	0.50
220:	32	10	22	1	32	31	10	21	1	31	83180.1868	-0.0042	0.020	-0.0043	0.50
221:	32	10	23	1	32	31	10	22	1	31	83180.1868	-0.0042	0.020	-0.0043	0.50
222:	32	11	21	1	32	31	11	20	1	31	83187.7034	-0.0032	0.020	-0.0032	0.50
223:	32	11	22	1	32	31	11	21	1	31	83187.7034	-0.0032	0.020	-0.0032	0.50
224:	32	5	28	1	32	31	5	27	1	31	83190.3941	0.0389	0.020		
225:	32	5	27	1	32	31	5	26	1	31	83190.5687	-0.0006	0.020		
226:	32	12	20	1	32	31	12	19	1	31	83196.8066	-0.0007	0.020	-0.0008	0.50
227:	32	12	21	1	32	31	12	20	1	31	83196.8066	-0.0007	0.020	-0.0008	0.50
228:	32	13	20	1	32	31	13	19	1	31	83207.2847	-0.0202	0.020	-0.0203	0.50
229:	32	13	19	1	32	31	13	18	1	31	83207.2847	-0.0202	0.020	-0.0203	0.50
230:	32	14	19	1	32	31	14	18	1	31	83219.0753	-0.0006	0.020	-0.0021	0.33
231:	32	14	18	1	32	31	14	17	1	31	83219.0753	-0.0006	0.020	-0.0021	0.33
232:	32	4	29	1	32	31	4	28	1	31	83219.0753	-0.0049	0.020	-0.0021	0.33
233:	32	3	30	1	32	31	3	29	1	31	83224.8171	-0.0016	0.020		
234:	32	4	28	1	32	31	4	27	1	31	83227.5104	-0.0027	0.020		
235:	32	15	17	1	32	31	15	16	1	31	83232.0290	-0.0075	0.020	-0.0075	0.50
236:	32	15	18	1	32	31	15	17	1	31	83232.0290	-0.0075	0.020	-0.0075	0.50
237:	32	16	16	1	32	31	16	15	1	31	83246.1305	0.0028	0.020	0.0028	0.50
238:	32	16	17	1	32	31	16	16	1	31	83246.1305	0.0028	0.020	0.0028	0.50
239:	32	17	16	1	32	31	17	15	1	31	83261.2976	-0.0093	0.020	-0.0093	0.50
240:	32	17	15	1	32	31	17	14	1	31	83261.2976	-0.0093	0.020	-0.0093	0.50
241:	32	18	14	1	32	31	18	13	1	31	83277.5400	-0.0024	0.020	-0.0024	0.50
242:	32	18	15	1	32	31	18	14	1	31	83277.5400	-0.0024	0.020	-0.0024	0.50
243:	32	19	13	1	32	31	19	12	1	31	83294.8062	-0.0036	0.020	-0.0037	0.50
244:	32	19	14	1	32	31	19	13	1	31	83294.8062	-0.0036	0.020	-0.0037	0.50
245:	32	20	12	1	32	31	20	11	1	31	83313.0995	0.0093	0.020	0.0094	0.50
246:	32	20	13	1	32	31	20	12	1	31	83313.0995	0.0093	0.020	0.0094	0.50
247:	32	21	11	1	32	31	21	10	1	31	83332.3965	0.0288	0.020	0.0288	0.50
248:	32	21	12	1	32	31	21	11	1	31	83332.3965	0.0288	0.020	0.0288	0.50
249:	32	22	10	1	32	31	22	9	1	31	83352.5790	-0.0505	0.040	-0.0506	0.50
250:	32	22	11	1	32	31	22	10	1	31	83352.5790	-0.0505	0.040	-0.0506	0.50
251:	32	23	10	1	32	31	23	9	1	31	83373.8653	0.0005	0.020	0.0006	0.50
252:	32	23	9	1	32	31	23	8	1	31	83373.8653	0.0005	0.020	0.0006	0.50
253:	32	3	29	1	32	31	3	28	1	31	83393.2645	-0.0131	0.020		
254:	32	24	8	1	32	31	24	7	1	31	83396.0335	-0.0298	0.020	-0.0298	0.50
255:	32	24	9	1	32	31	24	8	1	31	83396.0335	-0.0298	0.020	-0.0298	0.50
256:	79	3	76	1	79	79	2	77	1	79	83426.6779	-0.0026	0.040		
257:	32	27	5	1	32	31	27	4	1	31	83468.3220	-0.0311	0.020	-0.0312	0.50
258:	32	27	6	1	32	31	27	5	1	31	83468.3220	-0.0311	0.020	-0.0312	0.50
259:	65	2	63	1	65	65	1	64	1	65	83531.0997	0.0289	0.040		
260:	32	1	31	1	32	31	1	30	1	31	83783.6262	-0.0127	0.020		
261:	32	2	30	1	32	31	2	29	1	31	83983.7207	0.0026	0.020		
262:	33	1	33	1	33	32	1	32	1	32	84248.5773	-0.0100	0.020		
263:	33	0	33	1	33	32	0	32	1	32	84523.0880	-0.0103	0.020		
264:	32	1	32	1	32	31	0	31	1	31	85275.5219	-0.0790	0.040		
265:	33	2	32	1	33	32	2	31	1	32	85452.6636	-0.0134	0.040		
266:	52	3	50	1	52	52	2	51	1	52	85588.6270	-0.0887	0.090		
267:	33	8	26	1	33	32	8	25	1	32	85770.6902	-0.0003	0.020	-0.0003	0.50
268:	33	8	25	1	33	32	8	24	1	32	85770.6902	-0.0003	0.020	-0.0003	0.50
269:	33	7	26	1	33	32	7	25	1	32	85771.2009	-0.0196	0.020	-0.0197	0.50
270:	33	7	27	1	33	32	7	26	1	32	85771.2009	-0.0196	0.020	-0.0197	0.50
271:	33	9	24	1	33	32	9	23	1	32	85773.6905	-0.0179	0.020	-0.0180	0.50
272:	33	9	25	1	33	32	9	24	1	32	85773.6905	-0.0179	0.020	-0.0180	0.50
273:	33	6	28	1	33	32	6	27	1	32	85777.2350	0.0104	0.020	0.0081	0.50
274:	33	6	27	1	33	32	6	26	1	32	85777.2350	0.0057	0.020	0.0081	0.50
275:	33	10	24	1	33	32	10	23	1	32	85779.2935	0.0094	0.020	0.0094	0.50
276:	33	10	23	1	33	32	10	22	1	32	85779.2935	0.0094	0.020	0.0094	0.50
277:	33	11	22	1	33	32	11	21	1	32	85786.8586	-0.0059	0.020	-0.0059	0.50
278:	33	11	23	1	33	32	11	22	1	32	85786.8586	-0.0059	0.020	-0.0059	0.50
279:	33	5	29	1	33	32	5	28	1	32	85792.7849	0.0039	0.020		
280:	33	5	28	1	33	32	5	27	1	32	85793.0886	0.0250	0.020		
281:	33	12	22	1	33	32	12	21	1	32	85796.1143	-0.0061	0.020	-0.0061	0.50
282:	33	12	21	1	33	32	12	20	1	32	85796.1143	-0.0061	0.020	-0.0061	0.50
283:	33	13	21	1	33	32	13	20	1	32	85806.8380	-0.0073	0.020	-0.0073	0.50
284:	33	13	20	1	33	32	13	19	1	32	85806.8380	-0.0073	0.020	-0.0073	0.50
285:	33	14	19	1	33	32	14	18	1	32	85818.9078	0.0037	0.020	0.0038	0.50
286:	33	14	20	1	33	32	14	19	1	32	85818.9078	0.0037	0.020	0.0038	0.50

287:	33	4	30	1	33	32	4	29	1	32	85824.0575	-0.0073	0.020		
288:	33	3	31	1	33	32	3	30	1	32	85826.3343	0.0116	0.020		
289:	33	15	18	1	33	32	15	17	1	32	85832.1976	-0.0070	0.020	-0.0070	0.50
290:	33	15	19	1	33	32	15	18	1	32	85832.1976	-0.0070	0.020	-0.0070	0.50
291:	33	4	29	1	33	32	4	28	1	32	85834.5255	0.0218	0.020		
292:	33	16	18	1	33	32	16	17	1	32	85846.7196	0.0369	0.020	0.0369	0.50
293:	33	16	17	1	33	32	16	16	1	32	85846.7196	0.0369	0.020	0.0369	0.50
294:	33	17	17	1	33	32	17	16	1	32	85862.2810	-0.0105	0.020	-0.0106	0.50
295:	33	17	16	1	33	32	17	15	1	32	85862.2810	-0.0105	0.020	-0.0106	0.50
296:	33	18	16	1	33	32	18	15	1	32	85879.0065	0.0098	0.020	0.0098	0.50
297:	33	18	15	1	33	32	18	14	1	32	85879.0065	0.0098	0.020	0.0098	0.50
298:	33	19	14	1	33	32	19	13	1	32	85896.7631	-0.0082	0.020	-0.0083	0.50
299:	33	19	15	1	33	32	19	14	1	32	85896.7631	-0.0082	0.020	-0.0083	0.50
300:	33	20	14	1	33	32	20	13	1	32	85915.5762	-0.0186	0.020	-0.0187	0.50
301:	33	20	13	1	33	32	20	12	1	32	85915.5762	-0.0186	0.020	-0.0187	0.50
302:	33	21	12	1	33	32	21	11	1	32	85935.4774	0.0270	0.020	0.0270	0.50
303:	33	21	13	1	33	32	21	12	1	32	85935.4774	0.0270	0.020	0.0270	0.50
304:	33	22	12	1	33	32	22	11	1	32	85956.3162	-0.0076	0.020	-0.0077	0.50
305:	33	22	11	1	33	32	22	10	1	32	85956.3162	-0.0076	0.020	-0.0077	0.50
306:	33	23	10	1	33	32	23	9	1	32	85978.2177	0.0142	0.020	0.0143	0.50
307:	33	23	11	1	33	32	23	10	1	32	85978.2177	0.0142	0.020	0.0143	0.50
308:	33	24	9	1	33	32	24	8	1	32	86001.1245	0.0460	0.040	0.0460	0.50
309:	33	24	10	1	33	32	24	9	1	32	86001.1245	0.0460	0.040	0.0460	0.50
310:	97	5	92	1	97	97	4	93	1	97	86019.1507	-0.0274	0.040		
311:	33	3	30	1	33	32	3	29	1	32	86021.2002	0.0230	0.020		
312:	33	25	9	1	33	32	25	8	1	32	86024.9299	-0.0096	0.020	-0.0096	0.50
313:	33	25	8	1	33	32	25	7	1	32	86024.9299	-0.0096	0.020	-0.0096	0.50
314:	33	26	7	1	33	32	26	6	1	32	86049.7960	0.0183	0.020	0.0183	0.50
315:	33	26	8	1	33	32	26	7	1	32	86049.7960	0.0183	0.020	0.0183	0.50
316:	33	28	5	1	33	32	28	4	1	32	86102.3235	-0.0283	0.020	-0.0284	0.50
317:	33	28	6	1	33	32	28	5	1	32	86102.3235	-0.0283	0.020	-0.0284	0.50
318:	33	29	4	1	33	32	29	3	1	32	86130.0930	0.0215	0.020	0.0215	0.50
319:	33	29	5	1	33	32	29	4	1	32	86130.0930	0.0215	0.020	0.0215	0.50
320:	33	30	3	1	33	32	30	2	1	32	86158.7295	-0.0057	0.020	-0.0057	0.50
321:	33	30	4	1	33	32	30	3	1	32	86158.7295	-0.0057	0.020	-0.0057	0.50
322:	96	5	91	1	96	96	4	92	1	96	86295.2708	-0.0319	0.040		
323:	33	1	32	1	33	32	1	31	1	32	86365.3453	0.0045	0.020		
324:	33	2	31	1	33	32	2	30	1	32	86630.9902	-0.0174	0.020		
325:	34	1	34	1	34	33	1	33	1	33	86786.1941	0.0117	0.020		
326:	35	0	35	1	35	34	1	34	1	34	86820.3093	0.0443	0.040		
327:	34	0	34	1	34	33	0	33	1	33	87042.7921	0.0008	0.020		
328:	33	1	33	1	33	32	0	32	1	32	87521.4625	0.0141	0.020		
329:	54	3	52	1	54	54	2	53	1	54	87758.1790	-0.0504	0.060		
330:	34	2	33	1	34	33	2	32	1	33	88024.8016	0.0018	0.020		
331:	81	3	78	1	81	81	2	79	1	81	88118.2490	-0.0026	0.040		
332:	92	5	87	1	92	92	4	88	1	92	88273.0196	-0.0394	0.060		
333:	34	8	26	1	34	33	8	25	1	33	88370.0945	0.0102	0.020	0.0103	0.50
334:	34	8	27	1	34	33	8	26	1	33	88370.0945	0.0103	0.020	0.0103	0.50
335:	34	7	27	1	34	33	7	26	1	33	88371.1764	0.0404	0.040	0.0405	0.50
336:	34	7	28	1	34	33	7	27	1	33	88371.1764	0.0405	0.040	0.0405	0.50
337:	34	9	25	1	34	33	9	24	1	33	88372.8685	0.0184	0.020	0.0184	0.50
338:	34	9	26	1	34	33	9	25	1	33	88372.8685	0.0184	0.020	0.0184	0.50
339:	34	6	29	1	34	33	6	28	1	33	88378.1100	-0.0022	0.020	-0.0054	0.50
340:	34	6	28	1	34	33	6	27	1	33	88378.1100	-0.0086	0.020	-0.0054	0.50
341:	34	10	25	1	34	33	10	24	1	33	88378.3788	0.0286	0.020	0.0286	0.50
342:	34	10	24	1	34	33	10	23	1	33	88378.3788	0.0286	0.020	0.0286	0.50
343:	34	11	23	1	34	33	11	22	1	33	88385.9846	0.0047	0.020	0.0048	0.50
344:	34	11	24	1	34	33	11	23	1	33	88385.9846	0.0047	0.020	0.0048	0.50
345:	34	5	30	1	34	33	5	29	1	33	88395.4180	-0.0357	0.020	0.0141	0.33
346:	34	12	23	1	34	33	12	22	1	33	88395.4180	0.0389	0.020	0.0141	0.33
347:	34	12	22	1	34	33	12	21	1	33	88395.4180	0.0389	0.020	0.0141	0.33
348:	34	5	29	1	34	33	5	28	1	33	88395.8117	-0.0116	0.020		
349:	34	13	22	1	34	33	13	21	1	33	88406.3364	0.0143	0.020	0.0144	0.50
350:	34	13	21	1	34	33	13	20	1	33	88406.3364	0.0143	0.020	0.0144	0.50
351:	34	14	21	1	34	33	14	20	1	33	88418.6559	-0.0051	0.020	-0.0051	0.50
352:	34	14	20	1	34	33	14	19	1	33	88418.6559	-0.0051	0.020	-0.0051	0.50
353:	34	3	32	1	34	33	3	31	1	33	88427.3867	-0.0135	0.020		
354:	34	4	31	1	34	33	4	30	1	33	88429.3900	0.0338	0.020		
355:	34	15	19	1	34	33	15	18	1	33	88432.2859	-0.0098	0.020	-0.0098	0.50
356:	34	15	20	1	34	33	15	19	1	33	88432.2859	-0.0098	0.020	-0.0098	0.50
357:	67	2	65	1	67	67	1	66	1	67	88433.9385	0.0580	0.040		
358:	34	4	30	1	34	33	4	29	1	33	88442.2171	0.0259	0.020		
359:	34	16	18	1	34	33	16	17	1	33	88447.1585	0.0027	0.020	0.0027	0.50
360:	34	16	19	1	34	33	16	18	1	33	88447.1585	0.0027	0.020	0.0027	0.50
361:	34	17	18	1	34	33	17	17	1	33	88463.1711	-0.0191	0.020	-0.0192	0.50
362:	34	17	17	1	34	33	17	16	1	33	88463.1711	-0.0191	0.020	-0.0192	0.50
363:	34	18	16	1	34	33	18	15	1	33	88480.3726	0.0111	0.020	0.0112	0.50
364:	34	18	17	1	34	33	18	16	1	33	88480.3726	0.0111	0.020	0.0112	0.50
365:	34	19	15	1	34	33	19	14	1	33	88498.6451	0.0046	0.020	0.0047	0.50
366:	34	19	16	1	34	33	19	15	1	33	88498.6451	0.0046	0.020	0.0047	0.50
367:	34	20	14	1	34	33	20	13	1	33	88518.0086	0.0039	0.020	0.0040	0.50
368:	34	20	15	1	34	33	20	14	1	33	88518.0086	0.0039	0.020	0.0040	0.50
369:	34	21	13	1	34	33	21	12	1	33	88538.4983	0.0624	0.040	0.0625	0.50

370:	34 21 14	1 34	33 21 13	1 33	88538.4983	0.0624	0.040	0.0625	0.50
371:	34 22 12	1 34	33 22 11	1 33	88559.9279	0.0089	0.020	0.0089	0.50
372:	34 22 13	1 34	33 22 12	1 33	88559.9279	0.0089	0.020	0.0089	0.50
373:	34 23 11	1 34	33 23 10	1 33	88582.4605	0.0193	0.020	0.0194	0.50
374:	34 23 12	1 34	33 23 11	1 33	88582.4605	0.0193	0.020	0.0194	0.50
375:	34 24 10	1 34	33 24 9	1 33	88606.0305	0.0394	0.040	0.0394	0.50
376:	34 24 11	1 34	33 24 10	1 33	88606.0305	0.0394	0.040	0.0394	0.50
377:	44 8 37	1 44	45 7 38	1 45	88613.0441	-0.0194	0.040	-0.0249	0.50
378:	44 8 36	1 44	45 7 39	1 45	88613.0441	-0.0304	0.040	-0.0249	0.50
379:	34 25 9	1 34	33 25 8	1 33	88630.5916	0.0329	0.020	0.0330	0.50
380:	34 25 10	1 34	33 25 9	1 33	88630.5916	0.0329	0.020	0.0330	0.50
381:	34 3 31	1 34	33 3 30	1 33	88651.5365	-0.0141	0.020		
382:	34 26 8	1 34	33 26 7	1 33	88656.1453	0.0109	0.020	0.0109	0.50
383:	34 26 9	1 34	33 26 8	1 33	88656.1453	0.0109	0.020	0.0109	0.50
384:	34 30 4	1 34	33 30 3	1 33	88768.3897	0.0456	0.040	0.0457	0.50
385:	34 30 5	1 34	33 30 4	1 33	88768.3897	0.0456	0.040	0.0457	0.50
386:	34 1 33	1 34	33 1 32	1 33	88943.2259	0.0152	0.020		
387:	34 2 32	1 34	33 2 31	1 33	89277.0441	0.0010	0.020		
388:	35 1 35	1 35	34 1 34	1 34	89322.9251	-0.0022	0.020		
389:	55 1 54	1 55	55 0 55	1 55	89496.4430	0.0234	0.020		
390:	35 0 35	1 35	34 0 34	1 34	89562.0076	0.0016	0.020		
391:	36 0 36	1 36	35 1 35	1 35	89578.2328	-0.0090	0.020		
392:	34 1 34	1 34	33 0 33	1 33	89784.5265	-0.0058	0.020		
393:	41 1 40	1 41	40 2 39	1 40	89907.2207	0.0123	0.040		
394:	56 3 54	1 56	56 2 55	1 56	90071.9595	0.0505	0.060		
395:	52 2 51	1 52	52 1 52	1 52	90095.7922	-0.0473	0.040	-0.0473	0.50
396:	52 2 51	1 52	52 1 52	1 52	90095.7922	-0.0473	0.040	-0.0473	0.50
397:	22 2 21	1 22	21 1 20	1 21	90409.1656	-0.0113	0.040		
398:	35 2 34	1 35	34 2 33	1 34	90595.5012	0.0148	0.020		
399:	62 3 59	1 62	61 4 58	1 61	90842.4700	0.0080	0.040		
400:	35 8 28	1 35	34 8 27	1 34	90969.5206	0.0181	0.020	0.0181	0.50
401:	35 8 27	1 35	34 8 26	1 34	90969.5206	0.0181	0.020	0.0181	0.50
402:	35 7 29	1 35	34 7 28	1 34	90971.1218	0.0008	0.020	0.0008	0.50
403:	35 7 28	1 35	34 7 27	1 34	90971.1218	0.0007	0.020	0.0008	0.50
404:	35 9 26	1 35	34 9 25	1 34	90971.9674	-0.0182	0.020	-0.0182	0.50
405:	35 9 27	1 35	34 9 26	1 34	90971.9674	-0.0182	0.020	-0.0182	0.50
406:	35 10 26	1 35	34 10 25	1 34	90977.3955	0.0071	0.020	0.0071	0.50
407:	35 10 25	1 35	34 10 24	1 34	90977.3955	0.0071	0.020	0.0071	0.50
408:	35 6 29	1 35	34 6 28	1 34	90979.1238	-0.0256	0.020	-0.0213	0.50
409:	35 6 30	1 35	34 6 29	1 34	90979.1238	-0.0168	0.020	-0.0213	0.50
410:	35 11 24	1 35	34 11 23	1 34	90985.0604	0.0091	0.020	0.0092	0.50
411:	35 11 25	1 35	34 11 24	1 34	90985.0604	0.0091	0.020	0.0092	0.50
412:	35 12 24	1 35	34 12 23	1 34	90994.5909	0.0093	0.020	0.0094	0.50
413:	35 12 23	1 35	34 12 22	1 34	90994.5909	0.0093	0.020	0.0094	0.50
414:	35 5 31	1 35	34 5 30	1 34	90998.4145	0.0348	0.020		
415:	35 5 30	1 35	34 5 29	1 34	90998.8178	-0.0415	0.040		
416:	35 13 23	1 35	34 13 22	1 34	91005.7361	0.0030	0.020	0.0030	0.50
417:	35 13 22	1 35	34 13 21	1 34	91005.7361	0.0030	0.020	0.0030	0.50
418:	35 14 21	1 35	34 14 20	1 34	91018.3498	0.0049	0.020	0.0049	0.50
419:	35 14 22	1 35	34 14 21	1 34	91018.3498	0.0049	0.020	0.0049	0.50
420:	35 3 33	1 35	34 3 32	1 34	91027.9809	-0.0046	0.020		
421:	35 15 20	1 35	34 15 19	1 34	91032.3016	-0.0059	0.020	-0.0060	0.50
422:	35 15 21	1 35	34 15 20	1 34	91032.3016	-0.0059	0.020	-0.0060	0.50
423:	35 4 32	1 35	34 4 31	1 34	91034.9319	-0.0120	0.020		
424:	35 16 19	1 35	34 16 18	1 34	91047.5440	-0.0005	0.020	-0.0005	0.50
425:	35 16 20	1 35	34 16 19	1 34	91047.5440	-0.0005	0.020	-0.0005	0.50
426:	35 4 31	1 35	34 4 30	1 34	91050.6333	0.0087	0.020		
427:	35 17 18	1 35	34 17 17	1 34	91063.9952	-0.0051	0.020	-0.0052	0.50
428:	35 17 19	1 35	34 17 18	1 34	91063.9952	-0.0051	0.020	-0.0052	0.50
429:	35 18 17	1 35	34 18 16	1 34	91081.6564	0.0223	0.020	0.0224	0.50
430:	35 18 18	1 35	34 18 17	1 34	91081.6564	0.0223	0.020	0.0224	0.50
431:	35 19 16	1 35	34 19 15	1 34	91100.3929	-0.0213	0.020	-0.0214	0.50
432:	35 19 17	1 35	34 19 16	1 34	91100.3929	-0.0213	0.020	-0.0214	0.50
433:	35 20 15	1 35	34 20 14	1 34	91120.3288	0.0122	0.020	0.0123	0.50
434:	35 20 16	1 35	34 20 15	1 34	91120.3288	0.0122	0.020	0.0123	0.50
435:	35 21 14	1 35	34 21 13	1 34	91141.3184	-0.0027	0.020	-0.0027	0.50
436:	35 21 15	1 35	34 21 14	1 34	91141.3184	-0.0027	0.020	-0.0027	0.50
437:	35 22 14	1 35	34 22 13	1 34	91163.4190	0.0071	0.020	0.0072	0.50
438:	35 22 13	1 35	34 22 12	1 34	91163.4190	0.0071	0.020	0.0072	0.50
439:	35 23 12	1 35	34 23 11	1 34	91186.5866	0.0117	0.020	0.0118	0.50
440:	35 23 13	1 35	34 23 12	1 34	91186.5866	0.0117	0.020	0.0118	0.50
441:	35 24 11	1 35	34 24 10	1 34	91210.8063	0.0082	0.020	0.0083	0.50
442:	35 24 12	1 35	34 24 11	1 34	91210.8063	0.0082	0.020	0.0083	0.50
443:	43 8 36	1 43	44 7 37	1 44	91222.8284	-0.0096	0.040	-0.0136	0.50
444:	43 8 35	1 43	44 7 38	1 44	91222.8284	-0.0176	0.040	-0.0136	0.50
445:	35 25 10	1 35	34 25 9	1 34	91236.0812	0.0106	0.020	0.0106	0.50
446:	35 25 11	1 35	34 25 10	1 34	91236.0812	0.0106	0.020	0.0106	0.50
447:	35 26 9	1 35	34 26 8	1 34	91262.3795	-0.0031	0.020	-0.0031	0.50
448:	35 26 10	1 35	34 26 9	1 34	91262.3795	-0.0031	0.020	-0.0031	0.50
449:	35 3 32	1 35	34 3 31	1 34	91284.4646	-0.0110	0.020		
450:	35 27 8	1 35	34 27 7	1 34	91289.7401	0.0155	0.020	0.0155	0.50
451:	35 27 9	1 35	34 27 8	1 34	91289.7401	0.0155	0.020	0.0155	0.50
452:	35 28 7	1 35	34 28 6	1 34	91318.0812	-0.0062	0.020	-0.0063	0.50

453:	35 28 8 1 35	34 28 7 1 34	91318.0812	-0.0062	0.020	-0.0063 0.50
454:	35 1 34 1 35	34 1 33 1 34	91517.1230	-0.0051	0.020	
455:	36 1 36 1 36	35 1 35 1 35	91858.8550	0.0030	0.020	
456:	35 2 33 1 35	34 2 32 1 34	91921.5635	0.0116	0.020	
457:	35 1 35 1 35	34 0 34 1 34	92064.6406	-0.0278	0.020	
458:	36 0 36 1 36	35 0 35 1 35	92080.9025	-0.0018	0.020	
459:	37 0 37 1 37	36 1 36 1 36	92319.0019	-0.0114	0.020	
460:	83 3 80 1 83	83 2 81 1 83	93009.4779	0.0515	0.060	
461:	36 2 35 1 36	35 2 34 1 35	93164.7231	0.0059	0.020	
462:	42 1 41 1 42	41 2 40 1 41	93337.0591	0.0285	0.040	
463:	36 8 29 1 36	35 8 28 1 35	93568.9543	0.0082	0.020	0.0083 0.50
464:	36 8 28 1 36	35 8 27 1 35	93568.9543	0.0082	0.020	0.0083 0.50
465:	36 9 28 1 36	35 9 27 1 35	93571.1410	0.0261	0.020	-0.0054 0.25
466:	36 9 27 1 36	35 9 26 1 35	93571.1410	0.0261	0.020	-0.0054 0.25
467:	36 7 30 1 36	35 7 29 1 35	93571.1410	-0.0368	0.020	-0.0054 0.25
468:	36 7 29 1 36	35 7 28 1 35	93571.1410	-0.0370	0.020	-0.0054 0.25
469:	36 10 27 1 36	35 10 26 1 35	93576.3797	-0.0182	0.020	-0.0182 0.50
470:	36 10 26 1 36	35 10 25 1 35	93576.3797	-0.0182	0.020	-0.0182 0.50
471:	36 6 30 1 36	35 6 29 1 35	93580.3299	0.0039	0.020	0.0100 0.50
472:	36 6 31 1 36	35 6 30 1 35	93580.3299	0.0160	0.020	0.0100 0.50
473:	36 11 26 1 36	35 11 25 1 35	93584.0802	0.0028	0.020	0.0028 0.50
474:	36 11 25 1 36	35 11 24 1 35	93584.0802	0.0028	0.020	0.0028 0.50
475:	36 12 25 1 36	35 12 24 1 35	93593.7340	0.0078	0.020	0.0078 0.50
476:	36 12 24 1 36	35 12 23 1 35	93593.7340	0.0078	0.020	0.0078 0.50
477:	54 2 53 1 54	54 1 54 1 54	93595.0629	0.0057	0.040	
478:	36 5 32 1 36	35 5 31 1 35	93601.6294	0.0646	0.040	
479:	36 5 31 1 36	35 5 30 1 35	93602.1449	-0.0375	0.020	
480:	36 13 23 1 36	35 13 22 1 35	93605.0879	0.0113	0.020	0.0114 0.50
481:	36 13 24 1 36	35 13 23 1 35	93605.0879	0.0113	0.020	0.0114 0.50
482:	36 14 22 1 36	35 14 21 1 35	93617.9429	-0.0104	0.020	-0.0104 0.50
483:	36 14 23 1 36	35 14 22 1 35	93617.9429	-0.0104	0.020	-0.0104 0.50
484:	36 3 34 1 36	35 3 33 1 35	93627.9927	-0.0192	0.020	
485:	36 15 21 1 36	35 15 20 1 35	93632.2260	-0.0116	0.020	-0.0117 0.50
486:	36 15 22 1 36	35 15 21 1 35	93632.2260	-0.0116	0.020	-0.0117 0.50
487:	36 4 33 1 36	35 4 32 1 35	93640.8243	0.0095	0.020	
488:	36 16 21 1 36	35 16 20 1 35	93647.8535	0.0071	0.020	0.0072 0.50
489:	36 16 20 1 36	35 16 19 1 35	93647.8535	0.0071	0.020	0.0072 0.50
490:	36 4 32 1 36	35 4 31 1 35	93659.8748	0.0181	0.020	
491:	36 17 20 1 36	35 17 19 1 35	93664.7206	0.0013	0.020	0.0014 0.50
492:	36 17 19 1 36	35 17 18 1 35	93664.7206	0.0013	0.020	0.0014 0.50
493:	36 18 19 1 36	35 18 18 1 35	93682.7989	-0.0128	0.020	-0.0129 0.50
494:	36 18 18 1 36	35 18 17 1 35	93682.7989	-0.0128	0.020	-0.0129 0.50
495:	36 19 17 1 36	35 19 16 1 35	93702.0982	0.0081	0.020	0.0081 0.50
496:	36 19 18 1 36	35 19 17 1 35	93702.0982	0.0081	0.020	0.0081 0.50
497:	36 20 17 1 36	35 20 16 1 35	93722.5458	0.0181	0.020	0.0181 0.50
498:	36 20 16 1 36	35 20 15 1 35	93722.5458	0.0181	0.020	0.0181 0.50
499:	36 21 16 1 36	35 21 15 1 35	93744.1229	0.0195	0.020	0.0196 0.50
500:	36 21 15 1 36	35 21 14 1 35	93744.1229	0.0195	0.020	0.0196 0.50
501:	36 22 14 1 36	35 22 13 1 35	93766.7828	-0.0167	0.020	-0.0167 0.50
502:	36 22 15 1 36	35 22 14 1 35	93766.7828	-0.0167	0.020	-0.0167 0.50
503:	36 23 14 1 36	35 23 13 1 35	93790.5887	-0.0127	0.020	-0.0127 0.50
504:	36 23 13 1 36	35 23 12 1 35	93790.5887	-0.0127	0.020	-0.0127 0.50
505:	36 24 12 1 36	35 24 11 1 35	93815.5205	0.0243	0.020	0.0243 0.50
506:	36 24 13 1 36	35 24 12 1 35	93815.5205	0.0243	0.020	0.0243 0.50
507:	36 25 12 1 36	35 25 11 1 35	93841.4560	-0.0163	0.020	-0.0163 0.50
508:	36 25 11 1 36	35 25 10 1 35	93841.4560	-0.0163	0.020	-0.0163 0.50
509:	36 26 10 1 36	35 26 9 1 35	93868.4640	-0.0551	0.040	-0.0552 0.50
510:	36 26 11 1 36	35 26 10 1 35	93868.4640	-0.0551	0.040	-0.0552 0.50
511:	36 27 9 1 36	35 27 8 1 35	93896.6581	0.0312	0.020	0.0312 0.50
512:	36 27 10 1 36	35 27 9 1 35	93896.6581	0.0312	0.020	0.0312 0.50
513:	36 3 33 1 36	35 3 32 1 35	93920.0064	-0.0051	0.020	
514:	36 28 8 1 36	35 28 7 1 35	93925.7874	0.0016	0.020	0.0016 0.50
515:	36 28 9 1 36	35 28 8 1 35	93925.7874	0.0016	0.020	0.0016 0.50
516:	36 29 8 1 36	35 29 7 1 35	93955.9680	-0.0185	0.020	-0.0185 0.50
517:	36 29 7 1 36	35 29 6 1 35	93955.9680	-0.0185	0.020	-0.0185 0.50
518:	36 1 35 1 36	35 1 34 1 35	94087.0188	0.0279	0.020	
519:	36 1 36 1 36	35 0 35 1 35	94361.5322	0.0177	0.020	
520:	37 1 37 1 37	36 1 36 1 36	94393.9776	-0.0089	0.020	
521:	36 2 34 1 36	35 2 33 1 35	94564.2816	-0.0015	0.020	
522:	37 0 37 1 37	36 0 36 1 36	94599.6179	-0.0056	0.020	
523:	38 0 38 1 38	37 1 37 1 37	95043.3151	0.0107	0.020	
524:	60 3 58 1 60	60 2 59 1 60	95117.2477	0.0552	0.060	
525:	84 5 79 1 84	84 4 80 1 84	95386.5691	-0.0048	0.040	
526:	37 2 36 1 37	36 2 35 1 36	95732.4647	-0.0106	0.020	
527:	33 4 30 1 33	33 3 31 1 33	95860.2595	-0.0585	0.090	
528:	34 4 31 1 34	34 3 32 1 34	95862.2437	-0.0303	0.060	
529:	32 4 29 1 32	32 3 30 1 32	95862.5177	-0.0582	0.090	
530:	31 4 28 1 31	31 3 29 1 31	95868.4128	0.0983	0.060	
531:	35 4 32 1 35	35 3 33 1 35	95869.2606	0.0282	0.040	
532:	36 4 33 1 36	36 3 34 1 36	95882.0193	-0.0159	0.040	
533:	29 4 26 1 29	29 3 27 1 29	95887.5622	-0.0224	0.060	
534:	28 4 25 1 28	28 3 26 1 28	95899.9077	-0.0278	0.060	
535:	37 4 34 1 37	37 3 35 1 37	95901.5785	0.0024	0.040	

536:	21	4	17	1	21	21	3	18	1	21	95911.7065	0.0798	0.060		
537:	27	4	24	1	27	27	3	25	1	27	95913.3836	-0.0220	0.060		
538:	26	4	23	1	26	26	3	24	1	26	95927.5128	-0.0350	0.060		
539:	38	4	35	1	38	38	3	36	1	38	95928.8187	0.0216	0.040		
540:	25	4	22	1	25	25	3	23	1	25	95941.9488	-0.0217	0.060		
541:	20	4	16	1	20	20	3	17	1	20	95944.5197	0.0993	0.060		
542:	24	4	21	1	24	24	3	22	1	24	95956.4176	0.0816	0.060		
543:	39	4	36	1	39	39	3	37	1	39	95964.6750	-0.0111	0.040		
544:	23	4	20	1	23	23	3	21	1	23	95970.3710	0.0120	0.040		
545:	19	4	15	1	19	19	3	16	1	19	95971.6839	-0.0130	0.040		
546:	40	4	37	1	40	40	3	38	1	40	96010.2350	-0.0383	0.060		
547:	19	4	16	1	19	19	3	17	1	19	96019.0997	0.0981	0.060		
548:	18	4	15	1	18	18	3	16	1	18	96028.6618	-0.0114	0.040		
549:	17	4	14	1	17	17	3	15	1	17	96037.2316	-0.0003	0.040		
550:	16	4	13	1	16	16	3	14	1	16	96044.6814	0.0059	0.040		
551:	42	4	39	1	42	42	3	40	1	42	96134.8242	-0.0257	0.060		
552:	37	8	30	1	37	36	8	29	1	36	96168.4261	0.0106	0.020	0.0107	0.50
553:	37	8	29	1	37	36	8	28	1	36	96168.4261	0.0106	0.020	0.0107	0.50
554:	37	9	28	1	37	36	9	27	1	36	96170.2441	0.0065	0.020	0.0066	0.50
555:	37	9	29	1	37	36	9	28	1	36	96170.2441	0.0065	0.020	0.0066	0.50
556:	37	7	31	1	37	36	7	30	1	36	96171.3128	0.0041	0.020	0.0041	0.50
557:	37	7	30	1	37	36	7	29	1	36	96171.3128	0.0039	0.020	0.0041	0.50
558:	37	10	28	1	37	36	10	27	1	36	96175.3667	-0.0111	0.020	-0.0111	0.50
559:	37	10	27	1	37	36	10	26	1	36	96175.3667	-0.0111	0.020	-0.0111	0.50
560:	37	6	31	1	37	36	6	30	1	36	96181.6418	-0.0105	0.020	-0.0024	0.50
561:	37	6	32	1	37	36	6	31	1	36	96181.6418	0.0057	0.020	-0.0024	0.50
562:	37	11	26	1	37	36	11	25	1	36	96183.0697	0.0127	0.020	0.0127	0.50
563:	37	11	27	1	37	36	11	26	1	36	96183.0697	0.0127	0.020	0.0127	0.50
564:	37	12	26	1	37	36	12	25	1	36	96192.8197	0.0083	0.020	0.0083	0.50
565:	37	12	25	1	37	36	12	24	1	36	96192.8197	0.0083	0.020	0.0083	0.50
566:	37	13	25	1	37	36	13	24	1	36	96204.3679	0.0174	0.020	0.0175	0.50
567:	37	13	24	1	37	36	13	23	1	36	96204.3679	0.0174	0.020	0.0175	0.50
568:	37	5	33	1	37	36	5	32	1	36	96205.0307	0.0163	0.020		
569:	37	5	32	1	37	36	5	31	1	36	96205.7921	-0.0120	0.020		
570:	43	4	40	1	43	43	3	41	1	43	96216.0715	-0.0025	0.040		
571:	37	14	23	1	37	36	14	22	1	36	96217.4890	0.0047	0.020	0.0048	0.50
572:	37	14	24	1	37	36	14	23	1	36	96217.4890	0.0047	0.020	0.0048	0.50
573:	37	3	35	1	37	36	3	34	1	36	96227.3972	-0.0151	0.020		
574:	37	15	23	1	37	36	15	22	1	36	96232.0852	0.0014	0.020	0.0014	0.50
575:	37	15	22	1	37	36	15	21	1	36	96232.0852	0.0014	0.020	0.0014	0.50
576:	37	4	34	1	37	36	4	33	1	36	96246.9436	-0.0095	0.020		
577:	37	16	22	1	37	36	16	21	1	36	96248.0556	-0.0032	0.020	-0.0032	0.50
578:	37	16	21	1	37	36	16	20	1	36	96248.0556	-0.0032	0.020	-0.0032	0.50
579:	37	17	20	1	37	36	17	19	1	36	96265.3451	0.0008	0.020	0.0008	0.50
580:	37	17	21	1	37	36	17	20	1	36	96265.3451	0.0008	0.020	0.0008	0.50
581:	37	4	33	1	37	36	4	32	1	36	96269.9496	0.0054	0.020		
582:	37	18	20	1	37	36	18	19	1	36	96283.8880	-0.0039	0.020	-0.0039	0.50
583:	37	18	19	1	37	36	18	18	1	36	96283.8880	-0.0039	0.020	-0.0039	0.50
584:	37	19	19	1	37	36	19	18	1	36	96303.6700	0.0048	0.020	0.0049	0.50
585:	37	19	18	1	37	36	19	17	1	36	96303.6700	0.0048	0.020	0.0049	0.50
586:	37	21	16	1	37	36	21	15	1	36	96346.7703	-0.0091	0.020	-0.0092	0.50
587:	37	21	17	1	37	36	21	16	1	36	96346.7703	-0.0091	0.020	-0.0092	0.50
588:	37	22	15	1	37	36	22	14	1	36	96370.0931	0.0141	0.020	0.0141	0.50
589:	37	22	16	1	37	36	22	15	1	36	96370.0931	0.0141	0.020	0.0141	0.50
590:	37	23	14	1	37	36	23	13	1	36	96394.4843	-0.0336	0.020	-0.0336	0.50
591:	37	23	15	1	37	36	23	14	1	36	96394.4843	-0.0336	0.020	-0.0336	0.50
592:	37	24	13	1	37	36	24	12	1	36	96420.0749	-0.0076	0.020	-0.0076	0.50
593:	37	24	14	1	37	36	24	13	1	36	96420.0749	-0.0076	0.020	-0.0076	0.50
594:	41	8	34	1	41	42	7	35	1	42	96439.1100	0.0399	0.060	0.0378	0.50
595:	41	8	33	1	41	42	7	36	1	42	96439.1100	0.0357	0.060	0.0378	0.50
596:	37	25	12	1	37	36	25	11	1	36	96446.7351	-0.0255	0.020	-0.0255	0.50
597:	37	25	13	1	37	36	25	12	1	36	96446.7351	-0.0255	0.020	-0.0255	0.50
598:	61	3	59	1	61	61	2	60	1	61	96462.1675	0.0017	0.040		
599:	46	4	43	1	46	46	3	44	1	46	96549.4540	0.0304	0.040		
600:	37	3	34	1	37	36	3	33	1	36	96558.1743	-0.0214	0.020		
601:	37	30	7	1	37	36	30	6	1	36	96596.4378	-0.0421	0.040	-0.0422	0.50
602:	37	30	8	1	37	36	30	7	1	36	96596.4378	-0.0421	0.040	-0.0422	0.50
603:	37	31	6	1	37	36	31	5	1	36	96629.5829	-0.0369	0.020	-0.0369	0.50
604:	37	31	7	1	37	36	31	6	1	36	96629.5829	-0.0369	0.020	-0.0369	0.50
605:	37	1	36	1	37	36	1	35	1	36	96652.7186	0.0012	0.020		
606:	37	1	37	1	37	36	0	36	1	36	96674.6439	0.0471	0.040		
607:	47	4	44	1	47	47	3	45	1	47	96694.4056	0.0032	0.040		
608:	48	4	45	1	48	48	3	46	1	48	96858.2906	-0.0289	0.060		
609:	38	1	38	1	38	37	1	37	1	37	96928.3576	-0.0045	0.020		
610:	49	4	46	1	49	49	3	47	1	49	97042.3833	0.0018	0.040		
611:	38	0	38	1	38	37	0	37	1	37	97118.2396	-0.0379	0.020		
612:	82	5	77	1	82	82	4	78	1	82	97554.9433	0.0257	0.040		
613:	30	7	24	1	30	31	6	25	1	31	97689.4217	0.0402	0.060	0.0366	0.50
614:	30	7	23	1	30	31	6	26	1	31	97689.4217	0.0330	0.060	0.0366	0.50
615:	52	4	49	1	52	52	3	50	1	52	97727.3242	-0.0576	0.060		
616:	26	2	25	1	26	25	1	24	1	25	97732.1073	0.0317	0.040		
617:	53	4	50	1	53	53	3	51	1	53	98003.9512	0.0586	0.040		
618:	38	2	37	1	38	37	2	36	1	37	98298.7449	-0.0018	0.020		

619:	54	4	51	1	54	54	3	52	1	54	98306.4312	0.0420	0.040		
620:	38	8	31	1	38	37	8	30	1	37	98767.9064	-0.0049	0.020	-0.0049	0.50
621:	38	8	30	1	38	37	8	29	1	37	98767.9064	-0.0049	0.020	-0.0049	0.50
622:	38	9	30	1	38	37	9	29	1	37	98769.3554	0.0020	0.020	0.0020	0.50
623:	38	9	29	1	38	37	9	28	1	37	98769.3554	0.0020	0.020	0.0020	0.50
624:	38	7	31	1	38	37	7	30	1	37	98771.5105	-0.0049	0.020	-0.0048	0.50
625:	38	7	32	1	38	37	7	31	1	37	98771.5105	-0.0046	0.020	-0.0048	0.50
626:	38	10	29	1	38	37	10	28	1	37	98774.3123	-0.0149	0.020	-0.0149	0.50
627:	38	10	28	1	38	37	10	27	1	37	98774.3123	-0.0149	0.020	-0.0149	0.50
628:	38	11	28	1	38	37	11	27	1	37	98781.9832	-0.0054	0.020	-0.0054	0.50
629:	38	11	27	1	38	37	11	26	1	37	98781.9832	-0.0054	0.020	-0.0054	0.50
630:	38	6	33	1	38	37	6	32	1	37	98783.1343	0.0230	0.020	0.0121	0.50
631:	38	6	32	1	38	37	6	31	1	37	98783.1343	0.0011	0.020	0.0121	0.50
632:	38	12	27	1	38	37	12	26	1	37	98791.8339	-0.0014	0.020	-0.0014	0.50
633:	38	12	26	1	38	37	12	25	1	37	98791.8339	-0.0014	0.020	-0.0014	0.50
634:	38	13	25	1	38	37	13	24	1	37	98803.5498	-0.0030	0.020	-0.0031	0.50
635:	38	13	26	1	38	37	13	25	1	37	98803.5498	-0.0030	0.020	-0.0031	0.50
636:	38	5	34	1	38	37	5	33	1	37	98808.7692	0.0353	0.020		
637:	38	5	33	1	38	37	5	32	1	37	98809.7378	0.0010	0.020		
638:	38	14	24	1	38	37	14	23	1	37	98816.9368	0.0013	0.020	0.0014	0.50
639:	38	14	25	1	38	37	14	24	1	37	98816.9368	0.0013	0.020	0.0014	0.50
640:	38	3	36	1	38	37	3	35	1	37	98826.1171	-0.0021	0.020		
641:	38	15	23	1	38	37	15	22	1	37	98831.8496	0.0061	0.020	0.0061	0.50
642:	38	15	24	1	38	37	15	23	1	37	98831.8496	0.0061	0.020	0.0061	0.50
643:	38	16	23	1	38	37	16	22	1	37	98848.1682	-0.0112	0.020	-0.0113	0.50
644:	38	16	22	1	38	37	16	21	1	37	98848.1682	-0.0112	0.020	-0.0113	0.50
645:	38	4	35	1	38	37	4	34	1	37	98853.3385	-0.0018	0.020		
646:	38	17	22	1	38	37	17	21	1	37	98865.8577	-0.0152	0.020	-0.0152	0.50
647:	38	17	21	1	38	37	17	20	1	37	98865.8577	-0.0152	0.020	-0.0152	0.50
648:	38	4	34	1	38	37	4	33	1	37	98880.9331	-0.0148	0.020		
649:	38	18	21	1	38	37	18	20	1	37	98884.8255	-0.0462	0.040	-0.0463	0.50
650:	38	18	20	1	38	37	18	19	1	37	98884.8255	-0.0462	0.040	-0.0463	0.50
651:	38	19	19	1	38	37	19	18	1	37	98905.1137	-0.0228	0.020	-0.0228	0.50
652:	38	19	20	1	38	37	19	19	1	37	98905.1137	-0.0228	0.020	-0.0228	0.50
653:	19	6	14	1	19	20	5	15	1	20	98915.0637	0.1025	0.040	0.0987	0.50
654:	19	6	13	1	19	20	5	16	1	20	98915.0637	0.0949	0.040	0.0987	0.50
655:	38	20	18	1	38	37	20	17	1	37	98926.6041	-0.0322	0.020	-0.0322	0.50
656:	38	20	19	1	38	37	20	18	1	37	98926.6041	-0.0322	0.020	-0.0322	0.50
657:	38	21	17	1	38	37	21	16	1	37	98949.3362	-0.0104	0.020	-0.0104	0.50
658:	38	21	18	1	38	37	21	17	1	37	98949.3362	-0.0104	0.020	-0.0104	0.50
659:	38	22	17	1	38	37	22	16	1	37	98973.2355	-0.0117	0.020	-0.0117	0.50
660:	38	22	16	1	38	37	22	15	1	37	98973.2355	-0.0117	0.020	-0.0117	0.50
661:	56	4	53	1	56	56	3	54	1	56	98993.6420	-0.0415	0.060		
662:	38	23	16	1	38	37	23	15	1	37	98998.3173	-0.0039	0.020	-0.0039	0.50
663:	38	23	15	1	38	37	23	14	1	37	98998.3173	-0.0039	0.020	-0.0039	0.50
664:	38	1	38	1	38	37	0	37	1	37	99003.3617	0.0263	0.020		
665:	38	24	14	1	38	37	24	13	1	37	99024.5464	-0.0075	0.020	-0.0076	0.50
666:	38	24	15	1	38	37	24	14	1	37	99024.5464	-0.0075	0.020	-0.0076	0.50
667:	38	25	13	1	38	37	25	12	1	37	99051.9130	-0.0193	0.020	-0.0194	0.50
668:	38	25	14	1	38	37	25	13	1	37	99051.9130	-0.0193	0.020	-0.0194	0.50
669:	38	26	13	1	38	37	26	12	1	37	99080.4580	0.0133	0.020	0.0133	0.50
670:	38	26	12	1	38	37	26	11	1	37	99080.4580	0.0133	0.020	0.0133	0.50
671:	38	29	9	1	38	37	29	8	1	37	99172.6493	-0.0271	0.020	-0.0272	0.50
672:	38	29	10	1	38	37	29	9	1	37	99172.6493	-0.0271	0.020	-0.0272	0.50
673:	38	3	35	1	38	37	3	34	1	37	99199.0521	0.0117	0.020		
674:	38	1	37	1	38	37	1	36	1	37	99214.2585	0.0073	0.020		
675:	39	1	39	1	39	38	1	38	1	38	99461.9980	-0.0118	0.020		
676:	39	0	39	1	39	38	0	38	1	38	99636.9604	0.0019	0.020		
677:	58	4	55	1	58	58	3	56	1	58	99797.5646	-0.0063	0.040		
678:	38	2	36	1	38	37	2	35	1	37	99843.5107	0.0002	0.020		
679:	44	1	43	1	44	43	2	42	1	43	100135.9191	-0.0524	0.060		
680:	59	4	56	1	59	59	3	57	1	59	100245.6338	-0.0105	0.040		
681:	29	7	23	1	29	30	6	24	1	30	100293.4137	0.0484	0.040	0.0461	0.50
682:	29	7	22	1	29	30	6	25	1	30	100293.4137	0.0436	0.040	0.0461	0.50
683:	40	0	40	1	40	39	1	39	1	39	100445.6646	0.0351	0.020		
684:	39	2	38	1	39	38	2	37	1	38	100863.5170	-0.0033	0.020		
685:	61	4	58	1	61	61	3	59	1	61	101238.4698	0.0410	0.040		
686:	28	2	27	1	28	27	1	26	1	27	101250.8838	0.0094	0.040		
687:	39	1	39	1	39	38	0	38	1	38	101347.0620	-0.0055	0.020		
688:	39	8	32	1	39	38	8	31	1	38	101367.4200	-0.0143	0.020	-0.0144	0.50
689:	39	8	31	1	39	38	8	30	1	38	101367.4200	-0.0143	0.020	-0.0144	0.50
690:	39	9	31	1	39	38	9	30	1	38	101368.4590	-0.0031	0.020	-0.0032	0.50
691:	39	9	30	1	39	38	9	29	1	38	101368.4590	-0.0031	0.020	-0.0032	0.50
692:	39	7	33	1	39	38	7	32	1	38	101371.8170	0.0175	0.020	0.0174	0.50
693:	39	7	32	1	39	38	7	31	1	38	101371.8170	0.0171	0.020	0.0174	0.50
694:	39	10	29	1	39	38	10	28	1	38	101373.2394	-0.0058	0.020	-0.0059	0.50
695:	39	10	30	1	39	38	10	29	1	38	101373.2394	-0.0058	0.020	-0.0059	0.50
696:	39	11	28	1	39	38	11	27	1	38	101380.8534	-0.0175	0.020	-0.0175	0.50
697:	39	11	29	1	39	38	11	28	1	38	101380.8534	-0.0175	0.020	-0.0175	0.50
698:	39	6	34	1	39	38	6	33	1	38	101384.7476	0.0040	0.020	-0.0106	0.50
699:	39	6	33	1	39	38	6	32	1	38	101384.7476	-0.0252	0.020	-0.0106	0.50
700:	39	12	27	1	39	38	12	26	1	38	101390.7733	-0.0231	0.020	-0.0232	0.50
701:	39	12	28	1	39	38	12	27	1	38	101390.7733	-0.0231	0.020	-0.0232	0.50

702:	39 13 26 1 39	38 13 25 1 38	101402.6845	0.0027	0.020	0.0027	0.50
703:	39 13 27 1 39	38 13 26 1 38	101402.6845	0.0027	0.020	0.0027	0.50
704:	39 5 35 1 39	38 5 34 1 38	101412.7558	0.0279	0.020		
705:	39 5 34 1 39	38 5 33 1 38	101413.9775	-0.0157	0.020		
706:	39 14 25 1 39	38 14 24 1 38	101416.2949	-0.0098	0.020	-0.0098	0.50
707:	39 14 26 1 39	38 14 25 1 38	101416.2949	-0.0098	0.020	-0.0098	0.50
708:	39 3 37 1 39	38 3 36 1 38	101424.0631	-0.0027	0.020		
709:	39 15 24 1 39	38 15 23 1 38	101431.5013	-0.0132	0.020	-0.0132	0.50
710:	39 15 25 1 39	38 15 24 1 38	101431.5013	-0.0132	0.020	-0.0132	0.50
711:	39 16 23 1 39	38 16 22 1 38	101448.2177	0.0119	0.020	0.0120	0.50
712:	39 16 24 1 39	38 16 23 1 38	101448.2177	0.0119	0.020	0.0120	0.50
713:	39 4 36 1 39	38 4 35 1 38	101459.9573	0.0024	0.020		
714:	39 17 22 1 39	38 17 21 1 38	101466.2958	-0.0067	0.020	-0.0067	0.50
715:	39 17 23 1 39	38 17 22 1 38	101466.2958	-0.0067	0.020	-0.0067	0.50
716:	39 18 21 1 39	38 18 20 1 38	101485.7419	-0.0067	0.020	-0.0067	0.50
717:	39 18 22 1 39	38 18 21 1 38	101485.7419	-0.0067	0.020	-0.0067	0.50
718:	39 4 35 1 39	38 4 34 1 38	101492.9352	0.0016	0.020		
719:	39 19 20 1 39	38 19 19 1 38	101506.4895	-0.0120	0.020	-0.0120	0.50
720:	39 19 21 1 39	38 19 20 1 38	101506.4895	-0.0120	0.020	-0.0120	0.50
721:	18 6 13 1 18	19 5 14 1 19	101515.8001	0.1041	0.040	0.1019	0.50
722:	18 6 12 1 18	19 5 15 1 19	101515.8001	0.0996	0.040	0.1019	0.50
723:	39 20 19 1 39	38 20 18 1 38	101528.5550	0.0269	0.020	0.0270	0.50
724:	39 20 20 1 39	38 20 19 1 38	101528.5550	0.0269	0.020	0.0270	0.50
725:	39 21 18 1 39	38 21 17 1 38	101551.7896	-0.0122	0.020	-0.0122	0.50
726:	39 21 19 1 39	38 21 18 1 38	101551.7896	-0.0122	0.020	-0.0122	0.50
727:	39 23 16 1 39	38 23 15 1 38	101602.0350	0.0266	0.020	0.0267	0.50
728:	39 23 17 1 39	38 23 16 1 38	101602.0350	0.0266	0.020	0.0267	0.50
729:	39 24 15 1 39	38 24 14 1 38	101628.8892	-0.0181	0.020	-0.0181	0.50
730:	39 24 16 1 39	38 24 15 1 38	101628.8892	-0.0181	0.020	-0.0181	0.50
731:	39 25 14 1 39	38 25 13 1 38	101656.9747	-0.0097	0.020	-0.0098	0.50
732:	39 25 15 1 39	38 25 14 1 38	101656.9747	-0.0097	0.020	-0.0098	0.50
733:	39 26 13 1 39	38 26 12 1 38	101686.2358	0.0085	0.020	0.0085	0.50
734:	39 26 14 1 39	38 26 13 1 38	101686.2358	0.0085	0.020	0.0085	0.50
735:	39 27 12 1 39	38 27 11 1 38	101716.6133	-0.0107	0.020	-0.0108	0.50
736:	39 27 13 1 39	38 27 12 1 38	101716.6133	-0.0107	0.020	-0.0108	0.50
737:	39 28 11 1 39	38 28 10 1 38	101748.1917	0.0279	0.020	0.0279	0.50
738:	39 28 12 1 39	38 28 11 1 38	101748.1917	0.0279	0.020	0.0279	0.50
739:	39 1 38 1 39	38 1 37 1 38	101771.5562	-0.0074	0.020		
740:	39 3 36 1 39	38 3 35 1 38	101842.5283	-0.0006	0.020		
741:	39 31 8 1 39	38 31 7 1 38	101849.5367	0.0037	0.020	0.0038	0.50
742:	39 31 9 1 39	38 31 8 1 38	101849.5367	0.0037	0.020	0.0038	0.50
743:	39 34 5 1 39	38 34 4 1 38	101960.8367	0.0370	0.020	0.0371	0.50
744:	39 34 6 1 39	38 34 5 1 38	101960.8367	0.0370	0.020	0.0371	0.50
745:	40 1 40 1 40	39 1 39 1 39	101994.9581	-0.0028	0.020		
746:	40 0 40 1 40	39 0 39 1 39	102155.7631	0.0245	0.020		
747:	39 2 37 1 39	38 2 36 1 38	102479.5973	-0.0040	0.020		
748:	41 0 41 1 41	40 1 40 1 40	103125.4082	0.0670	0.040		
749:	40 8 32 1 40	39 8 31 1 39	103966.9730	-0.0122	0.020	-0.0122	0.50
750:	40 8 33 1 40	39 8 32 1 39	103966.9730	-0.0122	0.020	-0.0122	0.50
751:	40 9 31 1 40	39 9 30 1 39	103967.5521	-0.0115	0.020	-0.0115	0.50
752:	40 9 32 1 40	39 9 31 1 39	103967.5521	-0.0115	0.020	-0.0115	0.50
753:	40 10 31 1 40	39 10 30 1 39	103972.1602	0.0291	0.020	0.0128	0.25
754:	40 10 30 1 40	39 10 29 1 39	103972.1602	0.0291	0.020	0.0128	0.25
755:	40 7 34 1 40	39 7 33 1 39	103972.1602	-0.0032	0.020	0.0128	0.25
756:	40 7 33 1 40	39 7 32 1 39	103972.1602	-0.0038	0.020	0.0128	0.25
757:	40 11 29 1 40	39 11 28 1 39	103979.6788	-0.0238	0.020	-0.0239	0.50
758:	40 11 30 1 40	39 11 29 1 39	103979.6788	-0.0238	0.020	-0.0239	0.50
759:	40 6 35 1 40	39 6 34 1 39	103986.5662	0.0289	0.020	0.0097	0.50
760:	40 6 34 1 40	39 6 33 1 39	103986.5662	-0.0096	0.020	0.0097	0.50
761:	40 12 28 1 40	39 12 27 1 39	103989.7344	0.0414	0.040	0.0414	0.50
762:	40 12 29 1 40	39 12 28 1 39	103989.7344	0.0414	0.040	0.0414	0.50
763:	40 13 27 1 40	39 13 26 1 39	104001.7507	0.0154	0.020	0.0154	0.50
764:	40 13 28 1 40	39 13 27 1 39	104001.7507	0.0154	0.020	0.0154	0.50
765:	40 14 27 1 40	39 14 26 1 39	104015.5403	-0.0496	0.040	-0.0496	0.50
766:	40 14 26 1 40	39 14 25 1 39	104015.5403	-0.0496	0.040	-0.0496	0.50
767:	40 5 36 1 40	39 5 35 1 39	104017.0208	0.0200	0.020		
768:	40 5 35 1 40	39 5 34 1 39	104018.6030	0.0155	0.020		
769:	40 3 38 1 40	39 3 37 1 39	104021.2317	0.0464	0.040		
770:	40 15 25 1 40	39 15 24 1 39	104031.1898	0.0953	0.060	0.0954	0.50
771:	40 15 26 1 40	39 15 25 1 39	104031.1898	0.0953	0.060	0.0954	0.50
772:	40 16 25 1 40	39 16 24 1 39	104048.1387	0.0035	0.020	0.0035	0.50
773:	40 16 24 1 40	39 16 23 1 39	104048.1387	0.0035	0.020	0.0035	0.50
774:	40 17 23 1 40	39 17 22 1 39	104066.6914	0.0609	0.020	0.0136	0.33
775:	40 17 24 1 40	39 17 23 1 39	104066.6914	0.0609	0.020	0.0136	0.33
776:	40 4 37 1 40	39 4 36 1 39	104066.6914	-0.0810	0.020	0.0136	0.33
777:	40 18 23 1 40	39 18 22 1 39	104086.5188	-0.0009	0.020	-0.0009	0.50
778:	40 18 22 1 40	39 18 21 1 39	104086.5188	-0.0009	0.020	-0.0009	0.50
779:	40 4 36 1 40	39 4 35 1 39	104105.9319	-0.0388	0.040		
780:	40 19 21 1 40	39 19 20 1 39	104107.7403	-0.0170	0.020	-0.0170	0.50
781:	40 19 22 1 40	39 19 21 1 39	104107.7403	-0.0170	0.020	-0.0170	0.50
782:	40 20 21 1 40	39 20 20 1 39	104130.3538	0.0462	0.040	0.0463	0.50
783:	40 20 20 1 40	39 20 19 1 39	104130.3538	0.0462	0.040	0.0463	0.50
784:	40 21 19 1 40	39 21 18 1 39	104154.2157	0.0735	0.040	0.0735	0.50

785:	40	21	20	1	40	39	21	19	1	39	104154.2157	0.0735	0.040	0.0735	0.50
786:	40	22	18	1	40	39	22	17	1	39	104179.2930	0.0548	0.040		
787:	40	22	19	1	40	39	22	18	1	39	104179.3019	0.0637	0.040		
788:	40	23	18	1	40	39	23	17	1	39	104205.5871	0.0109	0.020	0.0109	0.50
789:	40	23	17	1	40	39	23	16	1	39	104205.5871	0.0109	0.020	0.0109	0.50
790:	40	24	16	1	40	39	24	15	1	39	104233.2230	0.0834	0.060	0.0834	0.50
791:	40	24	17	1	40	39	24	16	1	39	104233.2230	0.0834	0.060	0.0834	0.50
792:	40	25	16	1	40	39	25	15	1	39	104261.9595	0.0457	0.040	0.0457	0.50
793:	40	25	15	1	40	39	25	14	1	39	104261.9595	0.0457	0.040	0.0457	0.50
794:	40	1	39	1	40	39	1	38	1	39	104324.6563	-0.0010	0.020		
795:	40	29	11	1	40	39	29	10	1	39	104388.8998	0.0333	0.020	0.0334	0.50
796:	40	29	12	1	40	39	29	11	1	39	104388.8998	0.0333	0.020	0.0334	0.50
797:	40	3	37	1	40	39	3	36	1	39	104488.6987	0.0857	0.060		
798:	41	1	41	1	41	40	1	40	1	40	104527.2701	0.0234	0.020		
799:	41	0	41	1	41	40	0	40	1	40	104674.6716	-0.0011	0.020		
800:	67	4	64	1	67	67	3	65	1	67	105041.4196	-0.0087	0.040		
801:	40	2	38	1	40	39	2	37	1	39	105113.1172	0.0166	0.020		
802:	67	3	65	1	67	67	2	66	1	67	105177.4503	0.0218	0.040		
803:	75	5	70	1	75	75	4	71	1	75	105307.4014	0.0377	0.040		
804:	42	0	42	1	42	41	1	41	1	41	105791.9210	0.0260	0.020		
805:	88	3	85	1	88	88	2	86	1	88	105835.4022	0.0200	0.060		
806:	41	2	40	1	41	40	2	39	1	40	105988.5326	-0.0119	0.020		
807:	41	1	41	1	41	40	0	40	1	40	106076.5775	-0.0006	0.020		
808:	74	5	69	1	74	74	4	70	1	74	106365.2251	0.0028	0.040		
809:	41	8	34	1	41	40	8	33	1	40	106566.6233	0.0588	0.020	0.0124	0.25
810:	41	8	33	1	41	40	8	32	1	40	106566.6233	0.0588	0.020	0.0124	0.25
811:	41	9	33	1	41	40	9	32	1	40	106566.6233	-0.0340	0.020	0.0124	0.25
812:	41	9	32	1	41	40	9	31	1	40	106566.6233	-0.0340	0.020	0.0124	0.25
813:	41	10	31	1	41	40	10	30	1	40	106570.9332	-0.0504	0.040	-0.0504	0.50
814:	41	10	32	1	41	40	10	31	1	40	106570.9332	-0.0504	0.040	-0.0504	0.50
815:	41	7	35	1	41	40	7	34	1	40	106572.6201	0.0108	0.020	0.0104	0.50
816:	41	7	34	1	41	40	7	33	1	40	106572.6201	0.0099	0.020	0.0104	0.50
817:	41	11	31	1	41	40	11	30	1	40	106578.4767	-0.0056	0.020	-0.0057	0.50
818:	41	11	30	1	41	40	11	29	1	40	106578.4767	-0.0056	0.020	-0.0057	0.50
819:	41	6	36	1	41	40	6	35	1	40	106588.5329	0.0367	0.020	0.0106	0.25
820:	41	12	30	1	41	40	12	29	1	40	106588.5329	0.0096	0.020	0.0106	0.25
821:	41	12	29	1	41	40	12	28	1	40	106588.5329	0.0096	0.020	0.0106	0.25
822:	41	6	35	1	41	40	6	34	1	40	106588.5329	-0.0139	0.020	0.0106	0.25
823:	69	4	66	1	69	69	3	67	1	69	106598.3954	0.0456	0.040		
824:	41	13	29	1	41	40	13	28	1	40	106600.7327	0.0213	0.020	0.0213	0.50
825:	41	13	28	1	41	40	13	27	1	40	106600.7327	0.0213	0.020	0.0213	0.50
826:	41	14	27	1	41	40	14	26	1	40	106614.8025	0.0136	0.020	0.0137	0.50
827:	41	14	28	1	41	40	14	27	1	40	106614.8025	0.0136	0.020	0.0137	0.50
828:	41	3	39	1	41	40	3	38	1	40	106617.4359	0.0240	0.020		
829:	41	5	37	1	41	40	5	36	1	40	106621.5605	0.0044	0.020		
830:	41	5	36	1	41	40	5	35	1	40	106623.5885	0.0543	0.040		
831:	41	15	27	1	41	40	15	26	1	40	106630.5834	0.0024	0.020	0.0025	0.50
832:	41	15	26	1	41	40	15	25	1	40	106630.5834	0.0024	0.020	0.0025	0.50
833:	41	16	26	1	41	40	16	25	1	40	106647.9792	0.0138	0.020	0.0139	0.50
834:	41	16	25	1	41	40	16	24	1	40	106647.9792	0.0138	0.020	0.0139	0.50
835:	41	17	24	1	41	40	17	23	1	40	106666.8555	0.0013	0.020	0.0014	0.50
836:	41	17	25	1	41	40	17	24	1	40	106666.8555	0.0013	0.020	0.0014	0.50
837:	41	4	38	1	41	40	4	37	1	40	106673.7852	0.0195	0.020		
838:	41	18	23	1	41	40	18	22	1	40	106687.1980	0.0155	0.020	0.0156	0.50
839:	41	18	24	1	41	40	18	23	1	40	106687.1980	0.0155	0.020	0.0156	0.50
840:	41	19	22	1	41	40	19	21	1	40	106708.9086	0.0075	0.020	0.0075	0.50
841:	41	19	23	1	41	40	19	22	1	40	106708.9086	0.0075	0.020	0.0075	0.50
842:	41	4	37	1	41	40	4	36	1	40	106720.1595	0.0254	0.020		
843:	41	20	21	1	41	40	20	20	1	40	106731.9265	-0.0454	0.040	-0.0454	0.50
844:	41	20	22	1	41	40	20	21	1	40	106731.9265	-0.0454	0.040	-0.0454	0.50
845:	41	22	20	1	41	40	22	19	1	40	106782.0298	-0.0250	0.020	-0.0251	0.50
846:	41	22	19	1	41	40	22	18	1	40	106782.0298	-0.0250	0.020	-0.0251	0.50
847:	41	24	18	1	41	40	24	17	1	40	106837.2208	-0.0268	0.020	-0.0268	0.50
848:	41	24	17	1	41	40	24	16	1	40	106837.2208	-0.0268	0.020	-0.0268	0.50
849:	46	1	45	1	46	45	2	44	1	45	106839.2082	0.0277	0.040		
850:	41	25	17	1	41	40	25	16	1	40	106866.7482	0.0310	0.020	0.0311	0.50
851:	41	25	16	1	41	40	25	15	1	40	106866.7482	0.0310	0.020	0.0311	0.50
852:	41	1	40	1	41	40	1	39	1	40	106873.5543	-0.0138	0.020		
853:	41	26	15	1	41	40	26	14	1	40	106897.4693	0.0530	0.040	0.0530	0.50
854:	41	26	16	1	41	40	26	15	1	40	106897.4693	0.0530	0.040	0.0530	0.50
855:	41	27	15	1	41	40	27	14	1	40	106929.2553	-0.0768	0.040	-0.0769	0.50
856:	41	27	14	1	41	40	27	13	1	40	106929.2553	-0.0768	0.040	-0.0769	0.50
857:	41	28	13	1	41	40	28	12	1	40	106962.4607	0.0081	0.020	0.0081	0.50
858:	41	28	14	1	41	40	28	13	1	40	106962.4607	0.0081	0.020	0.0081	0.50
859:	41	29	12	1	41	40	29	11	1	40	106996.7934	0.0276	0.020	0.0276	0.50
860:	41	29	13	1	41	40	29	12	1	40	106996.7934	0.0276	0.020	0.0276	0.50
861:	42	1	42	1	42	41	1	41	1	41	107058.9379	0.0403	0.020		
862:	41	31	10	1	41	40	31	9	1	40	107068.8933	-0.0309	0.020	-0.0309	0.50
863:	41	31	11	1	41	40	31	10	1	40	107068.8933	-0.0309	0.020	-0.0309	0.50
864:	41	3	38	1	41	40	3	37	1	40	107137.2159	0.0061	0.020		
865:	42	0	42	1	42	41	0	41	1	41	107193.7888	-0.0116	0.020		
866:	41	2	39	1	41	40	2	38	1	40	107743.8596	0.0173	0.020		
867:	32	2	31	1	32	31	1	30	1	31	108066.6416	-0.0063	0.040		

868:	75 2 73	1 75	75 1 74	1 75	108127.5757	-0.0365	0.060		
869:	43 0 43	1 43	42 1 42	1 42	108446.1383	-0.0074	0.020		
870:	42 1 42	1 42	41 0 41	1 41	108460.8256	0.0225	0.020		
871:	42 2 41	1 42	41 2 40	1 41	108548.8182	0.0299	0.020		
872:	42 9 33	1 42	41 9 32	1 41	109165.7340	-0.0092	0.020	-0.0093	0.50
873:	42 9 34	1 42	41 9 33	1 41	109165.7340	-0.0092	0.020	-0.0093	0.50
874:	42 8 34	1 42	41 8 33	1 41	109166.1755	0.0027	0.020	0.0028	0.50
875:	42 8 35	1 42	41 8 34	1 41	109166.1755	0.0027	0.020	0.0028	0.50
876:	42 10 32	1 42	41 10 31	1 41	109169.8098	0.0076	0.020	0.0077	0.50
877:	42 10 33	1 42	41 10 32	1 41	109169.8098	0.0076	0.020	0.0077	0.50
878:	42 7 36	1 42	41 7 35	1 41	109173.1499	0.0110	0.020	0.0104	0.50
879:	42 7 35	1 42	41 7 34	1 41	109173.1499	0.0098	0.020	0.0104	0.50
880:	42 11 32	1 42	41 11 31	1 41	109177.1891	-0.0196	0.020	-0.0196	0.50
881:	42 11 31	1 42	41 11 30	1 41	109177.1891	-0.0196	0.020	-0.0196	0.50
882:	42 12 30	1 42	41 12 29	1 41	109187.2667	-0.0187	0.020	-0.0188	0.50
883:	42 12 31	1 42	41 12 30	1 41	109187.2667	-0.0187	0.020	-0.0188	0.50
884:	42 6 37	1 42	41 6 36	1 41	109190.6655	0.0410	0.020		
885:	42 6 36	1 42	41 6 35	1 41	109190.6775	-0.0129	0.020		
886:	42 13 29	1 42	41 13 28	1 41	109199.5965	-0.0115	0.020	-0.0116	0.50
887:	42 13 30	1 42	41 13 29	1 41	109199.5965	-0.0115	0.020	-0.0116	0.50
888:	42 3 40	1 42	41 3 39	1 41	109212.6843	0.0032	0.020		
889:	42 14 28	1 42	41 14 27	1 41	109213.9076	0.0082	0.020	0.0083	0.50
890:	42 14 29	1 42	41 14 28	1 41	109213.9076	0.0082	0.020	0.0083	0.50
891:	42 5 38	1 42	41 5 37	1 41	109226.3809	-0.0160	0.020		
892:	42 5 37	1 42	41 5 36	1 41	109228.8227	-0.0264	0.020		
893:	42 15 27	1 42	41 15 26	1 41	109229.9857	0.0140	0.020	0.0141	0.50
894:	42 15 28	1 42	41 15 27	1 41	109229.9857	0.0140	0.020	0.0141	0.50
895:	42 16 26	1 42	41 16 25	1 41	109247.7380	0.0444	0.040	0.0444	0.50
896:	42 16 27	1 42	41 16 26	1 41	109247.7380	0.0444	0.040	0.0444	0.50
897:	42 17 25	1 42	41 17 24	1 41	109266.9441	-0.0268	0.020	-0.0268	0.50
898:	42 17 26	1 42	41 17 25	1 41	109266.9441	-0.0268	0.020	-0.0268	0.50
899:	42 4 39	1 42	41 4 38	1 41	109280.9094	0.0055	0.020		
900:	42 18 24	1 42	41 18 23	1 41	109287.7489	0.0149	0.020	0.0149	0.50
901:	42 18 25	1 42	41 18 24	1 41	109287.7489	0.0149	0.020	0.0149	0.50
902:	42 19 23	1 42	41 19 22	1 41	109309.9242	-0.0058	0.020	-0.0059	0.50
903:	42 19 24	1 42	41 19 23	1 41	109309.9242	-0.0058	0.020	-0.0059	0.50
904:	42 20 22	1 42	41 20 21	1 41	109333.5200	0.0016	0.020	0.0016	0.50
905:	42 20 23	1 42	41 20 22	1 41	109333.5200	0.0016	0.020	0.0016	0.50
906:	42 4 38	1 42	41 4 37	1 41	109335.5084	0.0059	0.020		
907:	42 21 21	1 42	41 21 20	1 41	109358.4595	-0.0070	0.020	-0.0071	0.50
908:	42 21 22	1 42	41 21 21	1 41	109358.4595	-0.0070	0.020	-0.0071	0.50
909:	42 22 20	1 42	41 22 19	1 41	109384.7577	0.0093	0.020	0.0093	0.50
910:	42 22 21	1 42	41 22 20	1 41	109384.7577	0.0093	0.020	0.0093	0.50
911:	42 1 41	1 42	41 1 40	1 41	109418.4010	0.0342	0.020		
912:	42 24 19	1 42	41 24 18	1 41	109441.1951	-0.0332	0.020	-0.0333	0.50
913:	42 24 18	1 42	41 24 17	1 41	109441.1951	-0.0332	0.020	-0.0333	0.50
914:	36 8 29	1 36	37 7 30	1 37	109462.9766	-0.0122	0.040	-0.0126	0.50
915:	36 8 28	1 36	37 7 31	1 37	109462.9766	-0.0129	0.040	-0.0126	0.50
916:	42 25 17	1 42	41 25 16	1 41	109471.3755	-0.0159	0.020	-0.0159	0.50
917:	42 25 18	1 42	41 25 17	1 41	109471.3755	-0.0159	0.020	-0.0159	0.50
918:	42 26 16	1 42	41 26 15	1 41	109502.8352	0.0188	0.020	0.0188	0.50
919:	42 26 17	1 42	41 26 16	1 41	109502.8352	0.0188	0.020	0.0188	0.50
920:	42 27 15	1 42	41 27 14	1 41	109535.4524	-0.0372	0.020	-0.0372	0.50
921:	42 27 16	1 42	41 27 15	1 41	109535.4524	-0.0372	0.020	-0.0372	0.50
922:	42 28 14	1 42	41 28 13	1 41	109569.3788	-0.0195	0.020	-0.0196	0.50
923:	42 28 15	1 42	41 28 14	1 41	109569.3788	-0.0195	0.020	-0.0196	0.50
924:	43 1 43	1 43	42 1 42	1 42	109589.9577	0.0139	0.020		
925:	42 31 11	1 42	41 31 10	1 41	109678.3947	-0.0212	0.020	-0.0212	0.50
926:	42 31 12	1 42	41 31 11	1 41	109678.3947	-0.0212	0.020	-0.0212	0.50
927:	43 0 43	1 43	42 0 42	1 42	109713.2003	0.0519	0.040		
928:	33 2 32	1 33	32 1 31	1 32	109735.7021	0.0161	0.040		
929:	42 3 39	1 42	41 3 38	1 41	109788.2148	0.0140	0.020		
930:	42 35 7	1 42	41 35 6	1 41	109840.3408	0.0100	0.020	0.0100	0.50
931:	42 35 8	1 42	41 35 7	1 41	109840.3408	0.0100	0.020	0.0100	0.50
932:	42 36 6	1 42	41 36 5	1 41	109883.6634	-0.0174	0.020	-0.0175	0.50
933:	42 36 7	1 42	41 36 6	1 41	109883.6634	-0.0174	0.020	-0.0175	0.50
934:	70 3 68	1 70	70 2 69	1 70	109910.6692	-0.0153	0.040		
935:	63 2 62	1 63	63 1 63	1 63	110033.9696	0.0163	0.040		
936:	47 1 46	1 47	46 2 45	1 46	110150.0796	-0.0031	0.040		
937:	42 2 40	1 42	41 2 39	1 41	110371.6682	-0.0033	0.020		
938:	43 1 43	1 43	42 0 42	1 42	110856.9414	-0.0049	0.020		
939:	44 0 44	1 44	43 1 43	1 43	111088.9077	-0.0261	0.020		
940:	43 2 42	1 43	42 2 41	1 42	111107.5080	-0.0124	0.020		
941:	71 3 69	1 71	71 2 70	1 71	111537.8040	-0.0013	0.040		
942:	43 9 34	1 43	42 9 33	1 42	111764.7801	-0.0408	0.040	-0.0409	0.50
943:	43 9 35	1 43	42 9 34	1 42	111764.7801	-0.0408	0.040	-0.0409	0.50
944:	43 8 35	1 43	42 8 34	1 42	111765.8094	-0.0012	0.020	-0.0012	0.50
945:	43 8 36	1 43	42 8 35	1 42	111765.8094	-0.0012	0.020	-0.0012	0.50
946:	43 10 33	1 43	42 10 32	1 42	111768.5748	-0.0108	0.020	-0.0108	0.50
947:	43 10 34	1 43	42 10 33	1 42	111768.5748	-0.0108	0.020	-0.0108	0.50
948:	43 7 36	1 43	42 7 35	1 42	111773.7551	-0.0007	0.020	0.0001	0.50
949:	43 7 37	1 43	42 7 36	1 42	111773.7551	0.0008	0.020	0.0001	0.50
950:	43 11 32	1 43	42 11 31	1 42	111775.8949	0.0145	0.020	0.0145	0.50

951:	43 11 33	1 43	42 11 32	1 42	111775.8949	0.0145	0.020	0.0145	0.50
952:	43 12 31	1 43	42 12 30	1 42	111785.9734	-0.0046	0.020	-0.0046	0.50
953:	43 12 32	1 43	42 12 31	1 42	111785.9734	-0.0046	0.020	-0.0046	0.50
954:	43 6 38	1 43	42 6 37	1 42	111792.9751	0.0488	0.020	0.0061	0.50
955:	43 6 37	1 43	42 6 36	1 42	111792.9751	-0.0365	0.020	0.0061	0.50
956:	43 13 30	1 43	42 13 29	1 42	111798.4226	-0.0008	0.020	-0.0008	0.50
957:	43 13 31	1 43	42 13 30	1 42	111798.4226	-0.0008	0.020	-0.0008	0.50
958:	43 3 41	1 43	42 3 40	1 42	111806.9347	0.0053	0.020		
959:	43 14 30	1 43	42 14 29	1 42	111812.9295	0.0102	0.020	0.0103	0.50
960:	43 14 29	1 43	42 14 28	1 42	111812.9295	0.0102	0.020	0.0103	0.50
961:	43 15 29	1 43	42 15 28	1 42	111829.2637	-0.0004	0.020	-0.0005	0.50
962:	43 15 28	1 43	42 15 27	1 42	111829.2637	-0.0004	0.020	-0.0005	0.50
963:	43 5 39	1 43	42 5 38	1 42	111831.4921	-0.0336	0.020		
964:	43 5 38	1 43	42 5 37	1 42	111834.5789	0.0293	0.020		
965:	43 16 28	1 43	42 16 27	1 42	111847.3019	-0.0156	0.020	-0.0157	0.50
966:	43 16 27	1 43	42 16 26	1 42	111847.3019	-0.0156	0.020	-0.0157	0.50
967:	43 17 26	1 43	42 17 25	1 42	111866.9959	0.0176	0.020	0.0177	0.50
968:	43 17 27	1 43	42 17 26	1 42	111866.9959	0.0176	0.020	0.0177	0.50
969:	43 4 40	1 43	42 4 39	1 42	111888.1467	-0.0068	0.020	-0.0189	0.33
970:	43 18 26	1 43	42 18 25	1 42	111888.1467	-0.0249	0.020	-0.0189	0.33
971:	43 18 25	1 43	42 18 24	1 42	111888.1467	-0.0249	0.020	-0.0189	0.33
972:	43 19 24	1 43	42 19 23	1 42	111910.8639	0.0224	0.020	0.0225	0.50
973:	43 19 25	1 43	42 19 24	1 42	111910.8639	0.0224	0.020	0.0225	0.50
974:	43 20 24	1 43	42 20 23	1 42	111934.9410	-0.0029	0.020	-0.0030	0.50
975:	43 20 23	1 43	42 20 22	1 42	111934.9410	-0.0029	0.020	-0.0030	0.50
976:	43 4 39	1 43	42 4 38	1 42	111952.1610	0.0016	0.020		
977:	43 1 42	1 43	42 1 41	1 42	111959.1646	0.0046	0.020		
978:	43 21 22	1 43	42 21 21	1 42	111960.4595	0.0147	0.020	0.0147	0.50
979:	43 21 23	1 43	42 21 22	1 42	111960.4595	0.0147	0.020	0.0147	0.50
980:	43 23 21	1 43	42 23 20	1 42	112015.4864	-0.0473	0.040	-0.0473	0.50
981:	43 23 20	1 43	42 23 19	1 42	112015.4864	-0.0473	0.040	-0.0473	0.50
982:	43 24 20	1 43	42 24 19	1 42	112045.0827	0.0039	0.020	0.0040	0.50
983:	43 24 19	1 43	42 24 18	1 42	112045.0827	0.0039	0.020	0.0040	0.50
984:	35 8 28	1 35	36 7 29	1 36	112065.2838	-0.0678	0.060	-0.0681	0.50
985:	35 8 27	1 35	36 7 30	1 36	112065.2838	-0.0683	0.060	-0.0681	0.50
986:	68 5 63	1 68	68 4 64	1 68	112069.9793	0.0696	0.040		
987:	43 25 18	1 43	42 25 17	1 42	112075.9457	0.0121	0.020	0.0121	0.50
988:	43 25 19	1 43	42 25 18	1 42	112075.9457	0.0121	0.020	0.0121	0.50
989:	43 26 17	1 43	42 26 16	1 42	112108.0181	-0.0645	0.040	-0.0645	0.50
990:	43 26 18	1 43	42 26 17	1 42	112108.0181	-0.0645	0.040	-0.0645	0.50
991:	44 1 44	1 44	43 1 43	1 43	112120.4083	-0.0063	0.020		
992:	43 27 16	1 43	42 27 15	1 42	112141.5198	0.0081	0.020	0.0081	0.50
993:	43 27 17	1 43	42 27 16	1 42	112141.5198	0.0081	0.020	0.0081	0.50
994:	75 4 72	1 75	75 3 73	1 75	112166.2036	0.0223	0.040		
995:	43 28 15	1 43	42 28 14	1 42	112176.2004	-0.0069	0.020	-0.0070	0.50
996:	43 28 16	1 43	42 28 15	1 42	112176.2004	-0.0069	0.020	-0.0070	0.50
997:	43 29 14	1 43	42 29 13	1 42	112212.1694	0.0125	0.020	0.0125	0.50
998:	43 29 15	1 43	42 29 14	1 42	112212.1694	0.0125	0.020	0.0125	0.50
999:	44 0 44	1 44	43 0 43	1 43	112232.7257	-0.0061	0.020		
1000:	43 30 13	1 43	42 30 12	1 42	112249.3477	0.0000	0.020	0.0001	0.50
1001:	43 30 14	1 43	42 30 13	1 42	112249.3477	0.0000	0.020	0.0001	0.50
1002:	43 31 12	1 43	42 31 11	1 42	112287.7544	-0.0127	0.020	-0.0128	0.50
1003:	43 31 13	1 43	42 31 12	1 42	112287.7544	-0.0127	0.020	-0.0128	0.50
1004:	43 32 11	1 43	42 32 10	1 42	112327.4151	0.0120	0.020	0.0121	0.50
1005:	43 32 12	1 43	42 32 11	1 42	112327.4151	0.0120	0.020	0.0121	0.50
1006:	43 34 9	1 43	42 34 8	1 42	112410.2727	0.0006	0.020	0.0007	0.50
1007:	43 34 10	1 43	42 34 9	1 42	112410.2727	0.0006	0.020	0.0007	0.50
1008:	43 3 40	1 43	42 3 39	1 42	112441.4277	-0.0025	0.020		
1009:	77 2 75	1 77	77 1 76	1 77	112958.4751	-0.0447	0.060		
1010:	43 2 41	1 43	42 2 40	1 42	112996.4416	-0.0001	0.020		
1011:	35 2 34	1 35	34 1 33	1 34	113047.3754	-0.0452	0.040		
1012:	44 1 44	1 44	43 0 43	1 43	113264.1964	-0.0162	0.020		
1013:	66 1 65	1 66	66 0 66	1 66	113439.5034	-0.0359	0.020		
1014:	44 2 43	1 44	43 2 42	1 43	113664.7211	-0.0244	0.020		
1015:	66 5 61	1 66	66 4 62	1 66	113673.9102	0.0008	0.040		
1016:	45 0 45	1 45	44 1 44	1 44	113721.0345	-0.0423	0.040		
1017:	77 4 74	1 77	77 3 75	1 77	114320.5567	0.0210	0.040		
1018:	44 9 35	1 44	43 9 34	1 43	114363.8991	0.0088	0.020	0.0089	0.50
1019:	44 9 36	1 44	43 9 35	1 43	114363.8991	0.0088	0.020	0.0089	0.50
1020:	44 8 37	1 44	43 8 36	1 43	114365.4779	-0.0009	0.020	-0.0009	0.50
1021:	44 8 36	1 44	43 8 35	1 43	114365.4779	-0.0009	0.020	-0.0009	0.50
1022:	44 10 35	1 44	43 10 34	1 43	114367.3351	0.0018	0.020	0.0018	0.50
1023:	44 10 34	1 44	43 10 33	1 43	114367.3351	0.0018	0.020	0.0018	0.50
1024:	44 7 38	1 44	43 7 37	1 43	114374.4721	0.0147	0.020	-0.0051	0.25
1025:	44 7 37	1 44	43 7 36	1 43	114374.4721	0.0125	0.020	-0.0051	0.25
1026:	44 11 34	1 44	43 11 33	1 43	114374.4721	-0.0238	0.020	-0.0051	0.25
1027:	44 11 33	1 44	43 11 32	1 43	114374.4721	-0.0238	0.020	-0.0051	0.25
1028:	44 12 33	1 44	43 12 32	1 43	114384.6007	0.0015	0.020	0.0015	0.50
1029:	44 12 32	1 44	43 12 31	1 43	114384.6007	0.0015	0.020	0.0015	0.50
1030:	44 6 38	1 44	43 6 37	1 43	114395.4634	-0.0520	0.040	0.0030	0.50
1031:	44 6 39	1 44	43 6 38	1 43	114395.4634	0.0579	0.040	0.0030	0.50
1032:	44 13 31	1 44	43 13 30	1 43	114397.1645	0.0090	0.020	0.0091	0.50
1033:	44 13 32	1 44	43 13 31	1 43	114397.1645	0.0090	0.020	0.0091	0.50

1034:	44	3	42	1	44	43	3	41	1	43	114400.0877	-0.0075	0.020		
1035:	44	14	30	1	44	43	14	29	1	43	114411.8429	-0.0033	0.020	-0.0034	0.50
1036:	44	14	31	1	44	43	14	30	1	43	114411.8429	-0.0033	0.020	-0.0034	0.50
1037:	44	15	30	1	44	43	15	29	1	43	114428.4541	-0.0021	0.020	-0.0022	0.50
1038:	44	15	29	1	44	43	15	28	1	43	114428.4541	-0.0021	0.020	-0.0022	0.50
1039:	44	5	40	1	44	43	5	39	1	43	114436.9372	-0.0068	0.020		
1040:	44	5	39	1	44	43	5	38	1	43	114440.6570	0.0032	0.020		
1041:	44	16	29	1	44	43	16	28	1	43	114446.8318	-0.0029	0.020	-0.0029	0.50
1042:	44	16	28	1	44	43	16	27	1	43	114446.8318	-0.0029	0.020	-0.0029	0.50
1043:	44	17	27	1	44	43	17	26	1	43	114466.8916	0.0181	0.020	0.0181	0.50
1044:	44	17	28	1	44	43	17	27	1	43	114466.8916	0.0181	0.020	0.0181	0.50
1045:	44	18	27	1	44	43	18	26	1	43	114488.4863	-0.0064	0.020	-0.0065	0.50
1046:	44	18	26	1	44	43	18	25	1	43	114488.4863	-0.0064	0.020	-0.0065	0.50
1047:	44	4	41	1	44	43	4	40	1	43	114495.4730	-0.0050	0.020		
1048:	44	1	43	1	44	43	1	42	1	43	114496.0961	0.0064	0.020		
1049:	44	20	24	1	44	43	20	23	1	43	114536.2656	0.0197	0.020	0.0197	0.50
1050:	44	20	25	1	44	43	20	24	1	43	114536.2656	0.0197	0.020	0.0197	0.50
1051:	44	21	23	1	44	43	21	22	1	43	114562.2942	-0.0021	0.020	-0.0021	0.50
1052:	44	21	24	1	44	43	21	23	1	43	114562.2942	-0.0021	0.020	-0.0021	0.50
1053:	44	4	40	1	44	43	4	39	1	43	114570.1855	-0.0071	0.020		
1054:	44	22	23	1	44	43	22	22	1	43	114589.7483	-0.0056	0.020	-0.0056	0.50
1055:	44	22	22	1	44	43	22	21	1	43	114589.7483	-0.0056	0.020	-0.0056	0.50
1056:	44	23	21	1	44	43	23	20	1	43	114618.5897	-0.0043	0.020	-0.0044	0.50
1057:	44	23	22	1	44	43	23	21	1	43	114618.5897	-0.0043	0.020	-0.0044	0.50
1058:	44	24	21	1	44	43	24	20	1	43	114648.7680	-0.0276	0.020	-0.0276	0.50
1059:	44	24	20	1	44	43	24	19	1	43	114648.7680	-0.0276	0.020	-0.0276	0.50
1060:	45	1	45	1	45	44	1	44	1	44	114650.3387	0.0002	0.020		
1061:	34	8	27	1	34	35	7	28	1	35	114667.0145	-0.0127	0.040	-0.0129	0.50
1062:	34	8	26	1	34	35	7	29	1	35	114667.0145	-0.0130	0.040	-0.0129	0.50
1063:	44	25	20	1	44	43	25	19	1	43	114680.3079	-0.0324	0.020	-0.0324	0.50
1064:	44	25	19	1	44	43	25	18	1	43	114680.3079	-0.0324	0.020	-0.0324	0.50
1065:	44	26	18	1	44	43	26	17	1	43	114713.2041	-0.0077	0.020	-0.0078	0.50
1066:	44	26	19	1	44	43	26	18	1	43	114713.2041	-0.0077	0.020	-0.0078	0.50
1067:	44	27	18	1	44	43	27	17	1	43	114747.3959	0.0006	0.020	0.0007	0.50
1068:	44	27	17	1	44	43	27	16	1	43	114747.3959	0.0006	0.020	0.0007	0.50
1069:	45	0	45	1	45	44	0	44	1	44	114752.5498	-0.0078	0.020		
1070:	44	28	16	1	44	43	28	15	1	43	114782.9066	0.0301	0.020	0.0302	0.50
1071:	44	28	17	1	44	43	28	16	1	43	114782.9066	0.0301	0.020	0.0302	0.50
1072:	44	29	16	1	44	43	29	15	1	43	114819.6387	-0.0034	0.020	-0.0034	0.50
1073:	44	29	15	1	44	43	29	14	1	43	114819.6387	-0.0034	0.020	-0.0034	0.50
1074:	44	31	13	1	44	43	31	12	1	43	114896.9580	-0.0167	0.020	-0.0168	0.50
1075:	44	31	14	1	44	43	31	13	1	43	114896.9580	-0.0167	0.020	-0.0168	0.50
1076:	44	32	12	1	44	43	32	11	1	43	114937.4934	-0.0224	0.020	-0.0224	0.50
1077:	44	32	13	1	44	43	32	12	1	43	114937.4934	-0.0224	0.020	-0.0224	0.50
1078:	44	33	11	1	44	43	33	10	1	43	114979.2938	0.0045	0.020	0.0046	0.50
1079:	44	33	12	1	44	43	33	11	1	43	114979.2938	0.0045	0.020	0.0046	0.50
1080:	44	34	10	1	44	43	34	9	1	43	115022.2840	0.0026	0.020	0.0026	0.50
1081:	44	34	11	1	44	43	34	10	1	43	115022.2840	0.0026	0.020	0.0026	0.50
1082:	44	3	41	1	44	43	3	40	1	43	115096.6964	-0.0097	0.020		
1083:	44	2	42	1	44	43	2	41	1	43	115618.0174	0.0042	0.020		
1084:	45	1	45	1	45	44	0	44	1	44	115681.8129	-0.0063	0.020		
1085:	23	7	17	1	23	24	6	18	1	24	115902.0093	0.0856	0.040	0.0855	0.50
1086:	23	7	16	1	23	24	6	19	1	24	115902.0093	0.0853	0.040	0.0855	0.50
1087:	45	2	44	1	45	44	2	43	1	44	116220.4674	-0.0037	0.020		
1088:	37	2	36	1	37	36	1	35	1	36	116340.4778	-0.0163	0.040		
1089:	46	0	46	1	46	45	1	45	1	45	116343.3342	-0.0289	0.020		
1090:	62	5	57	1	62	62	4	58	1	62	116395.5767	-0.0261	0.060		
1091:	79	4	76	1	79	79	3	77	1	79	116621.0939	0.0769	0.060		
1092:	49	1	48	1	49	48	2	47	1	48	116682.9466	0.0095	0.040		
1093:	8	4	5	1	8	7	3	4	1	7	116858.3178	0.0462	0.040		
1094:	45	9	36	1	45	44	9	35	1	44	116962.9593	0.0085	0.020	0.0086	0.50
1095:	45	9	37	1	45	44	9	36	1	44	116962.9593	0.0085	0.020	0.0086	0.50
1096:	45	8	38	1	45	44	8	37	1	44	116965.1751	-0.0027	0.020	-0.0028	0.50
1097:	45	8	37	1	45	44	8	36	1	44	116965.1751	-0.0028	0.020	-0.0028	0.50
1098:	45	10	35	1	45	44	10	34	1	44	116966.0492	0.0051	0.020	0.0051	0.50
1099:	45	10	36	1	45	44	10	35	1	44	116966.0492	0.0051	0.020	0.0051	0.50
1100:	45	11	35	1	45	44	11	34	1	44	116973.0600	0.0059	0.020	0.0059	0.50
1101:	45	11	34	1	45	44	11	33	1	44	116973.0600	0.0059	0.020	0.0059	0.50
1102:	45	7	39	1	45	44	7	38	1	44	116975.2536	0.0032	0.020	0.0018	0.50
1103:	45	7	38	1	45	44	7	37	1	44	116975.2536	0.0003	0.020	0.0018	0.50
1104:	61	5	56	1	61	61	4	57	1	61	116976.7118	0.0197	0.040		
1105:	45	12	34	1	45	44	12	33	1	44	116983.1534	0.0061	0.020	0.0062	0.50
1106:	45	12	33	1	45	44	12	32	1	44	116983.1534	0.0061	0.020	0.0062	0.50
1107:	45	3	43	1	45	44	3	42	1	44	116992.1161	-0.0026	0.020		
1108:	45	13	32	1	45	44	13	31	1	44	116995.7874	-0.0147	0.020	-0.0147	0.50
1109:	45	13	33	1	45	44	13	32	1	44	116995.7874	-0.0147	0.020	-0.0147	0.50
1110:	45	6	40	1	45	44	6	39	1	44	116998.1239	0.0577	0.040		
1111:	45	6	39	1	45	44	6	38	1	44	116998.1239	-0.0828	0.060		
1112:	45	14	31	1	45	44	14	30	1	44	117010.6736	-0.0047	0.020	-0.0047	0.50
1113:	45	14	32	1	45	44	14	31	1	44	117010.6736	-0.0047	0.020	-0.0047	0.50
1114:	45	15	31	1	45	44	15	30	1	44	117027.5393	-0.0061	0.020	-0.0062	0.50
1115:	45	15	30	1	45	44	15	29	1	44	117027.5393	-0.0061	0.020	-0.0062	0.50
1116:	45	1	44	1	45	44	1	43	1	44	117029.3180	-0.0138	0.020		

1117:	45	5	41	1	45	44	5	40	1	44	117042.6487	-0.0038	0.020		
1118:	45	16	30	1	45	44	16	29	1	44	117046.2358	-0.0067	0.020	-0.0067	0.50
1119:	45	16	29	1	45	44	16	28	1	44	117046.2358	-0.0067	0.020	-0.0067	0.50
1120:	45	5	40	1	45	44	5	39	1	44	117047.1812	-0.0003	0.020		
1121:	45	17	29	1	45	44	17	28	1	44	117066.6778	0.0238	0.020	0.0239	0.50
1122:	45	17	28	1	45	44	17	27	1	44	117066.6778	0.0238	0.020	0.0239	0.50
1123:	45	18	28	1	45	44	18	27	1	44	117088.6878	-0.0067	0.020	-0.0068	0.50
1124:	45	18	27	1	45	44	18	26	1	44	117088.6878	-0.0067	0.020	-0.0068	0.50
1125:	45	4	42	1	45	44	4	41	1	44	117102.8265	-0.0112	0.020		
1126:	45	19	26	1	45	44	19	25	1	44	117112.3164	0.0162	0.020	0.0162	0.50
1127:	45	19	27	1	45	44	19	26	1	44	117112.3164	0.0162	0.020	0.0162	0.50
1128:	45	20	26	1	45	44	20	25	1	44	117137.4118	-0.0093	0.020	-0.0094	0.50
1129:	45	20	25	1	45	44	20	24	1	44	117137.4118	-0.0093	0.020	-0.0094	0.50
1130:	45	21	24	1	45	44	21	23	1	44	117164.0075	-0.0108	0.020	-0.0108	0.50
1131:	45	21	25	1	45	44	21	24	1	44	117164.0075	-0.0108	0.020	-0.0108	0.50
1132:	46	1	46	1	46	45	1	45	1	45	117179.7714	0.0286	0.020		
1133:	45	4	41	1	45	44	4	40	1	44	117189.6826	-0.0113	0.020		
1134:	45	22	23	1	45	44	22	22	1	44	117192.0454	-0.0146	0.020	-0.0146	0.50
1135:	45	22	24	1	45	44	22	23	1	44	117192.0454	-0.0146	0.020	-0.0146	0.50
1136:	45	23	22	1	45	44	23	21	1	44	117221.5301	0.0101	0.020	0.0101	0.50
1137:	45	23	23	1	45	44	23	22	1	44	117221.5301	0.0101	0.020	0.0101	0.50
1138:	45	24	21	1	45	44	24	20	1	44	117252.3726	-0.0033	0.020	-0.0034	0.50
1139:	45	24	22	1	45	44	24	21	1	44	117252.3726	-0.0033	0.020	-0.0034	0.50
1140:	46	0	46	1	46	45	0	45	1	45	117272.6178	-0.0069	0.020		
1141:	45	25	21	1	45	44	25	20	1	44	117284.6526	0.0439	0.040	0.0439	0.50
1142:	45	25	20	1	45	44	25	19	1	44	117284.6526	0.0439	0.040	0.0439	0.50
1143:	45	26	19	1	45	44	26	18	1	44	117318.1970	-0.0039	0.020	-0.0039	0.50
1144:	45	26	20	1	45	44	26	19	1	44	117318.1970	-0.0039	0.020	-0.0039	0.50
1145:	45	27	19	1	45	44	27	18	1	44	117353.1554	0.0183	0.020	0.0184	0.50
1146:	45	27	18	1	45	44	27	17	1	44	117353.1554	0.0183	0.020	0.0184	0.50
1147:	45	28	17	1	45	44	28	16	1	44	117389.4248	0.0225	0.020	0.0225	0.50
1148:	45	28	18	1	45	44	28	17	1	44	117389.4248	0.0225	0.020	0.0225	0.50
1149:	60	5	55	1	60	60	4	56	1	60	117519.8997	0.0858	0.060		
1150:	67	2	66	1	67	67	1	67	1	67	117600.0107	-0.0229	0.040		
1151:	45	3	42	1	45	44	3	41	1	44	117753.7906	-0.0102	0.020		
1152:	80	4	77	1	80	80	3	78	1	80	117825.0159	-0.0112	0.040		
1153:	46	1	46	1	46	45	0	45	1	45	118108.9636	-0.0408	0.040		
1154:	45	2	43	1	45	44	2	42	1	44	118236.2582	0.0074	0.020		
1155:	46	2	45	1	46	45	2	44	1	45	118774.7029	-0.0050	0.020		
1156:	57	5	52	1	57	57	4	53	1	57	118934.5954	0.0754	0.040		
1157:	47	0	47	1	47	46	1	46	1	46	118956.5562	0.0090	0.020		
1158:	56	5	51	1	56	56	4	52	1	56	119339.6020	0.0746	0.040		
1159:	68	2	67	1	68	68	1	68	1	68	119508.6975	0.0330	0.040		
1160:	20	3	18	1	20	19	2	17	1	19	119516.7401	0.0303	0.040		
1161:	46	1	45	1	46	45	1	44	1	45	119559.0936	0.0000	0.020		
1162:	46	9	37	1	46	45	9	36	1	45	119562.0086	0.0064	0.020	0.0064	0.50
1163:	46	9	38	1	46	45	9	37	1	45	119562.0086	0.0064	0.020	0.0064	0.50
1164:	46	10	36	1	46	45	10	35	1	45	119564.7578	0.0406	0.020	0.0406	0.50
1165:	46	10	37	1	46	45	10	36	1	45	119564.7578	0.0406	0.020	0.0406	0.50
1166:	46	8	39	1	46	45	8	38	1	45	119564.8871	-0.0213	0.020	-0.0214	0.50
1167:	46	8	38	1	46	45	8	37	1	45	119564.8871	-0.0214	0.020	-0.0214	0.50
1168:	46	11	35	1	46	45	11	34	1	45	119571.5573	0.0039	0.020	0.0039	0.50
1169:	46	11	36	1	46	45	11	35	1	45	119571.5573	0.0039	0.020	0.0039	0.50
1170:	46	7	39	1	46	45	7	38	1	45	119576.1310	-0.0080	0.020	-0.0062	0.50
1171:	46	7	40	1	46	45	7	39	1	45	119576.1310	-0.0042	0.020	-0.0062	0.50
1172:	46	12	35	1	46	45	12	34	1	45	119581.6096	-0.0108	0.020	-0.0108	0.50
1173:	46	12	34	1	46	45	12	33	1	45	119581.6096	-0.0108	0.020	-0.0108	0.50
1174:	46	3	44	1	46	45	3	43	1	45	119582.9454	0.0033	0.020		
1175:	46	13	33	1	46	45	13	32	1	45	119594.3527	-0.0088	0.020	-0.0089	0.50
1176:	46	13	34	1	46	45	13	33	1	45	119594.3527	-0.0088	0.020	-0.0089	0.50
1177:	46	6	40	1	46	45	6	39	1	45	119601.0076	-0.0834	0.020	0.0060	0.50
1178:	46	6	41	1	46	45	6	40	1	45	119601.0076	0.0953	0.020	0.0060	0.50
1179:	46	14	32	1	46	45	14	31	1	45	119609.4095	-0.0037	0.020	-0.0037	0.50
1180:	46	14	33	1	46	45	14	32	1	45	119609.4095	-0.0037	0.020	-0.0037	0.50
1181:	46	15	32	1	46	45	15	31	1	45	119626.5300	0.0005	0.020	0.0005	0.50
1182:	46	15	31	1	46	45	15	30	1	45	119626.5300	0.0005	0.020	0.0005	0.50
1183:	39	2	38	1	39	38	1	37	1	38	119635.8154	0.0226	0.040		
1184:	46	16	31	1	46	45	16	30	1	45	119645.5361	-0.0024	0.020	-0.0024	0.50
1185:	46	16	30	1	46	45	16	29	1	45	119645.5361	-0.0024	0.020	-0.0024	0.50
1186:	46	5	42	1	46	45	5	41	1	45	119648.6580	0.0070	0.020		
1187:	46	5	41	1	46	45	5	40	1	45	119654.1458	-0.0084	0.020		
1188:	46	17	29	1	46	45	17	28	1	45	119666.3141	-0.0030	0.020	-0.0030	0.50
1189:	46	17	30	1	46	45	17	29	1	45	119666.3141	-0.0030	0.020	-0.0030	0.50
1190:	46	18	29	1	46	45	18	28	1	45	119688.7450	-0.0294	0.020	-0.0294	0.50
1191:	46	18	28	1	46	45	18	27	1	45	119688.7450	-0.0294	0.020	-0.0294	0.50
1192:	47	1	47	1	47	46	1	46	1	46	119708.6407	-0.0129	0.020		
1193:	46	4	43	1	46	45	4	42	1	45	119710.1856	-0.0041	0.020		
1194:	46	19	28	1	46	45	19	27	1	45	119712.8285	-0.0134	0.020	-0.0135	0.50
1195:	46	19	27	1	46	45	19	26	1	45	119712.8285	-0.0134	0.020	-0.0135	0.50
1196:	55	5	50	1	55	55	4	51	1	55	119713.9938	0.0484	0.040		
1197:	69	1	68	1	69	69	0	69	1	69	119724.2296	-0.0115	0.020		
1198:	46	20	27	1	46	45	20	26	1	45	119738.4466	-0.0204	0.020	-0.0204	0.50
1199:	46	20	26	1	46	45	20	25	1	45	119738.4466	-0.0204	0.020	-0.0204	0.50

1200:	46	21	26	1	46	45	21	25	1	45	119765.5754	-0.0324	0.020	-0.0325	0.50
1201:	46	21	25	1	46	45	21	24	1	45	119765.5754	-0.0324	0.020	-0.0325	0.50
1202:	47	0	47	1	47	46	0	46	1	46	119792.9232	-0.0035	0.020		
1203:	46	4	42	1	46	45	4	41	1	45	119810.7530	-0.0054	0.020		
1204:	46	23	24	1	46	45	23	23	1	45	119824.3058	-0.0025	0.020	-0.0025	0.50
1205:	46	23	23	1	46	45	23	22	1	45	119824.3058	-0.0025	0.020	-0.0025	0.50
1206:	46	24	22	1	46	45	24	21	1	45	119855.8284	0.0117	0.020	0.0118	0.50
1207:	46	24	23	1	46	45	24	22	1	45	119855.8284	0.0117	0.020	0.0118	0.50
1208:	46	25	21	1	46	45	25	20	1	45	119888.7211	-0.0143	0.020	-0.0144	0.50
1209:	46	25	22	1	46	45	25	21	1	45	119888.7211	-0.0143	0.020	-0.0144	0.50
1210:	50	1	49	1	50	49	2	48	1	49	119902.7275	0.0767	0.060		
1211:	46	27	20	1	46	45	27	19	1	45	119958.7111	-0.0227	0.020	-0.0228	0.50
1212:	46	27	19	1	46	45	27	18	1	45	119958.7111	-0.0227	0.020	-0.0228	0.50
1213:	46	28	19	1	46	45	28	18	1	45	119995.7446	-0.0370	0.020	-0.0371	0.50
1214:	46	28	18	1	46	45	28	17	1	45	119995.7446	-0.0370	0.020	-0.0371	0.50
1215:	76	3	74	1	76	76	2	75	1	76	119999.3179	0.0362	0.040		
1216:	54	5	49	1	54	54	4	50	1	54	120059.4635	0.0158	0.040		
1217:	46	30	17	1	46	45	30	16	1	45	120073.9453	0.0437	0.040	0.0438	0.50
1218:	46	30	16	1	46	45	30	15	1	45	120073.9453	0.0437	0.040	0.0438	0.50
1219:	46	31	16	1	46	45	31	15	1	45	120114.9579	0.0123	0.020	0.0124	0.50
1220:	46	31	15	1	46	45	31	14	1	45	120114.9579	0.0123	0.020	0.0124	0.50
1221:	46	32	14	1	46	45	32	13	1	45	120157.3062	0.0125	0.020	0.0125	0.50
1222:	46	32	15	1	46	45	32	14	1	45	120157.3062	0.0125	0.020	0.0125	0.50
1223:	46	34	12	1	46	45	34	11	1	45	120245.8869	0.0408	0.040	0.0409	0.50
1224:	46	34	13	1	46	45	34	12	1	45	120245.8869	0.0408	0.040	0.0409	0.50
1225:	46	35	11	1	46	45	35	10	1	45	120292.0086	-0.0123	0.020	-0.0123	0.50
1226:	46	35	12	1	46	45	35	11	1	45	120292.0086	-0.0123	0.020	-0.0123	0.50
1227:	53	5	48	1	53	53	4	49	1	53	120377.7002	-0.0146	0.060		
1228:	46	3	43	1	46	45	3	42	1	45	120412.4511	-0.0028	0.020		
1229:	47	1	47	1	47	46	0	46	1	46	120545.0491	0.0158	0.020		
1230:	52	5	47	1	52	52	4	48	1	52	120670.4185	0.0040	0.060		
1231:	46	2	44	1	46	45	2	43	1	45	120851.0361	0.0126	0.020		
1232:	47	2	46	1	47	46	2	45	1	46	121327.4605	-0.0093	0.020		
1233:	49	5	44	1	49	49	4	45	1	49	121411.2538	-0.0151	0.060		
1234:	48	0	48	1	48	47	1	47	1	47	121561.3476	0.0015	0.020		
1235:	48	5	43	1	48	48	4	44	1	48	121617.6034	-0.0031	0.060		
1236:	70	1	69	1	70	70	0	70	1	70	121800.3448	-0.0051	0.020		
1237:	47	5	42	1	47	47	4	43	1	47	121806.1073	0.0584	0.040		
1238:	63	5	59	1	63	63	4	60	1	63	121860.5631	0.0354	0.040		
1239:	59	5	55	1	59	59	4	56	1	59	121893.7241	0.0916	0.060		
1240:	65	5	61	1	65	65	4	62	1	65	121912.0811	0.0383	0.040		
1241:	66	5	62	1	66	66	4	63	1	66	121958.7779	0.0350	0.040		
1242:	67	5	63	1	67	67	4	64	1	67	122020.9864	0.0275	0.040		
1243:	47	1	46	1	47	46	1	45	1	46	122085.6151	0.0048	0.020		
1244:	45	5	40	1	45	45	4	41	1	45	122134.5394	-0.0028	0.060		
1245:	47	9	38	1	47	46	9	37	1	46	122161.0521	0.0078	0.020	0.0078	0.50
1246:	47	9	39	1	47	46	9	38	1	46	122161.0521	0.0078	0.020	0.0078	0.50
1247:	53	5	49	1	53	53	4	50	1	53	122161.7584	0.1312	0.060		
1248:	47	10	38	1	47	46	10	37	1	46	122163.3566	0.0050	0.020	0.0050	0.50
1249:	47	10	37	1	47	46	10	36	1	46	122163.3566	0.0050	0.020	0.0050	0.50
1250:	47	8	40	1	47	46	8	39	1	46	122164.6655	-0.0057	0.020	-0.0058	0.50
1251:	47	8	39	1	47	46	8	38	1	46	122164.6655	-0.0058	0.020	-0.0058	0.50
1252:	47	11	37	1	47	46	11	36	1	46	122169.9891	-0.0033	0.020	-0.0034	0.50
1253:	47	11	36	1	47	46	11	35	1	46	122169.9891	-0.0033	0.020	-0.0034	0.50
1254:	47	3	45	1	47	46	3	44	1	46	122172.5027	-0.0069	0.020		
1255:	47	7	40	1	47	46	7	39	1	46	122177.1239	0.0048	0.020	0.0074	0.50
1256:	47	7	41	1	47	46	7	40	1	46	122177.1239	0.0099	0.020	0.0074	0.50
1257:	47	12	35	1	47	46	12	34	1	46	122180.0131	-0.0040	0.020	-0.0040	0.50
1258:	47	12	36	1	47	46	12	35	1	46	122180.0131	-0.0040	0.020	-0.0040	0.50
1259:	47	13	34	1	47	46	13	33	1	46	122192.8349	0.0032	0.020	0.0032	0.50
1260:	47	13	35	1	47	46	13	34	1	46	122192.8349	0.0032	0.020	0.0032	0.50
1261:	47	6	41	1	47	46	6	40	1	46	122204.1332	-0.0406	0.040		
1262:	47	14	33	1	47	46	14	32	1	46	122208.0446	-0.0041	0.020	-0.0041	0.50
1263:	47	14	34	1	47	46	14	33	1	46	122208.0446	-0.0041	0.020	-0.0041	0.50
1264:	52	5	48	1	52	52	4	49	1	52	122220.5217	0.1117	0.060		
1265:	47	15	33	1	47	46	15	32	1	46	122225.4007	-0.0052	0.020	-0.0052	0.50
1266:	47	15	32	1	47	46	15	31	1	46	122225.4007	-0.0052	0.020	-0.0052	0.50
1267:	48	1	48	1	48	47	1	47	1	47	122237.0744	-0.0218	0.020		
1268:	47	16	32	1	47	46	16	31	1	46	122244.7055	-0.0147	0.020	-0.0147	0.50
1269:	47	16	31	1	47	46	16	30	1	46	122244.7055	-0.0147	0.020	-0.0147	0.50
1270:	47	5	43	1	47	46	5	42	1	46	122254.9261	-0.0117	0.020		
1271:	47	5	42	1	47	46	5	41	1	46	122261.5943	-0.0007	0.020		
1272:	47	17	31	1	47	46	17	30	1	46	122265.8709	0.0105	0.020	0.0106	0.50
1273:	47	17	30	1	47	46	17	29	1	46	122265.8709	0.0105	0.020	0.0106	0.50
1274:	51	5	47	1	51	51	4	48	1	51	122281.3141	0.0758	0.040		
1275:	47	18	29	1	47	46	18	28	1	46	122288.7113	-0.0182	0.020	-0.0182	0.50
1276:	47	18	30	1	47	46	18	29	1	46	122288.7113	-0.0182	0.020	-0.0182	0.50
1277:	47	19	29	1	47	46	19	28	1	46	122313.2679	0.0129	0.020	0.0129	0.50
1278:	47	19	28	1	47	46	19	27	1	46	122313.2679	0.0129	0.020	0.0129	0.50
1279:	48	0	48	1	48	47	0	47	1	47	122313.4540	0.0014	0.020		
1280:	47	4	44	1	47	46	4	43	1	46	122317.5059	0.0173	0.020		
1281:	47	20	27	1	47	46	20	26	1	46	122339.3574	-0.0231	0.020	-0.0232	0.50
1282:	47	20	28	1	47	46	20	27	1	46	122339.3574	-0.0231	0.020	-0.0232	0.50

1283:	50	5	46	1	50	50	4	47	1	50	122343.6021	0.1238	0.060		
1284:	47	21	27	1	47	46	21	26	1	46	122367.0411	-0.0209	0.020	-0.0209	0.50
1285:	47	21	26	1	47	46	21	25	1	46	122367.0411	-0.0209	0.020	-0.0209	0.50
1286:	47	22	25	1	47	46	22	24	1	46	122396.2638	0.0000	0.020	0.0001	0.50
1287:	47	22	26	1	47	46	22	25	1	46	122396.2638	0.0000	0.020	0.0001	0.50
1288:	47	23	24	1	47	46	23	23	1	46	122426.9156	-0.0405	0.040	-0.0406	0.50
1289:	47	23	25	1	47	46	23	24	1	46	122426.9156	-0.0405	0.040	-0.0406	0.50
1290:	47	4	43	1	47	46	4	42	1	46	122433.4915	0.0072	0.020		
1291:	71	5	67	1	71	71	4	68	1	71	122448.7988	0.0413	0.040		
1292:	47	24	23	1	47	46	24	22	1	46	122459.0756	-0.0390	0.020	-0.0390	0.50
1293:	47	24	24	1	47	46	24	23	1	46	122459.0756	-0.0390	0.020	-0.0390	0.50
1294:	42	5	37	1	42	42	4	38	1	42	122524.2595	0.0562	0.040		
1295:	47	26	22	1	47	46	26	21	1	46	122527.7617	0.0157	0.020	0.0158	0.50
1296:	47	26	21	1	47	46	26	20	1	46	122527.7617	0.0157	0.020	0.0158	0.50
1297:	47	5	43	1	47	47	4	44	1	47	122533.1651	0.0803	0.040		
1298:	47	27	20	1	47	46	27	19	1	46	122564.1974	0.0148	0.020	0.0148	0.50
1299:	47	27	21	1	47	46	27	20	1	46	122564.1974	0.0148	0.020	0.0148	0.50
1300:	47	28	19	1	47	46	28	18	1	46	122602.0373	0.0258	0.020	0.0259	0.50
1301:	47	28	20	1	47	46	28	19	1	46	122602.0373	0.0258	0.020	0.0259	0.50
1302:	47	29	18	1	47	46	29	17	1	46	122641.2161	-0.0012	0.020	-0.0013	0.50
1303:	47	29	19	1	47	46	29	18	1	46	122641.2161	-0.0012	0.020	-0.0013	0.50
1304:	45	5	41	1	45	45	4	42	1	45	122657.1870	0.0128	0.060		
1305:	47	30	17	1	47	46	30	16	1	46	122681.8071	0.0213	0.020	0.0214	0.50
1306:	47	30	18	1	47	46	30	17	1	46	122681.8071	0.0213	0.020	0.0214	0.50
1307:	44	5	40	1	44	44	4	41	1	44	122717.3953	0.0359	0.040		
1308:	47	31	16	1	47	46	31	15	1	46	122723.7515	0.0494	0.040	0.0494	0.50
1309:	47	31	17	1	47	46	31	16	1	46	122723.7515	0.0494	0.040	0.0494	0.50
1310:	47	32	15	1	47	46	32	14	1	46	122766.9262	-0.0258	0.020	-0.0259	0.50
1311:	47	32	16	1	47	46	32	15	1	46	122766.9262	-0.0258	0.020	-0.0259	0.50
1312:	47	33	14	1	47	46	33	13	1	46	122811.5592	0.0380	0.020	0.0381	0.50
1313:	47	33	15	1	47	46	33	14	1	46	122811.5592	0.0380	0.020	0.0381	0.50
1314:	39	5	34	1	39	39	4	35	1	39	122814.8647	0.0249	0.040		
1315:	42	5	38	1	42	42	4	39	1	42	122832.5652	0.0440	0.040		
1316:	47	34	13	1	47	46	34	12	1	46	122857.4081	0.0135	0.020	0.0136	0.50
1317:	47	34	14	1	47	46	34	13	1	46	122857.4081	0.0135	0.020	0.0136	0.50
1318:	41	5	37	1	41	41	4	38	1	41	122887.0597	0.0316	0.040		
1319:	40	5	36	1	40	40	4	37	1	40	122939.3455	0.1078	0.060		
1320:	37	5	32	1	37	37	4	33	1	37	122965.0842	0.0929	0.040		
1321:	48	1	48	1	48	47	0	47	1	47	122989.2475	0.0447	0.040		
1322:	74	5	70	1	74	74	4	71	1	74	122990.9514	0.0944	0.060		
1323:	36	5	31	1	36	36	4	32	1	36	123029.1626	0.0313	0.040		
1324:	47	3	44	1	47	46	3	43	1	46	123072.4036	0.0282	0.020		
1325:	37	5	33	1	37	37	4	34	1	37	123080.9478	0.1048	0.060		
1326:	51	1	50	1	51	50	2	49	1	50	123090.2356	0.0558	0.060		
1327:	36	5	32	1	36	36	4	33	1	36	123122.8788	0.0971	0.040		
1328:	34	5	29	1	34	34	4	30	1	34	123138.6043	0.0335	0.040		
1329:	35	5	31	1	35	35	4	32	1	35	123162.0715	0.0397	0.040		
1330:	33	5	28	1	33	33	4	29	1	33	123185.0435	0.1049	0.060		
1331:	75	5	71	1	75	75	4	72	1	75	123219.9788	0.0212	0.040		
1332:	31	5	26	1	31	31	4	27	1	31	123263.4788	0.1563	0.090		
1333:	32	5	28	1	32	32	4	29	1	32	123263.9293	0.1469	0.090		
1334:	31	5	27	1	31	31	4	28	1	31	123292.5918	0.0843	0.040		
1335:	30	5	25	1	30	30	4	26	1	30	123296.2629	0.0965	0.040		
1336:	30	5	26	1	30	30	4	27	1	30	123318.8433	0.0951	0.040		
1337:	29	5	24	1	29	29	4	25	1	29	123325.3024	0.0283	0.040		
1338:	70	2	69	1	70	70	1	70	1	70	123342.6827	0.0497	0.040	0.0705	0.50
1339:	29	5	25	1	29	29	4	26	1	29	123342.6827	0.0913	0.040	0.0705	0.50
1340:	28	5	24	1	28	28	4	25	1	28	123364.2467	0.1124	0.060		
1341:	21	5	16	1	21	21	4	17	1	21	123458.7836	0.0634	0.040		
1342:	21	5	17	1	21	21	4	18	1	21	123460.1359	0.0436	0.040		
1343:	47	2	45	1	47	46	2	44	1	46	123462.2225	0.0197	0.020		
1344:	19	5	14	1	19	19	4	15	1	19	123472.9449	0.0163	0.060		
1345:	19	5	15	1	19	19	4	16	1	19	123473.6545	0.1020	0.060		
1346:	76	5	72	1	76	76	4	73	1	76	123475.2272	0.0793	0.060		
1347:	18	5	13	1	18	18	4	14	1	18	123478.1987	0.0351	0.040		
1348:	18	5	14	1	18	18	4	15	1	18	123478.6433	0.0723	0.040		
1349:	16	5	11	1	16	16	4	12	1	16	123485.7318	0.1272	0.040	0.0469	0.50
1350:	16	5	12	1	16	16	4	13	1	16	123485.7318	-0.0335	0.040	0.0469	0.50
1351:	14	5	9	1	14	14	4	10	1	14	123489.8565	0.0179	0.090	-0.0100	0.50
1352:	14	5	10	1	14	14	4	11	1	14	123489.8565	-0.0380	0.090	-0.0100	0.50
1353:	48	2	47	1	48	47	2	46	1	47	123878.8077	0.0342	0.020		
1354:	48	1	47	1	48	47	1	46	1	47	124609.1436	0.0038	0.020		
1355:	48	9	40	1	48	47	9	39	1	47	124760.0864	0.0096	0.020	0.0097	0.50
1356:	48	9	39	1	48	47	9	38	1	47	124760.0864	0.0096	0.020	0.0097	0.50
1357:	48	3	46	1	48	47	3	45	1	47	124760.7866	0.0181	0.020		
1358:	48	10	39	1	48	47	10	38	1	47	124761.9543	0.0078	0.020	0.0078	0.50
1359:	48	10	38	1	48	47	10	37	1	47	124761.9543	0.0078	0.020	0.0078	0.50
1360:	48	8	40	1	48	47	8	39	1	47	124764.4820	0.0151	0.020	0.0152	0.50
1361:	48	8	41	1	48	47	8	40	1	47	124764.4820	0.0152	0.020	0.0152	0.50
1362:	49	1	49	1	49	48	1	48	1	48	124765.0751	-0.0191	0.020		
1363:	48	11	38	1	48	47	11	37	1	47	124768.3782	0.0082	0.020	0.0082	0.50
1364:	48	11	37	1	48	47	11	36	1	47	124768.3782	0.0082	0.020	0.0082	0.50
1365:	48	7	42	1	48	47	7	41	1	47	124778.2672	0.0785	0.020	0.0034	0.25

1366:	48 7 41	1 48	47 7 40	1 47	124778.2672	0.0718	0.020	0.0034	0.25
1367:	48 12 37	1 48	47 12 36	1 47	124778.2672	-0.0682	0.020	0.0034	0.25
1368:	48 12 36	1 48	47 12 35	1 47	124778.2672	-0.0682	0.020	0.0034	0.25
1369:	48 13 36	1 48	47 13 35	1 47	124791.2401	0.0295	0.020	0.0295	0.50
1370:	48 13 35	1 48	47 13 34	1 47	124791.2401	0.0295	0.020	0.0295	0.50
1371:	48 14 35	1 48	47 14 34	1 47	124806.5908	0.0081	0.020	0.0082	0.50
1372:	48 14 34	1 48	47 14 33	1 47	124806.5908	0.0081	0.020	0.0082	0.50
1373:	48 6 43	1 48	47 6 42	1 47	124807.1576	-0.0184	0.020		
1374:	48 6 42	1 48	47 6 41	1 47	124807.4810	0.0202	0.020		
1375:	48 15 33	1 48	47 15 32	1 47	124824.1709	-0.0015	0.020	-0.0015	0.50
1376:	48 15 34	1 48	47 15 33	1 47	124824.1709	-0.0015	0.020	-0.0015	0.50
1377:	49 0 49	1 49	48 0 48	1 48	124834.2019	0.0140	0.020		
1378:	48 16 33	1 48	47 16 32	1 47	124843.7807	-0.0044	0.020	-0.0044	0.50
1379:	48 16 32	1 48	47 16 31	1 47	124843.7807	-0.0044	0.020	-0.0044	0.50
1380:	48 5 44	1 48	47 5 43	1 47	124861.5053	-0.0056	0.020		
1381:	48 17 31	1 48	47 17 30	1 47	124865.2827	0.0017	0.020	0.0017	0.50
1382:	48 17 32	1 48	47 17 31	1 47	124865.2827	0.0017	0.020	0.0017	0.50
1383:	48 5 43	1 48	47 5 42	1 47	124869.5461	0.0169	0.020		
1384:	48 18 30	1 48	47 18 29	1 47	124888.5389	-0.0183	0.020	-0.0183	0.50
1385:	48 18 31	1 48	47 18 30	1 47	124888.5389	-0.0183	0.020	-0.0183	0.50
1386:	48 19 29	1 48	47 19 28	1 47	124913.5194	-0.0170	0.020	-0.0170	0.50
1387:	48 19 30	1 48	47 19 29	1 47	124913.5194	-0.0170	0.020	-0.0170	0.50
1388:	48 4 45	1 48	47 4 44	1 47	124924.7056	0.0200	0.020		
1389:	48 20 28	1 48	47 20 27	1 47	124940.1644	0.0054	0.020	0.0055	0.50
1390:	48 20 29	1 48	47 20 28	1 47	124940.1644	0.0054	0.020	0.0055	0.50
1391:	48 21 27	1 48	47 21 26	1 47	124968.3859	0.0080	0.020	0.0080	0.50
1392:	48 21 28	1 48	47 21 27	1 47	124968.3859	0.0080	0.020	0.0080	0.50
1393:	48 22 26	1 48	47 22 25	1 47	124998.1736	0.0182	0.020	0.0182	0.50
1394:	48 22 27	1 48	47 22 26	1 47	124998.1736	0.0182	0.020	0.0182	0.50
1395:	48 23 26	1 48	47 23 25	1 47	125029.4651	0.0046	0.020	0.0047	0.50
1396:	48 23 25	1 48	47 23 24	1 47	125029.4651	0.0046	0.020	0.0047	0.50
1397:	48 4 44	1 48	47 4 43	1 47	125057.9922	0.0207	0.020		
1398:	48 25 24	1 48	47 25 23	1 47	125096.5904	0.0386	0.020	0.0386	0.50
1399:	48 25 23	1 48	47 25 22	1 47	125096.5904	0.0386	0.020	0.0386	0.50
1400:	81 5 77	1 81	81 4 78	1 81	125179.4804	0.0172	0.040		
1401:	48 28 20	1 48	47 28 19	1 47	125208.1262	0.0379	0.020	0.0379	0.50
1402:	48 28 21	1 48	47 28 20	1 47	125208.1262	0.0379	0.020	0.0379	0.50
1403:	48 29 20	1 48	47 29 19	1 47	125248.0726	-0.0322	0.020	-0.0322	0.50
1404:	48 29 19	1 48	47 29 18	1 47	125248.0726	-0.0322	0.020	-0.0322	0.50
1405:	48 30 18	1 48	47 30 17	1 47	125289.5480	0.0337	0.020	0.0338	0.50
1406:	48 30 19	1 48	47 30 18	1 47	125289.5480	0.0337	0.020	0.0338	0.50
1407:	79 3 77	1 79	79 2 78	1 79	125301.6304	0.0594	0.040		
1408:	49 1 49	1 49	48 0 48	1 48	125440.9090	0.0645	0.040		
1409:	48 36 12	1 48	47 36 11	1 47	125566.3733	-0.0078	0.020	-0.0078	0.50
1410:	48 36 13	1 48	47 36 12	1 47	125566.3733	-0.0078	0.020	-0.0078	0.50
1411:	48 38 10	1 48	47 38 9	1 47	125669.0518	-0.0630	0.040	-0.0630	0.50
1412:	48 38 11	1 48	47 38 10	1 47	125669.0518	-0.0630	0.040	-0.0630	0.50
1413:	48 39 9	1 48	47 39 8	1 47	125722.3590	-0.0035	0.020	-0.0036	0.50
1414:	48 39 10	1 48	47 39 9	1 47	125722.3590	-0.0035	0.020	-0.0036	0.50
1415:	48 3 45	1 48	47 3 44	1 47	125733.2488	-0.0016	0.020		
1416:	86 4 83	1 86	86 3 84	1 86	125765.9041	-0.0480	0.060		
1417/	97 6 91	1 97	97 5 92	1 97	125845.2120	0.1441	0.040		
1418:	48 2 46	1 48	47 2 45	1 47	126069.6711	0.0085	0.020		
1419:	52 1 51	1 52	51 2 50	1 51	126245.0845	0.0063	0.040		
1420:	19 7 12	1 19	20 6 15	1 20	126297.3900	0.1388	0.060	0.1388	0.50
1421:	19 7 13	1 19	20 6 14	1 20	126297.3900	0.1388	0.060	0.1388	0.50
1422:	49 2 48	1 49	48 2 47	1 48	126428.6509	0.0124	0.020		
1423:	23 3 21	1 23	22 2 20	1 22	126492.8837	-0.0350	0.040		
1424:	50 0 50	1 50	49 1 49	1 49	126748.4392	-0.0198	0.020		
1425:	85 5 81	1 85	85 4 82	1 85	127113.5769	0.0585	0.060		
1426:	49 1 48	1 49	48 1 47	1 48	127129.9917	0.0338	0.020		
1427:	12 4 8	1 12	11 3 9	1 11	127249.9890	0.0891	0.060		
1428:	50 1 50	1 50	49 1 49	1 49	127292.6850	0.0146	0.020		
1429:	49 3 47	1 49	48 3 46	1 48	127347.7334	0.0656	0.040		
1430:	50 0 50	1 50	49 0 49	1 49	127355.1352	0.0195	0.020		
1431:	49 9 40	1 49	48 9 39	1 48	127359.1307	0.0314	0.020	0.0314	0.50
1432:	49 9 41	1 49	48 9 40	1 48	127359.1307	0.0314	0.020	0.0314	0.50
1433:	49 10 40	1 49	48 10 39	1 48	127360.4775	-0.0233	0.020	-0.0233	0.50
1434:	49 10 39	1 49	48 10 38	1 48	127360.4775	-0.0233	0.020	-0.0233	0.50
1435:	49 8 42	1 49	48 8 41	1 48	127364.3079	0.0122	0.020	0.0122	0.50
1436:	49 8 41	1 49	48 8 40	1 48	127364.3079	0.0121	0.020	0.0122	0.50
1437:	49 11 38	1 49	48 11 37	1 48	127366.6725	-0.0119	0.020	-0.0120	0.50
1438:	49 11 39	1 49	48 11 38	1 48	127366.6725	-0.0119	0.020	-0.0120	0.50
1439:	49 12 38	1 49	48 12 37	1 48	127376.6529	0.0790	0.040	0.0790	0.50
1440:	49 12 37	1 49	48 12 36	1 48	127376.6529	0.0790	0.040	0.0790	0.50
1441:	49 7 42	1 49	48 7 41	1 48	127379.3628	-0.0073	0.020	-0.0030	0.50
1442:	49 7 43	1 49	48 7 42	1 48	127379.3628	0.0014	0.020	-0.0030	0.50
1443:	49 13 37	1 49	48 13 36	1 48	127389.4671	-0.0291	0.020	-0.0292	0.50
1444:	49 13 36	1 49	48 13 35	1 48	127389.4671	-0.0291	0.020	-0.0292	0.50
1445:	49 14 35	1 49	48 14 34	1 48	127405.0021	-0.0106	0.020	-0.0107	0.50
1446:	49 14 36	1 49	48 14 35	1 48	127405.0021	-0.0106	0.020	-0.0107	0.50
1447:	49 15 34	1 49	48 15 33	1 48	127422.8290	0.0024	0.020	0.0024	0.50
1448:	49 15 35	1 49	48 15 34	1 48	127422.8290	0.0024	0.020	0.0024	0.50

1449:	49 16 33	1 49	48 16 32	1 48	127442.7557	0.0250	0.020	0.0250	0.50
1450:	49 16 34	1 49	48 16 33	1 48	127442.7557	0.0250	0.020	0.0250	0.50
1451:	49 17 32	1 49	48 17 31	1 48	127464.6110	0.0345	0.020	0.0345	0.50
1452:	49 17 33	1 49	48 17 32	1 48	127464.6110	0.0345	0.020	0.0345	0.50
1453:	49 5 45	1 49	48 5 44	1 48	127468.3636	-0.0026	0.020		
1454:	49 5 44	1 49	48 5 43	1 48	127478.0247	0.0410	0.020		
1455:	49 18 32	1 49	48 18 31	1 48	127488.2650	0.0102	0.020	0.0102	0.50
1456:	49 18 31	1 49	48 18 30	1 48	127488.2650	0.0102	0.020	0.0102	0.50
1457:	49 19 30	1 49	48 19 29	1 48	127513.6775	-0.0059	0.020	-0.0060	0.50
1458:	49 19 31	1 49	48 19 30	1 48	127513.6775	-0.0059	0.020	-0.0060	0.50
1459:	49 4 46	1 49	48 4 45	1 48	127531.7153	-0.0144	0.020		
1460:	49 20 30	1 49	48 20 29	1 48	127540.8381	0.0388	0.020	0.0388	0.50
1461:	49 20 29	1 49	48 20 28	1 48	127540.8381	0.0388	0.020	0.0388	0.50
1462:	49 21 28	1 49	48 21 27	1 48	127569.5245	-0.0279	0.020	-0.0279	0.50
1463:	49 21 29	1 49	48 21 28	1 48	127569.5245	-0.0279	0.020	-0.0279	0.50
1464:	49 22 28	1 49	48 22 27	1 48	127599.8900	-0.0129	0.020	-0.0130	0.50
1465:	49 22 27	1 49	48 22 26	1 48	127599.8900	-0.0129	0.020	-0.0130	0.50
1466:	49 23 27	1 49	48 23 26	1 48	127631.8628	0.0447	0.040	0.0447	0.50
1467:	49 23 26	1 49	48 23 25	1 48	127631.8628	0.0447	0.040	0.0447	0.50
1468:	49 25 24	1 49	48 25 23	1 48	127700.2361	0.0009	0.020	0.0010	0.50
1469:	49 25 25	1 49	48 25 24	1 48	127700.2361	0.0009	0.020	0.0010	0.50
1470:	49 26 24	1 49	48 26 23	1 48	127736.6658	-0.0265	0.020	-0.0266	0.50
1471:	49 26 23	1 49	48 26 22	1 48	127736.6658	-0.0265	0.020	-0.0266	0.50
1472:	49 27 22	1 49	48 27 21	1 48	127774.6299	0.0070	0.020	0.0071	0.50
1473:	49 27 23	1 49	48 27 22	1 48	127774.6299	0.0070	0.020	0.0071	0.50
1474:	49 29 20	1 49	48 29 19	1 48	127854.8165	-0.0181	0.020	-0.0181	0.50
1475:	49 29 21	1 49	48 29 20	1 48	127854.8165	-0.0181	0.020	-0.0181	0.50
1476:	49 33 16	1 49	48 33 15	1 48	128032.2308	0.0125	0.020	0.0126	0.50
1477:	49 33 17	1 49	48 33 16	1 48	128032.2308	0.0125	0.020	0.0126	0.50
1478:	49 2 47	1 49	48 2 46	1 48	128673.2985	0.0198	0.020		
1479:	50 2 49	1 50	49 2 48	1 49	128977.1104	0.0232	0.020		
1480:	51 1 51	1 51	50 1 50	1 50	129819.7637	-0.0817	0.060		
1481:	51 0 51	1 51	50 0 50	1 50	129876.1735	-0.0441	0.040		
1482:	50 9 41	1 50	49 9 40	1 49	129958.1946	0.0830	0.040	0.0830	0.50
1483:	50 9 42	1 50	49 9 41	1 49	129958.1946	0.0830	0.040	0.0830	0.50
1484:	50 8 43	1 50	49 8 42	1 49	129964.2135	0.0549	0.040	0.0548	0.50
1485:	50 8 42	1 50	49 8 41	1 49	129964.2135	0.0547	0.040	0.0548	0.50
1486:	50 13 37	1 50	49 13 36	1 49	129987.6814	-0.0052	0.020	-0.0053	0.50
1487:	50 13 38	1 50	49 13 37	1 49	129987.6814	-0.0052	0.020	-0.0053	0.50
1488:	50 14 36	1 50	49 14 35	1 49	130003.3608	0.0238	0.020	0.0238	0.50
1489:	50 14 37	1 50	49 14 36	1 49	130003.3608	0.0238	0.020	0.0238	0.50
1490:	50 17 33	1 50	49 17 32	1 49	130063.6829	-0.0612	0.040	-0.0613	0.50
1491:	50 17 34	1 50	49 17 33	1 49	130063.6829	-0.0612	0.040	-0.0613	0.50
1492:	50 5 46	1 50	49 5 45	1 49	130075.4160	-0.0828	0.060		
1493:	50 20 30	1 50	49 20 29	1 49	130141.3044	0.0057	0.020	0.0057	0.50
1494:	50 20 31	1 50	49 20 30	1 49	130141.3044	0.0057	0.020	0.0057	0.50
1495:	50 25 25	1 50	49 25 24	1 49	130303.7995	0.0350	0.020	0.0351	0.50
1496:	50 25 26	1 50	49 25 25	1 49	130303.7995	0.0350	0.020	0.0351	0.50
1497:	50 27 23	1 50	49 27 22	1 49	130379.5988	-0.0091	0.020	-0.0092	0.50
1498:	50 27 24	1 50	49 27 23	1 49	130379.5988	-0.0091	0.020	-0.0092	0.50
1499:	77 12 65	1 77	76 12 64	1 76	200087.6391	-0.0371	0.060	-0.0372	0.50
1500:	77 12 66	1 77	76 12 65	1 76	200087.6391	-0.0371	0.060	-0.0372	0.50
1501:	77 11 67	1 77	76 11 66	1 76	200088.2111	0.0071	0.025	0.0071	0.50
1502:	77 11 66	1 77	76 11 65	1 76	200088.2111	0.0071	0.025	0.0071	0.50
1503:	77 13 64	1 77	76 13 63	1 76	200095.4491	-0.0468	0.060	-0.0469	0.50
1504:	77 13 65	1 77	76 13 64	1 76	200095.4491	-0.0468	0.060	-0.0469	0.50
1505:	77 10 68	1 77	76 10 67	1 76	200099.7683	0.0528	0.060	0.0528	0.50
1506:	77 10 67	1 77	76 10 66	1 76	200099.7683	0.0527	0.060	0.0528	0.50
1507:	77 14 63	1 77	76 14 62	1 76	200109.9253	-0.0342	0.060	-0.0343	0.50
1508:	77 14 64	1 77	76 14 63	1 76	200109.9253	-0.0342	0.060	-0.0343	0.50
1509:	77 9 69	1 77	76 9 68	1 76	200126.4739	-0.0146	0.025	-0.0167	0.50
1510:	77 9 68	1 77	76 9 67	1 76	200126.4739	-0.0188	0.025	-0.0167	0.50
1511:	77 15 62	1 77	76 15 61	1 76	200129.9187	-0.0045	0.025	-0.0046	0.50
1512:	77 15 63	1 77	76 15 62	1 76	200129.9187	-0.0045	0.025	-0.0046	0.50
1513:	77 16 61	1 77	76 16 60	1 76	200154.5948	0.0008	0.025	0.0008	0.50
1514:	77 16 62	1 77	76 16 61	1 76	200154.5948	0.0008	0.025	0.0008	0.50
1515:	77 8 70	1 77	76 8 69	1 76	200175.9001	0.0385	0.025	-0.0256	0.50
1516:	77 8 69	1 77	76 8 68	1 76	200175.9001	-0.0897	0.025	-0.0256	0.50
1517:	77 17 60	1 77	76 17 59	1 76	200183.3852	-0.0214	0.025	-0.0215	0.50
1518:	77 17 61	1 77	76 17 60	1 76	200183.3852	-0.0214	0.025	-0.0215	0.50
1519:	77 18 59	1 77	76 18 58	1 76	200215.9217	-0.0274	0.025	-0.0274	0.50
1520:	77 18 60	1 77	76 18 59	1 76	200215.9217	-0.0274	0.025	-0.0274	0.50
1521:	77 4 74	1 77	76 4 73	1 76	200218.3689	0.0989	0.060		
1522:	77 19 58	1 77	76 19 57	1 76	200251.9445	0.0305	0.060	0.0306	0.50
1523:	77 19 59	1 77	76 19 58	1 76	200251.9445	0.0305	0.060	0.0306	0.50
1524:	77 7 71	1 77	76 7 70	1 76	200260.5556	0.0010	0.025		
1525:	77 7 70	1 77	76 7 69	1 76	200263.3869	-0.0399	0.060		
1526:	77 20 57	1 77	76 20 56	1 76	200291.0887	0.0212	0.025	0.0212	0.50
1527:	77 20 58	1 77	76 20 57	1 76	200291.0887	0.0212	0.025	0.0212	0.50
1528:	77 21 56	1 77	76 21 55	1 76	200333.2749	0.0461	0.060	0.0462	0.50
1529:	77 21 57	1 77	76 21 56	1 76	200333.2749	0.0461	0.060	0.0462	0.50
1530:	77 22 55	1 77	76 22 54	1 76	200378.2409	-0.0143	0.025	-0.0143	0.50
1531:	77 22 56	1 77	76 22 55	1 76	200378.2409	-0.0143	0.025	-0.0143	0.50

1532:	77 6 72 1 77	76 6 71 1 76	200392.8318	0.0459	0.060		
1533:	77 23 54 1 77	76 23 53 1 76	200426.0834	0.0508	0.060	0.0508	0.50
1534:	77 23 55 1 77	76 23 54 1 76	200426.0834	0.0508	0.060	0.0508	0.50
1535:	79 0 79 1 79	78 1 78 1 78	200449.0170	-0.0401	0.060		
1536:	79 1 79 1 79	78 1 78 1 78	200467.8463	-0.0119	0.025		
1537:	79 0 79 1 79	78 0 78 1 78	200470.2292	-0.0396	0.060		
1538:	77 24 53 1 77	76 24 52 1 76	200476.5636	0.0958	0.060	0.0959	0.50
1539:	77 24 54 1 77	76 24 53 1 76	200476.5636	0.0958	0.060	0.0959	0.50
1540:	79 1 79 1 79	78 0 78 1 78	200488.9946	-0.0753	0.060		
1541:	77 5 73 1 77	76 5 72 1 76	200500.6416	-0.0015	0.025		
1542:	77 25 52 1 77	76 25 51 1 76	200529.4947	0.0113	0.025	0.0114	0.50
1543:	77 25 53 1 77	76 25 52 1 76	200529.4947	0.0113	0.025	0.0114	0.50
1544:	77 26 51 1 77	76 26 50 1 76	200585.0499	0.0357	0.060	0.0358	0.50
1545:	77 26 52 1 77	76 26 51 1 76	200585.0499	0.0357	0.060	0.0358	0.50
1546:	77 27 50 1 77	76 27 49 1 76	200642.9330	-0.0711	0.060	-0.0711	0.50
1547:	77 27 51 1 77	76 27 50 1 76	200642.9330	-0.0711	0.060	-0.0711	0.50
1548:	77 28 49 1 77	76 28 48 1 76	200703.4305	0.0263	0.025	0.0264	0.50
1549:	77 28 50 1 77	76 28 49 1 76	200703.4305	0.0263	0.025	0.0264	0.50
1550:	77 29 48 1 77	76 29 47 1 76	200766.2133	0.0429	0.060	0.0429	0.50
1551:	77 29 49 1 77	76 29 48 1 76	200766.2133	0.0429	0.060	0.0429	0.50
1552:	77 30 47 1 77	76 30 46 1 76	200831.2662	0.0030	0.025	0.0031	0.50
1553:	77 30 48 1 77	76 30 47 1 76	200831.2662	0.0030	0.025	0.0031	0.50
1554:	77 31 46 1 77	76 31 45 1 76	200898.6475	0.0020	0.025	0.0020	0.50
1555:	77 31 47 1 77	76 31 46 1 76	200898.6475	0.0020	0.025	0.0020	0.50
1556:	77 5 72 1 77	76 5 71 1 76	200931.0774	0.0009	0.025		
1557:	78 3 76 1 78	77 3 75 1 77	201662.4442	0.0165	0.025		
1558:	79 1 78 1 79	78 2 77 1 78	201772.7286	-0.0416	0.060		
1559:	77 3 74 1 77	76 3 73 1 76	201989.1199	-0.0123	0.025		
1560:	77 4 73 1 77	76 4 72 1 76	202028.2489	-0.0017	0.025		
1561:	78 2 76 1 78	77 2 75 1 77	202363.8291	0.0386	0.060		
1562:	79 2 78 1 79	78 2 77 1 78	202416.7713	-0.0530	0.060		
1563:	79 1 78 1 79	78 1 77 1 78	202486.9429	-0.0494	0.060		
1564:	78 12 66 1 78	77 12 65 1 77	202682.8121	-0.0198	0.025	-0.0198	0.50
1565:	78 12 67 1 78	77 12 66 1 77	202682.8121	-0.0198	0.025	-0.0198	0.50
1566:	78 11 67 1 78	77 11 66 1 77	202684.1363	0.0595	0.060	0.0595	0.50
1567:	78 11 68 1 78	77 11 67 1 77	202684.1363	0.0595	0.060	0.0595	0.50
1568:	78 13 65 1 78	77 13 64 1 77	202690.1944	-0.0084	0.025	-0.0084	0.50
1569:	78 13 66 1 78	77 13 65 1 77	202690.1944	-0.0084	0.025	-0.0084	0.50
1570:	78 10 69 1 78	77 10 68 1 77	202696.7194	0.0389	0.060	0.0389	0.50
1571:	78 10 68 1 78	77 10 67 1 77	202696.7194	0.0388	0.060	0.0389	0.50
1572:	78 15 63 1 78	77 15 62 1 77	202724.2165	-0.0714	0.060	-0.0715	0.50
1573:	78 15 64 1 78	77 15 63 1 77	202724.2165	-0.0714	0.060	-0.0715	0.50
1574:	78 9 70 1 78	77 9 69 1 77	202725.0862	-0.0115	0.025	-0.0142	0.50
1575:	78 9 69 1 78	77 9 68 1 77	202725.0862	-0.0168	0.025	-0.0142	0.50
1576:	78 16 62 1 78	77 16 61 1 77	202748.9987	0.0097	0.025	0.0097	0.50
1577:	78 16 63 1 78	77 16 62 1 77	202748.9987	0.0097	0.025	0.0097	0.50
1578:	78 8 71 1 78	77 8 70 1 77	202776.9358	-0.0378	0.060		
1579:	78 17 61 1 78	77 17 60 1 77	202777.9709	0.0370	0.060	0.0371	0.50
1580:	78 17 62 1 78	77 17 61 1 77	202777.9709	0.0370	0.060	0.0371	0.50
1581:	78 4 75 1 78	77 4 74 1 77	202794.6253	0.0327	0.025		
1582:	78 18 60 1 78	77 18 59 1 77	202810.6621	-0.0321	0.025	-0.0321	0.50
1583:	78 18 61 1 78	77 18 60 1 77	202810.6621	-0.0321	0.025	-0.0321	0.50
1584:	78 7 72 1 78	77 7 71 1 77	202865.5039	0.0439	0.060		
1585:	78 7 71 1 78	77 7 70 1 77	202868.8479	0.0101	0.025		
1586:	78 20 58 1 78	77 20 57 1 77	202886.4426	-0.0186	0.025	-0.0187	0.50
1587:	78 20 59 1 78	77 20 58 1 77	202886.4426	-0.0186	0.025	-0.0187	0.50
1588:	78 21 57 1 78	77 21 56 1 77	202929.0253	-0.0122	0.025	-0.0122	0.50
1589:	78 21 58 1 78	77 21 57 1 77	202929.0253	-0.0122	0.025	-0.0122	0.50
1590:	78 22 56 1 78	77 22 55 1 77	202974.4751	-0.0566	0.060	-0.0566	0.50
1591:	78 22 57 1 78	77 22 56 1 77	202974.4751	-0.0566	0.060	-0.0566	0.50
1592:	80 1 80 1 80	79 1 79 1 79	202987.6679	-0.1310	0.080		
1593:	80 0 80 1 80	79 0 79 1 79	202989.9322	-0.0070	0.080		
1594:	78 6 73 1 78	77 6 72 1 77	203001.9264	-0.0043	0.025		
1595:	78 6 72 1 78	77 6 71 1 77	203053.1807	0.0045	0.025		
1596:	78 24 54 1 78	77 24 53 1 77	203073.8116	-0.0103	0.025	-0.0103	0.50
1597:	78 24 55 1 78	77 24 54 1 77	203073.8116	-0.0103	0.025	-0.0103	0.50
1598:	78 5 74 1 78	77 5 73 1 77	203105.2278	0.0190	0.025		
1599:	78 25 53 1 78	77 25 52 1 77	203127.4438	0.0024	0.025	0.0025	0.50
1600:	78 25 54 1 78	77 25 53 1 77	203127.4438	0.0024	0.025	0.0025	0.50
1601:	79 2 78 1 79	78 1 77 1 78	203131.1228	0.0763	0.090		
1602:	87 8 80 1 87	87 7 81 1 87	203180.1906	-0.0713	0.040		
1603:	78 26 52 1 78	77 26 51 1 77	203183.5544	-0.0616	0.060	-0.0617	0.50
1604:	78 26 53 1 78	77 26 52 1 77	203183.5544	-0.0616	0.060	-0.0617	0.50
1605:	78 28 50 1 78	77 28 49 1 77	203303.4699	0.0628	0.060	0.0629	0.50
1606:	78 28 51 1 78	77 28 50 1 77	203303.4699	0.0628	0.060	0.0629	0.50
1607:	86 8 79 1 86	86 7 80 1 86	203306.7707	-0.0265	0.060		
1608:	78 29 49 1 78	77 29 48 1 77	203366.9281	0.0006	0.025	0.0007	0.50
1609:	78 29 50 1 78	77 29 49 1 77	203366.9281	0.0006	0.025	0.0007	0.50
1610/	85 8 78 1 85	85 7 79 1 85	203428.7133	-0.0830	0.025		
1611:	78 30 48 1 78	77 30 47 1 77	203432.8407	0.0323	0.025	0.0323	0.50
1612:	78 30 49 1 78	77 30 48 1 77	203432.8407	0.0323	0.025	0.0323	0.50
1613:	84 8 76 1 84	84 7 77 1 84	203491.6344	-0.1546	0.060		
1614:	78 31 47 1 78	77 31 46 1 77	203501.0119	-0.0001	0.025	-0.0002	0.50

```
1615: 78 31 48  1 78   77 31 47  1 77       203501.0119  -0.0001  0.025  -0.0002 0.50
1616: 78  5 73  1 78   77  5 72  1 77       203579.6578  -0.0186  0.025
1617: 78 33 45  1 78   77 33 44  1 77       203644.2893   0.0426  0.060   0.0427 0.50
1618: 78 33 46  1 78   77 33 45  1 77       203644.2893   0.0426  0.060   0.0427 0.50
1619: 31  5 27  1 31   30  4 26  1 30       203883.9542   0.0380  0.040
1620: 79  8 71  1 79   79  7 72  1 79       204045.6854   0.0339  0.060
1621: 79  8 72  1 79   79  7 73  1 79       204069.4719  -0.1409  0.060
1622: 79  3 77  1 79   78  3 76  1 78       204200.7986   0.0032  0.025
1623/ 77  8 69  1 77   77  7 70  1 77       204233.3202  -0.1891  0.060
1624: 74  3 72  1 74   73  2 71  1 73       204299.7424   0.0443  0.090
1625: 76  8 68  1 76   76  7 69  1 76       204320.9926   0.0463  0.060
1626: 80  1 79  1 80   79  2 78  1 79       204358.0638   0.0145  0.040
1627: 75  8 67  1 75   75  7 68  1 75       204404.2827   0.0119  0.060
1628: 74  8 66  1 74   74  7 67  1 74       204483.6517   0.0166  0.060
1629: 74  8 67  1 74   74  7 68  1 74       204493.5633   0.0151  0.060
1630: 78  3 75  1 78   77  3 74  1 77       204557.1535   0.0313  0.025
1631: 78  4 74  1 78   77  4 73  1 77       204689.7668   0.0122  0.025
1632: 69  8 62  1 69   69  7 63  1 69       204829.7573  -0.0145  0.025
1633: 79  2 77  1 79   78  2 76  1 78       204861.8141   0.0812  0.060
1634: 80  1 79  1 80   79  1 78  1 79       205002.1439   0.0405  0.060
1635: 65  8 57  1 65   65  7 58  1 65       205041.8838   0.0209  0.060
1636: 61  8 53  1 61   61  7 54  1 61       205213.8839  -0.0683  0.060
1637: 79 12 67  1 79   78 12 66  1 78       205277.8683   0.0136  0.025   0.0137 0.50
1638: 79 12 68  1 79   78 12 67  1 78       205277.8683   0.0136  0.025   0.0137 0.50
1639: 79 11 69  1 79   78 11 68  1 78       205279.8360  -0.0078  0.025  -0.0078 0.50
1640: 79 11 68  1 79   78 11 67  1 78       205279.8360  -0.0078  0.025  -0.0078 0.50
1641: 79 13 67  1 79   78 13 66  1 78       205284.7236  -0.0321  0.025  -0.0322 0.50
1642: 79 13 66  1 79   78 13 65  1 78       205284.7236  -0.0321  0.025  -0.0322 0.50
1643: 79 10 70  1 79   78 10 69  1 78       205293.5940   0.0176  0.025   0.0176 0.50
1644: 79 10 69  1 79   78 10 68  1 78       205293.5940   0.0174  0.025   0.0176 0.50
1645: 79 14 65  1 79   78 14 64  1 78       205298.7021  -0.0036  0.025  -0.0037 0.50
1646: 79 14 66  1 79   78 14 65  1 78       205298.7021  -0.0036  0.025  -0.0037 0.50
1647: 79 15 65  1 79   78 15 64  1 78       205318.4164  -0.0526  0.060  -0.0526 0.50
1648: 79 15 64  1 79   78 15 63  1 78       205318.4164  -0.0526  0.060  -0.0526 0.50
1649: 79  9 71  1 79   78  9 70  1 78       205323.6726  -0.0167  0.025  -0.0200 0.50
1650: 79  9 70  1 79   78  9 69  1 78       205323.6726  -0.0232  0.025  -0.0200 0.50
1651: 82  2 80  1 82   81  3 79  1 81       205338.0031   0.0712  0.090
1652: 79 16 64  1 79   78 16 63  1 78       205343.2127   0.0232  0.025   0.0232 0.50
1653: 79 16 63  1 79   78 16 62  1 78       205343.2127   0.0232  0.025   0.0232 0.50
1654: 79  4 76  1 79   78  4 75  1 78       205369.0681  -0.0436  0.060
1655: 79 17 62  1 79   78 17 61  1 78       205372.2011  -0.0562  0.060  -0.0562 0.50
1656: 79 17 63  1 79   78 17 62  1 78       205372.2011  -0.0562  0.060  -0.0562 0.50
1657: 56  8 48  1 56   56  7 49  1 56       205376.7003  -0.0528  0.025
1658: 79  8 71  1 79   78  8 70  1 78       205378.2497  -0.0803  0.025   0.0131 0.50
1659: 79  8 72  1 79   78  8 71  1 78       205378.2497   0.1065  0.025   0.0131 0.50
1660: 55  8 47  1 55   55  7 48  1 55       205403.1938  -0.0124  0.025
1661: 79 18 61  1 79   78 18 60  1 78       205405.1736  -0.0543  0.060  -0.0543 0.50
1662: 79 18 62  1 79   78 18 61  1 78       205405.1736  -0.0543  0.060  -0.0543 0.50
1663: 79 19 60  1 79   78 19 59  1 78       205441.7873   0.0174  0.025   0.0174 0.50
1664: 79 19 61  1 79   78 19 60  1 78       205441.7873   0.0174  0.025   0.0174 0.50
1665: 79  7 73  1 79   78  7 72  1 78       205470.5506   0.0346  0.060
1666: 79  7 72  1 79   78  7 71  1 78       205474.5196   0.0406  0.060
1667: 79 20 59  1 79   78 20 58  1 78       205481.6766   0.0451  0.060   0.0452 0.50
1668: 79 20 60  1 79   78 20 59  1 78       205481.6766   0.0451  0.060   0.0452 0.50
1669: 51  8 43  1 51   51  7 44  1 51       205491.2532  -0.0527  0.060  -0.0830 0.50
1670: 51  8 44  1 51   51  7 45  1 51       205491.2532  -0.1132  0.060  -0.0830 0.50
1671: 81  0 81  1 81   80  1 80  1 80       205492.8757   0.1035  0.100
1672: 81  1 81  1 81   80  1 80  1 80       205507.4767  -0.0567  0.060
1673: 50  8 42  1 50   50  7 43  1 50       205509.3421   0.0685  0.060   0.0001 0.33
1674: 50  8 43  1 50   50  7 44  1 50       205509.3421   0.0225  0.060   0.0001 0.33
1675: 81  0 81  1 81   80  0 80  1 80       205509.3421  -0.0909  0.060   0.0001 0.33
1676: 79 21 59  1 79   78 21 58  1 78       205524.6469   0.0292  0.060   0.0292 0.50
1677: 79 21 58  1 79   78 21 57  1 78       205524.6469   0.0292  0.060   0.0292 0.50
1678: 49  8 42  1 49   49  7 43  1 49       205525.8030   0.0078  0.060   0.0252 0.50
1679: 49  8 41  1 49   49  7 42  1 49       205525.8030   0.0426  0.060   0.0252 0.50
1680: 48  8 40  1 48   48  7 41  1 48       205540.8391   0.0043  0.025  -0.0087 0.50
1681: 48  8 41  1 48   48  7 42  1 48       205540.8391  -0.0218  0.025  -0.0087 0.50
1682: 47  8 39  1 47   47  7 40  1 47       205554.5748   0.0115  0.090   0.0018 0.50
1683: 47  8 40  1 47   47  7 41  1 47       205554.5748  -0.0080  0.090   0.0018 0.50
1684: 46  8 38  1 46   46  7 39  1 46       205567.0291   0.0180  0.060   0.0108 0.50
1685: 46  8 39  1 46   46  7 40  1 46       205567.0291   0.0035  0.060   0.0108 0.50
1686: 79 22 57  1 79   78 22 56  1 78       205570.5054  -0.0698  0.060  -0.0699 0.50
1687: 79 22 58  1 79   78 22 57  1 78       205570.5054  -0.0698  0.060  -0.0699 0.50
1688: 80  2 79  1 80   79  1 78  1 79       205582.6155   0.0449  0.090
1689: 44  8 36  1 44   44  7 37  1 44       205588.2730  -0.0439  0.025  -0.0478 0.50
1690: 44  8 37  1 44   44  7 38  1 44       205588.2730  -0.0517  0.025  -0.0478 0.50
1691: 43  8 35  1 43   43  7 36  1 43       205597.3595   0.0618  0.060   0.0590 0.50
1692: 43  8 36  1 43   43  7 37  1 43       205597.3595   0.0561  0.060   0.0590 0.50
1693: 42  8 34  1 42   42  7 35  1 42       205605.2240  -0.0188  0.025  -0.0209 0.50
1694: 42  8 35  1 42   42  7 36  1 42       205605.2240  -0.0229  0.025  -0.0209 0.50
1695: 15  8  8  1 15   15  7  9  1 15       205606.2205  -0.0955  0.060  -0.0956 0.50
1696: 15  8  7  1 15   15  7  8  1 15       205606.2205  -0.0955  0.060  -0.0956 0.50
1697: 79  6 74  1 79   78  6 73  1 78       205611.0274  -0.1198  0.100
```

1698:	41	8	33	1	41	41	7	34	1	41	205612.2443	0.0341	0.060	0.0327	0.50
1699:	41	8	34	1	41	41	7	35	1	41	205612.2443	0.0311	0.060	0.0327	0.50
1700:	18	8	11	1	18	18	7	12	1	18	205614.4668	-0.0749	0.060	-0.0750	0.50
1701:	18	8	10	1	18	18	7	11	1	18	205614.4668	-0.0749	0.060	-0.0750	0.50
1702:	19	8	11	1	19	19	7	12	1	19	205617.3339	0.0238	0.060	0.0238	0.50
1703:	19	8	12	1	19	19	7	13	1	19	205617.3339	0.0238	0.060	0.0238	0.50
1704:	40	8	32	1	40	40	7	33	1	40	205618.2208	-0.0350	0.025	-0.0361	0.50
1705:	40	8	33	1	40	40	7	34	1	40	205618.2208	-0.0371	0.025	-0.0361	0.50
1706:	79	23	56	1	79	78	23	55	1	78	205619.3551	-0.0262	0.025	-0.0262	0.50
1707:	79	23	57	1	79	78	23	56	1	78	205619.3551	-0.0262	0.025	-0.0262	0.50
1708:	21	8	13	1	21	21	7	14	1	21	205622.8030	0.0666	0.060	0.0667	0.50
1709:	21	8	14	1	21	21	7	15	1	21	205622.8030	0.0666	0.060	0.0667	0.50
1710:	39	8	31	1	39	39	7	32	1	39	205623.4626	0.0279	0.060	0.0272	0.50
1711:	39	8	32	1	39	39	7	33	1	39	205623.4626	0.0264	0.060	0.0272	0.50
1712:	22	8	15	1	22	22	7	16	1	22	205625.3118	-0.0278	0.025	-0.0278	0.50
1713:	22	8	14	1	22	22	7	15	1	22	205625.3118	-0.0278	0.025	-0.0278	0.50
1714:	38	8	30	1	38	38	7	31	1	38	205627.7502	-0.0499	0.060	-0.0657	0.25
1715:	38	8	31	1	38	38	7	32	1	38	205627.7502	-0.0509	0.060	-0.0657	0.25
1716:	23	8	15	1	23	23	7	16	1	23	205627.7502	-0.0808	0.060	-0.0657	0.25
1717:	23	8	16	1	23	23	7	17	1	23	205627.7502	-0.0808	0.060	-0.0657	0.25
1718:	24	8	16	1	24	24	7	17	1	24	205630.2767	0.0966	0.060	0.0967	0.50
1719:	24	8	17	1	24	24	7	18	1	24	205630.2767	0.0966	0.060	0.0967	0.50
1720:	37	8	29	1	37	37	7	30	1	37	205631.4000	-0.0042	0.025	-0.0046	0.50
1721:	37	8	30	1	37	37	7	31	1	37	205631.4000	-0.0049	0.025	-0.0046	0.50
1722:	36	8	28	1	36	36	7	29	1	36	205634.2434	-0.0543	0.060	-0.0662	0.25
1723:	36	8	29	1	36	36	7	30	1	36	205634.2434	-0.0547	0.060	-0.0662	0.25
1724:	26	8	19	1	26	26	7	20	1	26	205634.2434	-0.0779	0.060	-0.0662	0.25
1725:	26	8	18	1	26	26	7	19	1	26	205634.2434	-0.0779	0.060	-0.0662	0.25
1726:	27	8	20	1	27	27	7	21	1	27	205635.9863	-0.0595	0.060	-0.0595	0.50
1727:	27	8	19	1	27	27	7	20	1	27	205635.9863	-0.0595	0.060	-0.0595	0.50
1728:	28	8	21	1	28	28	7	22	1	28	205637.4357	-0.0564	0.070	-0.0564	0.50
1729:	28	8	20	1	28	28	7	21	1	28	205637.4357	-0.0563	0.070	-0.0564	0.50
1730:	34	8	26	1	34	34	7	27	1	34	205638.0703	-0.0780	0.070	-0.0781	0.50
1731:	34	8	27	1	34	34	7	28	1	34	205638.0703	-0.0782	0.070	-0.0781	0.50
1732:	29	8	21	1	29	29	7	22	1	29	205638.5676	-0.0552	0.070	-0.0553	0.50
1733:	29	8	22	1	29	29	7	23	1	29	205638.5676	-0.0553	0.070	-0.0553	0.50
1734:	33	8	25	1	33	33	7	26	1	33	205639.1077	-0.0923	0.070	-0.0924	0.50
1735:	33	8	26	1	33	33	7	27	1	33	205639.1077	-0.0924	0.070	-0.0924	0.50
1736:	32	8	24	1	32	32	7	25	1	32	205639.6656	-0.0644	0.070	-0.0907	0.25
1737:	32	8	25	1	32	32	7	26	1	32	205639.6656	-0.0645	0.070	-0.0907	0.25
1738:	31	8	23	1	31	31	7	24	1	31	205639.6656	-0.1168	0.070	-0.0907	0.25
1739:	31	8	24	1	31	31	7	25	1	31	205639.6656	-0.1169	0.070	-0.0907	0.25
1740:	79	6	73	1	79	78	6	72	1	78	205669.6289	0.0661	0.060		
1741:	79	24	55	1	79	78	24	54	1	78	205670.9705	0.0347	0.060	0.0347	0.50
1742:	79	24	56	1	79	78	24	55	1	78	205670.9705	0.0347	0.060	0.0347	0.50
1743:	79	5	75	1	79	78	5	74	1	78	205709.0083	0.0143	0.025		
1744:	79	25	54	1	79	78	25	53	1	78	205725.2112	0.0554	0.060	0.0554	0.50
1745:	79	25	55	1	79	78	25	54	1	78	205725.2112	0.0554	0.060	0.0554	0.50
1746:	79	26	53	1	79	78	26	52	1	78	205781.9019	-0.0696	0.060	-0.0696	0.50
1747:	79	26	54	1	79	78	26	53	1	78	205781.9019	-0.0696	0.060	-0.0696	0.50
1748:	79	27	52	1	79	78	27	51	1	78	205841.2673	-0.0558	0.060	-0.0559	0.50
1749:	79	27	53	1	79	78	27	52	1	78	205841.2673	-0.0558	0.060	-0.0559	0.50
1750:	79	28	51	1	79	78	28	50	1	78	205903.1486	-0.0097	0.025	-0.0097	0.50
1751:	79	28	52	1	79	78	28	51	1	78	205903.1486	-0.0097	0.025	-0.0097	0.50
1752:	79	30	49	1	79	78	30	48	1	78	206034.0714	-0.0261	0.060	-0.0262	0.50
1753:	79	30	50	1	79	78	30	49	1	78	206034.0714	-0.0261	0.060	-0.0262	0.50
1754:	79	5	74	1	79	78	5	73	1	78	206230.7058	-0.0043	0.025		
1755:	80	3	78	1	80	79	3	77	1	79	206737.7809	-0.0136	0.025		
1756:	79	3	76	1	79	78	3	75	1	78	207119.8994	-0.0265	0.060		
1757:	80	2	78	1	80	79	2	77	1	79	207359.3582	0.0824	0.060		
1758:	81	2	80	1	81	80	2	79	1	80	207459.7671	-0.0124	0.025		
1759:	81	1	80	1	81	80	1	79	1	80	207517.4733	0.1142	0.060		
1760:	80	12	68	1	80	79	12	67	1	79	207872.7704	0.0278	0.060	0.0278	0.50
1761:	80	12	69	1	80	79	12	68	1	79	207872.7704	0.0278	0.060	0.0278	0.50
1762:	80	11	69	1	80	79	11	68	1	79	207875.5217	0.0180	0.025	0.0180	0.50
1763:	80	11	70	1	80	79	11	69	1	79	207875.5217	0.0180	0.025	0.0180	0.50
1764:	80	13	67	1	80	79	13	66	1	79	207879.1959	0.0430	0.060	0.0431	0.50
1765:	80	13	68	1	80	79	13	67	1	79	207879.1959	0.0430	0.060	0.0431	0.50
1766:	80	14	66	1	80	79	14	65	1	79	207892.8163	-0.0047	0.025	-0.0048	0.50
1767:	80	14	67	1	80	79	14	66	1	79	207892.8163	-0.0047	0.025	-0.0048	0.50
1768:	80	15	65	1	80	79	15	64	1	79	207912.5045	0.0404	0.060	0.0405	0.50
1769:	80	15	66	1	80	79	15	65	1	79	207912.5045	0.0404	0.060	0.0405	0.50
1770:	80	9	72	1	80	79	9	71	1	79	207922.3098	0.0466	0.060	0.0426	0.50
1771:	80	9	71	1	80	79	9	70	1	79	207922.3098	0.0385	0.060	0.0426	0.50
1772:	80	4	77	1	80	79	4	76	1	79	207941.8150	0.0101	0.025		
1773:	80	17	63	1	80	79	17	62	1	79	207966.3595	-0.0149	0.025	-0.0150	0.50
1774:	80	17	64	1	80	79	17	63	1	79	207966.3595	-0.0149	0.025	-0.0150	0.50
1775:	80	8	73	1	80	79	8	72	1	79	207979.4616	0.0904	0.025	-0.0219	0.50
1776:	80	8	72	1	80	79	8	71	1	79	207979.4616	-0.1342	0.025	-0.0219	0.50
1777:	80	18	62	1	80	79	18	61	1	79	207999.5070	-0.0405	0.060	-0.0406	0.50
1778:	80	18	63	1	80	79	18	62	1	79	207999.5070	-0.0405	0.060	-0.0406	0.50
1779:	82	1	82	1	82	81	1	81	1	81	208026.9929	-0.0677	0.060		
1780:	82	0	82	1	82	81	0	81	1	81	208028.7604	0.0141	0.025		

1781:	81 2 80	1 81	80 1 79	1 80	208040.2350	-0.0117	0.040		
1782:	82 1 82	1 82	81 0 81	1 81	208041.8837	0.0617	0.060		
1783:	80 7 74	1 80	79 7 73	1 79	208075.7060	-0.0158	0.025		
1784:	80 20 60	1 80	79 20 59	1 79	208076.6044	0.0292	0.060	0.0292	0.50
1785:	80 20 61	1 80	79 20 60	1 79	208076.6044	0.0292	0.060	0.0292	0.50
1786:	80 7 73	1 80	79 7 72	1 79	208080.3800	0.0191	0.025		
1787:	80 21 59	1 80	79 21 58	1 79	208119.9197	-0.0467	0.060	-0.0467	0.50
1788:	80 21 60	1 80	79 21 59	1 79	208119.9197	-0.0467	0.060	-0.0467	0.50
1789:	80 22 58	1 80	79 22 57	1 79	208166.2790	-0.1039	0.060	-0.1039	0.50
1790:	80 22 59	1 80	79 22 58	1 79	208166.2790	-0.1039	0.060	-0.1039	0.50
1791:	80 5 76	1 80	79 5 75	1 79	208312.0477	0.1130	0.060		
1792:	80 26 54	1 80	79 26 53	1 79	208380.2174	0.1399	0.060	0.1399	0.50
1793:	80 26 55	1 80	79 26 54	1 79	208380.2174	0.1399	0.060	0.1399	0.50
1794:	83 2 81	1 83	82 3 80	1 82	208381.3991	-0.0436	0.090		
1795:	43 4 39	1 43	42 3 40	1 42	208406.9943	0.0473	0.040		
1796:	80 28 52	1 80	79 28 51	1 79	208502.6017	-0.0532	0.060	-0.0532	0.50
1797:	80 28 53	1 80	79 28 52	1 79	208502.6017	-0.0532	0.060	-0.0532	0.50
1798:	82 1 81	1 82	81 2 80	1 81	209509.8842	0.0390	0.090		
1799:	80 3 77	1 80	79 3 76	1 79	209677.5402	0.0116	0.025		
1800:	81 2 79	1 81	80 2 78	1 80	209856.5993	-0.0170	0.025		
1801:	82 2 81	1 82	81 2 80	1 81	209980.6059	-0.0247	0.025		
1802:	80 4 76	1 80	79 4 75	1 79	210004.8254	-0.0205	0.025		
1803:	82 1 81	1 82	81 1 80	1 81	210032.7347	0.0018	0.025		
1804:	81 12 69	1 81	80 12 68	1 80	210467.5014	0.0074	0.025	0.0074	0.50
1805:	81 12 70	1 81	80 12 69	1 80	210467.5014	0.0074	0.025	0.0074	0.50
1806:	81 11 71	1 81	80 11 70	1 80	210471.0428	-0.0122	0.025	-0.0122	0.50
1807:	81 11 70	1 81	80 11 69	1 80	210471.0428	-0.0122	0.025	-0.0122	0.50
1808:	81 13 68	1 81	80 13 67	1 80	210473.3726	-0.0192	0.025	-0.0193	0.50
1809:	81 13 69	1 81	80 13 68	1 80	210473.3726	-0.0192	0.025	-0.0193	0.50
1810:	81 14 67	1 81	80 14 66	1 80	210486.9597	0.1982	0.025	0.0003	0.25
1811:	81 14 68	1 81	80 14 67	1 80	210486.9597	0.1982	0.025	0.0003	0.25
1812:	81 10 72	1 81	80 10 71	1 80	210486.9597	-0.1975	0.025	0.0003	0.25
1813:	81 10 71	1 81	80 10 70	1 80	210486.9597	-0.1978	0.025	0.0003	0.25
1814:	81 15 66	1 81	80 15 65	1 80	210506.2761	0.0055	0.025	0.0056	0.50
1815:	81 15 67	1 81	80 15 66	1 80	210506.2761	0.0055	0.025	0.0056	0.50
1816:	81 4 78	1 81	80 4 77	1 80	210512.6344	-0.0183	0.025		
1817:	81 9 73	1 81	80 9 72	1 80	210520.8137	-0.0055	0.025	-0.0105	0.50
1818:	81 9 72	1 81	80 9 71	1 80	210520.8137	-0.0154	0.025	-0.0105	0.50
1819:	81 16 65	1 81	80 16 64	1 80	210530.9222	-0.0743	0.060	-0.0743	0.50
1820:	81 16 66	1 81	80 16 65	1 80	210530.9222	-0.0743	0.060	-0.0743	0.50
1821:	83 1 83	1 83	82 1 82	1 82	210546.3304	-0.0490	0.060		
1822:	83 0 83	1 83	82 0 82	1 82	210547.9195	0.0447	0.060		
1823:	83 1 83	1 83	82 0 82	1 82	210559.5598	0.1046	0.060		
1824:	81 8 74	1 81	80 8 73	1 80	210580.7506	0.0919	0.060	-0.0428	0.50
1825:	81 8 73	1 81	80 8 72	1 80	210580.7506	-0.1774	0.060	-0.0428	0.50
1826:	81 7 75	1 81	80 7 74	1 80	210681.0633	-0.0135	0.025		
1827:	81 7 74	1 81	80 7 73	1 80	210686.4526	-0.0424	0.060		
1828:	81 23 58	1 81	80 23 57	1 80	210811.7440	-0.0266	0.025	-0.0266	0.50
1829:	81 23 59	1 81	80 23 58	1 80	210811.7440	-0.0266	0.025	-0.0266	0.50
1830:	81 6 76	1 81	80 6 75	1 80	210829.5816	-0.1201	0.060		
1831:	81 24 57	1 81	80 24 56	1 80	210864.3778	-0.0524	0.060	-0.0524	0.50
1832:	81 24 58	1 81	80 24 57	1 80	210864.3778	-0.0524	0.060	-0.0524	0.50
1833:	81 5 77	1 81	80 5 76	1 80	210913.9599	-0.0066	0.025		
1834:	81 25 56	1 81	80 25 55	1 80	210919.8299	-0.0118	0.025	-0.0119	0.50
1835:	81 25 57	1 81	80 25 56	1 80	210919.8299	-0.0118	0.025	-0.0119	0.50
1836:	81 27 54	1 81	80 27 53	1 80	211038.6302	-0.0030	0.025	-0.0031	0.50
1837:	81 27 55	1 81	80 27 54	1 80	211038.6302	-0.0030	0.025	-0.0031	0.50
1838:	81 33 48	1 81	80 33 47	1 80	211454.9650	-0.0124	0.025	-0.0125	0.50
1839:	81 33 49	1 81	80 33 48	1 80	211454.9650	-0.0124	0.025	-0.0125	0.50
1840:	81 5 76	1 81	80 5 75	1 80	211540.0384	-0.0424	0.060		
1841:	34 5 29	1 34	33 4 30	1 33	211629.4472	0.0476	0.040		
1842:	81 37 44	1 81	80 37 43	1 80	211779.2373	-0.0656	0.060	-0.0657	0.50
1843:	81 37 45	1 81	80 37 44	1 80	211779.2373	-0.0656	0.060	-0.0657	0.50
1844:	82 3 80	1 82	81 3 79	1 81	211807.8895	0.0411	0.060		
1845:	13 7 6	1 13	12 6 7	1 12	212046.9884	0.1360	0.090	0.1360	0.50
1846:	13 7 7	1 13	12 6 6	1 12	212046.9884	0.1360	0.090	0.1360	0.50
1847:	83 1 82	1 83	82 2 81	1 82	212077.4669	0.0534	0.090		
1848:	81 3 78	1 81	80 3 77	1 80	212230.0288	0.0940	0.060		
1849:	82 2 80	1 82	81 2 79	1 81	212353.9934	0.0656	0.060		
1850:	83 2 82	1 83	82 2 81	1 82	212501.1511	0.0654	0.060		
1851:	83 1 82	1 83	82 1 81	1 82	212548.2468	0.0478	0.060		
1852:	81 4 77	1 81	80 4 76	1 80	212657.7816	-0.0689	0.060		
1853:	83 2 82	1 83	82 1 81	1 82	212971.8762	0.0051	0.040		
1854:	84 0 84	1 84	83 1 83	1 83	213055.2434	0.0090	0.025		
1855:	82 12 70	1 82	81 12 69	1 81	213062.1123	0.0052	0.025	0.0052	0.50
1856:	82 12 71	1 82	81 12 70	1 81	213062.1123	0.0052	0.025	0.0052	0.50
1857:	84 1 84	1 84	83 1 83	1 83	213065.5687	0.0800	0.060		
1858:	82 13 69	1 82	81 13 68	1 81	213067.4810	0.0100	0.025	0.0101	0.50
1859:	82 13 70	1 82	81 13 69	1 81	213067.4810	0.0100	0.025	0.0101	0.50
1860:	84 1 84	1 84	83 0 83	1 83	213077.0065	-0.0625	0.060		
1861:	82 14 68	1 82	81 14 67	1 81	213080.5100	-0.0147	0.025	-0.0147	0.50
1862:	82 14 69	1 82	81 14 68	1 81	213080.5100	-0.0147	0.025	-0.0147	0.50
1863:	82 4 79	1 82	81 4 78	1 81	213081.5423	-0.0973	0.060		

1864:	79	3	77	1	79	78	2	76	1	78	213082.4393	0.0784	0.090		
1865:	82	10	73	1	82	81	10	72	1	81	213083.8254	-0.0150	0.025	-0.0153	0.50
1866:	82	10	72	1	82	81	10	71	1	81	213083.8254	-0.0154	0.025	-0.0153	0.50
1867:	82	15	67	1	82	81	15	66	1	81	213099.8274	-0.0587	0.060	-0.0588	0.50
1868:	82	15	68	1	82	81	15	67	1	81	213099.8274	-0.0587	0.060	-0.0588	0.50
1869:	82	9	74	1	82	81	9	73	1	81	213119.3917	0.0343	0.030	0.0283	0.50
1870:	82	9	73	1	82	81	9	72	1	81	213119.3917	0.0221	0.030	0.0283	0.50
1871:	48	4	45	1	48	47	3	44	1	47	213138.3714	-0.0090	0.040		
1872:	82	17	65	1	82	81	17	64	1	81	213154.0389	0.0594	0.060	0.0594	0.50
1873:	82	17	66	1	82	81	17	65	1	81	213154.0389	0.0594	0.060	0.0594	0.50
1874:	82	8	75	1	82	81	8	74	1	81	213182.2526	0.2459	0.060	0.0848	0.50
1875:	82	8	74	1	82	81	8	73	1	81	213182.2526	-0.0763	0.060	0.0848	0.50
1876:	82	18	64	1	82	81	18	63	1	81	213187.4794	-0.0545	0.060	-0.0545	0.50
1877:	82	18	65	1	82	81	18	64	1	81	213187.4794	-0.0545	0.060	-0.0545	0.50
1878:	82	19	63	1	82	81	19	62	1	81	213224.9010	0.0093	0.025	0.0094	0.50
1879:	82	19	64	1	82	81	19	63	1	81	213224.9010	0.0093	0.025	0.0094	0.50
1880:	24	6	19	1	24	23	5	18	1	23	213258.5678	0.0073	0.090	-0.0080	0.50
1881:	24	6	18	1	24	23	5	19	1	23	213258.5678	-0.0232	0.090	-0.0080	0.50
1882:	82	20	62	1	82	81	20	61	1	81	213265.8127	0.0407	0.060	0.0407	0.50
1883:	82	20	63	1	82	81	20	62	1	81	213265.8127	0.0407	0.060	0.0407	0.50
1884:	82	7	76	1	82	81	7	75	1	81	213286.6059	0.0262	0.025		
1885:	82	7	75	1	82	81	7	74	1	81	213292.7926	-0.1013	0.060		
1886:	82	21	61	1	82	81	21	60	1	81	213309.9302	-0.0277	0.025	-0.0277	0.50
1887:	82	21	62	1	82	81	21	61	1	81	213309.9302	-0.0277	0.025	-0.0277	0.50
1888:	82	22	60	1	82	81	22	59	1	81	213357.2263	-0.0525	0.060	-0.0525	0.50
1889:	82	22	61	1	82	81	22	60	1	81	213357.2263	-0.0525	0.060	-0.0525	0.50
1890:	82	23	59	1	82	81	23	58	1	81	213407.6248	0.0267	0.060	0.0267	0.50
1891:	82	23	60	1	82	81	23	59	1	81	213407.6248	0.0267	0.060	0.0267	0.50
1892:	82	6	77	1	82	81	6	76	1	81	213439.0825	0.0949	0.060		
1893:	82	24	58	1	82	81	24	57	1	81	213460.8410	0.0361	0.060	0.0362	0.50
1894:	82	24	59	1	82	81	24	58	1	81	213460.8410	0.0361	0.060	0.0362	0.50
1895:	82	5	78	1	82	81	5	77	1	81	213515.0245	-0.0010	0.025		
1896:	82	25	57	1	82	81	25	56	1	81	213516.7671	-0.0401	0.060	-0.0402	0.50
1897:	82	25	58	1	82	81	25	57	1	81	213516.7671	-0.0401	0.060	-0.0402	0.50
1898:	82	6	76	1	82	81	6	75	1	81	213524.5237	0.1033	0.060		
1899:	82	26	56	1	82	81	26	55	1	81	213575.4816	-0.0467	0.060	-0.0468	0.50
1900:	82	26	57	1	82	81	26	56	1	81	213575.4816	-0.0467	0.060	-0.0468	0.50
1901:	82	27	55	1	82	81	27	54	1	81	213636.9803	0.0781	0.060	0.0781	0.50
1902:	82	27	56	1	82	81	27	55	1	81	213636.9803	0.0781	0.060	0.0781	0.50
1903:	82	28	54	1	82	81	28	53	1	81	213700.8593	-0.0120	0.025	-0.0120	0.50
1904:	82	28	55	1	82	81	28	54	1	81	213700.8593	-0.0120	0.025	-0.0120	0.50
1905:	82	29	53	1	82	81	29	52	1	81	213767.4787	0.0938	0.060	0.0938	0.50
1906:	82	29	54	1	82	81	29	53	1	81	213767.4787	0.0938	0.060	0.0938	0.50
1907:	82	30	52	1	82	81	30	51	1	81	213836.4704	0.0734	0.060	0.0734	0.50
1908:	82	30	53	1	82	81	30	52	1	81	213836.4704	0.0734	0.060	0.0734	0.50
1909:	82	31	51	1	82	81	31	50	1	81	213907.8655	0.0000	0.025	0.0000	0.50
1910:	82	31	52	1	82	81	31	51	1	81	213907.8655	0.0000	0.025	0.0000	0.50
1911:	35	5	31	1	35	34	4	30	1	34	214135.5837	0.0805	0.040		
1912:	82	34	48	1	82	81	34	47	1	81	214136.5004	-0.1231	0.060	-0.1231	0.50
1913:	82	34	49	1	82	81	34	48	1	81	214136.5004	-0.1231	0.060	-0.1231	0.50
1914:	83	3	81	1	83	82	3	80	1	82	214340.9287	-0.0560	0.060		
1915:	85	2	83	1	85	84	3	82	1	84	214363.6347	-0.0083	0.040		
1916:	82	3	79	1	82	81	3	78	1	81	214777.1305	-0.0410	0.060		
1917:	83	2	81	1	83	82	2	80	1	82	214851.3125	-0.0466	0.060		
1918:	84	2	83	1	84	83	2	82	1	83	215021.1265	-0.0325	0.060		
1919:	84	1	83	1	84	83	1	82	1	83	215063.7171	-0.0161	0.025		
1920:	82	4	78	1	82	81	4	77	1	81	215307.4382	-0.0342	0.060		
1921:	85	0	85	1	85	84	1	84	1	84	215575.3197	0.0112	0.025		
1922:	85	1	85	1	85	84	1	84	1	84	215584.3231	-0.0637	0.060		
1923:	85	0	85	1	85	84	0	84	1	84	215585.5520	-0.0106	0.025		
1924:	85	1	85	1	85	84	0	84	1	84	215594.6037	-0.0373	0.025		
1925:	83	4	80	1	83	82	4	79	1	82	215648.7137	-0.0399	0.060		
1926:	83	12	71	1	83	82	12	70	1	82	215656.5534	-0.0266	0.025	-0.0267	0.50
1927:	83	12	72	1	83	82	12	71	1	82	215656.5534	-0.0266	0.025	-0.0267	0.50
1928:	83	13	71	1	83	82	13	70	1	82	215661.3603	-0.0275	0.025	-0.0276	0.50
1929:	83	13	70	1	83	82	13	69	1	82	215661.3603	-0.0275	0.025	-0.0276	0.50
1930:	83	11	72	1	83	82	11	71	1	82	215661.8269	0.0007	0.025	0.0007	0.50
1931:	83	11	73	1	83	82	11	72	1	82	215661.8269	0.0007	0.025	0.0007	0.50
1932:	83	14	69	1	83	82	14	68	1	82	215674.0625	-0.0461	0.060	-0.0462	0.50
1933:	83	14	70	1	83	82	14	69	1	82	215674.0625	-0.0461	0.060	-0.0462	0.50
1934:	83	10	74	1	83	82	10	73	1	82	215680.4464	-0.0045	0.025	-0.0048	0.50
1935:	83	10	73	1	83	82	10	72	1	82	215680.4464	-0.0050	0.025	-0.0048	0.50
1936:	83	15	68	1	83	82	15	67	1	82	215693.3210	0.0124	0.025	0.0125	0.50
1937:	83	15	69	1	83	82	15	68	1	82	215693.3210	0.0124	0.025	0.0125	0.50
1938:	83	9	75	1	83	82	9	74	1	82	215717.9340	0.0564	0.025	-0.0060	0.25
1939:	83	9	74	1	83	82	9	73	1	82	215717.9340	0.0415	0.025	-0.0060	0.25
1940:	83	16	67	1	83	82	16	66	1	82	215717.9340	-0.0610	0.025	-0.0060	0.25
1941:	83	16	68	1	83	82	16	67	1	82	215717.9340	-0.0610	0.025	-0.0060	0.25
1942:	83	17	66	1	83	82	17	65	1	82	215747.4917	0.0296	0.060	0.0296	0.50
1943:	83	17	67	1	83	82	17	66	1	82	215747.4917	0.0296	0.060	0.0296	0.50
1944:	83	18	65	1	83	82	18	64	1	82	215781.1864	-0.0087	0.025	-0.0088	0.50
1945:	83	18	66	1	83	82	18	65	1	82	215781.1864	-0.0087	0.025	-0.0088	0.50
1946:	83	8	76	1	83	82	8	75	1	82	215783.4401	0.0239	0.025		

1947:	83	8	75	1	83	82	8	74	1	82	215783.8258	0.0249	0.025		
1948:	83	19	64	1	83	82	19	63	1	82	215818.7415	-0.0697	0.060	-0.0697	0.50
1949:	83	19	65	1	83	82	19	64	1	82	215818.7415	-0.0697	0.060	-0.0697	0.50
1950:	83	20	63	1	83	82	20	62	1	82	215860.0005	-0.0189	0.025	-0.0189	0.50
1951:	83	20	64	1	83	82	20	63	1	82	215860.0005	-0.0189	0.025	-0.0189	0.50
1952:	83	7	77	1	83	82	7	76	1	82	215892.2236	-0.0048	0.025		
1953:	83	7	76	1	83	82	7	75	1	82	215899.5554	-0.0157	0.025		
1954:	83	21	62	1	83	82	21	61	1	82	215904.6513	0.0563	0.060	0.0563	0.50
1955:	83	21	63	1	83	82	21	62	1	82	215904.6513	0.0563	0.060	0.0563	0.50
1956:	83	22	61	1	83	82	22	60	1	82	215952.3448	-0.0163	0.025	-0.0164	0.50
1957:	83	22	62	1	83	82	22	61	1	82	215952.3448	-0.0163	0.025	-0.0164	0.50
1958:	83	23	60	1	83	82	23	59	1	82	216003.2517	0.0749	0.060	0.0749	0.50
1959:	83	23	61	1	83	82	23	60	1	82	216003.2517	0.0749	0.060	0.0749	0.50
1960:	83	6	78	1	83	82	6	77	1	82	216048.2434	0.0024	0.025		
1961:	83	24	59	1	83	82	24	58	1	82	216056.9812	0.0542	0.060	0.0542	0.50
1962:	83	24	60	1	83	82	24	59	1	82	216056.9812	0.0542	0.060	0.0542	0.50
1963:	83	5	79	1	83	82	5	78	1	82	216115.0917	0.0444	0.060		
1964:	83	26	57	1	83	82	26	56	1	82	216172.9309	0.0636	0.060	0.0637	0.50
1965:	83	26	58	1	83	82	26	57	1	82	216172.9309	0.0636	0.060	0.0637	0.50
1966:	83	27	56	1	83	82	27	55	1	82	216234.8850	-0.0245	0.025	-0.0246	0.50
1967:	83	27	57	1	83	82	27	56	1	82	216234.8850	-0.0245	0.025	-0.0246	0.50
1968:	83	28	55	1	83	82	28	54	1	82	216299.6949	0.1100	0.060	0.1100	0.50
1969:	83	28	56	1	83	82	28	55	1	82	216299.6949	0.1100	0.060	0.1100	0.50
1970:	83	29	54	1	83	82	29	53	1	82	216366.8323	-0.0085	0.025	-0.0085	0.50
1971:	83	29	55	1	83	82	29	54	1	82	216366.8323	-0.0085	0.025	-0.0085	0.50
1972:	83	30	53	1	83	82	30	52	1	82	216436.7093	0.0791	0.060	0.0791	0.50
1973:	83	30	54	1	83	82	30	53	1	82	216436.7093	0.0791	0.060	0.0791	0.50
1974:	83	34	49	1	83	82	34	48	1	82	216740.3432	0.0498	0.060	0.0498	0.50
1975:	83	34	50	1	83	82	34	49	1	82	216740.3432	0.0498	0.060	0.0498	0.50
1976:	83	5	78	1	83	82	5	77	1	82	216858.9801	0.0175	0.025		
1977:	83	3	80	1	83	82	3	79	1	82	217319.3518	0.0616	0.060		
1978:	84	2	82	1	84	83	2	81	1	83	217349.0494	0.0126	0.025		
1979:	85	2	84	1	85	84	2	83	1	84	217540.9530	0.0887	0.060		
1980:	85	1	84	1	85	84	1	83	1	84	217579.2749	-0.0374	0.060		
1981:	83	4	79	1	83	82	4	78	1	82	217953.5011	0.0307	0.060		
1982:	86	0	86	1	86	85	1	85	1	85	218095.1331	0.0965	0.060		
1983:	86	1	86	1	86	85	1	85	1	85	218103.0630	-0.0095	0.025		
1984:	86	0	86	1	86	85	0	85	1	85	218104.0079	-0.1070	0.060		
1985:	86	1	86	1	86	85	0	85	1	85	218112.1448	-0.0061	0.025		
1986:	84	4	81	1	84	83	4	80	1	83	218213.9997	0.0135	0.025		
1987:	84	12	72	1	84	83	12	71	1	83	218250.9320	0.0208	0.025	0.0208	0.50
1988:	84	12	73	1	84	83	12	72	1	83	218250.9320	0.0208	0.025	0.0208	0.50
1989:	84	13	72	1	84	83	13	71	1	83	218255.1425	0.0017	0.025	0.0018	0.50
1990:	84	13	71	1	84	83	13	70	1	83	218255.1425	0.0017	0.025	0.0018	0.50
1991:	84	11	73	1	84	83	11	72	1	83	218257.0005	-0.0426	0.060	-0.0426	0.50
1992:	84	11	74	1	84	83	11	73	1	83	218257.0005	-0.0426	0.060	-0.0426	0.50
1993:	84	14	70	1	84	83	14	69	1	83	218267.5228	0.0117	0.025	0.0118	0.50
1994:	84	14	71	1	84	83	14	70	1	83	218267.5228	0.0117	0.025	0.0118	0.50
1995:	84	10	75	1	84	83	10	74	1	83	218276.9999	0.0119	0.025	0.0117	0.50
1996:	84	10	74	1	84	83	10	73	1	83	218276.9999	0.0114	0.025	0.0117	0.50
1997:	84	15	69	1	84	83	15	68	1	83	218286.6075	0.0722	0.060	0.0722	0.50
1998:	84	15	70	1	84	83	15	69	1	83	218286.6075	0.0722	0.060	0.0722	0.50
1999:	84	16	68	1	84	83	16	67	1	83	218311.2452	0.0604	0.060	0.0604	0.50
2000:	84	16	69	1	84	83	16	68	1	83	218311.2452	0.0604	0.060	0.0604	0.50
2001:	84	9	76	1	84	83	9	75	1	83	218316.3884	0.0085	0.025	-0.0005	0.50
2002:	84	9	75	1	84	83	9	74	1	83	218316.3884	-0.0096	0.025	-0.0005	0.50
2003:	84	17	67	1	84	83	17	66	1	83	218340.7002	-0.0277	0.025	-0.0277	0.50
2004:	84	17	68	1	84	83	17	67	1	83	218340.7002	-0.0277	0.025	-0.0277	0.50
2005:	84	8	77	1	84	83	8	76	1	83	218384.8146	-0.0735	0.060		
2006:	84	8	76	1	84	83	8	75	1	83	218385.3847	0.0383	0.060		
2007:	84	19	65	1	84	83	19	64	1	83	218412.5247	0.0256	0.060	0.0256	0.50
2008:	84	19	66	1	84	83	19	65	1	83	218412.5247	0.0256	0.060	0.0256	0.50
2009:	84	20	64	1	84	83	20	63	1	83	218453.9829	-0.0462	0.060	-0.0462	0.50
2010:	84	20	65	1	84	83	20	64	1	83	218453.9829	-0.0462	0.060	-0.0462	0.50
2011:	84	7	78	1	84	83	7	77	1	83	218498.0408	0.0200	0.025		
2012:	84	21	64	1	84	83	21	63	1	83	218499.0149	0.0258	0.060	0.0259	0.50
2013:	84	21	63	1	84	83	21	62	1	83	218499.0149	0.0258	0.060	0.0259	0.50
2014:	84	7	77	1	84	83	7	76	1	83	218506.5810	0.0395	0.060		
2015:	84	22	62	1	84	83	22	61	1	83	218547.1895	-0.0064	0.025	-0.0065	0.50
2016:	84	22	63	1	84	83	22	62	1	83	218547.1895	-0.0064	0.025	-0.0065	0.50
2017:	84	23	61	1	84	83	23	60	1	83	218598.5259	0.0221	0.025	0.0221	0.50
2018:	84	23	62	1	84	83	23	61	1	83	218598.5259	0.0221	0.025	0.0221	0.50
2019:	84	24	60	1	84	83	24	59	1	83	218652.8338	0.0400	0.060	0.0401	0.50
2020:	84	24	61	1	84	83	24	60	1	83	218652.8338	0.0400	0.060	0.0401	0.50
2021:	84	6	79	1	84	83	6	78	1	83	218657.4475	0.0169	0.025		
2022:	84	25	59	1	84	83	25	58	1	83	218710.0219	0.0539	0.060	0.0539	0.50
2023:	84	25	60	1	84	83	25	59	1	83	218710.0219	0.0539	0.060	0.0539	0.50
2024:	84	5	80	1	84	83	5	79	1	83	218713.9594	-0.0088	0.025		
2025:	84	6	78	1	84	83	6	77	1	83	218766.3731	-0.0007	0.025		
2026:	84	26	58	1	84	83	26	57	1	83	218769.8937	-0.0505	0.060	-0.0505	0.50
2027:	84	26	59	1	84	83	26	58	1	83	218769.8937	-0.0505	0.060	-0.0505	0.50
2028:	84	28	56	1	84	83	28	55	1	83	218898.1074	0.0761	0.060	0.0762	0.50
2029:	84	28	57	1	84	83	28	56	1	83	218898.1074	0.0761	0.060	0.0762	0.50

```
2030: 84 29 55  1 84   83 29 54  1 83       218965.9671  -0.0599  0.060  -0.0600 0.50
2031: 84 29 56  1 84   83 29 55  1 83       218965.9671  -0.0599  0.060  -0.0600 0.50
2032: 84 30 54  1 84   83 30 53  1 83       219036.6446   0.0532  0.060   0.0532 0.50
2033: 84 30 55  1 84   83 30 54  1 83       219036.6446   0.0532  0.060   0.0532 0.50
2034: 84 32 52  1 84   83 32 51  1 83       219185.3183   0.0680  0.060   0.0680 0.50
2035: 84 32 53  1 84   83 32 52  1 83       219185.3183   0.0680  0.060   0.0680 0.50
2036: 37  5 32  1 37   36  4 33  1 36       219331.1389   0.0083  0.090
2037: 85  3 83  1 85   84  3 82  1 84       219403.6538  -0.0302  0.060
2038: 84 35 49  1 84   83 35 48  1 83       219426.5450   0.0742  0.060   0.0743 0.50
2039: 84 35 50  1 84   83 35 49  1 83       219426.5450   0.0742  0.060   0.0743 0.50
2040: 84  5 79  1 84   83  5 78  1 83       219521.6641  -0.1224  0.060
2041: 86  1 85  1 86   85  2 84  1 85       219752.3095   0.0449  0.090
2042: 85  2 83  1 85   84  2 82  1 84       219847.0458  -0.0185  0.025
2043: 16  7  9  1 16   15  6 10  1 15       219849.2037   0.0976  0.040   0.0977 0.50
2044: 16  7 10  1 16   15  6  9  1 15       219849.2037   0.0976  0.040   0.0977 0.50
2045: 84  3 81  1 84   83  3 80  1 83       219856.3568  -0.0108  0.025
2046: 52  4 49  1 52   51  3 48  1 51       220002.4140  -0.1112  0.090
2047: 86  2 85  1 86   85  2 84  1 85       220060.1471  -0.0665  0.060
2048: 86  1 85  1 86   85  1 84  1 85       220094.9290   0.0145  0.025
2049: 87  2 85  1 87   86  3 84  1 86       220216.6829   0.0462  0.090
2050: 86  2 85  1 86   85  1 84  1 85       220402.8104  -0.0530  0.090
2051: 84  4 80  1 84   83  4 79  1 83       220595.6485   0.0262  0.025
2052: 87  1 87  1 87   86  1 86  1 86       220621.4686  -0.0755  0.060
2053: 87  0 87  1 87   86  0 86  1 86       220622.4338  -0.0342  0.025
2054: 87  1 87  1 87   86  0 86  1 86       220629.4525  -0.1276  0.060
2055: 85  4 82  1 85   84  4 81  1 84       220777.3425   0.0105  0.025
2056: 85 12 73  1 85   84 12 72  1 84       220845.1401   0.0415  0.060   0.0415 0.50
2057: 85 12 74  1 85   84 12 73  1 84       220845.1401   0.0415  0.060   0.0415 0.50
2058: 85 13 73  1 85   84 13 72  1 84       220848.7359   0.0085  0.025   0.0085 0.50
2059: 85 13 72  1 85   84 13 71  1 84       220848.7359   0.0085  0.025   0.0085 0.50
2060: 85 11 74  1 85   84 11 73  1 84       220852.1327  -0.0131  0.025  -0.0131 0.50
2061: 85 11 75  1 85   84 11 74  1 84       220852.1327  -0.0130  0.025  -0.0131 0.50
2062: 85 14 71  1 85   84 14 70  1 84       220860.7749   0.0453  0.060   0.0454 0.50
2063: 85 14 72  1 85   84 14 71  1 84       220860.7749   0.0453  0.060   0.0454 0.50
2064: 85 10 76  1 85   84 10 75  1 84       220873.4394  -0.0109  0.025  -0.0113 0.50
2065: 85 10 75  1 85   84 10 74  1 84       220873.4394  -0.0116  0.025  -0.0113 0.50
2066: 85 15 70  1 85   84 15 69  1 84       220879.5904   0.0264  0.060   0.0265 0.50
2067: 85 15 71  1 85   84 15 70  1 84       220879.5904   0.0264  0.060   0.0265 0.50
2068: 85  9 77  1 85   84  9 76  1 84       220914.8713   0.0071  0.025  -0.0039 0.50
2069: 85  9 76  1 85   84  9 75  1 84       220914.8713  -0.0149  0.025  -0.0039 0.50
2070: 85 17 68  1 85   84 17 67  1 84       220933.7039  -0.0705  0.060  -0.0705 0.50
2071: 85 17 69  1 85   84 17 68  1 84       220933.7039  -0.0705  0.060  -0.0705 0.50
2072: 85 18 67  1 85   84 18 66  1 84       220967.8236  -0.0169  0.025  -0.0169 0.50
2073: 85 18 68  1 85   84 18 67  1 84       220967.8236  -0.0169  0.025  -0.0169 0.50
2074: 85  8 78  1 85   84  8 77  1 84       220986.4285   0.0047  0.025
2075: 85  8 77  1 85   84  8 76  1 84       220987.0384   0.0703  0.060
2076: 85 19 66  1 85   84 19 65  1 84       221005.9266  -0.0257  0.025  -0.0258 0.50
2077: 85 19 67  1 85   84 19 66  1 84       221005.9266  -0.0257  0.025  -0.0258 0.50
2078: 27  6 22  1 27   26  5 21  1 26       221043.1805   0.1338  0.040   0.0822 0.50
2079: 27  6 21  1 27   26  5 22  1 26       221043.1805   0.0305  0.040   0.0822 0.50
2080: 85 20 65  1 85   84 20 64  1 84       221047.7930  -0.0052  0.025  -0.0052 0.50
2081: 85 20 66  1 85   84 20 65  1 84       221047.7930  -0.0052  0.025  -0.0052 0.50
2082: 85 21 64  1 85   84 21 63  1 84       221093.0822  -0.0550  0.060  -0.0550 0.50
2083: 85 21 65  1 85   84 21 64  1 84       221093.0822  -0.0550  0.060  -0.0550 0.50
2084: 85  7 79  1 85   84  7 78  1 84       221103.9063  -0.0471  0.060
2085: 85  7 78  1 85   84  7 77  1 84       221113.8508   0.0302  0.060
2086: 85 23 62  1 85   84 23 61  1 84       221193.5955   0.0194  0.025   0.0195 0.50
2087: 85 23 63  1 85   84 23 62  1 84       221193.5955   0.0194  0.025   0.0195 0.50
2088: 85 24 61  1 85   84 24 60  1 84       221248.3214  -0.0806  0.060  -0.0807 0.50
2089: 85 24 62  1 85   84 24 61  1 84       221248.3214  -0.0806  0.060  -0.0807 0.50
2090: 85  6 80  1 85   84  6 79  1 84       221266.5226  -0.0001  0.025
2091: 85 25 60  1 85   84 25 59  1 84       221306.0961  -0.0610  0.060  -0.0610 0.50
2092: 85 25 61  1 85   84 25 60  1 84       221306.0961  -0.0610  0.060  -0.0610 0.50
2093: 85  5 81  1 85   84  5 80  1 84       221311.6919  -0.0335  0.060
2094: 85 26 59  1 85   84 26 58  1 84       221366.7207  -0.0356  0.060  -0.0356 0.50
2095: 85 26 60  1 85   84 26 59  1 84       221366.7207  -0.0356  0.060  -0.0356 0.50
2096: 85  6 79  1 85   84  6 78  1 84       221389.0908  -0.0826  0.060
2097: 85 30 55  1 85   84 30 54  1 84       221636.3352   0.0577  0.060   0.0578 0.50
2098: 85 30 56  1 85   84 30 55  1 84       221636.3352   0.0577  0.060   0.0578 0.50
2099: 85 31 54  1 85   84 31 53  1 84       221710.1551  -0.0172  0.025  -0.0172 0.50
2100: 85 31 55  1 85   84 31 54  1 84       221710.1551  -0.0172  0.025  -0.0172 0.50
2101: 38  5 34  1 38   37  4 33  1 37       221770.4406   0.0499  0.040
2102: 85 33 52  1 85   84 33 51  1 84       221865.4253  -0.0427  0.060  -0.0428 0.50
2103: 85 33 53  1 85   84 33 52  1 84       221865.4253  -0.0427  0.060  -0.0428 0.50
2104: 86  3 84  1 86   85  3 83  1 85       221933.2700  -0.0602  0.060
2105: 85  5 80  1 85   84  5 79  1 84       222186.7356   0.0250  0.060
2106: 87  1 86  1 87   86  2 85  1 86       222302.5429  -0.0268  0.040
2107: 86  2 84  1 86   85  2 83  1 85       222345.5053  -0.0196  0.025
2108: 85  3 82  1 85   84  3 81  1 84       222388.5802   0.0714  0.060
2109:116 14102  1116  115 14101  1115       301147.0930   0.0095  0.040   0.0096 0.50
2110:116 14103  1116  115 14102  1115       301147.0930   0.0095  0.040   0.0096 0.50
2111:116 15102  1116  115 15101  1115       301152.1777   0.0036  0.040   0.0036 0.50
2112:116 15101  1116  115 15100  1115       301152.1777   0.0036  0.040   0.0036 0.50
```

2113:116 13103	1116	115 13102	1115	301156.3349	0.0019	0.040	0.0019	0.50
2114:116 13104	1116	115 13103	1115	301156.3349	0.0019	0.040	0.0019	0.50
2115:116 16100	1116	115 16 99	1115	301169.0363	0.1779	0.150	0.1779	0.50
2116:116 16101	1116	115 16100	1115	301169.0363	0.1779	0.150	0.1779	0.50
2117:116 12105	1116	115 12104	1115	301183.9831	0.0231	0.040	0.0229	0.50
2118:116 12104	1116	115 12103	1115	301183.9831	0.0227	0.040	0.0229	0.50
2119:116 17 99	1116	115 17 98	1115	301195.1527	-0.0537	0.150	-0.0537	0.50
2120:116 17100	1116	115 17 99	1115	301195.1527	-0.0537	0.150	-0.0537	0.50
2121:116 18 98	1116	115 18 97	1115	301229.8714	0.0463	0.050	0.0464	0.50
2122:116 18 99	1116	115 18 98	1115	301229.8714	0.0463	0.050	0.0464	0.50
2123:116 11106	1116	115 11105	1115	301236.1175	-0.0239	0.040	-0.0290	0.50
2124:116 11105	1116	115 11104	1115	301236.1175	-0.0341	0.040	-0.0290	0.50
2125:116 19 97	1116	115 19 96	1115	301271.8983	0.2127	0.150	0.2127	0.50
2126:116 19 98	1116	115 19 97	1115	301271.8983	0.2127	0.150	0.2127	0.50
2127:116 10106	1116	115 10105	1115	301322.9254	-0.0633	0.040	0.0443	0.50
2128:116 10107	1116	115 10106	1115	301322.9254	0.1518	0.040	0.0443	0.50
2129:116 9108	1116	115 9107	1115	301459.8554	0.0344	0.040		
2130:116 9107	1116	115 9106	1115	301463.3664	-0.0266	0.040		
2131: 52 13 40	1 52	52 12 41	1 52	342002.3457	-0.0384	0.060	-0.0385	0.50
2132: 52 13 39	1 52	52 12 40	1 52	342002.3457	-0.0384	0.060	-0.0385	0.50
2133: 53 13 41	1 53	53 12 42	1 53	342015.3752	0.0172	0.060	0.0172	0.50
2134: 53 13 40	1 53	53 12 41	1 53	342015.3752	0.0172	0.060	0.0172	0.50
2135: 54 13 41	1 54	54 12 42	1 54	342028.3227	0.0138	0.060	0.0138	0.50
2136: 54 13 42	1 54	54 12 43	1 54	342028.3227	0.0138	0.060	0.0138	0.50
2137:115 13102	1115	115 12103	1115	342034.2942	0.0717	0.060	0.0709	0.50
2138:115 13103	1115	115 12104	1115	342034.2942	0.0700	0.060	0.0709	0.50
2139: 55 13 42	1 55	55 12 43	1 55	342041.2274	0.0053	0.060	0.0053	0.50
2140: 55 13 43	1 55	55 12 44	1 55	342041.2274	0.0053	0.060	0.0053	0.50
2141: 56 13 44	1 56	56 12 45	1 56	342054.1335	0.0509	0.060	0.0509	0.50
2142: 56 13 43	1 56	56 12 44	1 56	342054.1335	0.0509	0.060	0.0509	0.50
2143: 57 13 44	1 57	57 12 45	1 57	342066.9209	0.0459	0.060	0.0459	0.50
2144: 57 13 45	1 57	57 12 46	1 57	342066.9209	0.0459	0.060	0.0459	0.50
2145: 58 13 46	1 58	58 12 47	1 58	342079.5489	-0.0348	0.060	-0.0349	0.50
2146: 58 13 45	1 58	58 12 46	1 58	342079.5489	-0.0348	0.060	-0.0349	0.50
2147: 59 13 46	1 59	59 12 47	1 59	342092.1898	-0.0032	0.060	-0.0033	0.50
2148: 59 13 47	1 59	59 12 48	1 59	342092.1898	-0.0032	0.060	-0.0033	0.50
2149:132 4128	1132	131 4127	1131	342096.7694	-0.1518	0.150		
2150: 60 13 48	1 60	60 12 49	1 60	342104.7146	0.0278	0.060	0.0278	0.50
2151: 60 13 47	1 60	60 12 48	1 60	342104.7146	0.0278	0.060	0.0278	0.50
2152:112 13 99	1112	112 12100	1112	342108.5311	-0.0871	0.060	-0.0876	0.50
2153:112 13100	1112	112 12101	1112	342108.5311	-0.0880	0.060	-0.0876	0.50
2154: 61 13 48	1 61	61 12 49	1 61	342117.0923	0.0436	0.060	0.0437	0.50
2155: 61 13 49	1 61	61 12 50	1 61	342117.0923	0.0436	0.060	0.0437	0.50
2156: 62 13 49	1 62	62 12 50	1 62	342129.3016	0.0397	0.060	0.0397	0.50
2157: 62 13 50	1 62	62 12 51	1 62	342129.3016	0.0397	0.060	0.0397	0.50
2158:111 13 98	1111	111 12 99	1111	342130.6466	-0.0453	0.060	-0.0457	0.50
2159:111 13 99	1111	111 12100	1111	342130.6466	-0.0460	0.060	-0.0457	0.50
2160: 63 13 50	1 63	63 12 51	1 63	342141.3488	0.0390	0.060	0.0390	0.50
2161: 63 13 51	1 63	63 12 52	1 63	342141.3488	0.0390	0.060	0.0390	0.50
2162:133 3130	1133	132 3129	1132	342149.2627	-0.1137	0.150		
2163:110 13 97	1110	110 12 98	1110	342151.4348	-0.0194	0.060	-0.0197	0.50
2164:110 13 98	1110	110 12 99	1110	342151.4348	-0.0200	0.060	-0.0197	0.50
2165: 65 13 53	1 65	65 12 54	1 65	342164.8331	-0.0075	0.060	-0.0076	0.50
2166: 65 13 52	1 65	65 12 53	1 65	342164.8331	-0.0075	0.060	-0.0076	0.50
2167: 66 13 53	1 66	66 12 54	1 66	342176.1361	-0.1526	0.060	-0.1526	0.50
2168: 66 13 54	1 66	66 12 55	1 66	342176.1361	-0.1526	0.060	-0.1526	0.50
2169: 67 13 55	1 67	67 12 56	1 67	342187.5183	0.0169	0.060	0.0170	0.50
2170: 67 13 54	1 67	67 12 55	1 67	342187.5183	0.0169	0.060	0.0170	0.50
2171: 68 13 55	1 68	68 12 56	1 68	342198.4373	-0.0231	0.060	-0.0232	0.50
2172: 68 13 56	1 68	68 12 57	1 68	342198.4373	-0.0231	0.060	-0.0232	0.50
2173:107 13 94	1107	107 12 95	1107	342206.1810	0.0079	0.060	0.0078	0.50
2174:107 13 95	1107	107 12 96	1107	342206.1810	0.0076	0.060	0.0078	0.50
2175: 69 13 56	1 69	69 12 57	1 69	342209.1438	-0.0039	0.060	-0.0039	0.50
2176: 69 13 57	1 69	69 12 58	1 69	342209.1438	-0.0039	0.060	-0.0039	0.50
2177: 70 13 58	1 70	70 12 59	1 70	342219.5429	-0.0015	0.060	-0.0015	0.50
2178: 70 13 57	1 70	70 12 58	1 70	342219.5429	-0.0015	0.060	-0.0015	0.50
2179:106 13 93	1106	106 12 94	1106	342221.9786	-0.0097	0.060	-0.0099	0.50
2180:106 13 94	1106	106 12 95	1106	342221.9786	-0.0100	0.060	-0.0099	0.50
2181: 71 13 58	1 71	71 12 59	1 71	342229.6722	0.0404	0.060	0.0405	0.50
2182: 71 13 59	1 71	71 12 60	1 71	342229.6722	0.0404	0.060	0.0405	0.50
2183:105 13 92	1105	105 12 93	1105	342236.5493	-0.0906	0.060	-0.0908	0.50
2184:105 13 93	1105	105 12 94	1105	342236.5493	-0.0908	0.060	-0.0908	0.50
2185: 72 13 59	1 72	72 12 60	1 72	342239.3593	-0.0311	0.060	-0.0311	0.50
2186: 72 13 60	1 72	72 12 61	1 72	342239.3593	-0.0311	0.060	-0.0311	0.50
2187: 73 13 61	1 73	73 12 62	1 73	342248.7061	-0.0950	0.060	-0.0951	0.50
2188: 73 13 60	1 73	73 12 61	1 73	342248.7061	-0.0950	0.060	-0.0951	0.50
2189: 74 13 61	1 74	74 12 62	1 74	342257.8544	0.0101	0.060	0.0101	0.50
2190: 74 13 62	1 74	74 12 63	1 74	342257.8544	0.0101	0.060	0.0101	0.50
2191:103 13 90	1103	103 12 91	1103	342262.6721	0.1060	0.060	0.1060	0.50
2192:103 13 91	1103	103 12 92	1103	342262.6721	0.1059	0.060	0.1060	0.50
2193: 75 13 63	1 75	75 12 64	1 75	342266.4994	-0.0003	0.060	-0.0004	0.50
2194: 75 13 62	1 75	75 12 63	1 75	342266.4994	-0.0003	0.060	-0.0004	0.50
2195:102 13 89	1102	102 12 90	1102	342273.9277	0.0308	0.060	0.0308	0.50

2196:102 13 90	1102	102 12 91	1102	342273.9277	0.0307	0.060	0.0308	0.50
2197: 76 13 63	1 76	76 12 64	1 76	342274.8340	0.0863	0.060	0.0864	0.50
2198: 76 13 64	1 76	76 12 65	1 76	342274.8340	0.0863	0.060	0.0864	0.50
2199: 77 13 65	1 77	77 12 66	1 77	342282.6233	0.0559	0.060	0.0560	0.50
2200: 77 13 64	1 77	77 12 65	1 77	342282.6233	0.0559	0.060	0.0560	0.50
2201:101 13 88	1101	101 12 89	1101	342284.2261	0.0496	0.060	0.0496	0.50
2202:101 13 89	1101	101 12 90	1101	342284.2261	0.0495	0.060	0.0496	0.50
2203: 78 13 65	1 78	78 12 66	1 78	342290.0363	0.0980	0.120	0.0981	0.50
2204: 78 13 66	1 78	78 12 67	1 78	342290.0363	0.0980	0.120	0.0981	0.50
2205:100 13 87	1100	100 12 88	1100	342293.2975	-0.1347	0.120	-0.1348	0.50
2206:100 13 88	1100	100 12 89	1100	342293.2975	-0.1348	0.120	-0.1348	0.50
2207: 79 13 67	1 79	79 12 68	1 79	342296.7252	-0.1141	0.060	-0.1142	0.50
2208: 79 13 66	1 79	79 12 67	1 79	342296.7252	-0.1141	0.060	-0.1142	0.50
2209: 99 13 86	1 99	99 12 87	1 99	342301.7477	0.0562	0.060	0.0562	0.50
2210: 99 13 87	1 99	99 12 88	1 99	342301.7477	0.0561	0.060	0.0562	0.50
2211: 81 13 69	1 81	81 12 70	1 81	342309.1318	-0.0157	0.060	0.0676	0.25
2212: 81 13 68	1 81	81 12 69	1 81	342309.1318	-0.0157	0.060	0.0676	0.25
2213: 98 13 85	1 98	98 12 86	1 98	342309.1318	0.1510	0.060	0.0676	0.25
2214: 98 13 86	1 98	98 12 87	1 98	342309.1318	0.1509	0.060	0.0676	0.25
2215: 82 13 69	1 82	82 12 70	1 82	342314.4868	-0.0245	0.060	-0.0245	0.50
2216: 82 13 70	1 82	82 12 71	1 82	342314.4868	-0.0245	0.060	-0.0245	0.50
2217: 97 13 84	1 97	97 12 85	1 97	342315.4724	0.1455	0.120	0.1456	0.50
2218: 97 13 85	1 97	97 12 86	1 97	342315.4724	0.1455	0.120	0.1456	0.50
2219: 83 13 70	1 83	83 12 71	1 83	342319.3789	0.0598	0.060	0.0598	0.50
2220: 83 13 71	1 83	83 12 72	1 83	342319.3789	0.0598	0.060	0.0598	0.50
2221: 96 13 84	1 96	96 12 85	1 96	342320.7133	-0.0424	0.060	-0.0425	0.50
2222: 96 13 83	1 96	96 12 84	1 96	342320.7133	-0.0424	0.060	-0.0425	0.50
2223: 84 13 72	1 84	84 12 73	1 84	342323.6325	0.0838	0.060	0.0839	0.50
2224: 84 13 71	1 84	84 12 72	1 84	342323.6325	0.0838	0.060	0.0839	0.50
2225: 95 13 83	1 95	95 12 84	1 95	342325.1972	-0.0964	0.060	-0.0964	0.50
2226: 95 13 82	1 95	95 12 83	1 95	342325.1972	-0.0964	0.060	-0.0964	0.50
2227: 85 13 73	1 85	85 12 74	1 85	342327.2077	0.0302	0.060	0.0303	0.50
2228: 85 13 72	1 85	85 12 73	1 85	342327.2077	0.0302	0.060	0.0303	0.50
2229: 94 13 81	1 94	94 12 82	1 94	342328.9031	-0.0629	0.060	-0.0630	0.50
2230: 94 13 82	1 94	94 12 83	1 94	342328.9031	-0.0629	0.060	-0.0630	0.50
2231: 86 13 74	1 86	86 12 75	1 86	342330.2548	0.0719	0.075	0.0720	0.50
2232: 86 13 73	1 86	86 12 74	1 86	342330.2548	0.0720	0.075	0.0720	0.50
2233: 93 13 80	1 93	93 12 81	1 93	342331.6138	-0.1846	0.150	-0.1847	0.50
2234: 93 13 81	1 93	93 12 82	1 93	342331.6138	-0.1847	0.150	-0.1847	0.50
2235: 87 13 75	1 87	87 12 76	1 87	342332.6021	0.0603	0.075	0.0604	0.50
2236: 87 13 74	1 87	87 12 75	1 87	342332.6021	0.0603	0.075	0.0604	0.50
2237: 88 13 76	1 88	88 12 77	1 88	342333.9710	-0.2599	0.075	-0.0525	0.25
2238: 88 13 75	1 88	88 12 76	1 88	342333.9710	-0.2599	0.075	-0.0525	0.25
2239: 92 13 80	1 92	92 12 81	1 92	342333.9710	0.1549	0.075	-0.0525	0.25
2240: 92 13 79	1 92	92 12 80	1 92	342333.9710	0.1549	0.075	-0.0525	0.25
2241: 89 13 77	1 89	89 12 78	1 89	342335.2860	0.0591	0.060	0.0272	0.17
2242: 89 13 76	1 89	89 12 77	1 89	342335.2860	0.0591	0.060	0.0272	0.17
2243: 90 13 77	1 90	90 12 78	1 90	342335.2860	-0.2197	0.060	0.0272	0.17
2244: 90 13 78	1 90	90 12 79	1 90	342335.2860	-0.2197	0.060	0.0272	0.17
2245: 91 13 78	1 91	91 12 79	1 91	342335.2860	0.2423	0.060	0.0272	0.17
2246: 91 13 79	1 91	91 12 80	1 91	342335.2860	0.2423	0.060	0.0272	0.17
2247:131 5126	1131	130 5125	1130	342481.6282	-0.1578	0.150		
2248:132 15118	1132	131 15117	1131	342484.4388	-0.1085	0.080	-0.1086	0.50
2249:132 15117	1132	131 15116	1131	342484.4388	-0.1085	0.080	-0.1086	0.50
2250:132 16117	1132	131 16116	1131	342491.6083	0.0169	0.080	0.0170	0.50
2251:132 16116	1132	131 16115	1131	342491.6083	0.0169	0.080	0.0170	0.50
2252:132 14118	1132	131 14117	1131	342493.4292	0.0068	0.080	0.0069	0.50
2253:132 14119	1132	131 14118	1131	342493.4292	0.0068	0.080	0.0069	0.50
2254:132 17115	1132	131 17114	1131	342511.7987	0.1237	0.080	0.1238	0.50
2255:132 17116	1132	131 17115	1131	342511.7987	0.1237	0.080	0.1238	0.50
2256:132 18114	1132	131 18113	1131	342542.8269	0.0913	0.080	0.0913	0.50
2257:132 18115	1132	131 18114	1131	342542.8269	0.0913	0.080	0.0913	0.50
2258:132 12121	1132	131 12120	1131	342577.5687	0.0381	0.080	0.0348	0.50
2259:132 12120	1132	131 12119	1131	342577.5687	0.0313	0.080	0.0348	0.50
2260:132 12121	1132	131 12120	1131	342577.5796	0.0490	0.080	0.0457	0.50
2261:132 12120	1132	131 12119	1131	342577.5796	0.0422	0.080	0.0457	0.50
2262:132 19113	1132	131 19112	1131	342583.3895	0.1304	0.080	0.1304	0.50
2263:132 19114	1132	131 19113	1131	342583.3895	0.1304	0.080	0.1304	0.50
2264:134 3132	1134	133 3131	1133	342601.9162	0.0817	0.080		
2265:134 2132	1134	133 2131	1133	342606.4325	0.1497	0.080		
2266:132 20112	1132	131 20111	1131	342632.1969	0.0870	0.080	0.0870	0.50
2267:132 20113	1132	131 20112	1131	342632.1969	0.0870	0.080	0.0870	0.50
2268:132 11122	1132	131 11121	1131	342668.4574	-0.1155	0.150	-0.1834	0.50
2269:132 11121	1132	131 11120	1131	342668.4574	-0.2512	0.150	-0.1834	0.50
2270:132 21111	1132	131 21110	1131	342688.5152	0.0944	0.080	0.0945	0.50
2271:132 21112	1132	131 21111	1131	342688.5152	0.0944	0.080	0.0945	0.50
2272:132 10123	1132	131 10122	1131	342810.9470	0.0864	0.080		
2273:132 23109	1132	131 23108	1131	342820.8276	-0.0428	0.080	-0.0429	0.50
2274:132 23110	1132	131 23109	1131	342820.8276	-0.0428	0.080	-0.0429	0.50
2275:132 25107	1132	131 25106	1131	342976.6513	-0.0712	0.080	-0.0712	0.50
2276:132 25108	1132	131 25107	1131	342976.6513	-0.0712	0.080	-0.0712	0.50
2277:132 9124	1132	131 9123	1131	343024.2150	-0.1949	0.150		
2278:132 9123	1132	131 9122	1131	343051.4599	-0.0022	0.080		

```
2279:135  2134  1135  134  2133  1134       343152.8097  -0.0575  0.080  -0.1320 0.50
2280:135  1134  1135  134  1133  1134       343152.8097  -0.2065  0.080  -0.1320 0.50
2281: 75  6 69  1 75   74  5 70  1 74       343164.1034  -0.0173  0.080
2282: 53  8 45  1 53   52  7 46  1 52       343235.7380  -0.0887  0.080  -0.0476 0.50
2283: 53  8 46  1 53   52  7 45  1 52       343235.7380  -0.0063  0.080  -0.0476 0.50
2284:132  7126  1132  131  7125  1131       343401.0718  -0.0897  0.080
2285:131  6125  1131  130  6124  1130       343424.1094  -0.1274  0.150
2286:132  8124  1132  131  8123  1131       343537.1874  -0.1642  0.080
2287: 64  7 57  1 64   63  6 58  1 63       343648.3579   0.2020  0.150
2288:136  0136  1136  135  1135  1135       343721.4639  -0.0395  0.080  -0.0567 0.25
2289:136  1136  1136  135  1135  1135       343721.4639  -0.0556  0.080  -0.0567 0.25
2290:136  0136  1136  135  0135  1135       343721.4639  -0.0578  0.080  -0.0567 0.25
2291:136  1136  1136  135  0135  1135       343721.4639  -0.0739  0.080  -0.0567 0.25
2292:133  5129  1133  132  5128  1132       343978.3312  -0.0372  0.080
2293:135  3132  1135  134  4131  1134       346483.2975   0.0195  0.150
2294:133 34100  1133  132 34 99  1132       346500.4087  -0.0932  0.150  -0.0932 0.50
2295:133 34 99  1133  132 34 98  1132       346500.4087  -0.0932  0.150  -0.0932 0.50
2296: 71  3 68  1 71   70  2 69  1 70       346506.4751   0.1341  0.150
2297: 78  6 73  1 78   77  5 72  1 77       346545.3672   0.0390  0.150
2298:135  3132  1135  134  3131  1134       347145.7970  -0.1211  0.150
2299:134 15120  1134  133 15119  1133       347645.8987   0.2171  0.150   0.2172 0.50
2300:134 15119  1134  133 15118  1133       347645.8987   0.2171  0.150   0.2172 0.50
2301:134 17117  1134  133 17116  1133       347670.4872   0.2118  0.150   0.2119 0.50
2302:134 17118  1134  133 17117  1133       347670.4872   0.2118  0.150   0.2119 0.50
2303:134 18116  1134  133 18115  1133       347700.6380  -0.0485  0.080  -0.0486 0.50
2304:134 18117  1134  133 18116  1133       347700.6380  -0.0485  0.080  -0.0486 0.50
2305:134 19115  1134  133 19114  1133       347740.9042   0.0347  0.080   0.0348 0.50
2306:134 19116  1134  133 19115  1133       347740.9042   0.0347  0.080   0.0348 0.50
2307:134 12123  1134  133 12122  1133       347747.8174  -0.0101  0.080  -0.0148 0.50
2308:134 12122  1134  133 12121  1133       347747.8174  -0.0195  0.080  -0.0148 0.50
--------------------------------------------------------------------------------

PARAMETERS IN FIT (values truncated and Nlines statistics):

        10099            A  /MHz        15028.68713(46)                      1
        20099            B  /MHz         1334.107309(23)                     2
        30099            C  /MHz         1263.856698(22)                     3
          299           DJ  /kHz            0.1458341(29)                    4
         1199          DJK  /kHz           -7.54678(14)                      5
         2099           DK  /kHz          214.3805(86)                       6
        40199       deltaJ  /kHz            0.0220207(20)                    7
        41099       deltaK  /kHz            0.48160(41)                      8
          399           HJ  /Hz             0.00009962(13)                   9
         1299          HJK  /Hz            -0.006463(11)                    10
         2199          HKJ  /Hz            -0.05301(28)                     11
         3099           HK  /Hz             4.770(32)                       12
        40299           hJ  /Hz             0.00003144(15)                  13
         3199         LKKJ  /mHz           -0.02050(15)                     14
         2299          LJK  /mHz            0.000283(13)                    15

    110010000     X.aa*3/2  /MHz           -4.0900(24)                      16
    110040000    X.(b-c)/4  /MHz           -0.34403(83)                     17

 MICROWAVE AVG =        0.003530 MHz, IR AVG =        0.00000
 MICROWAVE RMS =        0.043454 MHz, IR RMS =        0.00000
 END OF ITERATION  1 OLD, NEW RMS ERROR=        0.93873         0.93873

  distinct frequency lines in fit:  1481
       distinct parameters of fit:    17

MICROWAVE       lines fitted       lines    lines      RMS      RMS ERROR    J range  Ka range    freq. range
            total   dv=0 dv.ne.0   UNFITTD  e>900
v"= 0         52     52      0        0      0       0.003114     1.28135     0   5    0   1     5125    18820
v"= 1       1429   1429      0        0      0       0.044178     0.92192     6 136    0  39    17936   347748
---------------------------------------------------------------------------------------------
total:      1481   1481      0        0      0       0.043400     0.93687

NOTE: the RMS values above are for Nlines statistics, but the 'total' values may differ slightly from
      those in the .FIT file since the o-c values for this evaluation are as rounded in the .FIT.


 PARAMETERS IN FIT WITH STANDARD ERRORS ON THOSE THAT ARE FITTED:
 (values rounded and degrees of freedom, Ndegf=Nlines-Nconst, statistics)

        10099            A  /MHz        15028.68713(43)                      1
        20099            B  /MHz         1334.107310(22)                     2
        30099            C  /MHz         1263.856699(21)                     3
          299           DJ  /kHz            0.1458342(27)                    4
         1199          DJK  /kHz           -7.54678(13)                      5
         2099           DK  /kHz          214.3805(81)                       6
        40199       deltaJ  /kHz            0.0220207(19)                    7
        41099       deltaK  /kHz            0.48160(39)                      8
          399           HJ  /Hz             0.00009962(12)                   9
```

1299	HJK	/Hz	-0.006464(10)	10
2199	HKJ	/Hz	-0.05301(26)	11
3099	HK	/Hz	4.770(30)	12
40299	hJ	/Hz	0.00003144(14)	13
3199	LKKJ	/mHz	-0.02051(14)	14
2299	LJK	/mHz	0.000283(12)	15
110010000	X.aa*3/2	/MHz	-4.0900(23)	16
110040000	X.(b-c)/4	/MHz	-0.34403(78)	17

CORRELATION COEFFICIENTS, C.ij:

	A	B	C	-DJ	-DJK	-DK	-deltaJ	-deltaK
A	1.0000							
B	0.1251	1.0000						
C	0.0985	0.3173	1.0000					
-DJ	-0.0534	-0.6259	-0.6239	1.0000				
-DJK	-0.2057	-0.3638	-0.3636	0.1517	1.0000			
-DK	-0.6944	0.0784	0.0916	-0.0384	-0.2462	1.0000		
-deltaJ	-0.0424	-0.5567	0.5404	0.0356	-0.0404	0.0382	1.0000	
-deltaK	0.0763	0.1316	-0.1409	0.1603	0.0287	-0.0719	-0.1707	1.0000
HJ	0.0265	0.4237	0.3235	-0.8176	0.1179	-0.0357	-0.1302	0.0025
HJK	0.0928	0.2726	0.3458	-0.1981	-0.7382	0.1774	0.1018	-0.0018
HKJ	0.1884	0.2122	0.1922	-0.0647	-0.8831	0.2206	0.0167	-0.0598
HK	0.6099	-0.0671	-0.0833	0.0444	0.2405	-0.9778	-0.0402	0.0577
hJ	0.0363	0.4035	-0.4440	-0.0429	0.1044	-0.0515	-0.8971	0.0219
LKKJ	-0.1535	-0.1298	-0.0911	0.0291	0.6634	-0.1745	0.0091	0.0569
LJK	-0.0930	-0.1584	-0.2196	0.0789	0.6801	-0.1423	-0.0866	0.0090
X.aa*3/2	-0.0115	-0.0128	-0.0150	0.0127	0.0090	0.0048	-0.0014	0.0000
X.(b-c)/4	0.0787	0.0288	0.0175	-0.0178	-0.0240	-0.0504	-0.0109	0.0080

	HJ	HJK	HKJ	HK	hJ	LKKJ	LJK	X.aa*3/2
HJ	1.0000							
HJK	-0.1859	1.0000						
HKJ	-0.0775	0.4903	1.0000					
HK	0.0395	-0.2091	-0.2265	1.0000				
hJ	0.1463	-0.1516	-0.0658	0.0544	1.0000			
LKKJ	0.0173	-0.1969	-0.9142	0.1854	0.0272	1.0000		
LJK	0.2011	-0.9082	-0.5382	0.1715	0.1242	0.2327	1.0000	
X.aa*3/2	-0.0073	-0.0070	-0.0057	-0.0042	0.0015	0.0035	0.0046	1.0000
X.(b-c)/4	0.0109	0.0135	0.0192	0.0441	0.0080	-0.0146	-0.0110	0.2101

	X.(b-c)/
X.(b-c)/4	1.0000

Mean value of |C.ij|, i.ne.j = 0.1800
Mean value of C.ij, i.ne.j = -0.0359

No correlations with absolute value greater than 0.9950

Worst fitted lines (obs-calc/error):

1417:	3.6	1610:	-3.3	1623:	-3.2	15:	-3.1
48:	-3.1	13:	-2.9	43:	-2.8	1613:	-2.6
721:	2.5	2167:	-2.5	653:	2.5	2043:	2.4
52:	-2.4	1327:	2.4	1335:	2.4	1336:	2.4
1621:	-2.3	1792:	2.3	1320:	2.3	1420:	2.3
2:	-2.2	1247:	2.2	1085:	2.1	2054:	-2.1
1657:	-2.1	1334:	2.1	1482:	2.1	1283:	2.1
2078:	2.1	2286:	-2.1	1912:	-2.1	1:	-2.1
884:	2.0	1454:	2.0	2040:	-2.0	1164:	2.0
861:	2.0	1911:	2.0	199:	-2.0	1297:	2.0
1830:	-2.0	58:	-2.0	1439:	2.0	264:	-2.0
1292:	-2.0	224:	1.9	1460:	1.9	53:	-1.9
1398:	1.9	855:	-1.9				

1417/	97	6	91	1	97	97	5	92	1	97	125845.2120	0.1441	0.040		
1610/	85	8	78	1	85	85	7	79	1	85	203428.7133	-0.0830	0.025		
1623/	77	8	69	1	77	77	7	70	1	77	204233.3202	-0.1891	0.060		
15/	2	1	2	0	2	3	0	3	0	3	5831.8000	-0.0062	0.002		
48/	1	1	1	0	2	0	0	0	0	1	16292.3020	-0.0092	0.003		
13:	2	1	2	0	3	3	0	3	0	4	5831.5480	-0.0058	0.002		
43:	1	1	0	0	2	1	0	1	0	2	13764.3855	-0.0056	0.002		
1613:	84	8	76	1	84	84	7	77	1	84	203491.6344	-0.1546	0.060		
721:	18	6	13	1	18	19	5	14	1	19	101515.8001	0.1041	0.040	0.1019	0.50
2167:	66	13	53	1	66	66	12	54	1	66	342176.1361	-0.1526	0.060	-0.1526	0.50

```
/////////////////////////////////////////////////////////////////////////////////////////////
//////////////// cut here - please display and print using a fixed width font ///////////////
/////////////////////////////////////////////////////////////////////////////////////////////


GA_valeronitrile, A-reduction                           Thu Dec 29 17:48:46 2022

-----------------------------------------------------------------------------------=========
                                             obs         o-c     error     blends     Notes
                                                                        o-c      wt
    / instead of : below denotes (o-c)>3*err
-----------------------------------------------------------------------------------=========
   1:  3  0  3  0  2    2  1  2  0  1         4380.9370   0.0035  0.002
   2:  3  0  3  0  4    2  1  2  0  3         4381.2600   0.0007  0.002
   3:  3  0  3  0  3    2  1  2  0  2         4381.8180   0.0011  0.002
   4:  1  1  0  0  0    1  0  1  0  1         6080.3900  -0.0017  0.002
   5:  1  1  0  0  2    1  0  1  0  2         6081.2640  -0.0046  0.002
   6:  1  1  0  0  2    1  0  1  0  1         6081.2830   0.0019  0.004
   7:  1  1  0  0  1    1  0  1  0  2         6081.8550  -0.0065  0.004
   8:  1  1  0  0  1    1  0  1  0  1         6081.8740   0.0000  0.002
   9:  2  1  1  0  1    2  0  2  0  1         6322.2040  -0.0037  0.002
  10:  2  1  1  0  3    2  0  2  0  3         6322.5740  -0.0003  0.002
  11:  2  1  1  0  2    2  0  2  0  2         6323.2330  -0.0012  0.002
  12:  2  1  2  0  2    1  1  1  0  2         6450.7260  -0.0015  0.002
  13:  2  1  2  0  1    1  1  1  0  0         6450.8450   0.0000  0.002
  14:  2  1  2  0  2    1  1  1  0  1         6451.3080   0.0000  0.002
  15:  2  1  2  0  3    1  1  1  0  2         6451.3610  -0.0018  0.002
  16:  2  1  2  0  1    1  1  1  0  1         6452.2950  -0.0013  0.002
  17:  2  0  2  0  3    1  0  1  0  2         6678.7800  -0.0016  0.002
  18:  3  1  2  0  2    3  0  3  0  2         6697.1490  -0.0031  0.002
  19:  3  1  2  0  2    3  0  3  0  3         6697.2540  -0.0029  0.003
  20:  3  1  2  0  4    3  0  3  0  4         6697.4020   0.0010  0.002
  21:  3  1  2  0  3    3  0  3  0  4         6698.0390   0.0046  0.004
  22:  3  1  2  0  3    3  0  3  0  3         6698.1120   0.0000  0.004
  23:  2  1  1  0  1    1  1  0  0  1         6919.1500   0.0010  0.002
  24:  2  1  1  0  3    1  1  0  0  2         6920.0860  -0.0013  0.002
  25:  2  1  1  0  2    1  1  0  0  1         6920.1180   0.0016  0.002
  26:  2  1  1  0  1    1  1  0  0  0         6920.6300  -0.0011  0.002
  27:  2  1  1  0  2    1  1  0  0  2         6920.7070  -0.0022  0.002
  28:  4  1  3  0  3    4  0  4  0  3         7220.5230  -0.0008  0.002
  29:  4  1  3  0  5    4  0  4  0  5         7220.7200  -0.0003  0.002
  30:  4  1  3  0  4    4  0  4  0  4         7221.4850   0.0003  0.002
  31:  5  1  4  0  4    5  0  5  0  4         7911.3010   0.0027  0.004
  32:  5  1  4  0  6    5  0  5  0  6         7911.4650  -0.0006  0.004
  33:  5  1  4  0  5    5  0  5  0  5         7912.2940   0.0064  0.004
  34:  4  0  4  0  3    3  1  3  0  2         8010.8580   0.0000  0.003
  35:  4  0  4  0  5    3  1  3  0  4         8011.0580  -0.0008  0.003
  36:  4  0  4  0  4    3  1  3  0  3         8011.5920  -0.0058  0.003
  37:  6  1  5  0  5    6  0  6  0  5         8790.8760   0.0012  0.002
  38:  6  1  5  0  7    6  0  6  0  7         8791.0250   0.0012  0.002
  39:  6  1  5  0  6    6  0  6  0  6         8791.9070   0.0003  0.002
  40:  3  1  3  0  2    2  1  2  0  1         9672.5920  -0.0004  0.002
  41:  3  1  3  0  3    2  1  2  0  2         9672.6720  -0.0040  0.002
  42:  3  1  3  0  4    2  1  2  0  3         9672.7110   0.0001  0.002
  43:  7  1  6  0  6    7  0  7  0  6         9880.6940   0.0016  0.002
  44:  7  1  6  0  8    7  0  7  0  8         9880.8310   0.0025  0.002
  45:  7  1  6  0  7    7  0  7  0  7         9881.7750   0.0016  0.002
  46:  3  0  3  0  3    2  0  2  0  2        10000.8500  -0.0052  0.003
  47:  3  0  3  0  4    2  0  2  0  3        10000.8950   0.0000  0.003
  48:  3  2  2  0  2    2  2  1  0  1        10028.7250   0.0035  0.002
  49:  3  2  1  0  3    2  2  0  0  2        10056.3200   0.0033  0.002
  50:  3  1  2  0  4    2  1  1  0  3        10375.7200  -0.0015  0.003
  51:  3  1  2  0  3    2  1  1  0  2        10375.7330   0.0000  0.003
  52:  3  1  2  0  2    2  1  1  0  1        10375.8400  -0.0053  0.003
  53:  8  1  7  0  7    8  0  8  0  7        11199.0300   0.0023  0.002
  54:  8  1  7  0  9    8  0  8  0  9        11199.1550   0.0008  0.002
  55:  8  1  7  0  8    8  0  8  0  8        11200.1600   0.0019  0.002
  56:  3  2  2  0  2    3  1  3  0  2        18599.2090  -0.0088  0.005
  57:  3  2  2  0  4    3  1  3  0  4        18599.4490  -0.0034  0.005
  58:  3  2  2  0  3    3  1  3  0  3        18600.1190  -0.0035  0.005
  59:  4  2  3  0  3    4  1  4  0  3        19076.3260  -0.0032  0.005
  60:  4  2  3  0  5    4  1  4  0  5        19076.4980  -0.0078  0.005
  61:  4  2  3  0  4    4  1  4  0  4        19077.1880  -0.0047  0.005
  62:  7  0  7  0  8    6  1  6  0  7        19150.2360  -0.0046  0.005
  63:  7  0  7  0  7    6  1  6  0  6        19150.6300  -0.0031  0.005
!
! hyperfine unresolved
!
  64: 31  7 24  1 31   31  6 25  1 31        75020.7567  -0.0485  0.025
  65: 22  2 20  1 22   21  2 19  1 21        75024.6028   0.0055  0.025
```

66:	33	7	27	1	33	33	6	28	1	33	75061.9695	0.0011	0.025		
67:	37	3	35	1	37	37	2	36	1	37	75096.4065	-0.0467	0.025		
68:	23	1	22	1	23	22	1	21	1	22	75283.9164	0.0153	0.025		
69:	32	7	26	1	32	32	6	27	1	32	75290.9653	-0.0329	0.025		
70:	30	7	23	1	30	30	6	24	1	30	75387.3488	-0.0121	0.025		
71:	31	7	25	1	31	31	6	26	1	31	75515.3035	-0.0510	0.025		
72:	29	7	22	1	29	29	6	23	1	29	75700.4238	0.0000	0.025		
73/	16	3	14	1	16	15	2	13	1	15	75715.7683	-0.1010	0.025		
74:	30	7	24	1	30	30	6	25	1	30	75731.1685	-0.0210	0.025		
75:	24	0	24	1	24	23	1	23	1	23	75738.7361	0.0006	0.025		
76:	24	1	24	1	24	23	1	23	1	23	75770.2779	-0.0067	0.025		
77:	24	0	24	1	24	23	0	23	1	23	75782.5474	0.0005	0.025		
78:	28	3	25	1	28	27	4	24	1	27	75786.7484	0.0152	0.025		
79:	24	1	24	1	24	23	0	23	1	23	75814.0936	-0.0025	0.025		
80:	47	7	41	1	47	47	6	42	1	47	75819.8176	0.0349	0.025		
81:	22	3	19	1	22	21	3	18	1	21	75834.3586	-0.0028	0.025		
82:	29	7	23	1	29	29	6	24	1	29	75935.6018	-0.0345	0.025		
83:	28	7	21	1	28	28	6	22	1	28	75968.4704	-0.0064	0.025		
84:	28	7	22	1	28	28	6	23	1	28	76126.6882	-0.0101	0.025		
85:	27	7	20	1	27	27	6	21	1	27	76198.5705	0.0108	0.025		
86:	48	8	40	1	48	48	7	41	1	48	76246.4834	0.0239	0.025		
87:	46	4	42	1	46	46	3	43	1	46	76250.0193	-0.0202	0.025		
88:	27	7	21	1	27	27	6	22	1	27	76303.1111	-0.0152	0.025		
89:	26	7	19	1	26	26	6	20	1	26	76396.4978	0.0332	0.025		
90:	23	2	22	1	23	22	1	21	1	22	76429.7242	0.0312	0.025		
91:	26	7	20	1	26	26	6	21	1	26	76464.2968	0.0038	0.025		
92:	25	7	18	1	25	25	6	19	1	25	76566.9429	0.0052	0.025		
93:	25	7	19	1	25	25	6	20	1	25	76610.0541	-0.0169	0.025		
94:	23	3	21	1	23	22	3	20	1	22	76688.8287	0.0018	0.025		
95:	24	7	18	1	24	24	6	19	1	24	76740.7622	0.0408	0.025		
96:	23	7	16	1	23	23	6	17	1	23	76840.5160	0.0636	0.025		
97:	23	7	17	1	23	23	6	18	1	23	76856.8584	0.0642	0.025		
98:	22	7	15	1	22	22	6	16	1	22	76949.3879	0.0462	0.025		
99:	23	13	10	1	23	22	13	9	1	22	77029.4913	0.0092	0.025	0.0092	0.50
100:	23	13	11	1	23	22	13	10	1	22	77029.4913	0.0092	0.025	0.0092	0.50
101:	23	14	10	1	23	22	14	9	1	22	77030.2184	0.0049	0.025	0.0050	0.50
102:	23	14	9	1	23	22	14	8	1	22	77030.2184	0.0049	0.025	0.0050	0.50
103:	23	12	11	1	23	22	12	10	1	22	77032.6461	-0.0082	0.025	-0.0083	0.50
104:	23	12	12	1	23	22	12	11	1	22	77032.6461	-0.0082	0.025	-0.0083	0.50
105:	23	15	8	1	23	22	15	7	1	22	77034.0623	0.0052	0.025	0.0052	0.50
106:	23	15	9	1	23	22	15	8	1	22	77034.0623	0.0052	0.025	0.0052	0.50
107:	23	16	7	1	23	22	16	6	1	22	77040.4643	0.0022	0.025	0.0022	0.50
108:	23	16	8	1	23	22	16	7	1	22	77040.4643	0.0022	0.025	0.0022	0.50
109:	23	11	12	1	23	22	11	11	1	22	77040.8810	-0.0224	0.025	-0.0224	0.50
110:	23	11	13	1	23	22	11	12	1	22	77040.8810	-0.0224	0.025	-0.0224	0.50
111:	23	17	6	1	23	22	17	5	1	22	77049.0410	0.0065	0.025	0.0065	0.50
112:	23	17	7	1	23	22	17	6	1	22	77049.0410	0.0065	0.025	0.0065	0.50
113:	23	10	14	1	23	22	10	13	1	22	77056.0431	0.0103	0.025	0.0103	0.50
114:	23	10	13	1	23	22	10	12	1	22	77056.0431	0.0103	0.025	0.0103	0.50
115:	23	18	5	1	23	22	18	4	1	22	77059.4757	-0.0099	0.025	-0.0100	0.50
116:	23	18	6	1	23	22	18	5	1	22	77059.4757	-0.0099	0.025	-0.0100	0.50
117:	7	5	3	1	7	6	4	2	1	6	77068.5134	0.0515	0.025	0.0070	0.50
118:	7	5	2	1	7	6	4	3	1	6	77068.5134	-0.0375	0.025	0.0070	0.50
119:	23	19	4	1	23	22	19	3	1	22	77071.5954	-0.0043	0.025	-0.0043	0.50
120:	23	19	5	1	23	22	19	4	1	22	77071.5954	-0.0043	0.025	-0.0043	0.50
121:	23	9	15	1	23	22	9	14	1	22	77080.9549	0.0126	0.025	0.0126	0.50
122:	23	9	14	1	23	22	9	13	1	22	77080.9549	0.0124	0.025	0.0126	0.50
123:	23	20	3	1	23	22	20	2	1	22	77085.1981	-0.0137	0.025	-0.0137	0.50
124:	23	20	4	1	23	22	20	3	1	22	77085.1981	-0.0137	0.025	-0.0137	0.50
125:	23	21	2	1	23	22	21	1	1	22	77100.2052	0.0114	0.025	0.0114	0.50
126:	23	21	3	1	23	22	21	2	1	22	77100.2052	0.0114	0.025	0.0114	0.50
127:	23	8	16	1	23	22	8	15	1	22	77120.5666	0.0070	0.025	0.0032	0.50
128:	23	8	15	1	23	22	8	14	1	22	77120.5666	-0.0006	0.025	0.0032	0.50
129:	20	7	14	1	20	20	6	15	1	20	77125.6982	-0.0192	0.025		
130:	23	7	17	1	23	22	7	16	1	22	77183.7673	-0.0247	0.025		
131:	23	7	16	1	23	22	7	15	1	22	77184.0316	-0.0381	0.025		
132:	24	1	23	1	24	23	2	22	1	23	77203.3320	0.0095	0.025		
133:	38	2	36	1	38	38	1	37	1	38	77225.4226	-0.0338	0.025		
134:	23	6	18	1	23	22	6	17	1	22	77286.0541	0.0119	0.025		
135:	23	6	17	1	23	22	6	16	1	22	77292.9578	-0.0012	0.025		
136:	16	7	9	1	16	16	6	10	1	16	77335.4910	0.1002	0.025	-0.0085	0.50
137:	16	7	10	1	16	16	6	11	1	16	77335.4910	-0.1173	0.025	-0.0085	0.50
138:	15	7	8	1	15	15	6	9	1	15	77368.0097	0.0469	0.030	-0.0028	0.50
139:	15	7	9	1	15	15	6	10	1	15	77368.0097	-0.0525	0.030	-0.0028	0.50
140:	14	7	7	1	14	14	6	8	1	14	77394.2884	0.0170	0.015	-0.0044	0.50
141:	14	7	8	1	14	14	6	9	1	14	77394.2884	-0.0258	0.015	-0.0044	0.50
142:	13	7	6	1	13	13	6	7	1	13	77415.1711	0.0107	0.025	0.0022	0.50
143:	13	7	7	1	13	13	6	8	1	13	77415.1711	-0.0064	0.025	0.0022	0.50
144:	23	4	20	1	23	22	4	19	1	22	77418.6491	0.0164	0.025		
145:	23	5	19	1	23	22	5	18	1	22	77427.8035	0.0237	0.025		
146:	12	7	5	1	12	12	6	6	1	12	77431.4261	0.0222	0.025	0.0190	0.50
147:	12	7	6	1	12	12	6	7	1	12	77431.4261	0.0158	0.025	0.0190	0.50
148:	11	7	4	1	11	11	6	5	1	11	77443.7106	-0.0014	0.025	-0.0026	0.50

149:	11	7	5	1	11	11	6	6	1	11	77443.7106	-0.0036	0.025	-0.0026 0.50
150:	9	7	2	1	9	9	6	3	1	9	77459.0922	0.0274	0.020	0.0274 0.50
151:	9	7	3	1	9	9	6	4	1	9	77459.0922	0.0273	0.020	0.0274 0.50
152:	10	7	3	1	10	10	6	4	1	10	77452.7044	-0.0305	0.020	-0.0308 0.50
153:	10	7	4	1	10	10	6	5	1	10	77452.7044	-0.0311	0.020	-0.0308 0.50
154:	8	7	1	1	8	8	6	2	1	8	77463.2640	0.0259	0.020	0.0260 0.50
155:	8	7	2	1	8	8	6	3	1	8	77463.2640	0.0259	0.020	0.0260 0.50
156/	38	3	36	1	38	38	2	37	1	38	77499.0446	-0.0845	0.025	
157:	23	5	18	1	23	22	5	17	1	22	77537.5039	0.0007	0.025	
158:	45	5	41	1	45	45	4	42	1	45	77754.3977	-0.0096	0.025	
159:	42	3	39	1	42	42	2	40	1	42	77770.3123	-0.0185	0.025	
160:	24	2	23	1	24	23	2	22	1	23	78082.9625	-0.0079	0.025	
161:	23	2	21	1	23	22	2	20	1	22	78196.0353	0.0020	0.025	
162:	23	4	19	1	23	22	4	18	1	22	78328.0372	0.0251	0.025	
163:	24	1	23	1	24	23	1	22	1	23	78349.1141	-0.0003	0.025	
164:	11	4	7	1	11	10	3	8	1	10	78533.5468	0.0087	0.025	
165:	25	0	25	1	25	24	1	24	1	24	78854.1483	-0.0022	0.025	
166:	25	1	25	1	25	24	1	24	1	24	78876.7943	-0.0117	0.025	
167:	25	0	25	1	25	24	0	24	1	24	78885.6920	-0.0078	0.025	
168:	25	1	25	1	25	24	0	24	1	24	78908.3536	-0.0017	0.025	
169:	24	2	23	1	24	23	1	22	1	23	79228.7043	-0.0580	0.025	
170:	23	3	20	1	23	22	3	19	1	22	79326.6981	0.0036	0.025	
171/	18	3	16	1	18	17	2	15	1	17	79324.5139	-0.0907	0.025	
172:	39	2	37	1	39	39	1	38	1	39	79698.9678	-0.0178	0.025	
173:	24	3	22	1	24	23	3	21	1	23	79922.0642	0.0469	0.025	
174:	24	14	10	1	24	23	14	9	1	23	80381.9802	-0.0191	0.025	-0.0191 0.50
175:	24	14	11	1	24	23	14	10	1	23	80381.9802	-0.0191	0.025	-0.0191 0.50
176:	24	13	11	1	24	23	13	10	1	23	80382.2956	0.0032	0.025	0.0032 0.50
177:	24	13	12	1	24	23	13	11	1	23	80382.2956	0.0032	0.025	0.0032 0.50
178:	24	15	10	1	24	23	15	9	1	23	80385.1972	0.0365	0.025	0.0366 0.50
179:	24	15	9	1	24	23	15	8	1	23	80385.1972	0.0365	0.025	0.0366 0.50
180:	23	8	15	1	23	23	7	16	1	23	89006.1423	-0.0408	0.025	
181:	23	8	16	1	23	23	7	17	1	23	89006.8321	0.0643	0.025	
182:	24	12	12	1	24	23	12	11	1	23	80386.9180	-0.0211	0.025	-0.0211 0.50
183:	24	12	13	1	24	23	12	12	1	23	80386.9180	-0.0211	0.025	-0.0211 0.50
184:	24	16	8	1	24	23	16	7	1	23	80391.1781	0.0273	0.025	0.0273 0.50
185:	24	16	9	1	24	23	16	8	1	23	80391.1781	0.0273	0.025	0.0273 0.50
186:	24	11	13	1	24	23	11	12	1	23	80397.2625	-0.0110	0.025	-0.0111 0.50
187:	24	11	14	1	24	23	11	13	1	23	80397.2625	-0.0110	0.025	-0.0111 0.50
188:	24	17	7	1	24	23	17	6	1	23	80399.5743	0.0517	0.025	0.0518 0.50
189:	24	17	8	1	24	23	17	7	1	23	80399.5743	0.0517	0.025	0.0518 0.50
190:	24	18	6	1	24	23	18	5	1	23	80409.9692	0.0204	0.025	0.0205 0.50
191:	24	18	7	1	24	23	18	6	1	23	80409.9692	0.0204	0.025	0.0205 0.50
192:	24	10	14	1	24	23	10	13	1	23	80415.3551	0.0064	0.025	0.0064 0.50
193:	24	10	15	1	24	23	10	14	1	23	80415.3551	0.0064	0.025	0.0064 0.50
194:	24	19	6	1	24	23	19	5	1	23	80422.1992	0.0146	0.025	0.0146 0.50
195:	24	19	5	1	24	23	19	4	1	23	80422.1992	0.0146	0.025	0.0146 0.50
196:	24	20	4	1	24	23	20	3	1	23	80436.0926	0.0490	0.025	0.0491 0.50
197:	24	20	5	1	24	23	20	4	1	23	80436.0926	0.0490	0.025	0.0491 0.50
198:	24	9	16	1	24	23	9	15	1	23	80444.4888	0.0187	0.025	0.0186 0.50
199:	24	9	15	1	24	23	9	14	1	23	80444.4888	0.0184	0.025	0.0186 0.50
200:	24	8	17	1	24	23	8	16	1	23	80490.2860	0.0195	0.025	0.0121 0.50
201:	24	8	16	1	24	23	8	15	1	23	80490.2860	0.0047	0.025	0.0121 0.50
202:	24	7	18	1	24	23	7	17	1	23	80562.9511	0.0604	0.025	
203/	24	7	17	1	24	23	7	16	1	23	80563.2752	-0.1014	0.025	
204:	24	6	18	1	24	23	6	17	1	23	80689.9694	0.0079	0.025	
205:	24	4	21	1	24	23	4	20	1	23	80773.4696	0.0144	0.025	
206:	24	5	20	1	24	23	5	19	1	23	80829.2513	-0.0094	0.025	
207:	24	5	19	1	24	23	5	18	1	23	80986.7435	-0.0666	0.025	
208:	33	4	29	1	33	32	5	28	1	32	81091.9324	-0.0547	0.025	
209:	25	2	24	1	25	24	2	23	1	24	81210.5215	-0.0206	0.025	
210:	43	4	40	1	43	43	3	41	1	43	81327.2332	-0.0084	0.025	
211:	24	2	22	1	24	23	2	21	1	23	81327.8092	0.0051	0.025	
212:	25	1	24	1	25	24	1	23	1	24	81419.6515	-0.0027	0.025	
213:	12	4	9	1	12	11	3	8	1	11	81506.9812	-0.0113	0.025	
214:	12	4	8	1	12	11	3	9	1	11	81900.3314	-0.0156	0.025	
215:	24	4	20	1	24	23	4	19	1	23	81915.0595	-0.0013	0.025	
216:	26	0	26	1	26	25	1	25	1	25	81966.4579	-0.0035	0.025	
217:	26	1	26	1	26	25	1	25	1	25	81982.6862	-0.0027	0.025	
218:	26	0	26	1	26	25	0	25	1	25	81989.1095	-0.0074	0.025	
219:	26	1	26	1	26	25	0	25	1	25	82005.3286	-0.0158	0.025	
220:	25	2	24	1	25	24	1	23	1	24	82090.1956	0.0055	0.025	
221:	40	2	38	1	40	40	1	39	1	40	82158.8368	0.0001	0.025	
222:	24	3	21	1	24	23	3	20	1	23	82785.2615	0.0121	0.025	
223:	25	3	23	1	25	24	3	22	1	24	83140.4372	-0.0054	0.025	
224:	25	14	11	1	25	24	14	10	1	24	83734.1080	-0.0069	0.025	-0.0069 0.50
225:	25	14	12	1	25	24	14	11	1	24	83734.1080	-0.0069	0.025	-0.0069 0.50
226:	25	13	12	1	25	24	13	11	1	24	83735.5712	0.0045	0.025	0.0046 0.50
227:	25	13	13	1	25	24	13	12	1	24	83735.5712	0.0045	0.025	0.0046 0.50
228:	25	15	10	1	25	24	15	9	1	24	83736.4960	0.0097	0.025	0.0097 0.50
229:	25	15	11	1	25	24	15	10	1	24	83736.4960	0.0097	0.025	0.0097 0.50
230:	25	12	13	1	25	24	12	12	1	24	83741.8778	0.0196	0.025	0.0196 0.50
231:	25	12	14	1	25	24	12	13	1	24	83741.8778	0.0196	0.025	0.0196 0.50

232:	25	17	8	1	25	24	17	7	1	24	83750.0643	-0.0080	0.025	-0.0081	0.50
233:	25	17	9	1	25	24	17	8	1	24	83750.0643	-0.0080	0.025	-0.0081	0.50
234:	25	11	14	1	25	24	11	13	1	24	83754.4956	-0.0034	0.025	-0.0034	0.50
235:	25	11	15	1	25	24	11	14	1	24	83754.4956	-0.0034	0.025	-0.0034	0.50
236:	25	18	8	1	25	24	18	7	1	24	83760.4161	0.0031	0.025	0.0032	0.50
237:	25	18	7	1	25	24	18	6	1	24	83760.4161	0.0031	0.025	0.0032	0.50
238:	25	10	15	1	25	24	10	14	1	24	83775.7952	-0.0196	0.025	-0.0196	0.50
239:	25	10	16	1	25	24	10	15	1	24	83775.7952	-0.0195	0.025	-0.0196	0.50
240:	25	9	17	1	25	24	9	16	1	24	83809.5640	0.0078	0.025	0.0075	0.50
241:	25	9	16	1	25	24	9	15	1	24	83809.5640	0.0071	0.025	0.0075	0.50
242:	26	1	25	1	26	25	2	24	1	25	83824.9658	0.0089	0.025		
243:	25	8	18	1	25	24	8	17	1	24	83862.1349	0.0118	0.025	-0.0021	0.50
244:	25	8	17	1	25	24	8	16	1	24	83862.1349	-0.0159	0.025	-0.0021	0.50
245:	47	8	40	1	47	47	7	41	1	47	84093.6882	-0.0252	0.025		
246:	44	8	37	1	44	44	7	38	1	44	84736.8082	-0.0304	0.025		
247:	40	8	32	1	40	40	7	33	1	40	84932.3890	-0.0280	0.025		
248:	41	8	34	1	41	41	7	35	1	41	85599.0618	-0.0257	0.025		
249:	27	0	27	1	27	26	1	26	1	26	85076.4484	0.0161	0.025		
250:	27	1	27	1	27	26	1	26	1	26	85088.0228	-0.0059	0.025		
251:	27	0	27	1	27	26	0	26	1	26	85092.6703	0.0104	0.025		
252:	27	1	27	1	27	26	0	26	1	26	85104.2405	-0.0158	0.025		
253:	25	4	21	1	25	24	4	20	1	24	85517.0226	0.0144	0.025		
254:	6	6	1	1	6	5	5	0	1	5	85636.1273	0.0307	0.025	0.0307	0.50
255:	6	6	0	1	6	5	5	1	1	5	85636.1273	0.0306	0.025	0.0307	0.50
256:	25	3	22	1	25	24	3	21	1	24	86206.2685	0.0094	0.025		
257:	26	3	24	1	26	25	3	23	1	25	86344.5397	-0.0064	0.025		
258:	26	14	12	1	26	25	14	11	1	25	87086.5532	-0.0194	0.025	-0.0194	0.50
259:	26	14	13	1	26	25	14	12	1	25	87086.5532	-0.0194	0.025	-0.0194	0.50
260:	26	15	11	1	26	25	15	10	1	25	87088.0152	-0.0266	0.025	-0.0266	0.50
261:	26	15	12	1	26	25	15	11	1	25	87088.0152	-0.0266	0.025	-0.0266	0.50
262:	26	13	13	1	26	25	13	12	1	25	87089.3197	-0.0030	0.025	-0.0030	0.50
263:	26	13	14	1	26	25	13	13	1	25	87089.3197	-0.0030	0.025	-0.0030	0.50
264:	26	12	14	1	26	25	12	13	1	25	87097.4496	0.0130	0.025	0.0130	0.50
265:	26	12	15	1	26	25	12	14	1	25	87097.4496	0.0130	0.025	0.0130	0.50
266:	26	11	15	1	26	25	11	14	1	25	87112.6181	0.0036	0.025	0.0036	0.50
267:	26	11	16	1	26	25	11	15	1	25	87112.6181	0.0036	0.025	0.0036	0.50
268:	26	10	17	1	26	25	10	16	1	25	87137.4554	-0.0240	0.025	-0.0240	0.50
269:	26	10	16	1	26	25	10	15	1	25	87137.4554	-0.0240	0.025	-0.0240	0.50
270:	26	9	18	1	26	25	9	17	1	25	87176.2705	0.0014	0.025	0.0008	0.50
271:	26	9	17	1	26	25	9	16	1	25	87176.2705	0.0001	0.025	0.0008	0.50
272:	26	8	19	1	26	25	8	18	1	25	87236.2592	0.0296	0.025	0.0044	0.50
273:	26	8	18	1	26	25	8	17	1	25	87236.2592	-0.0208	0.025	0.0044	0.50
274:	26	7	20	1	26	25	7	19	1	25	87330.2849	0.0041	0.025		
275:	26	7	19	1	26	25	7	18	1	25	87331.6515	-0.0113	0.025		
276:	20	8	12	1	20	20	7	13	1	20	89171.5541	0.0438	0.025	0.0026	0.50
277:	20	8	13	1	20	20	7	14	1	20	89171.5541	-0.0387	0.025	0.0026	0.50
278:	27	3	25	1	27	26	3	24	1	26	89534.9671	-0.0125	0.025		
279:	26	3	23	1	26	25	3	22	1	25	89586.5388	0.0101	0.025		
280:	28	1	27	1	28	27	2	26	1	27	90278.4503	-0.0048	0.025		
281:	27	14	14	1	27	26	14	13	1	26	90439.3929	0.0083	0.025	0.0084	0.50
282:	27	14	13	1	27	26	14	12	1	26	90439.3929	0.0083	0.025	0.0084	0.50
283:	27	15	12	1	27	26	15	11	1	26	90439.8333	-0.0017	0.025	-0.0018	0.50
284:	27	15	13	1	27	26	15	12	1	26	90439.8333	-0.0017	0.025	-0.0018	0.50
285:	27	13	14	1	27	26	13	13	1	26	90443.5751	-0.0032	0.025	-0.0033	0.50
286:	27	13	15	1	27	26	13	14	1	26	90443.5751	-0.0032	0.025	-0.0033	0.50
287:	27	16	11	1	27	26	16	10	1	26	90444.0383	-0.0012	0.025	-0.0013	0.50
288:	27	16	12	1	27	26	16	11	1	26	90444.0383	-0.0012	0.025	-0.0013	0.50
289:	27	17	10	1	27	26	17	9	1	26	90451.3684	0.0056	0.025	0.0056	0.50
290:	27	17	11	1	27	26	17	10	1	26	90451.3684	0.0056	0.025	0.0056	0.50
291:	27	12	15	1	27	26	12	14	1	26	90453.7013	0.0019	0.025	0.0019	0.50
292:	27	12	16	1	27	26	12	15	1	26	90453.7013	0.0019	0.025	0.0019	0.50
293:	27	18	9	1	27	26	18	8	1	26	90461.3442	0.0036	0.025	0.0036	0.50
294:	27	18	10	1	27	26	18	9	1	26	90461.3442	0.0036	0.025	0.0036	0.50
295:	27	11	16	1	27	26	11	15	1	26	90471.6590	0.0041	0.025	0.0042	0.50
296:	27	11	17	1	27	26	11	16	1	26	90471.6590	0.0041	0.025	0.0042	0.50
297:	27	19	8	1	27	26	19	7	1	26	90473.6263	-0.0005	0.025	-0.0005	0.50
298:	27	19	9	1	27	26	19	8	1	26	90473.6263	-0.0005	0.025	-0.0005	0.50
299:	27	20	7	1	27	26	20	6	1	26	90487.9472	-0.0107	0.025	-0.0108	0.50
300:	27	20	8	1	27	26	20	7	1	26	90487.9472	-0.0107	0.025	-0.0108	0.50
301:	27	10	18	1	27	26	10	17	1	26	90500.3944	0.0031	0.025	0.0032	0.50
302:	27	10	17	1	27	26	10	16	1	26	90500.3944	0.0031	0.025	0.0032	0.50
303:	27	21	6	1	27	26	21	5	1	26	90504.0989	-0.0312	0.025	-0.0313	0.50
304:	27	21	7	1	27	26	21	6	1	26	90504.0989	-0.0312	0.025	-0.0313	0.50
305:	27	22	5	1	27	26	22	4	1	26	90521.9956	0.0127	0.025	0.0127	0.50
306:	27	22	6	1	27	26	22	5	1	26	90521.9956	0.0127	0.025	0.0127	0.50
307:	27	2	25	1	27	26	2	24	1	26	90542.3408	0.0186	0.025		
308:	27	9	19	1	27	26	9	18	1	26	90544.6762	-0.0021	0.025	-0.0034	0.50
309:	27	9	18	1	27	26	9	17	1	26	90544.6762	-0.0047	0.025	-0.0034	0.50
310:	28	2	27	1	28	27	2	26	1	27	90565.2010	-0.0024	0.025		
311:	27	8	20	1	27	26	8	19	1	26	90612.7450	0.0571	0.025	0.0123	0.50
312:	27	8	19	1	27	26	8	18	1	26	90612.7450	-0.0325	0.025	0.0123	0.50
313:	28	1	27	1	28	27	1	26	1	27	90661.0476	-0.0023	0.025		
314:	27	7	21	1	27	26	7	20	1	26	90718.8354	0.0105	0.025		

315:	27	7	20	1	27	26	7	19	1	26	90721.0839	0.0048	0.025		
316:	27	4	24	1	27	26	4	23	1	26	90768.0324	0.0089	0.025		
317:	27	6	22	1	27	26	6	21	1	26	90880.0051	0.0135	0.025		
318:	27	6	21	1	27	26	6	20	1	26	90918.9794	-0.0045	0.025		
319:	27	6	21	1	27	26	6	20	1	26	90918.9823	-0.0016	0.025		
320:	28	2	27	1	28	27	1	26	1	27	90947.7934	-0.0048	0.025		
321:	41	1	40	1	41	41	0	41	1	41	92158.5865	0.0091	0.025		
322:	15	4	11	1	15	14	3	12	1	14	92188.1156	0.0158	0.025		
323:	8	6	3	1	8	7	5	2	1	7	92328.6675	0.0211	0.025	0.0202	0.50
324:	8	6	2	1	8	7	5	3	1	7	92328.6675	0.0193	0.025	0.0202	0.50
325:	28	3	26	1	28	27	3	25	1	27	92712.5797	0.0061	0.025		
326:	27	4	23	1	27	26	4	22	1	26	92732.8489	0.0343	0.025		
327:	27	3	24	1	27	26	3	23	1	26	92923.3205	0.0080	0.025		
328:	35	4	31	1	35	34	5	30	1	34	92985.0603	-0.0668	0.025		
329:	29	2	27	1	29	28	3	26	1	28	92942.3034	-0.0114	0.025		
330:	48	3	45	1	48	48	2	46	1	48	93209.5822	0.0065	0.025		
331:	16	4	13	1	16	15	3	12	1	15	93409.0557	0.0459	0.025		
332:	29	1	28	1	29	28	2	27	1	28	93462.6530	-0.0066	0.025		
333:	28	2	26	1	28	27	2	25	1	27	93578.5818	-0.0078	0.025		
334:	29	2	28	1	29	28	2	27	1	28	93676.5932	-0.0117	0.025		
335:	12	5	8	1	12	11	4	7	1	11	93740.9104	-0.0570	0.025		
336:	29	1	28	1	29	28	1	27	1	28	93749.3862	-0.0217	0.025		
337:	12	5	7	1	12	11	4	8	1	11	93753.8343	0.0003	0.025		
338:	28	15	13	1	28	27	15	12	1	27	93791.8843	0.0107	0.025	0.0107	0.50
339:	28	15	14	1	28	27	15	13	1	27	93791.8843	0.0107	0.025	0.0107	0.50
340:	28	14	15	1	28	27	14	14	1	27	93792.5850	0.0223	0.025	0.0224	0.50
341:	28	14	14	1	28	27	14	13	1	27	93792.5850	0.0223	0.025	0.0224	0.50
342:	28	16	12	1	28	27	16	11	1	27	93795.2962	0.0054	0.025	0.0055	0.50
343:	28	16	13	1	28	27	16	12	1	27	93795.2962	0.0054	0.025	0.0055	0.50
344:	28	13	15	1	28	27	13	14	1	27	93798.3520	0.0007	0.025	0.0008	0.50
345:	28	13	16	1	28	27	13	15	1	27	93798.3520	0.0007	0.025	0.0008	0.50
346:	28	17	11	1	28	27	17	10	1	27	93802.1036	-0.0021	0.025	-0.0022	0.50
347:	28	17	12	1	28	27	17	11	1	27	93802.1036	-0.0021	0.025	-0.0022	0.50
348:	28	12	16	1	28	27	12	15	1	27	93810.6718	0.0003	0.025	0.0004	0.50
349:	28	12	17	1	28	27	12	16	1	27	93810.6718	0.0003	0.025	0.0004	0.50
350:	28	18	11	1	28	27	18	10	1	27	93811.7909	-0.0107	0.025	-0.0108	0.50
351:	28	18	10	1	28	27	18	9	1	27	93811.7909	-0.0107	0.025	-0.0108	0.50
352:	28	19	9	1	28	27	19	8	1	27	93823.9964	0.0035	0.025	0.0035	0.50
353:	28	19	10	1	28	27	19	9	1	27	93823.9964	0.0035	0.025	0.0035	0.50
354:	28	11	17	1	28	27	11	16	1	27	93831.6414	-0.0135	0.025	-0.0136	0.50
355:	28	11	18	1	28	27	11	17	1	27	93831.6414	-0.0135	0.025	-0.0136	0.50
356:	28	20	8	1	28	27	20	7	1	27	93838.3903	0.0039	0.025	0.0040	0.50
357:	28	20	9	1	28	27	20	8	1	27	93838.3903	0.0039	0.025	0.0040	0.50
358:	28	21	7	1	28	27	21	6	1	27	93854.7668	0.0116	0.025	0.0116	0.50
359:	28	21	8	1	28	27	21	7	1	27	93854.7668	0.0116	0.025	0.0116	0.50
360:	28	10	19	1	28	27	10	18	1	27	93864.6146	0.0150	0.025	0.0150	0.50
361:	28	10	18	1	28	27	10	17	1	27	93864.6146	0.0149	0.025	0.0150	0.50
362:	28	22	6	1	28	27	22	5	1	27	93872.9266	0.0053	0.025	0.0053	0.50
363:	28	22	7	1	28	27	22	6	1	27	93872.9266	0.0053	0.025	0.0053	0.50
364:	28	23	5	1	28	27	23	4	1	27	93892.7430	0.0008	0.025	0.0008	0.50
365:	28	23	6	1	28	27	23	5	1	27	93892.7430	0.0008	0.025	0.0008	0.50
366:	28	24	4	1	28	27	24	3	1	27	93914.1153	0.0127	0.025	0.0127	0.50
367:	28	24	5	1	28	27	24	4	1	27	93914.1153	0.0127	0.025	0.0127	0.50
368:	28	9	20	1	28	27	9	19	1	27	93914.8596	0.0044	0.025	0.0020	0.50
369:	28	9	19	1	28	27	9	18	1	27	93914.8596	-0.0004	0.025	0.0020	0.50
370:	29	2	28	1	29	28	1	27	1	28	93963.3556	0.0022	0.025		
371:	28	8	21	1	28	27	8	20	1	27	93991.6781	0.0756	0.025	-0.0021	0.50
372:	28	8	20	1	28	27	8	19	1	27	93991.6781	-0.0798	0.025	-0.0021	0.50
373:	28	4	25	1	28	27	4	24	1	27	94072.1520	0.0027	0.025		
374:	28	7	22	1	28	27	7	21	1	27	94110.7692	0.0153	0.025		
375:	28	7	21	1	28	27	7	20	1	27	94114.3584	0.0012	0.025		
376:	28	6	23	1	28	27	6	22	1	27	94287.1783	-0.0035	0.025		
377:	45	2	43	1	45	45	1	44	1	45	94321.3067	0.0273	0.025		
378:	52	5	48	1	52	52	4	49	1	52	94324.2067	0.0546	0.025		
379:	28	6	22	1	28	27	6	21	1	27	94344.4661	0.0261	0.025		
380:	30	0	30	1	30	29	1	29	1	29	94397.1888	-0.0108	0.025		
381:	30	1	30	1	30	29	1	29	1	29	94401.3784	-0.0029	0.025		
382:	30	0	30	1	30	29	0	29	1	29	94403.0945	0.0090	0.025		
383:	30	1	30	1	30	29	0	29	1	29	94407.2610	-0.0061	0.025		
384:	28	5	24	1	28	27	5	23	1	27	94427.5012	0.0065	0.025		
385:	42	1	41	1	42	42	0	42	1	42	94545.6342	0.0451	0.025		
386:	53	9	45	1	53	53	8	46	1	53	94578.3557	-0.0287	0.025		
387:	52	9	44	1	52	52	8	45	1	52	94890.1052	-0.0225	0.025		
388:	28	5	23	1	28	27	5	22	1	27	94976.5305	0.0156	0.025		
389:	51	9	43	1	51	51	8	44	1	51	95226.9906	-0.0295	0.025		
390:	50	9	42	1	50	50	8	43	1	50	95580.5327	-0.0266	0.025		
391:	9	6	3	1	9	8	5	4	1	8	95673.7850	0.0120	0.025		
392:	16	4	12	1	16	15	3	13	1	15	95731.7046	-0.0004	0.025		
393:	29	3	27	1	29	28	3	26	1	28	95878.2909	-0.0080	0.025		
394:	49	9	41	1	49	49	8	42	1	49	95943.1789	-0.0356	0.025		
395:	28	3	25	1	28	27	3	24	1	27	96214.3799	-0.0101	0.025		
396:	48	9	40	1	48	48	8	41	1	48	96308.4532	-0.0176	0.025		
397:	28	4	24	1	28	27	4	23	1	27	96328.0464	0.0147	0.025		

398:	46 9 37	1 46	46 8 38	1 46	96351.1216	-0.0035	0.025		
399:	29 2 27	1 29	28 2 26	1 28	96609.4918	-0.0067	0.025		
400:	30 1 29	1 30	29 2 28	1 29	96626.6185	-0.0097	0.025		
401:	32 3 29	1 32	31 4 28	1 31	96661.2653	-0.0730	0.025		
402:	30 2 28	1 30	29 3 27	1 29	96704.5941	-0.0048	0.025		
403:	46 3 44	1 46	46 2 45	1 46	96761.7715	-0.0354	0.025		
404:	30 2 29	1 30	29 2 28	1 29	96785.6073	0.0026	0.025		
405:	30 1 29	1 30	29 1 28	1 29	96840.5739	0.0003	0.025		
406:	45 9 36	1 45	45 8 37	1 45	96877.3539	-0.0328	0.025		
407:	43 2 42	1 43	43 1 43	1 43	96933.6018	-0.0619	0.025	-0.0620	0.50
408:	43 2 42	1 43	43 1 43	1 43	96933.6018	-0.0619	0.025	-0.0620	0.50
409:	30 2 29	1 30	29 1 28	1 29	96999.5556	0.0055	0.025		
410:	46 9 38	1 46	46 8 39	1 46	97025.7328	-0.0570	0.025		
411:	13 5 9	1 13	12 4 8	1 12	97051.6673	-0.0303	0.025		
412:	13 5 8	1 13	12 4 9	1 12	97077.4668	0.0346	0.025		
413:	29 15 14	1 29	28 15 13	1 28	97144.1645	-0.0003	0.025	-0.0003	0.50
414:	29 15 15	1 29	28 15 14	1 28	97144.1645	-0.0003	0.025	-0.0003	0.50
415:	29 14 15	1 29	28 14 14	1 28	97146.1283	0.0094	0.025	0.0095	0.50
416:	29 14 16	1 29	28 14 15	1 28	97146.1283	0.0094	0.025	0.0095	0.50
417:	29 16 13	1 29	28 16 12	1 28	97146.6945	0.0019	0.025	0.0019	0.50
418:	29 16 14	1 29	28 16 13	1 28	97146.6945	0.0019	0.025	0.0019	0.50
419:	29 17 13	1 29	28 17 12	1 28	97152.9204	0.0050	0.025	0.0050	0.50
420:	29 17 12	1 29	28 17 11	1 28	97152.9204	0.0050	0.025	0.0050	0.50
421:	29 13 16	1 29	28 13 15	1 28	97153.6434	-0.0153	0.025	-0.0154	0.50
422:	29 13 17	1 29	28 13 16	1 28	97153.6434	-0.0153	0.025	-0.0154	0.50
423:	29 18 11	1 29	28 18 10	1 28	97162.2591	0.0000	0.025	0.0000	0.50
424:	29 18 12	1 29	28 18 11	1 28	97162.2591	0.0000	0.025	0.0000	0.50
425:	29 12 17	1 29	28 12 16	1 28	97168.3747	-0.0031	0.025	-0.0032	0.50
426:	29 12 18	1 29	28 12 17	1 28	97168.3747	-0.0031	0.025	-0.0032	0.50
427:	29 19 10	1 29	28 19 9	1 28	97174.3027	0.0065	0.025	0.0066	0.50
428:	29 19 11	1 29	28 19 10	1 28	97174.3027	0.0065	0.025	0.0066	0.50
429:	29 20 9	1 29	28 20 8	1 28	97188.6940	-0.0073	0.025	-0.0073	0.50
430:	29 20 10	1 29	28 20 9	1 28	97188.6940	-0.0073	0.025	-0.0073	0.50
431:	29 11 18	1 29	28 11 17	1 28	97192.6455	-0.0046	0.025	-0.0047	0.50
432:	29 11 19	1 29	28 11 18	1 28	97192.6455	-0.0046	0.025	-0.0047	0.50
433:	29 21 8	1 29	28 21 7	1 28	97205.2370	0.0133	0.025	0.0134	0.50
434:	29 21 9	1 29	28 21 8	1 28	97205.2370	0.0133	0.025	0.0134	0.50
435:	29 22 7	1 29	28 22 6	1 28	97223.6893	0.0236	0.025	0.0236	0.50
436:	29 22 8	1 29	28 22 7	1 28	97223.6893	0.0236	0.025	0.0236	0.50
437:	29 10 20	1 29	28 10 19	1 28	97230.1615	0.0072	0.025	0.0071	0.50
438:	29 10 19	1 29	28 10 18	1 28	97230.1615	0.0070	0.025	0.0071	0.50
439:	29 23 6	1 29	28 23 5	1 28	97243.8711	0.0011	0.025	0.0011	0.50
440:	29 23 7	1 29	28 23 6	1 28	97243.8711	0.0011	0.025	0.0011	0.50
441:	29 24 5	1 29	28 24 4	1 28	97265.7107	0.0014	0.025	0.0014	0.50
442:	29 24 6	1 29	28 24 5	1 28	97265.7107	0.0014	0.025	0.0014	0.50
443:	29 9 21	1 29	28 9 20	1 28	97286.9152	0.0429	0.025	0.0384	0.50
444:	29 9 20	1 29	28 9 19	1 28	97286.9152	0.0339	0.025	0.0384	0.50
445:	29 25 4	1 29	28 25 3	1 28	97289.0904	0.0113	0.025	0.0114	0.50
446:	29 25 5	1 29	28 25 4	1 28	97289.0904	0.0113	0.025	0.0114	0.50
447:	44 9 35	1 44	44 8 36	1 44	97344.3996	-0.0314	0.025		
448:	29 4 26	1 29	28 4 25	1 28	97361.0607	-0.0016	0.025		
449:	45 9 37	1 45	45 8 38	1 45	97369.8052	0.0451	0.025		
450:	29 8 22	1 29	28 8 21	1 28	97373.2118	0.1320	0.025	0.0001	0.50
451:	29 8 21	1 29	28 8 20	1 28	97373.2118	-0.1318	0.025	0.0001	0.50
452:	31 0 31	1 31	30 1 30	1 30	97502.0673	-0.0234	0.025		
453:	31 1 31	1 31	30 1 30	1 30	97505.0389	-0.0180	0.025		
454:	31 0 31	1 31	30 0 30	1 30	97506.2263	-0.0461	0.025	0.0094	0.50
455:	29 7 23	1 29	28 7 22	1 28	97506.2263	0.0649	0.025	0.0094	0.50
456:	31 1 31	1 31	30 0 30	1 30	97509.2347	-0.0039	0.025		
457:	29 7 22	1 29	28 7 21	1 28	97511.8098	-0.0034	0.025		
458:	29 6 24	1 29	28 6 23	1 28	97697.2346	0.0112	0.025		
459:	44 9 36	1 44	44 8 37	1 44	97699.9910	-0.0124	0.025		
460:	43 9 34	1 43	43 8 35	1 43	97760.4534	-0.0251	0.025		
461:	29 6 23	1 29	28 6 22	1 28	97779.8900	0.0237	0.025		
462:	29 5 25	1 29	28 5 24	1 28	97818.0733	0.0125	0.025		
463:	43 9 35	1 43	43 8 36	1 43	98014.5166	-0.0120	0.025		
464:	42 9 33	1 42	42 8 34	1 42	98132.4308	-0.0149	0.025		
465:	42 9 34	1 42	42 8 35	1 42	98311.9674	-0.0241	0.025		
466:	41 9 32	1 41	41 8 33	1 41	98466.1169	0.0052	0.025		
467:	29 5 24	1 29	28 5 23	1 28	98532.7627	0.0073	0.025		
468:	41 9 33	1 41	41 8 34	1 41	98591.5661	-0.0270	0.025		
469:	40 9 31	1 40	40 8 32	1 40	98766.2756	-0.0119	0.025		
470:	40 9 32	1 40	40 8 33	1 40	98852.9431	-0.0391	0.025		
471:	30 3 28	1 30	29 3 27	1 29	99033.2207	-0.0043	0.025		
472:	39 9 30	1 39	39 8 31	1 39	99037.0030	0.0278	0.025		
473:	39 9 31	1 39	39 8 32	1 39	99096.1290	-0.0368	0.025		
474:	38 9 29	1 38	38 8 30	1 38	99281.5341	0.0239	0.025		
475:	38 9 30	1 38	38 8 31	1 38	99321.3630	-0.0658	0.025		
476:	17 4 13	1 17	16 3 14	1 16	99367.9154	-0.0292	0.025		
477:	29 3 26	1 29	28 3 25	1 28	99458.3532	0.0104	0.025		
478:	37 9 28	1 37	37 8 29	1 37	99502.7178	0.0326	0.025		
479:	29 3 27	1 29	28 2 26	1 28	99545.5486	0.0659	0.025		
480:	30 2 28	1 30	29 2 27	1 29	99640.5764	-0.0066	0.025		

481:	36	9	27	1	36	36	8	28	1	36	99702.9226	0.0677	0.025		
482:	31	1	30	1	31	30	2	29	1	30	99774.9592	-0.0066	0.025		
483:	29	4	25	1	29	28	4	24	1	28	99902.7595	0.0053	0.025		
484:	31	1	30	1	31	30	1	29	1	30	99933.9344	-0.0078	0.025		
485:	31	2	30	1	31	30	1	29	1	30	100051.6353	-0.0006	0.025		
486:	30	17	14	1	30	29	17	13	1	29	100503.7817	-0.0107	0.025	-0.0107	0.50
487:	30	17	13	1	30	29	17	12	1	29	100503.7817	-0.0107	0.025	-0.0107	0.50
488:	30	13	17	1	30	29	13	16	1	29	100509.5308	0.0122	0.025	0.0122	0.50
489:	30	13	18	1	30	29	13	17	1	29	100509.5308	0.0122	0.025	0.0122	0.50
490:	30	18	12	1	30	29	18	11	1	29	100512.7234	0.0119	0.025	0.0120	0.50
491:	30	18	13	1	30	29	18	12	1	29	100512.7234	0.0119	0.025	0.0120	0.50
492:	30	12	18	1	30	29	12	17	1	29	100526.8301	-0.0136	0.025	-0.0136	0.50
493:	30	12	19	1	30	29	12	18	1	29	100526.8301	-0.0136	0.025	-0.0136	0.50
494:	30	20	10	1	30	29	20	9	1	29	100538.9134	0.0154	0.025	0.0155	0.50
495:	30	20	11	1	30	29	20	10	1	29	100538.9134	0.0154	0.025	0.0155	0.50
496:	30	11	19	1	30	29	11	18	1	29	100554.6893	0.0133	0.025	0.0134	0.50
497:	30	11	20	1	30	29	11	19	1	29	100554.6893	0.0133	0.025	0.0134	0.50
498:	30	21	10	1	30	29	21	9	1	29	100555.5403	0.0114	0.025	0.0114	0.50
499:	30	21	9	1	30	29	21	8	1	29	100555.5403	0.0114	0.025	0.0114	0.50
500/	30	9	21	1	30	30	8	22	1	30	100558.2053	0.0931	0.025		
501:	30	9	22	1	30	30	8	23	1	30	100559.2013	0.0679	0.025		
502:	30	22	8	1	30	29	22	7	1	29	100574.2422	0.0339	0.025	0.0340	0.50
503:	30	22	9	1	30	29	22	8	1	29	100574.2422	0.0339	0.025	0.0340	0.50
504:	30	23	7	1	30	29	23	6	1	29	100594.7926	0.0302	0.025	0.0302	0.50
505:	30	23	8	1	30	29	23	7	1	29	100594.7926	0.0302	0.025	0.0302	0.50
506:	30	10	21	1	30	29	10	20	1	29	100597.1104	0.0041	0.025	0.0039	0.50
507:	30	10	20	1	30	29	10	19	1	29	100597.1104	0.0037	0.025	0.0039	0.50
508:	32	0	32	1	32	31	1	31	1	31	100606.2806	0.0078	0.025		
509:	32	1	32	1	32	31	1	31	1	31	100608.3669	-0.0066	0.025		
510:	32	0	32	1	32	31	0	31	1	31	100609.2203	-0.0185	0.025		
511:	32	1	32	1	32	31	0	31	1	31	100611.3320	-0.0077	0.025		
512:	30	24	6	1	30	29	24	5	1	29	100617.0639	0.0127	0.025	0.0128	0.50
513:	30	24	7	1	30	29	24	6	1	29	100617.0639	0.0127	0.025	0.0128	0.50
514:	30	4	27	1	30	29	4	26	1	29	100634.2170	-0.0011	0.025		
515:	29	9	20	1	29	29	8	21	1	29	100654.9962	0.0377	0.025		
516:	29	9	21	1	29	29	8	22	1	29	100655.5517	-0.0048	0.025		
517:	30	9	22	1	30	29	9	21	1	29	100660.8216	0.0172	0.025	0.0092	0.50
518:	30	9	21	1	30	29	9	20	1	29	100660.8216	0.0011	0.025	0.0092	0.50
519:	30	8	23	1	30	29	8	22	1	29	100757.2512	0.0236	0.025		
520:	30	8	22	1	30	29	8	21	1	29	100757.6315	-0.0352	0.025		
521:	26	9	17	1	26	26	8	18	1	26	100886.4524	0.0370	0.025	-0.0157	0.50
522:	26	9	18	1	26	26	8	19	1	26	100886.4524	-0.0685	0.025	-0.0157	0.50
523:	7	7	0	1	7	6	6	1	1	6	100888.8495	-0.0233	0.025	-0.0233	0.50
524:	7	7	1	1	7	6	6	0	1	6	100888.8495	-0.0233	0.025	-0.0233	0.50
525:	30	7	24	1	30	29	7	23	1	29	100905.1532	0.0375	0.025		
526:	30	7	23	1	30	29	7	22	1	29	100913.8596	0.0347	0.025		
527:	25	9	16	1	25	25	8	17	1	25	100946.4710	0.0459	0.025	0.0178	0.50
528:	25	9	17	1	25	25	8	18	1	25	100946.4710	-0.0104	0.025	0.0178	0.50
529:	24	9	15	1	24	24	8	16	1	24	100999.0653	0.0461	0.025	0.0316	0.50
530:	24	9	16	1	24	24	8	17	1	24	100999.0653	0.0169	0.025	0.0316	0.50
531:	23	9	14	1	23	23	8	15	1	23	101044.8395	0.0094	0.025	0.0021	0.50
532:	23	9	15	1	23	23	8	16	1	23	101044.8395	-0.0052	0.025	0.0021	0.50
533:	22	9	13	1	22	22	8	14	1	22	101084.4842	0.0293	0.025	0.0257	0.50
534:	22	9	14	1	22	22	8	15	1	22	101084.4842	0.0221	0.025	0.0257	0.50
535:	30	6	25	1	30	29	6	24	1	29	101109.5271	-0.0352	0.025		
536:	21	9	12	1	21	21	8	13	1	21	101118.4917	0.0336	0.025	0.0320	0.50
537:	21	9	13	1	21	21	8	14	1	21	101118.4917	0.0303	0.025	0.0320	0.50
538:	20	9	11	1	20	20	8	12	1	20	101147.3788	0.0058	0.025	0.0050	0.50
539:	20	9	12	1	20	20	8	13	1	20	101147.3788	0.0042	0.025	0.0050	0.50
540:	19	9	10	1	19	19	8	11	1	19	101171.7316	0.0273	0.025	0.0270	0.50
541:	19	9	11	1	19	19	8	12	1	19	101171.7316	0.0266	0.025	0.0270	0.50
542:	18	9	9	1	18	18	8	10	1	18	101191.9320	0.0033	0.025	0.0032	0.50
543:	18	9	10	1	18	18	8	11	1	18	101191.9320	0.0031	0.025	0.0032	0.50
544:	30	5	26	1	30	29	5	25	1	29	101201.8341	0.0063	0.025		
545:	17	9	8	1	17	17	8	9	1	17	101208.5037	0.0076	0.025	0.0076	0.50
546:	17	9	9	1	17	17	8	10	1	17	101208.5037	0.0075	0.025	0.0076	0.50
547:	16	9	7	1	16	16	8	8	1	16	101221.8268	-0.0037	0.025	-0.0037	0.50
548:	16	9	8	1	16	16	8	9	1	16	101221.8268	-0.0037	0.025	-0.0037	0.50
549:	30	6	24	1	30	29	6	23	1	29	101226.9113	0.0235	0.025		
550:	15	9	6	1	15	15	8	7	1	15	101232.3262	-0.0048	0.025	-0.0049	0.50
551:	15	9	7	1	15	15	8	8	1	15	101232.3262	-0.0049	0.025	-0.0049	0.50
552:	14	9	5	1	14	14	8	6	1	14	101240.3687	-0.0032	0.025	-0.0032	0.50
553:	14	9	6	1	14	14	8	7	1	14	101240.3687	-0.0032	0.025	-0.0032	0.50
554:	13	9	4	1	13	13	8	5	1	13	101246.2936	-0.0093	0.025	-0.0094	0.50
555:	13	9	5	1	13	13	8	6	1	13	101246.2936	-0.0093	0.025	-0.0094	0.50
556:	12	9	3	1	12	12	8	4	1	12	101250.5101	0.0597	0.025	0.0598	0.50
557:	12	9	4	1	12	12	8	5	1	12	101250.5101	0.0597	0.025	0.0598	0.50
558:	11	9	2	1	11	11	8	3	1	11	101253.1107	-0.0056	0.025	-0.0057	0.50
559:	11	9	3	1	11	11	8	4	1	11	101253.1107	-0.0056	0.025	-0.0057	0.50
560:	10	9	1	1	10	10	8	2	1	10	101254.5618	-0.0183	0.025	-0.0183	0.50
561:	10	9	2	1	10	10	8	3	1	10	101254.5618	-0.0183	0.025	-0.0183	0.50
562:	30	3	28	1	30	29	2	27	1	29	101969.2453	0.0361	0.025		
563:	30	5	25	1	30	29	5	24	1	29	102114.1824	0.0005	0.025		

564:	31	3	29	1	31	30	3	28	1	30	102178.4800	0.0038	0.025		
565:	11	6	6	1	11	10	5	5	1	10	102358.9475	0.0367	0.025	-0.0044	0.50
566:	11	6	5	1	11	10	5	6	1	10	102358.9475	-0.0456	0.025	-0.0044	0.50
567:	30	3	27	1	30	29	3	26	1	29	102654.9793	0.0035	0.025		
568:	31	2	29	1	31	30	2	28	1	30	102675.6806	0.0042	0.025		
569:	32	1	31	1	32	31	2	30	1	31	102911.3123	0.0092	0.025		
570:	32	2	31	1	32	31	2	30	1	31	102998.1380	-0.0020	0.025		
571:	32	1	31	1	32	31	1	30	1	31	103028.9909	-0.0057	0.025		
572:	32	2	31	1	32	31	1	30	1	31	103115.8260	-0.0076	0.025		
573:	18	4	14	1	18	17	3	15	1	17	103124.2093	-0.0481	0.025		
574:	30	4	26	1	30	29	4	25	1	29	103449.3052	0.0076	0.025		
575:	15	5	11	1	15	14	4	10	1	14	103621.9662	0.0487	0.025		
576:	15	5	10	1	15	14	4	11	1	14	103709.1649	0.0196	0.025		
577:	33	0	33	1	33	32	1	32	1	32	103709.8233	-0.0282	0.025		
578:	33	1	33	1	33	32	1	32	1	32	103711.3297	-0.0076	0.025		
579:	33	0	33	1	33	32	0	32	1	32	103711.9425	-0.0098	0.025		
580:	33	1	33	1	33	32	0	32	1	32	103713.4383	0.0001	0.025		
581:	31	15	16	1	31	30	15	15	1	30	103849.5157	-0.0190	0.025	-0.0191	0.50
582:	31	15	17	1	31	30	15	16	1	30	103849.5157	-0.0190	0.025	-0.0191	0.50
583:	31	16	15	1	31	30	16	14	1	30	103850.0032	0.0396	0.025	0.0396	0.50
584:	31	16	16	1	31	30	16	15	1	30	103850.0032	0.0396	0.025	0.0396	0.50
585:	31	14	17	1	31	30	14	16	1	30	103854.4475	0.0349	0.025	0.0349	0.50
586:	31	14	18	1	31	30	14	17	1	30	103854.4475	0.0349	0.025	0.0349	0.50
587:	31	17	15	1	31	30	17	14	1	30	103854.7254	-0.0124	0.025	-0.0124	0.50
588:	31	17	14	1	31	30	17	13	1	30	103854.7254	-0.0124	0.025	-0.0124	0.50
589:	31	18	13	1	31	30	18	12	1	30	103863.1710	0.0139	0.025	0.0140	0.50
590:	31	18	14	1	31	30	18	13	1	30	103863.1710	0.0139	0.025	0.0140	0.50
591:	31	13	18	1	31	30	13	17	1	30	103865.9622	0.0141	0.025	0.0141	0.50
592:	31	13	19	1	31	30	13	18	1	30	103865.9622	0.0141	0.025	0.0141	0.50
593:	31	19	12	1	31	30	19	11	1	30	103874.7180	0.0181	0.025	0.0182	0.50
594:	31	19	13	1	31	30	19	12	1	30	103874.7180	0.0181	0.025	0.0182	0.50
595:	32	2	30	1	32	31	3	29	1	31	103885.5877	-0.0166	0.025		
596:	31	12	19	1	31	30	12	18	1	30	103886.1201	0.0259	0.025	0.0260	0.50
597:	31	12	20	1	31	30	12	19	1	30	103886.1201	0.0259	0.025	0.0260	0.50
598:	31	20	11	1	31	30	20	10	1	30	103888.9909	0.0202	0.025	0.0202	0.50
599:	31	20	12	1	31	30	20	11	1	30	103888.9909	0.0202	0.025	0.0202	0.50
600:	31	4	28	1	31	30	4	27	1	30	103891.3209	-0.0081	0.025		
601:	31	21	10	1	31	30	21	9	1	30	103905.6756	0.0113	0.025	0.0114	0.50
602:	31	21	11	1	31	30	21	10	1	30	103905.6756	0.0113	0.025	0.0114	0.50
603:	31	11	21	1	31	30	11	20	1	30	103917.7614	-0.0067	0.025	-0.0067	0.50
604:	31	11	20	1	31	30	11	19	1	30	103917.7614	-0.0067	0.025	-0.0067	0.50
605:	31	22	9	1	31	30	22	8	1	30	103924.5319	-0.0090	0.025	-0.0091	0.50
606:	31	22	10	1	31	30	22	9	1	30	103924.5319	-0.0090	0.025	-0.0091	0.50
607:	31	23	8	1	31	30	23	7	1	30	103945.4389	0.0286	0.025	0.0287	0.50
608:	31	23	9	1	31	30	23	8	1	30	103945.4389	0.0286	0.025	0.0287	0.50
609:	31	10	22	1	31	30	10	21	1	30	103965.5390	0.0317	0.025	0.0313	0.50
610:	31	10	21	1	31	30	10	20	1	30	103965.5390	0.0309	0.025	0.0313	0.50
611:	31	24	7	1	31	30	24	6	1	30	103968.1720	0.0539	0.025	0.0539	0.50
612:	31	24	8	1	31	30	24	7	1	30	103968.1720	0.0539	0.025	0.0539	0.50
613:	31	25	6	1	31	30	25	5	1	30	103992.5720	0.0334	0.025	0.0335	0.50
614:	31	25	7	1	31	30	25	6	1	30	103992.5720	0.0334	0.025	0.0335	0.50
615:	31	9	23	1	31	30	9	22	1	30	104036.7203	-0.0078	0.025	-0.0220	0.50
616:	31	9	22	1	31	30	9	21	1	30	104036.7203	-0.0360	0.025	-0.0220	0.50
617:	31	8	24	1	31	30	8	23	1	30	104144.1300	-0.0245	0.025		
618:	31	8	23	1	31	30	8	22	1	30	104144.8562	-0.0153	0.025		
619:	8	7	1	1	8	7	6	2	1	7	104236.1006	0.0388	0.025	0.0388	0.50
620:	8	7	2	1	8	7	6	1	1	7	104236.1006	0.0388	0.025	0.0388	0.50
621:	31	7	25	1	31	30	7	24	1	30	104307.6549	0.0052	0.025		
622:	31	7	24	1	31	30	7	23	1	30	104320.8349	-0.0125	0.025		
623:	31	3	29	1	31	30	2	28	1	30	104507.1190	0.0168	0.025		
624:	31	6	26	1	31	30	6	25	1	30	104523.5062	0.0215	0.025		
625:	31	5	27	1	31	30	5	26	1	30	104577.1385	0.0086	0.025		
626:	31	6	25	1	31	30	6	24	1	30	104687.4289	0.0258	0.025		
627:	32	3	30	1	32	31	3	29	1	31	105315.1890	0.0001	0.025		
628:	32	2	30	1	32	31	2	29	1	31	105717.0316	0.0014	0.025		
629:	31	5	26	1	31	30	5	25	1	30	105718.9694	0.0162	0.025		
630:	31	3	28	1	31	30	3	27	1	30	105805.7930	0.0139	0.025		
631:	33	1	32	1	33	32	2	31	1	32	106038.4546	-0.0149	0.025		
632:	33	2	32	1	33	32	2	31	1	32	106102.3360	-0.0058	0.025		
633:	33	1	32	1	33	32	1	31	1	32	106125.3001	-0.0064	0.025		
634:	34	3	31	1	34	33	4	30	1	33	106185.7436	-0.0575	0.025		
635:	33	2	32	1	33	32	1	31	1	32	106189.1634	-0.0154	0.025		
636:	34	0	34	1	34	33	1	33	1	33	106812.8391	-0.0612	0.025		
637:	34	1	34	1	34	33	1	33	1	33	106813.9122	-0.0377	0.025		
638:	34	0	34	1	34	33	0	33	1	33	106814.3890	0.0028	0.025		
639:	34	1	34	1	34	33	0	33	1	33	106815.3980	-0.0377	0.025		
640:	16	5	12	1	16	15	4	11	1	15	106869.3402	-0.0063	0.025		
641:	31	4	27	1	31	30	4	26	1	30	106961.3756	-0.0032	0.025		
642:	55	10	46	1	55	55	9	47	1	55	106999.0565	0.0535	0.025		
643:	16	5	11	1	16	15	4	12	1	15	107019.4726	0.0067	0.025		
644:	32	4	29	1	32	31	4	28	1	31	107132.3488	-0.0159	0.025		
645:	32	3	30	1	32	31	2	29	1	31	107146.5838	-0.0309	0.025		
646:	32	16	16	1	32	31	16	15	1	31	107201.8436	0.0037	0.025	0.0038	0.50

647:	32	16	17	1	32	31	16	16	1	31	107201.8436	0.0037	0.025	0.0038	0.50
648:	32	15	18	1	32	31	15	17	1	31	107202.6400	0.0123	0.025	0.0123	0.50
649:	32	15	17	1	32	31	15	16	1	31	107202.6400	0.0123	0.025	0.0123	0.50
650:	32	17	15	1	32	31	17	14	1	31	107205.7563	0.0042	0.025	0.0043	0.50
651:	32	17	16	1	32	31	17	15	1	31	107205.7563	0.0042	0.025	0.0043	0.50
652:	32	14	18	1	32	31	14	17	1	31	107209.1592	-0.0141	0.025	-0.0142	0.50
653:	32	14	19	1	32	31	14	18	1	31	107209.1592	-0.0141	0.025	-0.0142	0.50
654:	32	18	14	1	32	31	18	13	1	31	107213.5852	-0.0088	0.025	-0.0089	0.50
655:	32	18	15	1	32	31	18	14	1	31	107213.5852	-0.0088	0.025	-0.0089	0.50
656:	32	13	19	1	32	31	13	18	1	31	107222.9613	-0.0034	0.025	-0.0034	0.50
657:	32	13	20	1	32	31	13	19	1	31	107222.9613	-0.0034	0.025	-0.0034	0.50
658:	32	19	13	1	32	31	19	12	1	31	107224.8178	0.0248	0.025	0.0248	0.50
659:	32	19	14	1	32	31	19	13	1	31	107224.8178	0.0248	0.025	0.0248	0.50
660:	32	20	12	1	32	31	20	11	1	31	107238.9495	0.0352	0.025	0.0352	0.50
661:	32	20	13	1	32	31	20	12	1	31	107238.9495	0.0352	0.025	0.0352	0.50
662:	32	12	20	1	32	31	12	19	1	31	107246.1622	0.0078	0.025	0.0078	0.50
663:	32	12	21	1	32	31	12	20	1	31	107246.1622	0.0078	0.025	0.0078	0.50
664:	32	21	11	1	32	31	21	10	1	31	107255.6406	0.0178	0.025	0.0179	0.50
665:	32	21	12	1	32	31	21	11	1	31	107255.6406	0.0178	0.025	0.0179	0.50
666:	32	22	10	1	32	31	22	9	1	31	107274.6619	0.0061	0.025	0.0061	0.50
667:	32	22	11	1	32	31	22	10	1	31	107274.6619	0.0061	0.025	0.0061	0.50
668:	32	11	21	1	32	31	11	20	1	31	107281.9410	-0.0220	0.025	-0.0221	0.50
669:	32	11	22	1	32	31	11	21	1	31	107281.9410	-0.0220	0.025	-0.0221	0.50
670:	32	23	9	1	32	31	23	8	1	31	107295.8113	0.0068	0.025	0.0069	0.50
671:	32	23	10	1	32	31	23	9	1	31	107295.8113	0.0068	0.025	0.0069	0.50
672:	32	24	8	1	32	31	24	7	1	31	107318.9055	0.0053	0.025	0.0053	0.50
673:	32	24	9	1	32	31	24	8	1	31	107318.9055	0.0053	0.025	0.0053	0.50
674:	32	10	23	1	32	31	10	22	1	31	107335.4130	0.0029	0.025	0.0021	0.50
675:	32	10	22	1	32	31	10	21	1	31	107335.4130	0.0013	0.025	0.0021	0.50
676:	33	2	31	1	33	32	3	30	1	32	107336.0205	-0.0228	0.025		
677:	32	25	7	1	32	31	25	6	1	31	107343.8090	0.0038	0.025	0.0039	0.50
678:	32	25	8	1	32	31	25	7	1	31	107343.8090	0.0038	0.025	0.0039	0.50
679:	32	26	6	1	32	31	26	5	1	31	107370.4177	0.0124	0.025	0.0124	0.50
680:	32	26	7	1	32	31	26	6	1	31	107370.4177	0.0124	0.025	0.0124	0.50
681:	32	27	5	1	32	31	27	4	1	31	107398.6198	0.0148	0.025	0.0148	0.50
682:	32	27	6	1	32	31	27	5	1	31	107398.6198	0.0148	0.025	0.0148	0.50
683:	32	9	24	1	32	31	9	23	1	31	107414.7540	0.0315	0.025	0.0071	0.50
684:	32	9	23	1	32	31	9	22	1	31	107414.7540	-0.0172	0.025	0.0071	0.50
685:	32	28	4	1	32	31	28	3	1	31	107428.3071	-0.0160	0.025	-0.0161	0.50
686:	32	28	5	1	32	31	28	4	1	31	107428.3071	-0.0160	0.025	-0.0161	0.50
687:	32	29	3	1	32	31	29	2	1	31	107459.4944	0.0041	0.025	0.0042	0.50
688:	32	29	4	1	32	31	29	3	1	31	107459.4944	0.0041	0.025	0.0042	0.50
689:	32	8	25	1	32	31	8	24	1	31	107533.9660	-0.0024	0.025		
690:	32	8	24	1	32	31	8	23	1	31	107535.0795	-0.0392	0.025		
691:	9	7	2	1	9	8	6	3	1	8	107583.0103	0.0252	0.025	0.0253	0.50
692:	9	7	3	1	9	8	6	2	1	8	107583.0103	0.0252	0.025	0.0253	0.50
693:	33	3	31	1	33	32	3	30	1	32	108444.4613	-0.0131	0.025		
694:	33	2	31	1	33	32	2	30	1	32	108765.6226	-0.0053	0.025		
695:	32	3	29	1	32	31	3	28	1	31	108914.2800	0.0137	0.025		
696:	50	10	41	1	50	50	9	42	1	50	108955.1044	-0.0473	0.025		
697:	13	6	8	1	13	12	5	7	1	12	109032.3312	-0.0156	0.025		
698:	13	6	7	1	13	12	5	8	1	12	109032.8766	-0.0048	0.025		
699:	49	10	39	1	49	49	9	40	1	49	109122.6819	-0.0137	0.025	-0.0137	0.50
700:	49	10	39	1	49	49	9	40	1	49	109122.6819	-0.0137	0.025	-0.0137	0.50
701:	34	1	33	1	34	33	2	32	1	33	109158.6331	-0.0158	0.025		
702:	34	2	33	1	34	33	2	32	1	33	109205.5080	0.0117	0.025		
703:	34	1	33	1	34	33	1	32	1	33	109222.5055	-0.0157	0.025		
704:	34	2	33	1	34	33	1	32	1	33	109269.3681	-0.0003	0.025		
705:	32	5	27	1	32	31	5	26	1	31	109343.0550	0.0379	0.025		
706:	47	10	37	1	47	47	9	38	1	47	109836.6679	-0.0053	0.025		
707:	33	3	31	1	33	32	2	30	1	32	109873.9871	-0.0719	0.025		
708:	35	0	35	1	35	34	1	34	1	34	109915.4450	-0.0236	0.025		
709:	35	1	35	1	35	34	1	34	1	34	109916.1600	-0.0491	0.025	-0.0492	0.50
710:	35	1	35	1	35	34	1	34	1	34	109916.1600	-0.0491	0.025	-0.0492	0.50
711:	35	0	35	1	35	34	0	34	1	34	109916.5051	-0.0131	0.025		
712:	35	1	35	1	35	34	0	34	1	34	109917.2659	0.0071	0.025		
713:	32	4	28	1	32	31	4	27	1	31	110434.0136	0.0010	0.025		
714:	33	16	17	1	33	32	16	16	1	32	110553.8784	-0.0027	0.025	-0.0027	0.50
715:	33	16	18	1	33	32	16	17	1	32	110553.8784	-0.0027	0.025	-0.0027	0.50
716:	33	15	18	1	33	32	15	17	1	32	110556.0076	0.0056	0.025	0.0056	0.50
717:	33	15	19	1	33	32	15	18	1	32	110556.0076	0.0056	0.025	0.0056	0.50
718:	33	17	16	1	33	32	17	15	1	32	110556.8410	0.0053	0.025	0.0054	0.50
719:	33	17	17	1	33	32	17	16	1	32	110556.8410	0.0053	0.025	0.0054	0.50
720:	33	18	15	1	33	32	18	14	1	32	110564.0081	-0.0126	0.025	-0.0127	0.50
721:	33	18	16	1	33	32	18	15	1	32	110564.0081	-0.0126	0.025	-0.0127	0.50
722:	33	14	19	1	33	32	14	18	1	32	110564.3786	0.0197	0.025	0.0197	0.50
723:	33	14	20	1	33	32	14	19	1	32	110564.3786	0.0197	0.025	0.0197	0.50
724:	33	19	14	1	33	32	19	13	1	32	110574.8239	0.0153	0.025	0.0153	0.50
725:	33	19	15	1	33	32	19	14	1	32	110574.8239	0.0153	0.025	0.0153	0.50
726:	33	13	20	1	33	32	13	19	1	32	110580.5797	-0.0061	0.025	-0.0062	0.50
727:	33	13	21	1	33	32	13	20	1	32	110580.5797	-0.0061	0.025	-0.0062	0.50
728:	33	20	13	1	33	32	20	12	1	32	110588.7107	-0.0124	0.025	-0.0124	0.50
729:	33	20	14	1	33	32	20	13	1	32	110588.7107	-0.0124	0.025	-0.0124	0.50

730:	33 21 12	1 33	32 21 11	1 32	110605.3944	-0.0031	0.025	-0.0032	0.50
731:	33 21 13	1 33	32 21 12	1 32	110605.3944	-0.0031	0.025	-0.0032	0.50
732:	33 12 21	1 33	32 12 20	1 32	110607.0519	0.0019	0.025	0.0020	0.50
733:	33 12 22	1 33	32 12 21	1 32	110607.0519	0.0019	0.025	0.0020	0.50
734:	33 22 11	1 33	32 22 10	1 32	110624.5461	0.0015	0.025	0.0015	0.50
735:	33 22 12	1 33	32 22 11	1 32	110624.5461	0.0015	0.025	0.0015	0.50
736:	33 23 10	1 33	32 23 9	1 32	110645.9378	0.0019	0.025	0.0020	0.50
737:	33 23 11	1 33	32 23 10	1 32	110645.9378	0.0019	0.025	0.0020	0.50
738:	33 11 22	1 33	32 11 21	1 32	110647.2934	-0.0042	0.025	-0.0042	0.50
739:	33 11 23	1 33	32 11 22	1 32	110647.2934	-0.0041	0.025	-0.0042	0.50
740:	33 24 9	1 33	32 24 8	1 32	110669.3956	0.0084	0.025	0.0084	0.50
741:	33 24 10	1 33	32 24 9	1 32	110669.3956	0.0084	0.025	0.0084	0.50
742:	44 10 34	1 44	44 9 35	1 44	110694.2859	0.0194	0.025		
743:	33 25 8	1 33	32 25 7	1 32	110694.7370	-0.0113	0.025	-0.0113	0.50
744:	33 25 9	1 33	32 25 8	1 32	110694.7370	-0.0113	0.025	-0.0113	0.50
745:	43 10 33	1 43	43 9 34	1 43	110934.0377	0.0131	0.025		
746:	33 10 24	1 33	32 10 23	1 32	110706.8661	-0.0028	0.025	-0.0043	0.50
747:	33 10 23	1 33	32 10 22	1 32	110706.8661	-0.0056	0.025	-0.0043	0.50
748:	33 27 6	1 33	32 27 5	1 32	110750.7268	0.0037	0.025	0.0037	0.50
749:	33 27 7	1 33	32 27 6	1 32	110750.7268	0.0037	0.025	0.0037	0.50
750:	33 9 25	1 33	32 9 24	1 32	110794.9091	0.0402	0.025	-0.0010	0.50
751:	33 9 24	1 33	32 9 23	1 32	110794.9091	-0.0421	0.025	-0.0010	0.50
752:	33 8 26	1 33	32 8 25	1 32	110926.7860	0.0115	0.025		
753:	33 8 25	1 33	32 8 24	1 32	110928.5705	-0.0188	0.025		
754:	33 7 27	1 33	32 7 26	1 32	111123.3501	0.0072	0.025		
755:	33 7 26	1 33	32 7 25	1 32	111152.2594	-0.0013	0.025		
756:	33 5 29	1 33	32 5 28	1 32	111295.9384	0.0036	0.025		
757:	33 6 28	1 33	32 6 27	1 32	111352.3849	0.0121	0.025		
758:	34 3 32	1 34	33 3 31	1 33	111567.3725	-0.0144	0.025		
759:	33 6 27	1 33	32 6 26	1 32	111657.7534	0.0120	0.025		
760:	34 2 32	1 34	33 2 31	1 33	111821.5486	-0.0076	0.025		
761:	33 3 30	1 33	32 3 29	1 32	111985.9908	-0.0316	0.025		
762:	35 10 26	1 35	35 9 27	1 35	112257.7047	0.0412	0.025		
763:	35 10 25	1 35	35 9 26	1 35	112257.1362	0.0077	0.025		
764:	35 1 34	1 35	34 2 33	1 34	112273.4986	-0.0128	0.025		
765:	35 2 34	1 35	34 2 33	1 34	112307.7971	0.0153	0.025		
766:	35 1 34	1 35	34 1 33	1 34	112320.3538	-0.0048	0.025		
767:	35 2 34	1 35	34 1 33	1 34	112354.6213	-0.0077	0.025		
768:	33 10 23	1 33	33 9 24	1 33	112462.1270	0.0638	0.025	-0.0304	0.50
769:	33 10 24	1 33	33 9 25	1 33	112462.1270	-0.1246	0.025	-0.0304	0.50
770:	32 10 22	1 32	32 9 23	1 32	112550.1933	0.0506	0.025	-0.0038	0.50
771:	32 10 23	1 32	32 9 24	1 32	112550.1933	-0.0582	0.025	-0.0038	0.50
772:	31 10 21	1 31	31 9 22	1 31	112629.5229	0.0206	0.025	-0.0102	0.50
773:	31 10 22	1 31	31 9 23	1 31	112629.5229	-0.0410	0.025	-0.0102	0.50
774:	34 3 32	1 34	33 2 31	1 33	112675.8156	-0.0024	0.025		
775:	30 10 20	1 30	30 9 21	1 30	112700.7726	0.0220	0.025	0.0049	0.50
776:	30 10 21	1 30	30 9 22	1 30	112700.7726	-0.0122	0.025	0.0049	0.50
777:	29 10 19	1 29	29 9 20	1 29	112764.4729	0.0086	0.025	-0.0007	0.50
778:	29 10 20	1 29	29 9 21	1 29	112764.4729	-0.0100	0.025	-0.0007	0.50
779:	28 10 18	1 28	28 9 19	1 28	112821.2038	0.0127	0.025	0.0078	0.50
780:	28 10 19	1 28	28 9 20	1 28	112821.2038	0.0028	0.025	0.0078	0.50
781:	27 10 17	1 27	27 9 18	1 27	112871.4663	0.0148	0.025	0.0123	0.50
782:	27 10 18	1 27	27 9 19	1 27	112871.4663	0.0097	0.025	0.0123	0.50
783:	26 10 16	1 26	26 9 17	1 26	112915.7539	0.0128	0.025	0.0116	0.50
784:	26 10 17	1 26	26 9 18	1 26	112915.7539	0.0102	0.025	0.0116	0.50
785:	25 10 15	1 25	25 9 16	1 25	112954.5283	-0.0036	0.025	-0.0043	0.50
786:	25 10 16	1 25	25 9 17	1 25	112954.5283	-0.0049	0.025	-0.0043	0.50
787:	33 5 28	1 33	32 5 27	1 32	112980.0483	0.0100	0.025		
788:	24 10 14	1 24	24 9 15	1 24	112988.2788	0.0048	0.025	0.0045	0.50
789:	24 10 15	1 24	24 9 16	1 24	112988.2788	0.0042	0.025	0.0045	0.50
790:	23 10 13	1 23	23 9 14	1 23	113017.4765	0.0808	0.025	-0.0351	0.20
791:	23 10 14	1 23	23 9 15	1 23	113017.4765	0.0805	0.025	-0.0351	0.20
792:	36 0 36	1 36	35 1 35	1 35	113017.4765	-0.1123	0.025	-0.0351	0.60
793:	36 1 36	1 36	35 1 35	1 35	113018.2364	0.1256	0.025	0.0163	0.50
794:	36 0 36	1 36	35 0 35	1 35	113018.2364	-0.0929	0.025	0.0163	0.50
795:	36 1 36	1 36	35 0 35	1 35	113018.8478	-0.0034	0.025		
796:	34 4 31	1 34	33 4 30	1 33	113567.3270	0.0064	0.025		
797:	33 4 29	1 33	32 4 28	1 32	113863.2917	0.0368	0.025		
798:	34 16 18	1 34	33 16 17	1 33	113906.0935	0.0027	0.025	0.0027	0.50
799:	34 16 19	1 34	33 16 18	1 33	113906.0935	0.0027	0.025	0.0027	0.50
800:	34 17 17	1 34	33 17 16	1 33	113907.9947	0.0057	0.025	0.0057	0.50
801:	34 17 18	1 34	33 17 17	1 33	113907.9947	0.0057	0.025	0.0057	0.50
802:	34 15 19	1 34	33 15 18	1 33	113909.6716	0.0071	0.025	0.0071	0.50
803:	34 15 20	1 34	33 15 19	1 33	113909.6716	0.0071	0.025	0.0071	0.50
804:	34 18 16	1 34	33 18 15	1 33	113914.4386	0.0035	0.025	0.0035	0.50
805:	34 18 17	1 34	33 18 16	1 33	113914.4386	0.0035	0.025	0.0035	0.50
806:	34 14 20	1 34	33 14 19	1 33	113919.9892	0.0086	0.025	0.0087	0.50
807:	34 14 21	1 34	33 14 20	1 33	113919.9892	0.0086	0.025	0.0087	0.50
808:	34 19 15	1 34	33 19 14	1 33	113924.7490	0.0064	0.025	0.0064	0.50
809:	34 19 16	1 34	33 19 15	1 33	113924.7490	0.0064	0.025	0.0064	0.50
810:	34 13 21	1 34	33 13 20	1 33	113938.8194	-0.0097	0.025	-0.0097	0.50
811:	34 13 22	1 34	33 13 21	1 33	113938.8194	-0.0097	0.025	-0.0097	0.50
812:	34 21 13	1 34	33 21 12	1 33	113954.9876	0.0059	0.025	0.0060	0.50

813:	34	21	14	1	34	33	21	13	1	33	113954.9876	0.0059	0.025	0.0060	0.50
814:	34	12	22	1	34	33	12	21	1	33	113968.8141	0.0076	0.025	0.0077	0.50
815:	34	12	23	1	34	33	12	22	1	33	113968.8141	0.0076	0.025	0.0077	0.50
816:	34	22	12	1	34	33	22	11	1	33	113974.2087	0.0096	0.025	0.0096	0.50
817:	34	22	13	1	34	33	22	12	1	33	113974.2087	0.0096	0.025	0.0096	0.50
818:	34	23	11	1	34	33	23	10	1	33	113995.8007	0.0057	0.025	0.0057	0.50
819:	34	23	12	1	34	33	23	11	1	33	113995.8007	0.0057	0.025	0.0057	0.50
820:	34	11	24	1	34	33	11	23	1	33	114013.8146	0.0055	0.025	0.0054	0.50
821:	34	11	23	1	34	33	11	22	1	33	114013.8146	0.0053	0.025	0.0054	0.50
822:	34	24	10	1	34	33	24	9	1	33	114019.5720	0.0031	0.025	0.0031	0.50
823:	34	24	11	1	34	33	24	10	1	33	114019.5720	0.0031	0.025	0.0031	0.50
824:	35	2	33	1	35	34	3	32	1	34	114030.0353	-0.0451	0.025		
825:	34	25	9	1	34	33	25	8	1	33	114045.3648	0.0077	0.025	0.0077	0.50
826:	34	25	10	1	34	33	25	9	1	33	114045.3648	0.0077	0.025	0.0077	0.50
827:	34	10	25	1	34	33	10	24	1	33	114079.9463	0.0068	0.025	0.0043	0.50
828:	34	10	24	1	34	33	10	23	1	33	114079.9463	0.0018	0.025	0.0043	0.50
829:	34	9	25	1	34	33	9	24	1	33	114177.3273	-0.0606	0.025	0.0078	0.50
830:	34	9	26	1	34	33	9	25	1	33	114177.3273	0.0762	0.025	0.0078	0.50
831:	34	8	27	1	34	33	8	26	1	33	114322.6763	0.0041	0.025		
832:	34	8	26	1	34	33	8	25	1	33	114325.5000	0.0089	0.025		
833:	34	7	28	1	34	33	7	27	1	33	114536.2656	-0.0131	0.025		
834:	34	7	27	1	34	33	7	26	1	33	114578.1717	0.0210	0.025		
835:	34	5	30	1	34	33	5	29	1	33	114636.5058	-0.0026	0.025		
836:	35	3	33	1	35	34	3	32	1	34	114684.8935	-0.0063	0.025		
837:	34	6	29	1	34	33	6	28	1	33	114765.0663	0.0017	0.025		
838:	35	2	33	1	35	34	2	32	1	34	114884.3251	-0.0171	0.025		
839:	34	3	31	1	34	33	3	30	1	33	115028.3982	0.0489	0.025		
840:	34	6	28	1	34	33	6	27	1	33	115172.3432	-0.0029	0.025		
841:	36	1	35	1	36	35	2	34	1	35	115384.3147	-0.0093	0.025		
842:	36	2	35	1	36	35	2	34	1	35	115409.3253	-0.0081	0.025		
843:	36	1	35	1	36	35	1	34	1	35	115418.5861	-0.0083	0.025		
844:	36	2	35	1	36	35	1	34	1	35	115443.5935	-0.0102	0.025		
845:	35	3	33	1	35	34	2	32	1	34	115539.1544	-0.0073	0.025		
846:	37	0	37	1	37	36	1	36	1	36	116119.2298	-0.0512	0.025		
847:	37	1	37	1	37	36	1	36	1	36	116119.7173	0.0687	0.025	-0.0084	0.50
848:	37	0	37	1	37	36	0	36	1	36	116119.7173	-0.0856	0.025	-0.0084	0.50
849:	37	1	37	1	37	36	0	36	1	36	116120.1932	0.0227	0.025		
850:	19	5	15	1	19	18	4	14	1	18	116347.2163	-0.0150	0.025		
851:	34	5	29	1	34	33	5	28	1	33	116621.7471	0.0139	0.025		
852:	35	4	32	1	35	34	4	31	1	34	116762.3538	-0.0034	0.025		
853:	19	5	14	1	19	18	4	15	1	18	116967.9412	0.0157	0.025		
854:	34	4	30	1	34	33	4	29	1	33	117245.9452	-0.0100	0.025	-0.0101	0.50
855:	34	4	30	1	34	33	4	29	1	33	117245.9452	-0.0100	0.025	-0.0101	0.50
856:	35	16	19	1	35	34	16	18	1	34	117258.4770	0.0050	0.025	0.0050	0.50
857:	35	16	20	1	35	34	16	19	1	34	117258.4770	0.0050	0.025	0.0050	0.50
858:	35	17	18	1	35	34	17	17	1	34	117259.2232	0.0108	0.025	0.0109	0.50
859:	35	17	19	1	35	34	17	18	1	34	117259.2232	0.0108	0.025	0.0109	0.50
860:	35	15	20	1	35	34	15	19	1	34	117263.6368	0.0148	0.025	0.0148	0.50
861:	35	15	21	1	35	34	15	20	1	34	117263.6368	0.0148	0.025	0.0148	0.50
862:	35	18	17	1	35	34	18	16	1	34	117264.8494	0.0145	0.025	0.0146	0.50
863:	35	18	18	1	35	34	18	17	1	34	117264.8494	0.0145	0.025	0.0146	0.50
864:	35	19	16	1	35	34	19	15	1	34	117274.5842	-0.0067	0.025	-0.0067	0.50
865:	35	19	17	1	35	34	19	16	1	34	117274.5842	-0.0067	0.025	-0.0067	0.50
866:	35	14	21	1	35	34	14	20	1	34	117276.0671	0.0174	0.025	0.0174	0.50
867:	35	14	22	1	35	34	14	21	1	34	117276.0671	0.0174	0.025	0.0174	0.50
868:	35	20	15	1	35	34	20	14	1	34	117287.9143	0.0003	0.025	0.0003	0.50
869:	35	20	16	1	35	34	20	15	1	34	117287.9143	0.0003	0.025	0.0003	0.50
870:	35	13	22	1	35	34	13	21	1	34	117297.7108	-0.0010	0.025	-0.0011	0.50
871:	35	13	23	1	35	34	13	22	1	34	117297.7108	-0.0010	0.025	-0.0011	0.50
872:	36	2	34	1	36	35	3	33	1	35	117298.3865	-0.0109	0.025		
873:	35	21	14	1	35	34	21	13	1	34	117304.3592	-0.0085	0.025	-0.0085	0.50
874:	35	21	15	1	35	34	21	14	1	34	117304.3592	-0.0085	0.025	-0.0085	0.50
875:	35	22	13	1	35	34	22	12	1	34	117323.5999	-0.0109	0.025	-0.0110	0.50
876:	35	22	14	1	35	34	22	13	1	34	117323.5999	-0.0109	0.025	-0.0110	0.50
877:	35	12	23	1	35	34	12	22	1	34	117331.4467	-0.0031	0.025	-0.0031	0.50
878:	35	12	24	1	35	34	12	23	1	34	117331.4467	-0.0031	0.025	-0.0031	0.50
879:	35	23	12	1	35	34	23	11	1	34	117345.3291	-0.0434	0.025	-0.0435	0.50
880:	35	23	13	1	35	34	23	12	1	34	117345.3291	-0.0434	0.025	-0.0435	0.50
881:	35	24	11	1	35	34	24	10	1	34	117369.4377	0.0027	0.025	0.0027	0.50
882:	35	24	12	1	35	34	24	11	1	34	117369.4377	0.0027	0.025	0.0027	0.50
883:	35	11	25	1	35	34	11	24	1	34	117381.5447	0.0088	0.025	0.0087	0.50
884:	35	11	24	1	35	34	11	23	1	34	117381.5447	0.0085	0.025	0.0087	0.50
885:	35	25	10	1	35	34	25	9	1	34	117395.6404	0.0200	0.025	0.0201	0.50
886:	35	25	11	1	35	34	25	10	1	34	117395.6404	0.0200	0.025	0.0201	0.50
887:	35	26	9	1	35	34	26	8	1	34	117423.8103	0.0281	0.025	0.0282	0.50
888:	35	26	10	1	35	34	26	9	1	34	117423.8103	0.0281	0.025	0.0282	0.50
889:	35	27	8	1	35	34	27	7	1	34	117453.7914	-0.0065	0.025	-0.0065	0.50
890:	35	27	9	1	35	34	27	8	1	34	117453.7914	-0.0065	0.025	-0.0065	0.50
891:	35	10	26	1	35	34	10	25	1	34	117454.6988	0.0200	0.025	0.0156	0.50
892:	35	10	25	1	35	34	10	24	1	34	117454.6988	0.0112	0.025	0.0156	0.50
893:	35	28	7	1	35	34	28	6	1	34	117485.5559	-0.0084	0.025	-0.0084	0.50
894:	35	28	8	1	35	34	28	7	1	34	117485.5559	-0.0084	0.025	-0.0084	0.50
895:	35	29	6	1	35	34	29	5	1	34	117518.9957	0.0026	0.025	0.0026	0.50

896:	35	29	7	1	35	34	29	6	1	34	117518.9957	0.0026	0.025	0.0026	0.50
897:	35	9	26	1	35	34	9	25	1	34	117562.1056	-0.0732	0.025		
898:	12	7	6	1	12	11	6	5	1	11	117618.8205	-0.0138	0.025	-0.0149	0.50
899:	12	7	5	1	12	11	6	6	1	11	117618.8205	-0.0159	0.025	-0.0149	0.50
900:	35	8	28	1	35	34	8	27	1	34	117721.7695	0.0177	0.025		
901:	35	8	27	1	35	34	8	26	1	34	117726.0740	0.0087	0.025		
902:	36	3	34	1	36	35	3	33	1	35	117797.8995	0.0095	0.025		
903:	35	7	29	1	35	34	7	28	1	34	117952.3306	0.0141	0.025		
904:	36	2	34	1	36	35	2	33	1	35	117953.2133	-0.0036	0.025		
905:	35	5	31	1	35	34	5	30	1	34	117962.8449	0.0024	0.025		
906:	35	7	28	1	35	34	7	27	1	34	118012.1065	0.0006	0.025		
907:	35	3	32	1	35	34	3	31	1	34	118049.5255	-0.0049	0.025		
908:	35	6	30	1	35	34	6	29	1	34	118174.8213	0.0062	0.025		
909:	36	3	34	1	36	35	2	33	1	35	118452.6994	-0.0100	0.025		
910:	37	1	36	1	37	36	2	35	1	36	118492.0382	-0.0031	0.025		
911:	37	2	36	1	37	36	2	35	1	36	118510.2402	-0.0112	0.025		
912:	37	1	36	1	37	36	1	35	1	36	118517.0449	-0.0057	0.025		
913:	37	2	36	1	37	36	1	35	1	36	118535.2553	-0.0054	0.025		
914:	35	6	29	1	35	34	6	28	1	34	118709.6790	0.0119	0.025		
915:	37	3	34	1	37	36	4	33	1	36	119080.9834	0.0142	0.025		
916:	38	1	38	1	38	37	1	37	1	37	119220.8538	0.0387	0.025	-0.0157	0.50
917:	38	0	38	1	38	37	0	37	1	37	119220.8538	-0.0702	0.025	-0.0157	0.50
918:	38	1	38	1	38	37	0	37	1	37	119221.2527	0.0701	0.025		
919:	36	4	33	1	36	35	4	32	1	35	119943.5137	0.0156	0.025		
920:	35	5	30	1	35	34	5	29	1	34	120258.5890	0.0048	0.025		
921:	20	5	15	1	20	19	4	16	1	19	120311.5532	0.0145	0.025		
922:	37	2	35	1	37	36	3	34	1	36	120527.8272	0.0218	0.025		
923:	35	4	31	1	35	34	4	30	1	34	120579.6164	-0.0114	0.025		
924:	36	17	19	1	36	35	17	18	1	35	120610.5107	0.0049	0.025	0.0050	0.50
925:	36	17	20	1	36	35	17	19	1	35	120610.5107	0.0049	0.025	0.0050	0.50
926:	36	16	20	1	36	35	16	19	1	35	120611.0204	-0.0072	0.025	-0.0072	0.50
927:	36	16	21	1	36	35	16	20	1	35	120611.0204	-0.0072	0.025	-0.0072	0.50
928:	36	18	19	1	36	35	18	18	1	35	120615.2226	0.0047	0.025	0.0048	0.50
929:	36	18	18	1	36	35	18	17	1	35	120615.2226	0.0047	0.025	0.0048	0.50
930:	36	15	21	1	36	35	15	20	1	35	120617.8799	-0.0011	0.025	-0.0012	0.50
931:	36	15	22	1	36	35	15	21	1	35	120617.8799	-0.0011	0.025	-0.0012	0.50
932:	36	19	17	1	36	35	19	16	1	35	120624.3531	0.0037	0.025	0.0037	0.50
933:	36	19	18	1	36	35	19	17	1	35	120624.3531	0.0037	0.025	0.0037	0.50
934:	36	14	23	1	36	35	14	22	1	35	120632.5836	0.0058	0.025	0.0058	0.50
935:	36	14	22	1	36	35	14	21	1	35	120632.5836	0.0058	0.025	0.0058	0.50
936:	36	20	16	1	36	35	20	15	1	35	120637.2807	-0.0036	0.025	-0.0037	0.50
937:	36	20	17	1	36	35	20	16	1	35	120637.2807	-0.0036	0.025	-0.0037	0.50
938:	36	21	16	1	36	35	21	15	1	35	120653.5564	0.0078	0.025	0.0078	0.50
939:	36	21	15	1	36	35	21	14	1	35	120653.5564	0.0078	0.025	0.0078	0.50
940:	36	13	23	1	36	35	13	22	1	35	120657.2630	0.0111	0.025	0.0112	0.50
941:	36	13	24	1	36	35	13	23	1	35	120657.2630	0.0111	0.025	0.0112	0.50
942:	36	22	14	1	36	35	22	13	1	35	120672.7871	0.0156	0.025	0.0156	0.50
943:	36	22	15	1	36	35	22	14	1	35	120672.7871	0.0156	0.025	0.0156	0.50
944:	36	23	13	1	36	35	23	12	1	35	120694.6409	-0.0181	0.025	-0.0182	0.50
945:	36	23	14	1	36	35	23	13	1	35	120694.6409	-0.0181	0.025	-0.0182	0.50
946:	36	12	25	1	36	35	12	24	1	35	120695.0183	0.0119	0.025	0.0120	0.50
947:	36	12	24	1	36	35	12	23	1	35	120695.0183	0.0119	0.025	0.0120	0.50
948:	36	24	12	1	36	35	24	11	1	35	120718.9822	0.0071	0.025	0.0072	0.50
949:	36	24	13	1	36	35	24	12	1	35	120718.9822	0.0071	0.025	0.0072	0.50
950:	36	25	11	1	36	35	25	10	1	35	120745.5304	0.0035	0.025	0.0036	0.50
951:	36	25	12	1	36	35	25	11	1	35	120745.5304	0.0035	0.025	0.0036	0.50
952:	36	11	26	1	36	35	11	25	1	35	120750.4989	-0.0179	0.025	-0.0182	0.50
953:	36	11	25	1	36	35	11	24	1	35	120750.4989	-0.0184	0.025	-0.0182	0.50
954:	36	10	26	1	36	35	10	25	1	35	120831.1741	0.0130	0.025		
955:	54	11	43	1	54	54	10	44	1	54	120869.7298	0.0101	0.025		
956:	36	29	7	1	36	35	29	6	1	35	120871.2834	0.0017	0.025	0.0018	0.50
957:	36	29	8	1	36	35	29	7	1	35	120871.2834	0.0017	0.025	0.0018	0.50
958:	37	3	35	1	37	36	3	34	1	36	120907.1185	-0.0111	0.025		
959:	36	9	27	1	36	35	9	26	1	35	120949.4348	0.0052	0.025		
960:	37	2	35	1	37	36	2	34	1	36	121027.2983	0.0004	0.025		
961:	36	3	33	1	36	35	3	32	1	35	121057.8940	0.0005	0.025		
962:	36	8	29	1	36	35	8	28	1	35	121124.0968	0.0077	0.025		
963:	36	8	28	1	36	35	8	27	1	35	121130.6071	0.0104	0.025		
964:	36	5	32	1	36	35	5	31	1	35	121273.9124	-0.0095	0.025		
965:	36	7	30	1	36	35	7	29	1	35	121371.1233	0.0174	0.025		
966:	37	3	35	1	37	36	2	34	1	36	121406.6183	-0.0038	0.025	-0.0039	0.50
967:	37	3	35	1	37	36	2	34	1	36	121406.6183	-0.0038	0.025	-0.0039	0.50
968:	36	7	29	1	36	35	7	28	1	35	121455.3443	0.0084	0.025		
969:	36	6	31	1	36	35	6	30	1	35	121580.1481	0.0133	0.025		
970:	38	1	37	1	38	37	2	36	1	37	121597.3614	-0.0155	0.025		
971:	38	2	37	1	38	37	2	36	1	37	121610.5888	-0.0200	0.025		
972:	38	1	37	1	38	37	1	36	1	37	121615.5920	0.0049	0.025		
973:	38	2	37	1	38	37	1	36	1	37	121628.8012	-0.0177	0.025		
974:	51	11	40	1	51	51	10	41	1	51	121831.2080	0.0286	0.025		
975:	50	11	39	1	50	50	10	40	1	50	122105.7600	0.0721	0.025		
976:	36	6	30	1	36	35	6	29	1	35	122271.5513	0.0285	0.025		
977:	21	5	17	1	21	20	4	16	1	20	122299.5680	0.0239	0.025		
978:	17	6	11	1	17	16	5	12	1	16	122319.9715	0.0048	0.025		

979:	39	1	39	1	39	38	1	38	1	38	122321.6187	0.0166	0.025	-0.0218 0.50
980:	39	0	39	1	39	38	0	38	1	38	122321.6187	-0.0601	0.025	-0.0218 0.50
981:	34	4	31	1	34	33	3	30	1	33	122409.8897	0.0209	0.025	
982:	10	8	2	1	10	9	7	3	1	9	122830.7108	0.0211	0.025	0.0211 0.50
983:	10	8	3	1	10	9	7	2	1	9	122830.7108	0.0211	0.025	0.0211 0.50
984:	37	4	34	1	37	36	4	33	1	36	123111.7401	0.0095	0.025	
985:	42	11	31	1	42	42	10	32	1	42	123690.0709	-0.0534	0.025	
986:	42	11	32	1	42	42	10	33	1	42	123690.7819	-0.0432	0.025	
987:	38	2	36	1	38	37	3	35	1	37	123726.4345	0.0543	0.025	
988:	41	11	31	1	41	41	10	32	1	41	123827.6254	0.0482	0.025	
989:	36	4	32	1	36	35	4	31	1	35	123862.5380	0.0373	0.025	
990:	36	5	31	1	36	35	5	30	1	35	123880.7748	0.0164	0.025	
991:	37	19	18	1	37	36	19	17	1	36	123974.0177	0.0040	0.025	0.0041 0.50
992:	37	19	19	1	37	36	19	18	1	36	123974.0177	0.0040	0.025	0.0041 0.50
993:	37	17	20	1	37	36	17	19	1	36	123961.8778	0.0085	0.025	0.0086 0.50
994:	37	17	21	1	37	36	17	20	1	36	123961.8778	0.0085	0.025	0.0086 0.50
995:	37	16	21	1	37	36	16	20	1	36	123963.7907	0.0300	0.025	0.0300 0.50
996:	37	16	22	1	37	36	16	21	1	36	123963.7907	0.0300	0.025	0.0300 0.50
997:	37	18	20	1	37	36	18	19	1	36	123965.5940	0.0123	0.025	0.0124 0.50
998:	37	18	19	1	37	36	18	18	1	36	123965.5940	0.0123	0.025	0.0124 0.50
999:	37	15	23	1	37	36	15	22	1	36	123972.4580	0.0095	0.025	0.0095 0.50
1000:	37	15	22	1	37	36	15	21	1	36	123972.4580	0.0095	0.025	0.0095 0.50
1001:	37	20	17	1	37	36	20	16	1	36	123986.5100	0.0132	0.025	0.0133 0.50
1002:	37	20	18	1	37	36	20	17	1	36	123986.5100	0.0132	0.025	0.0133 0.50
1003:	38	3	36	1	38	37	3	35	1	37	124013.2979	0.0131	0.025	
1004:	37	22	15	1	37	36	22	14	1	36	124021.6940	0.0216	0.025	0.0216 0.50
1005:	37	22	16	1	37	36	22	15	1	36	124021.6940	0.0216	0.025	0.0216 0.50
1006:	37	23	14	1	37	36	23	13	1	36	124043.6285	-0.0163	0.025	-0.0163 0.50
1007:	37	23	15	1	37	36	23	14	1	36	124043.6285	-0.0163	0.025	-0.0163 0.50
1008:	37	3	34	1	37	36	3	33	1	36	124060.9363	0.0162	0.025	
1009:	39	11	28	1	39	39	10	29	1	39	124069.1506	0.0452	0.025	-0.0370 0.50
1010:	39	11	29	1	39	39	10	30	1	39	124069.1506	-0.1192	0.025	-0.0370 0.50
1011:	37	25	12	1	37	36	25	11	1	36	124095.0643	-0.0010	0.025	-0.0010 0.50
1012:	37	25	13	1	37	36	25	12	1	36	124095.0643	-0.0010	0.025	-0.0010 0.50
1013:	38	2	36	1	38	37	2	35	1	37	124105.7003	-0.0041	0.025	
1014:	35	4	32	1	35	34	3	31	1	34	124143.9334	0.0566	0.025	
1015:	38	11	27	1	38	38	10	28	1	38	124175.3634	0.0455	0.025	-0.0038 0.50
1016:	38	11	28	1	38	38	10	29	1	38	124175.3634	-0.0531	0.025	-0.0038 0.50
1017:	37	10	27	1	37	36	10	26	1	36	124209.4320	0.0043	0.025	
1018:	37	11	26	1	37	37	10	27	1	37	124272.5033	0.0367	0.025	0.0076 0.50
1019:	37	11	27	1	37	37	10	28	1	37	124272.5033	-0.0215	0.025	0.0076 0.50
1020:	14	7	8	1	14	13	6	7	1	13	124301.0416	0.0273	0.025	0.0184 0.50
1021:	14	7	7	1	14	13	6	8	1	13	124301.0416	0.0094	0.025	0.0184 0.50
1022:	37	9	28	1	37	36	9	27	1	36	124339.2443	-0.0106	0.025	
1023:	36	11	25	1	36	36	10	26	1	36	124361.1139	0.0126	0.025	-0.0043 0.50
1024:	36	11	26	1	36	36	10	27	1	36	124361.1139	-0.0212	0.025	-0.0043 0.50
1025:	38	3	36	1	38	37	2	35	1	37	124392.6413	0.0322	0.025	
1026:	35	11	24	1	35	35	10	25	1	35	124441.7942	0.0492	0.025	0.0395 0.50
1027:	35	11	25	1	35	35	10	26	1	35	124441.7942	0.0298	0.025	0.0395 0.50
1028:	34	11	23	1	34	34	10	24	1	34	124514.9144	0.0179	0.025	0.0125 0.50
1029:	34	11	24	1	34	34	10	25	1	34	124514.9144	0.0071	0.025	0.0125 0.50
1030:	37	8	29	1	37	36	8	28	1	36	124539.4285	0.0038	0.025	
1031:	37	5	33	1	37	36	5	32	1	36	124568.9931	0.0301	0.025	
1032:	33	11	22	1	33	33	10	23	1	33	124581.0528	0.0211	0.025	0.0181 0.50
1033:	33	11	23	1	33	33	10	24	1	33	124581.0528	0.0151	0.025	0.0181 0.50
1034:	32	11	21	1	32	32	10	22	1	32	124640.6207	0.0148	0.025	0.0132 0.50
1035:	32	11	22	1	32	32	10	23	1	32	124640.6207	0.0116	0.025	0.0132 0.50
1036:	31	11	20	1	31	31	10	21	1	31	124694.0664	0.0119	0.025	0.0111 0.50
1037:	31	11	21	1	31	31	10	22	1	31	124694.0664	0.0102	0.025	0.0111 0.50
1038:	39	1	38	1	39	38	2	37	1	38	124700.8580	-0.0028	0.025	
1039:	39	2	38	1	39	38	2	37	1	38	124710.4589	0.0020	0.025	
1040:	39	1	38	1	39	38	1	37	1	38	124714.1040	0.0112	0.025	
1041:	39	2	38	1	39	38	1	37	1	38	124723.6848	-0.0039	0.025	
1042:	30	11	19	1	30	30	10	20	1	30	124741.8062	0.0118	0.025	0.0114 0.50
1043:	30	11	20	1	30	30	10	21	1	30	124741.8062	0.0109	0.025	0.0114 0.50
1044:	28	11	17	1	28	28	10	18	1	28	124821.7506	0.0211	0.025	0.0211 0.50
1045:	28	11	18	1	28	28	10	19	1	28	124821.7506	0.0209	0.025	0.0211 0.50
1046:	26	11	15	1	26	26	10	16	1	26	124883.4277	0.0172	0.025	0.0172 0.50
1047:	26	11	16	1	26	26	10	17	1	26	124883.4277	0.0171	0.025	0.0172 0.50
1048:	25	11	14	1	25	25	10	15	1	25	124908.3001	0.0246	0.025	0.0247 0.50
1049:	25	11	15	1	25	25	10	16	1	25	124908.3001	0.0246	0.025	0.0247 0.50
1050:	37	7	30	1	37	36	7	29	1	36	124909.2900	0.0054	0.025	
1051:	24	11	13	1	24	24	10	14	1	24	124929.6501	0.0589	0.025	0.0589 0.50
1052:	24	11	14	1	24	24	10	15	1	24	124929.6501	0.0589	0.025	0.0589 0.50
1053:	23	11	12	1	23	23	10	13	1	23	124947.6857	0.0194	0.025	0.0194 0.50
1054:	23	11	13	1	23	23	10	14	1	23	124947.6857	0.0193	0.025	0.0194 0.50
1055:	22	11	11	1	22	22	10	12	1	22	124962.8060	0.0103	0.025	0.0103 0.50
1056:	22	11	12	1	22	22	10	13	1	22	124962.8060	0.0103	0.025	0.0103 0.50
1057:	21	11	10	1	21	21	10	11	1	21	124975.2726	0.0116	0.025	0.0116 0.50
1058:	21	11	11	1	21	21	10	12	1	21	124975.2726	0.0116	0.025	0.0116 0.50
1059:	40	1	40	1	40	39	1	39	1	39	125422.0170	0.0163	0.025	-0.0107 0.50
1060:	40	0	40	1	40	39	0	39	1	39	125422.0170	-0.0377	0.025	-0.0107 0.50
1061:	11	8	4	1	11	10	7	3	1	10	126178.1074	-0.0060	0.025	-0.0060 0.50

1062:	11	8	3	1	11	10	7	4	1	10	126178.1074	-0.0060	0.025	-0.0060	0.50
1063:	38	4	35	1	38	37	4	34	1	37	126268.1504	0.0042	0.025		
1064:	39	2	37	1	39	38	3	36	1	38	126900.6939	-0.0234	0.025		
1065:	39	3	37	1	39	38	3	36	1	38	127116.8830	-0.0355	0.025		
1066:	39	2	37	1	39	38	2	36	1	38	127187.6307	0.0088	0.025		
1067:	38	16	23	1	38	37	16	22	1	37	127316.6369	-0.0370	0.025	-0.0370	0.50
1068:	38	16	22	1	38	37	16	21	1	37	127316.6369	-0.0370	0.025	-0.0370	0.50
1069:	38	19	20	1	38	37	19	19	1	37	127323.5508	-0.0283	0.025	-0.0283	0.50
1070:	38	19	19	1	38	37	19	18	1	37	127323.5508	-0.0283	0.025	-0.0283	0.50
1071:	38	20	19	1	38	37	20	18	1	37	127335.4885	-0.0565	0.025	-0.0565	0.50
1072:	38	20	18	1	38	37	20	17	1	37	127335.4885	-0.0565	0.025	-0.0565	0.50
1073:	39	3	37	1	39	38	2	36	1	38	127403.8179	-0.0052	0.025		
1074:	38	24	14	1	38	37	24	13	1	37	127417.0041	-0.0308	0.025	-0.0309	0.50
1075:	38	24	15	1	38	37	24	14	1	37	127417.0041	-0.0308	0.025	-0.0309	0.50
1076:	38	10	29	1	38	37	10	28	1	37	127589.4954	-0.0155	0.025	-0.0365	0.50
1077:	38	10	28	1	38	37	10	27	1	37	127589.4954	-0.0574	0.025	-0.0365	0.50
1078:	40	2	39	1	40	39	2	38	1	39	127809.8416	0.0108	0.025		
1079:	40	1	39	1	40	39	1	38	1	39	127812.4405	-0.0394	0.025		
1080:	40	2	39	1	40	39	1	38	1	39	127819.4179	-0.0088	0.025		
1081:	38	6	33	1	38	37	6	32	1	37	128371.0663	-0.0540	0.025		
1082:	19	6	14	1	19	18	5	13	1	18	128888.5445	-0.0296	0.025		
1083:	61	5	57	1	61	60	5	56	1	60	200018.0521	0.0608	0.030	0.0609	0.50
1084:	61	5	57	1	61	60	5	56	1	60	200018.0521	0.0608	0.030	0.0609	0.50
1085:	61	4	57	1	61	60	4	56	1	60	200048.8388	-0.0032	0.030		
1086:	26	10	17	1	26	25	9	16	1	25	200092.0158	0.0044	0.030	0.0038	0.50
1087:	26	10	16	1	26	25	9	17	1	25	200092.0158	0.0031	0.030	0.0038	0.50
1088:	61	5	57	1	61	60	4	56	1	60	200147.0388	0.0887	0.030		
1089:	59	9	50	1	59	58	9	49	1	58	200355.1138	0.0278	0.030		
1090:	59	6	53	1	59	58	6	52	1	58	200483.0577	-0.0404	0.030		
1091:	19	12	7	1	19	18	11	8	1	18	200492.7090	-0.0098	0.030	-0.0099	0.50
1092:	19	12	8	1	19	18	11	7	1	18	200492.7090	-0.0098	0.030	-0.0099	0.50
1093:	62	3	59	1	62	61	4	58	1	61	200646.3775	-0.0117	0.030		
1094:	62	4	59	1	62	61	4	58	1	61	200650.8222	-0.0256	0.030		
1095:	62	3	59	1	62	61	3	58	1	61	200652.3679	-0.0462	0.030		
1096:	62	4	59	1	62	61	3	58	1	61	200656.8821	0.0093	0.030		
1097:	60	21	39	1	60	59	21	38	1	59	200951.3690	0.0021	0.030	0.0021	0.50
1098:	60	21	40	1	60	59	21	39	1	59	200951.3690	0.0021	0.030	0.0021	0.50
1099:	60	22	38	1	60	59	22	37	1	59	200953.3222	0.0140	0.030	0.0140	0.50
1100:	60	22	39	1	60	59	22	38	1	59	200953.3222	0.0140	0.030	0.0140	0.50
1101:	60	20	40	1	60	59	20	39	1	59	200959.1850	0.0110	0.030	0.0111	0.50
1102:	60	20	41	1	60	59	20	40	1	59	200959.1850	0.0110	0.030	0.0111	0.50
1103:	60	23	37	1	60	59	23	36	1	59	200963.6824	0.0225	0.030	0.0225	0.50
1104:	60	23	38	1	60	59	23	37	1	59	200963.6824	0.0225	0.030	0.0225	0.50
1105:	60	19	41	1	60	59	19	40	1	59	200978.4525	0.0103	0.030	0.0103	0.50
1106:	60	19	42	1	60	59	19	41	1	59	200978.4525	0.0103	0.030	0.0103	0.50
1107:	60	24	36	1	60	59	24	35	1	59	200981.3802	0.0181	0.030	0.0182	0.50
1108:	60	24	37	1	60	59	24	36	1	59	200981.3802	0.0181	0.030	0.0182	0.50
1109:	60	25	35	1	60	59	25	34	1	59	201005.5727	0.0088	0.030	0.0089	0.50
1110:	60	25	36	1	60	59	25	35	1	59	201005.5727	0.0088	0.030	0.0089	0.50
1111:	60	18	42	1	60	59	18	41	1	59	201011.4167	0.0161	0.030	0.0162	0.50
1112:	60	18	43	1	60	59	18	42	1	59	201011.4167	0.0161	0.030	0.0162	0.50
1113:	39	7	33	1	39	38	6	32	1	38	201021.5846	-0.0248	0.030		
1114:	60	26	34	1	60	59	26	33	1	59	201035.5986	0.0241	0.030	0.0242	0.50
1115:	60	26	35	1	60	59	26	34	1	59	201035.5986	0.0241	0.030	0.0242	0.50
1116:	60	17	43	1	60	59	17	42	1	59	201061.0212	0.0175	0.030	0.0175	0.50
1117:	60	17	44	1	60	59	17	43	1	59	201061.0212	0.0175	0.030	0.0175	0.50
1118:	60	27	33	1	60	59	27	32	1	59	201070.8466	0.0199	0.030	0.0199	0.50
1119:	60	27	34	1	60	59	27	33	1	59	201070.8466	0.0199	0.030	0.0199	0.50
1120:	60	28	32	1	60	59	28	31	1	59	201110.8701	0.0198	0.030	0.0198	0.50
1121:	60	28	33	1	60	59	28	32	1	59	201110.8701	0.0198	0.030	0.0198	0.50
1122:	60	16	44	1	60	59	16	43	1	59	201131.2533	-0.0013	0.030	-0.0013	0.50
1123:	60	16	45	1	60	59	16	44	1	59	201131.2533	-0.0013	0.030	-0.0013	0.50
1124:	60	6	55	1	60	59	5	54	1	59	201154.5380	0.0791	0.030		
1125:	60	29	32	1	60	59	29	31	1	59	201155.2931	0.0416	0.030	0.0416	0.50
1126:	60	29	31	1	60	59	29	30	1	59	201155.2931	0.0416	0.030	0.0416	0.50
1127:	60	30	30	1	60	59	30	29	1	59	201203.7028	0.0052	0.030	0.0053	0.50
1128:	60	30	31	1	60	59	30	30	1	59	201203.7028	0.0052	0.030	0.0053	0.50
1129:	60	15	46	1	60	59	15	45	1	59	201227.7155	-0.0060	0.030	-0.0062	0.50
1130:	60	15	45	1	60	59	15	44	1	59	201227.7155	-0.0063	0.030	-0.0062	0.50
1131:	60	31	29	1	60	59	31	28	1	59	201255.8532	-0.0519	0.030	-0.0519	0.50
1132:	60	31	30	1	60	59	31	29	1	59	201255.8532	-0.0519	0.030	-0.0519	0.50
1133:	60	32	28	1	60	59	32	27	1	59	201311.6463	0.0155	0.030	0.0156	0.50
1134:	60	32	29	1	60	59	32	28	1	59	201311.6463	0.0155	0.030	0.0156	0.50
1135:	63	2	61	1	63	62	3	60	1	62	201336.9830	0.1344	0.030	-0.0191	0.30
1136:	63	3	61	1	63	62	3	60	1	62	201336.9830	0.0055	0.030	-0.0191	0.20
1137:	63	2	61	1	63	62	2	60	1	62	201336.9830	-0.0437	0.030	-0.0191	0.20
1138:	63	3	61	1	63	62	2	60	1	62	201336.9830	-0.1726	0.030	-0.0191	0.30
1139:	60	14	47	1	60	59	14	46	1	59	201358.4262	0.0195	0.030	0.0169	0.50
1140:	60	14	46	1	60	59	14	45	1	59	201358.4262	0.0143	0.030	0.0169	0.50
1141:	60	33	27	1	60	59	33	26	1	59	201370.6870	0.0235	0.030	0.0235	0.50
1142:	60	33	28	1	60	59	33	27	1	59	201370.6870	0.0235	0.030	0.0235	0.50
1143:	61	5	56	1	61	60	6	55	1	60	201399.0945	-0.0336	0.030		
1144:	60	34	26	1	60	59	34	25	1	59	201432.8384	0.0189	0.030	0.0190	0.50

1145:	60	34	27	1	60	59	34	26	1	59	201432.8384	0.0189	0.030	0.0190	0.50
1146:	60	13	48	1	60	59	13	47	1	59	201535.3169	0.0380	0.030	-0.0095	0.50
1147:	60	13	47	1	60	59	13	46	1	59	201535.3169	-0.0571	0.030	-0.0095	0.50
1148:	60	7	54	1	60	59	7	53	1	59	201549.0060	-0.0150	0.030		
1149:	60	36	24	1	60	59	36	23	1	59	201565.8761	0.0032	0.030	0.0032	0.50
1150:	60	36	25	1	60	59	36	24	1	59	201565.8761	0.0032	0.030	0.0032	0.50
1151:	60	37	23	1	60	59	37	22	1	59	201636.5147	0.0150	0.030	0.0150	0.50
1152:	60	37	24	1	60	59	37	23	1	59	201636.5147	0.0150	0.030	0.0150	0.50
1153:	60	12	49	1	60	59	12	48	1	59	201776.8493	-0.0262	0.030		
1154:	60	12	48	1	60	59	12	47	1	59	201778.2890	-0.0173	0.030		
1155:	60	39	21	1	60	59	39	20	1	59	201785.4173	0.0372	0.030	0.0373	0.50
1156:	60	39	22	1	60	59	39	21	1	59	201785.4173	0.0372	0.030	0.0373	0.50
1157:	60	41	19	1	60	59	41	18	1	59	201943.7757	-0.0119	0.030	-0.0120	0.50
1158:	60	41	20	1	60	59	41	19	1	59	201943.7757	-0.0119	0.030	-0.0120	0.50
1159:	23	11	12	1	23	22	10	13	1	22	202003.7154	0.0163	0.030	0.0163	0.50
1160:	23	11	13	1	23	22	10	12	1	22	202003.7154	0.0163	0.030	0.0163	0.50
1161:	34	8	26	1	34	33	7	27	1	33	202041.3478	0.0164	0.030		
1162:	64	1	63	1	64	63	2	62	1	63	202051.4420	-0.0013	0.030	-0.0039	0.30
1163:	64	2	63	1	64	63	2	62	1	63	202051.4420	-0.0034	0.030	-0.0039	0.20
1164:	64	1	63	1	64	63	1	62	1	63	202051.4420	-0.0043	0.030	-0.0039	0.20
1165:	64	2	63	1	64	63	1	62	1	63	202051.4420	-0.0065	0.030	-0.0039	0.30
1166:	60	11	49	1	60	59	11	48	1	59	202127.1518	0.0115	0.030		
1167:	16	13	3	1	16	15	12	4	1	15	202288.8464	0.0383	0.030	0.0383	0.50
1168:	16	13	4	1	16	15	12	3	1	15	202288.8464	0.0383	0.030	0.0383	0.50
1169:	61	6	56	1	61	60	6	55	1	60	202490.7330	0.0470	0.030		
1170:	60	10	50	1	60	59	10	49	1	59	202705.8247	0.0202	0.030		
1171:	65	0	65	1	65	64	1	64	1	64	202776.9353	-0.0153	0.030	-0.0153	0.30
1172:	65	1	65	1	65	64	1	64	1	64	202776.9353	-0.0153	0.030	-0.0153	0.20
1173:	65	0	65	1	65	64	0	64	1	64	202776.9353	-0.0153	0.030	-0.0153	0.20
1174:	65	1	65	1	65	64	0	64	1	64	202776.9353	-0.0153	0.030	-0.0153	0.30
1175:	61	5	56	1	61	60	5	55	1	60	202786.2680	0.0020	0.030		
1176:	60	8	53	1	60	59	8	52	1	59	202787.4615	0.0340	0.030		
1177:	60	9	52	1	60	59	9	51	1	59	202941.8694	0.0362	0.030		
1178:	39	6	33	1	39	38	5	34	1	38	202969.9449	-0.0679	0.030		
1179:	62	4	58	1	62	61	5	57	1	61	203023.3522	0.0297	0.030		
1180:	62	5	58	1	62	61	5	57	1	61	203097.8120	0.0108	0.030		
1181:	62	4	58	1	62	61	4	57	1	61	203121.4563	0.0257	0.030		
1182:	62	5	58	1	62	61	4	57	1	61	203195.9097	0.0004	0.030		
1183:	59	7	52	1	59	58	7	51	1	58	203297.6214	0.0048	0.030		
1184:	60	6	54	1	60	59	6	53	1	59	203411.6092	-0.0133	0.030		
1185:	27	10	18	1	27	26	9	17	1	26	203416.1533	0.0211	0.030	0.0197	0.50
1186:	27	10	17	1	27	26	9	18	1	26	203416.1533	0.0183	0.030	0.0197	0.50
1187:	63	3	60	1	63	62	4	59	1	62	203730.6967	-0.0017	0.030		
1188:	63	4	60	1	63	62	4	59	1	62	203734.0279	0.0341	0.030		
1189:	63	3	60	1	63	62	3	59	1	62	203735.1104	-0.0465	0.030		
1190:	63	4	60	1	63	62	3	59	1	62	203738.4775	0.0252	0.030		
1191:	88	18	70	1	88	88	17	71	1	88	203785.7032	0.0881	0.030	-0.0088	0.50
1192:	88	18	71	1	88	88	17	72	1	88	203785.7032	-0.1056	0.030	-0.0088	0.50
1193:	20	12	8	1	20	19	11	9	1	19	203842.7666	-0.0232	0.030	-0.0233	0.50
1194:	20	12	9	1	20	19	11	8	1	19	203842.7666	-0.0232	0.030	-0.0233	0.50
1195:	61	6	56	1	61	60	5	55	1	60	203877.8515	0.0277	0.030		
1196:	60	9	51	1	60	59	9	50	1	59	203968.8659	-0.0259	0.030		
1197:	87	18	69	1	87	87	17	70	1	87	204019.8440	0.1110	0.030	0.0433	0.50
1198:	87	18	70	1	87	87	17	71	1	87	204019.8440	-0.0243	0.030	0.0433	0.50
1199:	86	18	68	1	86	86	17	69	1	86	204243.9067	0.0919	0.030	0.0449	0.50
1200:	86	18	69	1	86	86	17	70	1	86	204243.9067	-0.0021	0.030	0.0449	0.50
1201:	61	21	40	1	61	60	21	39	1	60	204292.6935	-0.0181	0.030	-0.0181	0.50
1202:	61	21	41	1	61	60	21	40	1	60	204292.6935	-0.0181	0.030	-0.0181	0.50
1203:	61	22	39	1	61	60	22	38	1	60	204293.1629	0.0526	0.030	0.0527	0.50
1204:	61	22	40	1	61	60	22	39	1	60	204293.1629	0.0526	0.030	0.0527	0.50
1205:	61	23	38	1	61	60	23	37	1	60	204302.3735	0.1023	0.030	-0.0247	0.20
1206:	61	23	39	1	61	60	23	38	1	60	204302.3735	0.1023	0.030	-0.0247	0.20
1207:	61	20	41	1	61	60	20	40	1	60	204302.3735	-0.1093	0.030	-0.0247	0.30
1208:	61	20	42	1	61	60	20	41	1	60	204302.3735	-0.1093	0.030	-0.0247	0.30
1209:	61	24	37	1	61	60	24	36	1	60	204319.0945	0.0141	0.030	0.0142	0.50
1210:	61	24	38	1	61	60	24	37	1	60	204319.0945	0.0141	0.030	0.0142	0.50
1211:	61	19	42	1	61	60	19	41	1	60	204324.2134	-0.0139	0.030	-0.0140	0.50
1212:	61	19	43	1	61	60	19	42	1	60	204324.2134	-0.0139	0.030	-0.0140	0.50
1213:	61	25	36	1	61	60	25	35	1	60	204342.6618	0.0179	0.030	0.0180	0.50
1214:	61	25	37	1	61	60	25	36	1	60	204342.6618	0.0179	0.030	0.0180	0.50
1215:	61	18	43	1	61	60	18	42	1	60	204360.2692	-0.0252	0.030	-0.0252	0.50
1216:	61	18	44	1	61	60	18	43	1	60	204360.2692	-0.0252	0.030	-0.0252	0.50
1217:	61	26	35	1	61	60	26	34	1	60	204372.2290	-0.0073	0.030	-0.0074	0.50
1218:	61	26	36	1	61	60	26	35	1	60	204372.2290	-0.0073	0.030	-0.0074	0.50
1219:	61	27	34	1	61	60	27	33	1	60	204407.2720	0.0092	0.030	0.0093	0.50
1220:	61	27	35	1	61	60	27	34	1	60	204407.2720	0.0092	0.030	0.0093	0.50
1221:	61	17	44	1	61	60	17	43	1	60	204413.8161	0.0138	0.030	0.0138	0.50
1222:	61	17	45	1	61	60	17	44	1	60	204413.8161	0.0138	0.030	0.0138	0.50
1223:	64	2	62	1	64	63	3	61	1	63	204422.4349	0.1080	0.030	-0.0030	0.30
1224:	64	3	62	1	64	63	3	61	1	63	204422.4349	0.0148	0.030	-0.0030	0.20
1225:	64	2	62	1	64	63	2	61	1	63	204422.4349	-0.0208	0.030	-0.0030	0.20
1226:	64	3	62	1	64	63	2	61	1	63	204422.4349	-0.1141	0.030	-0.0030	0.30
1227:	61	28	33	1	61	60	28	32	1	60	204447.2363	0.0066	0.030	0.0066	0.50

1228:	61 28 34	1 61	60 28 33	1 60	204447.2363	0.0066	0.030	0.0066	0.50
1229:	85 18 67	1 85	85 17 68	1 85	204458.2311	0.0367	0.030	0.0042	0.50
1230:	85 18 68	1 85	85 17 69	1 85	204458.2311	-0.0283	0.030	0.0042	0.50
1231:	61 16 45	1 61	60 16 44	1 60	204488.9965	0.0131	0.030	0.0132	0.50
1232:	61 16 46	1 61	60 16 45	1 60	204488.9965	0.0131	0.030	0.0132	0.50
1233:	61 29 32	1 61	60 29 31	1 60	204491.6896	-0.0348	0.030	-0.0348	0.50
1234:	61 29 33	1 61	60 29 32	1 60	204491.6896	-0.0348	0.030	-0.0348	0.50
1235:	61 30 31	1 61	60 30 30	1 60	204540.4287	0.0302	0.030	0.0303	0.50
1236:	61 30 32	1 61	60 30 31	1 60	204540.4287	0.0302	0.030	0.0303	0.50
1237:	61 15 47	1 61	60 15 46	1 60	204591.7503	0.0113	0.030	0.0112	0.50
1238:	61 15 46	1 61	60 15 45	1 60	204591.7503	0.0109	0.030	0.0112	0.50
1239:	61 31 31	1 61	60 31 30	1 60	204592.9292	-0.0256	0.030	-0.0257	0.50
1240:	61 31 30	1 61	60 31 29	1 60	204592.9292	-0.0256	0.030	-0.0257	0.50
1241:	61 32 29	1 61	60 32 28	1 60	204649.0832	-0.0557	0.030	-0.0558	0.50
1242:	61 32 30	1 61	60 32 29	1 60	204649.0832	-0.0557	0.030	-0.0558	0.50
1243:	84 18 66	1 84	84 17 67	1 84	204663.2085	0.0156	0.030	-0.0067	0.50
1244:	84 18 67	1 84	84 17 68	1 84	204663.2085	-0.0290	0.030	-0.0067	0.50
1245:	61 33 28	1 61	60 33 27	1 60	204708.6676	-0.0626	0.030	-0.0626	0.50
1246:	61 33 29	1 61	60 33 28	1 60	204708.6676	-0.0626	0.030	-0.0626	0.50
1247:	61 7 55	1 61	60 7 54	1 60	204711.4034	-0.0140	0.030		
1248:	62 5 57	1 62	61 6 56	1 61	204722.2631	0.0001	0.030		
1249:	61 14 48	1 61	60 14 47	1 60	204730.5800	0.0022	0.030	-0.0018	0.50
1250:	61 14 47	1 61	60 14 46	1 60	204730.5800	-0.0058	0.030	-0.0018	0.50
1251:	61 34 27	1 61	60 34 26	1 60	204771.5359	-0.0003	0.030	-0.0004	0.50
1252:	61 34 28	1 61	60 34 27	1 60	204771.5359	-0.0003	0.030	-0.0004	0.50
1253:	83 18 65	1 83	83 17 66	1 83	204859.1594	0.0402	0.030	0.0250	0.50
1254:	83 18 66	1 83	83 17 67	1 83	204859.1594	0.0096	0.030	0.0250	0.50
1255:	61 36 25	1 61	60 36 24	1 60	204906.1405	0.0036	0.030	0.0036	0.50
1256:	61 36 26	1 61	60 36 25	1 60	204906.1405	0.0036	0.030	0.0036	0.50
1257:	61 13 49	1 61	60 13 48	1 60	204918.3462	0.0703	0.030	-0.0005	0.50
1258:	61 13 48	1 61	60 13 47	1 60	204918.3462	-0.0712	0.030	-0.0005	0.50
1259:	61 37 24	1 61	60 37 23	1 60	204977.6464	-0.0027	0.030	-0.0027	0.50
1260:	61 37 25	1 61	60 37 24	1 60	204977.6464	-0.0027	0.030	-0.0027	0.50
1261:	82 18 64	1 82	82 17 65	1 82	205046.2833	0.0121	0.030	0.0017	0.50
1262:	82 18 65	1 82	82 17 66	1 82	205046.2833	-0.0086	0.030	0.0017	0.50
1263:	61 38 23	1 61	60 38 22	1 60	205051.7765	-0.0292	0.030	-0.0293	0.50
1264:	61 38 24	1 61	60 38 23	1 60	205051.7765	-0.0292	0.030	-0.0293	0.50
1265:	61 39 22	1 61	60 39 21	1 60	205128.5495	0.0501	0.030	0.0501	0.50
1266:	61 39 23	1 61	60 39 22	1 60	205128.5495	0.0501	0.030	0.0501	0.50
1267:	65 1 64	1 65	64 2 63	1 64	205137.8451	-0.0143	0.030	-0.0162	0.30
1268:	65 2 64	1 65	64 2 63	1 64	205137.8451	-0.0158	0.030	-0.0162	0.20
1269:	65 1 64	1 65	64 1 63	1 64	205137.8451	-0.0165	0.030	-0.0162	0.20
1270:	65 2 64	1 65	64 1 63	1 64	205137.8451	-0.0180	0.030	-0.0162	0.30
1271:	61 12 50	1 61	60 12 49	1 60	205174.6274	0.0238	0.030		
1272:	61 12 49	1 61	60 12 48	1 60	205176.6613	0.0100	0.030		
1273:	81 18 63	1 81	81 17 64	1 81	205224.9657	0.0292	0.030	0.0222	0.50
1274:	81 18 64	1 81	81 17 65	1 81	205224.9657	0.0151	0.030	0.0222	0.50
1275:	24 11 14	1 24	23 10 13	1 23	205344.9349	-0.0049	0.030	-0.0050	0.50
1276:	24 11 13	1 24	23 10 14	1 23	205344.9349	-0.0049	0.030	-0.0050	0.50
1277:	61 11 51	1 61	60 11 50	1 60	205526.9420	0.0214	0.030		
1278:	61 44 17	1 61	60 44 16	1 60	205546.8386	-0.0515	0.030	-0.0516	0.50
1279:	61 44 18	1 61	60 44 17	1 60	205546.8386	-0.0515	0.030	-0.0516	0.50
1280:	61 11 50	1 61	60 11 49	1 60	205550.6769	0.0210	0.030		
1281:	79 18 61	1 79	79 17 62	1 79	205557.8514	-0.0570	0.030	-0.0602	0.50
1282:	79 18 62	1 79	79 17 63	1 79	205557.8514	-0.0632	0.030	-0.0602	0.50
1283:	62 6 57	1 62	61 6 56	1 61	205578.1172	0.0358	0.030		
1284:	17 13 4	1 17	16 12 5	1 16	205641.8924	0.0352	0.030	0.0353	0.50
1285:	17 13 5	1 17	16 12 4	1 16	205641.8924	0.0352	0.030	0.0353	0.50
1286:	62 5 57	1 62	61 5 56	1 61	205813.8130	-0.0078	0.030		
1287:	77 18 59	1 77	77 17 60	1 77	205860.1711	0.0211	0.030	0.0198	0.50
1288:	77 18 60	1 77	77 17 61	1 77	205860.1711	0.0184	0.030	0.0198	0.50
1289:	66 0 66	1 66	65 0 65	1 65	205863.7707	-0.0147	0.030	-0.0148	0.20
1290:	66 0 66	1 66	65 1 65	1 65	205863.7707	-0.0147	0.030	-0.0148	0.30
1291:	66 1 66	1 66	65 0 65	1 65	205863.7707	-0.0148	0.030	-0.0148	0.30
1292:	66 1 66	1 66	65 1 65	1 65	205863.7707	-0.0147	0.030	-0.0148	0.20
1293:	61 10 52	1 61	60 10 51	1 60	205978.5649	0.0345	0.030		
1294:	76 18 58	1 76	76 17 59	1 76	206000.3835	0.0117	0.030	0.0108	0.50
1295:	76 18 59	1 76	76 17 60	1 76	206000.3835	0.0099	0.030	0.0108	0.50
1296:	61 8 54	1 61	60 8 53	1 60	206085.4816	-0.0283	0.030		
1297:	63 4 59	1 63	62 5 58	1 62	206120.4136	-0.0056	0.030		
1298:	75 18 57	1 75	75 17 58	1 75	206133.6391	-0.0067	0.030	-0.0074	0.50
1299:	75 18 58	1 75	75 17 59	1 75	206133.6391	-0.0079	0.030	-0.0074	0.50
1300:	63 5 59	1 63	62 5 58	1 62	206176.8908	0.0443	0.030		
1301:	61 10 51	1 61	60 10 50	1 60	206188.5086	0.0033	0.030		
1302:	63 4 59	1 63	62 4 58	1 62	206194.8559	-0.0420	0.030		
1303:	60 8 52	1 60	59 8 51	1 59	206222.8614	-0.0001	0.030		
1304:	63 5 59	1 63	62 4 58	1 62	206251.2664	-0.0587	0.030		
1305:	74 18 56	1 74	74 17 57	1 74	206260.1891	-0.0132	0.030	-0.0136	0.50
1306:	74 18 57	1 74	74 17 58	1 74	206260.1891	-0.0139	0.030	-0.0136	0.50
1307:	61 6 55	1 61	60 6 54	1 60	206332.5223	-0.0036	0.030		
1308:	61 9 53	1 61	60 9 52	1 60	206348.8286	0.0068	0.030		
1309:	66 7 59	1 66	65 8 58	1 65	206362.6359	0.0063	0.030		
1310:	39 7 32	1 39	38 6 33	1 38	206463.6015	-0.0050	0.030		

1311:	72	18	54	1	72	72	17	55	1	72	206494.0407	-0.0093	0.030	-0.0095 0.50
1312:	72	18	55	1	72	72	17	56	1	72	206494.0407	-0.0096	0.030	-0.0095 0.50
1313:	60	7	53	1	60	59	7	52	1	59	206563.5252	0.0045	0.030	
1314:	71	18	53	1	71	71	17	54	1	71	206601.7260	-0.0439	0.030	-0.0440 0.50
1315:	71	18	54	1	71	71	17	55	1	71	206601.7260	-0.0441	0.030	-0.0440 0.50
1316:	62	6	57	1	62	61	5	56	1	61	206669.6171	-0.0220	0.030	
1317:	70	18	52	1	70	70	17	53	1	70	206703.6143	-0.0158	0.030	-0.0159 0.50
1318:	70	18	53	1	70	70	17	54	1	70	206703.6143	-0.0160	0.030	-0.0159 0.50
1319:	28	10	19	1	28	27	9	18	1	27	206736.0664	0.0155	0.030	0.0129 0.50
1320:	28	10	18	1	28	27	9	19	1	27	206736.0664	0.0101	0.030	0.0129 0.50
1321:	69	18	51	1	69	69	17	52	1	69	206799.7980	-0.0331	0.030	-0.0332 0.50
1322:	69	18	52	1	69	69	17	53	1	69	206799.7980	-0.0332	0.030	-0.0332 0.50
1323:	64	3	61	1	64	63	4	60	1	63	206814.1766	-0.0036	0.030	
1324:	64	4	61	1	64	63	4	60	1	63	206816.5969	-0.0160	0.030	
1325:	64	3	61	1	64	63	3	60	1	63	206817.4833	0.0077	0.030	
1326:	64	4	61	1	64	63	3	60	1	63	206819.8699	-0.0383	0.030	
1327:	68	18	50	1	68	68	17	51	1	68	206890.5841	0.0159	0.030	0.0160 0.50
1328:	68	18	51	1	68	68	17	52	1	68	206890.5841	0.0159	0.030	0.0160 0.50
1329:	67	18	49	1	67	67	17	50	1	67	206976.0177	-0.0137	0.030	-0.0138 0.50
1330:	67	18	50	1	67	67	17	51	1	67	206976.0177	-0.0138	0.030	-0.0138 0.50
1331:	66	18	48	1	66	66	17	49	1	66	207056.3955	-0.0114	0.030	-0.0115 0.50
1332:	66	18	49	1	66	66	17	50	1	66	207056.3955	-0.0114	0.030	-0.0115 0.50
1333:	65	18	47	1	65	65	17	48	1	65	207131.8588	-0.0169	0.030	-0.0170 0.50
1334:	65	18	48	1	65	65	17	49	1	65	207131.8588	-0.0169	0.030	-0.0170 0.50
1335:	21	12	9	1	21	20	11	10	1	20	207192.0934	-0.0051	0.030	-0.0051 0.50
1336:	21	12	10	1	21	20	11	9	1	20	207192.0934	-0.0051	0.030	-0.0051 0.50
1337:	64	18	46	1	64	64	17	47	1	64	207202.5990	-0.0157	0.030	-0.0158 0.50
1338:	64	18	47	1	64	64	17	48	1	64	207202.5990	-0.0157	0.030	-0.0158 0.50
1339:	63	18	45	1	63	63	17	46	1	63	207268.7929	-0.0038	0.030	-0.0038 0.50
1340:	63	18	46	1	63	63	17	47	1	63	207268.7929	-0.0038	0.030	-0.0038 0.50
1341:	62	18	44	1	62	62	17	45	1	62	207330.5726	-0.0177	0.030	-0.0177 0.50
1342:	62	18	45	1	62	62	17	46	1	62	207330.5726	-0.0177	0.030	-0.0177 0.50
1343:	61	18	43	1	61	61	17	44	1	61	207388.1369	-0.0235	0.030	-0.0236 0.50
1344:	61	18	44	1	61	61	17	45	1	61	207388.1369	-0.0235	0.030	-0.0236 0.50
1345:	43	7	37	1	43	42	6	36	1	42	207412.1117	0.0075	0.030	
1346:	14	14	0	1	14	13	13	1	1	13	207414.5121	0.0119	0.030	0.0120 0.50
1347:	14	14	1	1	14	13	13	0	1	13	207414.5121	0.0119	0.030	0.0120 0.50
1348:	60	18	42	1	60	60	17	43	1	60	207441.6429	-0.0254	0.030	-0.0254 0.50
1349:	60	18	43	1	60	60	17	44	1	60	207441.6429	-0.0254	0.030	-0.0254 0.50
1350:	59	18	41	1	59	59	17	42	1	59	207491.2538	-0.0176	0.030	-0.0176 0.50
1351:	59	18	42	1	59	59	17	43	1	59	207491.2538	-0.0176	0.030	-0.0176 0.50
1352:	65	2	63	1	65	64	3	62	1	64	207507.2661	0.0883	0.030	0.0080 0.30
1353:	65	3	63	1	65	64	3	62	1	64	207507.2661	0.0209	0.030	0.0080 0.20
1354:	65	2	63	1	65	64	2	62	1	64	207507.2661	-0.0048	0.030	0.0080 0.20
1355:	65	3	63	1	65	64	2	62	1	64	207507.2661	-0.0722	0.030	0.0080 0.30
1356:	58	18	40	1	58	58	17	41	1	58	207537.0905	-0.0334	0.030	-0.0334 0.50
1357:	58	18	41	1	58	58	17	42	1	58	207537.0905	-0.0334	0.030	-0.0334 0.50
1358:	57	18	39	1	57	57	17	40	1	57	207579.3697	-0.0067	0.030	-0.0068 0.50
1359:	57	18	40	1	57	57	17	41	1	57	207579.3697	-0.0067	0.030	-0.0068 0.50
1360:	61	9	52	1	61	60	9	51	1	60	207608.1679	-0.0426	0.030	
1361:	56	18	38	1	56	56	17	39	1	56	207618.1592	-0.0173	0.030	-0.0173 0.50
1362:	56	18	39	1	56	56	17	40	1	56	207618.1592	-0.0173	0.030	-0.0173 0.50
1363:	62	22	40	1	62	61	22	39	1	61	207632.3918	-0.0095	0.030	-0.0095 0.50
1364:	62	22	41	1	62	61	22	40	1	61	207632.3918	-0.0095	0.030	-0.0095 0.50
1365:	62	21	42	1	62	61	21	41	1	61	207633.6358	0.0147	0.030	0.0147 0.50
1366:	62	21	41	1	62	61	21	40	1	61	207633.6358	0.0147	0.030	0.0147 0.50
1367:	62	23	39	1	62	61	23	38	1	61	207640.2942	-0.0125	0.030	-0.0125 0.50
1368:	62	23	40	1	62	61	23	39	1	61	207640.2942	-0.0125	0.030	-0.0125 0.50
1369:	62	20	42	1	62	61	20	41	1	61	207645.4623	0.0167	0.030	0.0168 0.50
1370:	62	20	43	1	62	61	20	42	1	61	207645.4623	0.0167	0.030	0.0168 0.50
1371:	55	18	37	1	55	55	17	38	1	55	207653.6433	-0.0250	0.030	-0.0251 0.50
1372:	55	18	38	1	55	55	17	39	1	55	207653.6433	-0.0250	0.030	-0.0251 0.50
1373:	62	24	38	1	62	61	24	37	1	61	207656.1536	-0.0130	0.030	-0.0130 0.50
1374:	62	24	39	1	62	61	24	38	1	61	207656.1536	-0.0130	0.030	-0.0130 0.50
1375:	62	19	43	1	62	61	19	42	1	61	207669.7726	0.0002	0.030	0.0003 0.50
1376:	62	19	44	1	62	61	19	43	1	61	207669.7726	0.0002	0.030	0.0003 0.50
1377:	62	25	37	1	62	61	25	36	1	61	207679.0321	-0.0107	0.030	-0.0108 0.50
1378:	62	25	38	1	62	61	25	37	1	61	207679.0321	-0.0107	0.030	-0.0108 0.50
1379:	54	18	36	1	54	54	17	37	1	54	207686.0052	0.0120	0.030	0.0121 0.50
1380:	54	18	37	1	54	54	17	38	1	54	207686.0052	0.0120	0.030	0.0121 0.50
1381:	62	26	37	1	62	61	26	36	1	61	207708.1979	0.0235	0.030	0.0236 0.50
1382:	62	26	36	1	62	61	26	35	1	61	207708.1979	0.0235	0.030	0.0236 0.50
1383:	62	18	45	1	62	61	18	44	1	61	207709.0434	-0.0326	0.030	-0.0327 0.50
1384:	62	18	44	1	62	61	18	43	1	61	207709.0434	-0.0326	0.030	-0.0327 0.50
1385:	18	18	0	1	18	18	17	1	1	18	207709.8636	0.0398	0.030	0.0399 0.50
1386:	18	18	1	1	18	18	17	2	1	18	207709.8636	0.0398	0.030	0.0399 0.50
1387:	53	18	36	1	53	53	17	37	1	53	207715.2757	-0.0132	0.030	-0.0133 0.50
1388:	53	18	35	1	53	53	17	36	1	53	207715.2757	-0.0132	0.030	-0.0133 0.50
1389:	19	18	1	1	19	19	17	2	1	19	207719.8062	-0.0113	0.030	-0.0114 0.50
1390:	19	18	2	1	19	19	17	3	1	19	207719.8062	-0.0113	0.030	-0.0114 0.50
1391:	20	18	2	1	20	20	17	3	1	20	207730.0052	0.0007	0.030	0.0007 0.50
1392:	20	18	3	1	20	20	17	4	1	20	207730.0052	0.0007	0.030	0.0007 0.50
1393:	21	18	3	1	21	21	17	4	1	21	207740.3152	-0.0183	0.030	-0.0184 0.50

1394:	21	18	4	1	21	21	17	5	1	21	207740.3152	-0.0183	0.030	-0.0184	0.50
1395:	52	18	35	1	52	52	17	36	1	52	207741.6925	0.0013	0.030	0.0014	0.50
1396:	52	18	34	1	52	52	17	35	1	52	207741.6925	0.0013	0.030	0.0014	0.50
1397:	62	27	36	1	62	61	27	35	1	61	207742.9197	-0.0172	0.030	-0.0172	0.50
1398:	62	27	35	1	62	61	27	34	1	61	207742.9197	-0.0172	0.030	-0.0172	0.50
1399:	22	18	5	1	22	22	17	6	1	22	207750.7526	0.0010	0.030	0.0010	0.50
1400:	22	18	4	1	22	22	17	5	1	22	207750.7526	0.0010	0.030	0.0010	0.50
1401:	23	18	6	1	23	23	17	7	1	23	207761.2050	0.0022	0.030	0.0022	0.50
1402:	23	18	5	1	23	23	17	6	1	23	207761.2050	0.0022	0.030	0.0022	0.50
1403:	51	18	34	1	51	51	17	35	1	51	207765.3350	0.0031	0.030	0.0031	0.50
1404:	51	18	33	1	51	51	17	34	1	51	207765.3350	0.0031	0.030	0.0031	0.50
1405:	62	17	46	1	62	61	17	45	1	61	207766.6476	0.0013	0.030	0.0014	0.50
1406:	62	17	45	1	62	61	17	44	1	61	207766.6476	0.0013	0.030	0.0014	0.50
1407:	24	18	6	1	24	24	17	7	1	24	207771.6237	-0.0052	0.030	-0.0053	0.50
1408:	24	18	7	1	24	24	17	8	1	24	207771.6237	-0.0052	0.030	-0.0053	0.50
1409:	50	18	32	1	50	50	17	33	1	50	207786.3434	0.0027	0.030	0.0027	0.50
1410:	50	18	33	1	50	50	17	34	1	50	207786.3434	0.0027	0.030	0.0027	0.50
1411:	26	18	9	1	26	26	17	10	1	26	207792.1692	0.0077	0.030	0.0077	0.50
1412:	26	18	8	1	26	26	17	9	1	26	207792.1692	0.0077	0.030	0.0077	0.50
1413:	27	18	10	1	27	27	17	11	1	27	207802.1292	-0.0101	0.030	-0.0101	0.50
1414:	27	18	9	1	27	27	17	10	1	27	207802.1292	-0.0101	0.030	-0.0101	0.50
1415:	49	18	31	1	49	49	17	32	1	49	207804.8706	0.0262	0.030	0.0262	0.50
1416:	49	18	32	1	49	49	17	33	1	49	207804.8706	0.0262	0.030	0.0262	0.50
1417:	28	18	10	1	28	28	17	11	1	28	207811.8405	0.0052	0.030	0.0053	0.50
1418:	28	18	11	1	28	28	17	12	1	28	207811.8405	0.0052	0.030	0.0053	0.50
1419:	48	18	31	1	48	48	17	32	1	48	207821.0578	0.0908	0.030	0.0060	0.30
1420:	48	18	30	1	48	48	17	31	1	48	207821.0578	0.0908	0.030	0.0060	0.30
1421:	29	18	11	1	29	29	17	12	1	29	207821.0578	-0.1212	0.030	0.0060	0.20
1422:	29	18	12	1	29	29	17	13	1	29	207821.0578	-0.1212	0.030	0.0060	0.20
1423:	62	29	33	1	62	61	29	32	1	61	207827.3713	-0.0005	0.030	-0.0006	0.50
1424:	62	29	34	1	62	61	29	33	1	61	207827.3713	-0.0005	0.030	-0.0006	0.50
1425:	30	18	12	1	30	30	17	13	1	30	207830.1028	0.0048	0.030	0.0048	0.50
1426:	30	18	13	1	30	30	17	14	1	30	207830.1028	0.0048	0.030	0.0048	0.50
1427:	47	18	30	1	47	47	17	31	1	47	207834.8121	-0.0176	0.030	-0.0176	0.50
1428:	47	18	29	1	47	47	17	30	1	47	207834.8121	-0.0176	0.030	-0.0176	0.50
1429:	31	18	14	1	31	31	17	15	1	31	207838.5049	-0.0123	0.030	-0.0123	0.50
1430:	31	18	13	1	31	31	17	14	1	31	207838.5049	-0.0123	0.030	-0.0123	0.50
1431:	32	18	14	1	32	32	17	15	1	32	207846.4438	0.0845	0.030	0.0845	0.50
1432:	32	18	15	1	32	32	17	16	1	32	207846.4438	0.0845	0.030	0.0845	0.50
1433:	45	18	27	1	45	45	17	28	1	45	207856.2409	-0.0075	0.030	-0.0075	0.50
1434:	45	18	28	1	45	45	17	29	1	45	207856.2409	-0.0075	0.030	-0.0075	0.50
1435:	62	7	56	1	62	61	7	55	1	61	207859.9540	0.0296	0.030	-0.0100	0.40
1436:	34	18	16	1	34	34	17	17	1	34	207859.9540	-0.0365	0.030	-0.0100	0.30
1437:	34	18	17	1	34	34	17	18	1	34	207859.9540	-0.0365	0.030	-0.0100	0.30
1438:	44	18	26	1	44	44	17	27	1	44	207864.0346	0.0005	0.030	0.0005	0.50
1439:	44	18	27	1	44	44	17	28	1	44	207864.0346	0.0005	0.030	0.0005	0.50
1440:	35	18	18	1	35	35	17	19	1	35	207865.6002	-0.0128	0.030	-0.0129	0.50
1441:	35	18	17	1	35	35	17	18	1	35	207865.6002	-0.0128	0.030	-0.0129	0.50
1442:	39	18	22	1	39	39	17	23	1	39	207878.1680	0.0735	0.030	-0.0188	0.20
1443:	39	18	21	1	39	39	17	22	1	39	207878.1680	0.0735	0.030	-0.0188	0.20
1444:	40	18	22	1	40	40	17	23	1	40	207878.1680	-0.0804	0.030	-0.0188	0.30
1445:	40	18	23	1	40	40	17	24	1	40	207878.1680	-0.0804	0.030	-0.0188	0.30
1446:	62	31	31	1	62	61	31	30	1	61	207929.0819	-0.0463	0.030	-0.0464	0.50
1447:	62	31	32	1	62	61	31	31	1	61	207929.0819	-0.0463	0.030	-0.0464	0.50
1448:	62	15	48	1	62	61	15	47	1	61	207956.2290	-0.0281	0.030	-0.0285	0.50
1449:	62	15	47	1	62	61	15	46	1	61	207956.2290	-0.0287	0.030	-0.0285	0.50
1450:	62	32	30	1	62	61	32	29	1	61	207985.7938	0.0445	0.030	0.0446	0.50
1451:	62	32	31	1	62	61	32	30	1	61	207985.7938	0.0445	0.030	0.0446	0.50
1452:	63	5	58	1	63	62	6	57	1	62	207992.9902	0.0512	0.030		
1453:	62	33	29	1	62	61	33	28	1	61	208045.7937	-0.0856	0.030	-0.0857	0.50
1454:	62	33	30	1	62	61	33	29	1	61	208045.7937	-0.0856	0.030	-0.0857	0.50
1455:	62	14	49	1	62	61	14	48	1	61	208103.5788	-0.0178	0.030	-0.0240	0.50
1456:	62	14	48	1	62	61	14	47	1	61	208103.5788	-0.0301	0.030	-0.0240	0.50
1457:	62	35	27	1	62	61	35	26	1	61	208175.8362	-0.0520	0.030	-0.0520	0.50
1458:	62	35	28	1	62	61	35	27	1	61	208175.8362	-0.0520	0.030	-0.0520	0.50
1459:	66	1	65	1	66	65	2	64	1	65	208223.6034	-0.0211	0.030	-0.0224	0.30
1460:	66	2	65	1	66	65	2	64	1	65	208223.6034	-0.0222	0.030	-0.0224	0.20
1461:	66	1	65	1	66	65	1	64	1	65	208223.6034	-0.0226	0.030	-0.0224	0.20
1462:	66	2	65	1	66	65	1	64	1	65	208223.6034	-0.0237	0.030	-0.0224	0.30
1463:	62	36	26	1	62	61	36	25	1	61	208245.4600	0.0250	0.030	0.0251	0.50
1464:	62	36	27	1	62	61	36	26	1	61	208245.4600	0.0250	0.030	0.0251	0.50
1465:	62	13	50	1	62	61	13	49	1	61	208302.7196	0.1111	0.030	0.0067	0.50
1466:	62	13	49	1	62	61	13	48	1	61	208302.7196	-0.0976	0.030	0.0067	0.50
1467:	62	38	24	1	62	61	38	23	1	61	208392.9348	0.0180	0.030	0.0180	0.50
1468:	62	38	25	1	62	61	38	24	1	61	208392.9348	0.0180	0.030	0.0180	0.50
1469:	36	8	28	1	36	35	7	29	1	35	208409.4095	0.0115	0.030	0.0115	0.50
1470:	36	8	28	1	36	35	7	29	1	35	208409.4095	0.0115	0.030	0.0115	0.50
1471:	62	12	51	1	62	61	12	50	1	61	208574.3325	-0.0015	0.030		
1472:	62	12	50	1	62	61	12	49	1	61	208577.2181	-0.0243	0.030		
1473:	40	6	34	1	40	39	5	35	1	39	208620.5155	0.0258	0.030		
1474:	62	41	21	1	62	61	41	20	1	61	208633.4255	0.0024	0.030	0.0024	0.50
1475:	62	41	22	1	62	61	41	21	1	61	208633.4255	0.0024	0.030	0.0024	0.50
1476:	63	6	58	1	63	62	6	57	1	62	208661.6777	0.0116	0.030		

1477:	25 11 14 1 25	24 10 15 1 24	208684.0721	-0.0181	0.030	-0.0181	0.50
1478:	25 11 15 1 25	24 10 14 1 24	208684.0721	-0.0181	0.030	-0.0181	0.50
1479:	63 5 58 1 63	62 5 57 1 62	208848.7930	0.0356	0.030		
1480:	62 11 52 1 62	61 11 51 1 61	208946.4344	0.0041	0.030		
1481:	67 0 67 1 67	66 0 66 1 66	208949.9370	-0.0281	0.030	-0.0282	0.20
1482:	67 0 67 1 67	66 1 66 1 66	208949.9370	-0.0281	0.030	-0.0282	0.30
1483:	67 1 67 1 67	66 1 66 1 66	208949.9370	-0.0281	0.030	-0.0282	0.20
1484:	67 1 67 1 67	66 0 66 1 66	208949.9370	-0.0281	0.030	-0.0282	0.30
1485:	62 11 51 1 62	61 11 50 1 61	208978.8022	0.0026	0.030		
1486:	18 13 5 1 18	17 12 6 1 17	208994.7524	-0.0085	0.030	-0.0085	0.50
1487:	18 13 6 1 18	17 12 5 1 17	208994.7524	-0.0085	0.030	-0.0085	0.50
1488:	64 4 60 1 64	63 5 59 1 63	209212.5993	0.0506	0.030		
1489:	62 6 56 1 62	61 6 55 1 61	209253.2021	-0.0197	0.030		
1490:	64 5 60 1 64	63 5 59 1 63	209255.2372	0.0180	0.030		
1491:	64 4 60 1 64	63 4 59 1 63	209269.0464	0.0705	0.030		
1492:	64 5 60 1 64	63 4 59 1 63	209311.6940	0.0476	0.030		
1493:	62 8 55 1 62	61 8 54 1 61	209365.5630	-0.0264	0.030		
1494:	62 10 53 1 62	61 10 52 1 61	209411.5778	0.0503	0.030		
1495:	63 6 58 1 63	62 5 57 1 62	209517.4810	-0.0034	0.030		
1496:	46 7 40 1 46	45 6 39 1 45	209650.3887	0.0322	0.030		
1497:	62 10 52 1 62	61 10 51 1 61	209684.6568	0.0823	0.030		
1498:	62 9 54 1 62	61 9 53 1 61	209746.6838	0.0292	0.030		
1499:	61 7 54 1 61	60 7 53 1 60	209767.7890	0.0125	0.030		
1500:	64 6 58 1 64	63 7 57 1 63	209789.5477	-0.0170	0.030		
1501:	55 7 49 1 55	54 6 48 1 54	209792.2567	0.0202	0.030		
1502:	61 8 53 1 61	60 8 52 1 60	209870.6100	-0.0141	0.030		
1503:	65 3 62 1 65	64 4 61 1 64	209896.9143	0.0162	0.030		
1504:	65 4 62 1 65	64 4 61 1 64	209898.7365	0.0445	0.030		
1505:	65 3 62 1 65	64 3 61 1 64	209899.3726	0.0418	0.030		
1506:	65 4 62 1 65	64 3 61 1 64	209901.1674	0.0427	0.030		
1507:	56 7 50 1 56	55 6 49 1 55	210019.3359	-0.0350	0.030		
1508:	29 10 20 1 29	28 9 19 1 28	210051.3524	0.0073	0.030	0.0021	0.50
1509:	29 10 19 1 29	28 9 20 1 28	210051.3524	-0.0032	0.030	0.0021	0.50
1510:	40 7 33 1 40	39 6 34 1 39	210063.7604	0.0355	0.030		
1511:	50 7 44 1 50	49 6 43 1 49	210092.1460	0.0492	0.030		
1512:	48 7 42 1 48	47 6 41 1 47	210130.7667	0.0274	0.030		
1513:	57 7 51 1 57	56 6 50 1 56	210425.4254	0.0219	0.030		
1514:	22 12 10 1 22	21 11 11 1 21	210540.4353	-0.0515	0.030	-0.0515	0.50
1515:	22 12 11 1 22	21 11 10 1 21	210540.4353	-0.0515	0.030	-0.0515	0.50
1516:	66 2 64 1 66	65 3 63 1 65	210591.4356	0.0444	0.030	-0.0136	0.30
1517:	66 3 64 1 66	65 3 63 1 65	210591.4356	-0.0042	0.030	-0.0136	0.20
1518:	66 2 64 1 66	65 2 63 1 65	210591.4356	-0.0229	0.030	-0.0136	0.20
1519:	66 3 64 1 66	65 2 63 1 65	210591.4356	-0.0715	0.030	-0.0136	0.30
1520:	63 22 41 1 63	62 22 40 1 62	210971.1862	0.0161	0.030	0.0161	0.50
1521:	63 22 42 1 63	62 22 41 1 62	210971.1862	0.0161	0.030	0.0161	0.50
1522:	63 21 43 1 63	62 21 42 1 62	210974.0932	0.0082	0.030	0.0083	0.50
1523:	63 21 42 1 63	62 21 41 1 62	210974.0932	0.0082	0.030	0.0083	0.50
1524:	63 23 40 1 63	62 23 39 1 62	210977.7593	0.0053	0.030	0.0053	0.50
1525:	63 23 41 1 63	62 23 40 1 62	210977.7593	0.0053	0.030	0.0053	0.50
1526:	63 20 43 1 63	62 20 42 1 62	210988.0098	-0.0432	0.030	-0.0433	0.50
1527:	63 20 44 1 63	62 20 43 1 62	210988.0098	-0.0432	0.030	-0.0433	0.50
1528:	63 24 39 1 63	62 24 38 1 62	210992.6017	-0.0060	0.030	-0.0061	0.50
1529:	63 24 40 1 63	62 24 39 1 62	210992.6017	-0.0060	0.030	-0.0061	0.50
1530:	63 7 57 1 63	62 7 56 1 62	210995.6173	-0.0789	0.030		
1531:	63 26 37 1 63	62 26 36 1 62	211043.3668	-0.0071	0.030	-0.0072	0.50
1532:	63 26 38 1 63	62 26 37 1 62	211043.3668	-0.0071	0.030	-0.0072	0.50
1533:	63 18 45 1 63	62 18 44 1 62	211057.7307	-0.0104	0.030	-0.0105	0.50
1534:	63 18 46 1 63	62 18 45 1 62	211057.7307	-0.0104	0.030	-0.0105	0.50
1535:	63 27 36 1 63	62 27 35 1 62	211077.8268	-0.0075	0.030	-0.0075	0.50
1536:	63 27 37 1 63	62 27 36 1 62	211077.8268	-0.0075	0.030	-0.0075	0.50
1537:	63 17 46 1 63	62 17 45 1 62	211119.5122	-0.0225	0.030	-0.0226	0.50
1538:	63 17 47 1 63	62 17 46 1 62	211119.5122	-0.0225	0.030	-0.0226	0.50
1539:	33 9 25 1 33	32 8 24 1 32	211124.3872	-0.0190	0.030		
1540:	63 28 35 1 63	62 28 34 1 62	211117.6098	0.0231	0.030	0.0232	0.50
1541:	63 28 36 1 63	62 28 35 1 62	211117.6098	0.0231	0.030	0.0232	0.50
1542:	63 29 34 1 63	62 29 33 1 62	211162.1600	-0.0182	0.030	-0.0183	0.50
1543:	63 29 35 1 63	62 29 34 1 62	211162.1600	-0.0182	0.030	-0.0183	0.50
1544:	63 16 48 1 63	62 16 47 1 62	211205.1650	-0.0112	0.030	-0.0113	0.50
1545:	63 16 47 1 63	62 16 46 1 62	211205.1650	-0.0113	0.030	-0.0113	0.50
1546:	63 30 33 1 63	62 30 32 1 62	211211.2602	0.0328	0.030	0.0328	0.50
1547:	63 30 34 1 63	62 30 33 1 62	211211.2602	0.0328	0.030	0.0328	0.50
1548:	64 5 59 1 64	63 6 58 1 63	211221.2023	0.0151	0.030		
1549:	63 31 32 1 63	62 31 31 1 62	211264.4279	0.0187	0.030	0.0187	0.50
1550:	63 31 33 1 63	62 31 32 1 62	211264.4279	0.0187	0.030	0.0187	0.50
1551:	62 9 53 1 62	61 9 52 1 61	211272.0714	-0.0407	0.030		
1552:	37 8 30 1 37	36 7 29 1 36	211278.4042	-0.0420	0.030		
1553:	67 1 66 1 67	66 2 65 1 66	211308.7207	-0.0075	0.030	-0.0084	0.30
1554:	67 2 66 1 67	66 2 65 1 66	211308.7207	-0.0082	0.030	-0.0084	0.20
1555:	67 1 66 1 67	66 1 65 1 66	211308.7207	-0.0086	0.030	-0.0084	0.20
1556:	67 2 66 1 67	66 1 65 1 66	211308.7207	-0.0093	0.030	-0.0084	0.30
1557:	63 15 49 1 63	62 15 48 1 62	211321.3079	0.0191	0.030	0.0187	0.50
1558:	63 15 48 1 63	62 15 47 1 62	211321.3079	0.0181	0.030	0.0187	0.50
1559:	63 33 30 1 63	62 33 29 1 62	211382.1544	0.0600	0.030	0.0601	0.50

1560:	63	33	31	1	63	62	33	30	1	62	211382.1544	0.0600	0.030	0.0601	0.50
1561:	63	14	50	1	63	62	14	49	1	62	211477.5148	0.0261	0.030	0.0168	0.50
1562:	63	14	49	1	63	62	14	48	1	62	211477.5148	0.0073	0.030	0.0168	0.50
1563:	63	35	28	1	63	62	35	27	1	62	211513.4029	-0.0170	0.030	-0.0170	0.50
1564:	63	35	29	1	63	62	35	28	1	62	211513.4029	-0.0170	0.030	-0.0170	0.50
1565:	37	8	29	1	37	36	7	30	1	36	211577.6920	-0.0247	0.030		
1566:	63	36	28	1	63	62	36	27	1	62	211583.7654	0.0155	0.030		
1567:	63	37	26	1	63	62	37	25	1	62	211656.9601	-0.0325	0.030	-0.0325	0.50
1568:	63	37	27	1	63	62	37	26	1	62	211656.9601	-0.0325	0.030	-0.0325	0.50
1569:	63	13	51	1	63	62	13	50	1	62	211688.4204	0.0983	0.030	-0.0545	0.50
1570:	63	13	50	1	63	62	13	49	1	62	211688.4204	-0.2074	0.030	-0.0545	0.50
1571:	63	38	26	1	63	62	38	25	1	62	211733.0729	0.0539	0.030	0.0539	0.50
1572:	63	38	25	1	63	62	38	24	1	62	211733.0729	0.0539	0.030	0.0539	0.50
1573:	64	6	59	1	64	63	6	58	1	63	211742.0741	-0.0181	0.030		
1574:	64	5	59	1	64	63	5	58	1	63	211889.8902	-0.0240	0.030		
1575:	63	40	24	1	63	62	40	23	1	62	211893.0093	0.0411	0.030	0.0412	0.50
1576:	63	40	23	1	63	62	40	22	1	62	211893.0093	0.0411	0.030	0.0412	0.50
1577:	63	12	52	1	63	62	12	51	1	62	211976.1150	-0.0086	0.030		
1578:	63	41	23	1	63	62	41	22	1	62	211976.7229	0.0332	0.030	0.0332	0.50
1579:	63	41	22	1	63	62	41	21	1	62	211976.7229	0.0332	0.030	0.0332	0.50
1580:	63	12	51	1	63	62	12	50	1	62	211980.1762	-0.0481	0.030		
1581:	26	11	15	1	26	25	10	16	1	25	212020.8740	-0.0159	0.030	-0.0159	0.50
1582:	26	11	16	1	26	25	10	15	1	25	212020.8740	-0.0159	0.030	-0.0159	0.50
1583:	68	0	68	1	68	67	0	67	1	67	212035.4563	-0.0235	0.030	-0.0235	0.20
1584:	68	0	68	1	68	67	1	67	1	67	212035.4563	-0.0235	0.030	-0.0235	0.30
1585:	68	1	68	1	68	67	0	67	1	67	212035.4563	-0.0235	0.030	-0.0235	0.30
1586:	68	1	68	1	68	67	1	67	1	67	212035.4563	-0.0235	0.030	-0.0235	0.20
1587:	63	6	57	1	63	62	6	56	1	62	212179.4721	0.0227	0.030		
1588:	65	4	61	1	65	64	5	60	1	64	212300.7726	-0.0013	0.030		
1589:	65	5	61	1	65	64	5	60	1	64	212332.9917	0.0073	0.030		
1590:	65	4	61	1	65	64	4	60	1	64	212343.4782	0.0337	0.030	0.0338	0.50
1591:	65	4	61	1	65	64	4	60	1	64	212343.4782	0.0337	0.030	0.0338	0.50
1592:	19	13	7	1	19	18	12	6	1	18	212347.3989	-0.0185	0.030	-0.0185	0.50
1593:	19	13	6	1	19	18	12	7	1	18	212347.3989	-0.0185	0.030	-0.0185	0.50
1594:	63	11	53	1	63	62	11	52	1	62	212368.2769	-0.0312	0.030		
1595:	65	5	61	1	65	64	4	60	1	64	212375.6190	-0.0357	0.030		
1596:	64	6	59	1	64	63	5	58	1	63	212410.8321	0.0128	0.030		
1597:	63	11	52	1	63	62	11	51	1	62	212412.1359	0.0085	0.030		
1598:	63	8	56	1	63	62	8	55	1	62	212627.3643	-0.0053	0.030		
1599:	63	10	54	1	63	62	10	53	1	62	212843.7862	-0.0690	0.030		
1600:	62	7	55	1	62	61	7	54	1	61	212911.7262	-0.0649	0.030		
1601:	66	4	63	1	66	65	4	62	1	65	212980.1417	-0.0742	0.030		
1602:	66	3	63	1	66	65	3	62	1	65	212980.7745	0.0861	0.030		
1603:	63	9	55	1	63	62	9	54	1	62	213133.7670	-0.0591	0.030		
1604:	63	10	53	1	63	62	10	52	1	62	213195.9028	-0.0477	0.030		
1605:	30	10	21	1	30	29	9	20	1	29	213361.5989	0.0288	0.030	0.0188	0.50
1606:	30	10	20	1	30	29	9	21	1	29	213361.5989	0.0088	0.030	0.0188	0.50
1607:	62	8	54	1	62	61	8	53	1	61	213480.1403	0.0154	0.030		
1608:	67	2	65	1	67	66	3	64	1	66	213674.9874	0.0310	0.030	-0.0109	0.30
1609:	67	3	65	1	67	66	3	64	1	66	213674.9874	-0.0041	0.030	-0.0109	0.20
1610:	67	2	65	1	67	66	2	64	1	66	213674.9874	-0.0176	0.030	-0.0109	0.20
1611:	67	3	65	1	67	66	2	64	1	66	213674.9874	-0.0527	0.030	-0.0109	0.30
1612:	65	6	59	1	65	64	7	58	1	64	213732.4099	0.0006	0.030		
1613:	41	7	34	1	41	40	6	35	1	40	213808.4398	0.0145	0.030		
1614:	23	12	11	1	23	22	11	12	1	22	213887.7412	-0.0460	0.030	-0.0461	0.50
1615:	23	12	12	1	23	22	11	11	1	22	213887.7412	-0.0460	0.030	-0.0461	0.50
1616:	64	7	58	1	64	63	7	57	1	63	214119.9617	0.0295	0.030		
1617:	16	14	2	1	16	15	13	3	1	15	214123.0551	-0.0253	0.030	-0.0253	0.50
1618:	16	14	3	1	16	15	13	2	1	15	214123.0551	-0.0253	0.030	-0.0253	0.50
1619:	64	7	58	1	64	63	7	57	1	63	214119.9617	0.0295	0.030		
1620:	16	14	2	1	16	15	13	3	1	15	214123.0551	-0.0253	0.030	-0.0253	0.50
1621:	16	14	3	1	16	15	13	2	1	15	214123.0551	-0.0253	0.030	-0.0253	0.50
1622:	61	7	55	1	61	60	6	54	1	60	214235.1534	-0.0412	0.030		
1623:	38	8	31	1	38	37	7	30	1	37	214307.8877	-0.0024	0.030		
1624:	64	22	42	1	64	63	22	41	1	63	214309.4004	-0.0043	0.030	-0.0044	0.50
1625:	64	22	43	1	64	63	22	42	1	63	214309.4004	-0.0043	0.030	-0.0044	0.50
1626:	64	21	44	1	64	63	21	43	1	63	214314.0601	-0.0326	0.030	-0.0326	0.50
1627:	64	21	43	1	64	63	21	42	1	63	214314.0601	-0.0326	0.030	-0.0326	0.50
1628:	64	23	41	1	64	63	23	40	1	63	214314.6491	0.0486	0.030	0.0487	0.50
1629:	64	23	42	1	64	63	23	41	1	63	214314.6491	0.0486	0.030	0.0487	0.50
1630:	64	24	40	1	64	63	24	39	1	63	214328.3985	0.0081	0.030	0.0081	0.50
1631:	64	24	41	1	64	63	24	40	1	63	214328.3985	0.0081	0.030	0.0081	0.50
1632:	64	20	44	1	64	63	20	43	1	63	214330.3065	0.0100	0.030	0.0100	0.50
1633:	64	20	45	1	64	63	20	44	1	63	214330.3065	0.0100	0.030	0.0100	0.50
1634:	64	25	39	1	64	63	25	38	1	63	214349.7330	-0.0095	0.030	-0.0095	0.50
1635:	64	25	40	1	64	63	25	39	1	63	214349.7330	-0.0095	0.030	-0.0095	0.50
1636:	64	19	45	1	64	63	19	44	1	63	214360.1303	0.0167	0.030	0.0168	0.50
1637:	64	19	46	1	64	63	19	45	1	63	214360.1303	0.0167	0.030	0.0168	0.50
1638:	34	9	26	1	34	33	8	25	1	33	214373.1202	0.0522	0.030		
1639:	68	1	67	1	68	67	2	66	1	67	214393.1527	-0.0075	0.030	-0.0083	0.30
1640:	68	2	67	1	68	67	2	66	1	67	214393.1527	-0.0081	0.030	-0.0083	0.20
1641:	68	1	67	1	68	67	1	66	1	67	214393.1527	-0.0083	0.030	-0.0083	0.20
1642:	68	2	67	1	68	67	1	66	1	67	214393.1527	-0.0089	0.030	-0.0083	0.30

1643:	64 18 46	1 64	63 18 45	1 63	214406.2927	0.0073	0.030	0.0073	0.50
1644:	64 18 47	1 64	63 18 46	1 63	214406.2927	0.0073	0.030	0.0073	0.50
1645:	64 27 37	1 64	63 27 36	1 63	214411.9487	0.0086	0.030	0.0086	0.50
1646:	64 27 38	1 64	63 27 37	1 63	214411.9487	0.0086	0.030	0.0086	0.50
1647:	65 5 60	1 65	64 6 59	1 64	214415.3300	-0.0018	0.030		
1648:	64 27 37	1 64	63 27 36	1 63	214411.9487	0.0086	0.030	0.0086	0.50
1649:	64 27 38	1 64	63 27 37	1 63	214411.9487	0.0086	0.030	0.0086	0.50
1650:	65 5 60	1 65	64 6 59	1 64	214415.3300	-0.0018	0.030		
1651:	64 28 36	1 64	63 28 35	1 63	214451.5292	-0.0044	0.030	-0.0045	0.50
1652:	64 28 37	1 64	63 28 36	1 63	214451.5292	-0.0044	0.030	-0.0045	0.50
1653:	64 17 47	1 64	63 17 46	1 63	214472.4659	-0.0013	0.030	-0.0014	0.50
1654:	64 17 48	1 64	63 17 47	1 63	214472.4659	-0.0013	0.030	-0.0014	0.50
1655:	64 29 35	1 64	63 29 34	1 63	214496.0882	-0.0397	0.030	-0.0397	0.50
1656:	64 29 36	1 64	63 29 35	1 63	214496.0882	-0.0397	0.030	-0.0397	0.50
1657:	64 30 34	1 64	63 30 33	1 63	214545.3607	0.0370	0.030	0.0370	0.50
1658:	64 30 35	1 64	63 30 34	1 63	214545.3607	0.0370	0.030	0.0370	0.50
1659:	64 16 49	1 64	63 16 48	1 63	214563.6493	-0.0004	0.030	-0.0005	0.50
1660:	64 16 48	1 64	63 16 47	1 63	214563.6493	-0.0005	0.030	-0.0005	0.50
1661:	64 31 33	1 64	63 31 32	1 63	214598.7702	-0.0111	0.030	-0.0112	0.50
1662:	64 31 34	1 64	63 31 33	1 63	214598.7702	-0.0111	0.030	-0.0112	0.50
1663:	64 32 32	1 64	63 32 31	1 63	214656.2653	0.0551	0.030	0.0552	0.50
1664:	64 32 33	1 64	63 32 32	1 63	214656.2653	0.0551	0.030	0.0552	0.50
1665:	64 15 50	1 64	63 15 49	1 63	214686.8637	0.0166	0.030	0.0159	0.50
1666:	64 15 49	1 64	63 15 48	1 63	214686.8637	0.0151	0.030	0.0159	0.50
1667:	41 6 35	1 41	40 5 36	1 40	214691.0016	-0.0665	0.030		
1668:	64 33 31	1 64	63 33 30	1 63	214717.2897	-0.0688	0.030	-0.0688	0.50
1669:	64 33 32	1 64	63 33 31	1 63	214717.2897	-0.0688	0.030	-0.0688	0.50
1670:	38 8 30	1 38	37 7 31	1 37	214738.5048	0.0022	0.030		
1671:	64 34 30	1 64	63 34 29	1 63	214782.0030	-0.0048	0.030	-0.0049	0.50
1672:	64 34 31	1 64	63 34 30	1 63	214782.0030	-0.0048	0.030	-0.0049	0.50
1673:	65 6 60	1 65	64 6 59	1 64	214819.8858	-0.0295	0.030		
1674:	64 35 29	1 64	63 35 28	1 63	214850.0162	0.0498	0.030	0.0499	0.50
1675:	64 35 30	1 64	63 35 29	1 63	214850.0162	0.0498	0.030	0.0499	0.50
1676:	64 14 51	1 64	63 14 50	1 63	214852.3115	0.0306	0.030	0.0165	0.50
1677:	64 14 50	1 64	63 14 49	1 63	214852.3115	0.0024	0.030	0.0165	0.50
1678:	64 36 28	1 64	63 36 27	1 63	214921.0742	0.0096	0.030	0.0096	0.50
1679:	64 36 29	1 64	63 36 28	1 63	214921.0742	0.0096	0.030	0.0096	0.50
1680:	65 5 60	1 65	64 5 59	1 64	214936.1927	-0.0442	0.030		
1681:	63 9 54	1 63	62 9 53	1 62	214957.9204	-0.0136	0.030		
1682:	96 19 77	1 96	96 18 78	1 96	214989.1067	0.0837	0.030	-0.0294	0.50
1683:	96 19 78	1 96	96 18 79	1 96	214989.1067	-0.1425	0.030	-0.0294	0.50
1684:	64 38 26	1 64	63 38 25	1 63	215072.1437	0.0487	0.030	0.0487	0.50
1685:	64 38 27	1 64	63 38 26	1 63	215072.1437	0.0487	0.030	0.0487	0.50
1686:	64 13 52	1 64	63 13 51	1 63	215075.4104	-0.0540	0.030		
1687:	64 13 51	1 64	63 13 50	1 63	215075.9241	0.0154	0.030		
1688:	64 6 58	1 64	63 6 57	1 63	215115.1282	-0.0545	0.030		
1689:	69 1 69	1 69	68 0 68	1 68	215120.2949	-0.0248	0.030	-0.0249	0.30
1690:	69 0 69	1 69	68 1 68	1 68	215120.2949	-0.0248	0.030	-0.0249	0.30
1691:	69 0 69	1 69	68 0 68	1 68	215120.2949	-0.0248	0.030	-0.0249	0.20
1692:	69 1 69	1 69	68 1 68	1 68	215120.2949	-0.0248	0.030	-0.0249	0.20
1693:	64 39 25	1 64	63 39 24	1 63	215151.7900	0.0184	0.030	0.0184	0.50
1694:	64 39 26	1 64	63 39 25	1 63	215151.7900	0.0184	0.030	0.0184	0.50
1695:	65 6 60	1 65	64 5 59	1 64	215340.7858	-0.0346	0.030		
1696:	27 11 17	1 27	26 10 16	1 26	215355.0312	-0.0341	0.030	-0.0341	0.50
1697:	27 11 16	1 27	26 10 17	1 26	215355.0312	-0.0341	0.030	-0.0341	0.50
1698:	64 12 53	1 64	63 12 52	1 63	215380.0260	0.0014	0.030		
1699:	64 12 52	1 64	63 12 51	1 63	215385.8391	0.0744	0.030	-0.0142	0.40
1700:	66 4 62	1 66	65 5 61	1 65	215385.8391	-0.0732	0.030	-0.0142	0.60
1701:	66 5 62	1 66	65 5 61	1 65	215410.2105	0.0244	0.030		
1702:	66 4 62	1 66	65 4 61	1 65	215418.1136	-0.0090	0.030		
1703:	66 5 62	1 66	65 4 61	1 65	215442.4058	0.0093	0.030		
1704:	20 13 7	1 20	19 12 8	1 19	215699.7164	-0.0009	0.030	-0.0010	0.50
1705:	20 13 8	1 20	19 12 7	1 19	215699.7164	-0.0009	0.030	-0.0010	0.50
1706:	62 7 56	1 62	61 6 55	1 61	215762.5168	-0.0761	0.030		
1707:	64 11 54	1 64	63 11 53	1 63	215792.4284	0.0112	0.030		
1708:	64 11 53	1 64	63 11 52	1 63	215851.3349	0.0304	0.030		
1709:	64 8 57	1 64	63 8 56	1 63	215870.7705	-0.0212	0.030		
1710:	63 7 56	1 63	62 7 55	1 62	215998.5889	-0.0195	0.030		
1711:	67 3 64	1 67	66 4 63	1 66	216060.1338	-0.0630	0.030		
1712:	67 4 64	1 67	66 4 63	1 66	216061.3391	0.1698	0.030	-0.0047	0.50
1713:	67 3 64	1 67	66 3 63	1 66	216061.3391	-0.1791	0.030	-0.0047	0.50
1714:	67 4 64	1 67	66 3 63	1 66	216062.5411	0.0503	0.030		
1715:	64 10 55	1 64	63 10 54	1 63	216274.5899	0.0001	0.030		
1716:	64 9 56	1 64	63 9 55	1 63	216508.8455	-0.0243	0.030		
1717:	89 19 70	1 89	89 18 71	1 89	216546.0823	0.0248	0.030	0.0156	0.50
1718:	89 19 71	1 89	89 18 72	1 89	216546.0823	0.0064	0.030	0.0156	0.50
1719:	31 10 22	1 31	30 9 21	1 30	216666.2819	0.0250	0.030	0.0066	0.50
1720:	31 10 21	1 31	30 9 22	1 30	216666.2819	-0.0119	0.030	0.0066	0.50
1721:	64 10 54	1 64	63 10 53	1 63	216724.7306	-0.0003	0.030		
1722:	88 19 69	1 88	88 18 70	1 88	216733.1101	0.0363	0.030	0.0300	0.50
1723:	88 19 70	1 88	88 18 71	1 88	216733.1101	0.0237	0.030	0.0300	0.50
1724:	68 2 66	1 68	67 3 65	1 67	216757.8874	0.0249	0.030	-0.0053	0.30
1725:	68 3 66	1 68	67 3 65	1 67	216757.8874	-0.0003	0.030	-0.0053	0.20

1726:	68	2	66	1	68	67	2	65	1	67	216757.8874	-0.0101	0.030	-0.0053 0.20
1727:	68	3	66	1	68	67	2	65	1	67	216757.8874	-0.0354	0.030	-0.0053 0.30
1728:	87	19	68	1	87	87	18	69	1	87	216912.0396	-0.0001	0.030	-0.0044 0.50
1729:	87	19	69	1	87	87	18	70	1	87	216912.0396	-0.0087	0.030	-0.0044 0.50
1730:	63	8	55	1	63	62	8	54	1	62	217043.5668	-0.0046	0.030	
1731:	86	19	67	1	86	86	18	68	1	86	217083.1995	-0.0020	0.030	-0.0050 0.50
1732:	86	19	68	1	86	86	18	69	1	86	217083.1995	-0.0079	0.030	-0.0050 0.50
1733:	24	12	12	1	24	23	11	13	1	23	217233.8294	0.0064	0.030	-0.0009 0.30
1734:	24	12	13	1	24	23	11	12	1	23	217233.8294	0.0064	0.030	-0.0009 0.30
1735:	65	7	59	1	65	64	7	58	1	64	217233.8294	-0.0117	0.030	-0.0009 0.40
1736:	85	19	66	1	85	85	18	67	1	85	217246.8321	0.0341	0.030	0.0322 0.50
1737:	85	19	67	1	85	85	18	68	1	85	217246.8321	0.0302	0.030	0.0322 0.50
1738:	68	7	61	1	68	67	8	60	1	67	217263.1844	0.0075	0.030	
1739:	39	8	32	1	39	38	7	31	1	38	217283.2328	-0.0539	0.030	
1740:	84	19	65	1	84	84	18	66	1	84	217403.0766	0.0161	0.030	0.0149 0.50
1741:	84	19	66	1	84	84	18	67	1	84	217403.0766	0.0135	0.030	0.0149 0.50
1742:	69	1	68	1	69	68	2	67	1	68	217476.9025	-0.0080	0.030	-0.0085 0.30
1743:	69	2	68	1	69	68	2	67	1	68	217476.9025	-0.0084	0.030	-0.0085 0.20
1744:	69	1	68	1	69	68	1	67	1	68	217476.9025	-0.0085	0.030	-0.0085 0.20
1745:	69	2	68	1	69	68	1	67	1	68	217476.9025	-0.0089	0.030	-0.0085 0.30
1746:	17	14	3	1	17	16	13	4	1	16	217477.5495	0.0278	0.030	0.0278 0.50
1747:	17	14	4	1	17	16	13	3	1	16	217477.5495	0.0278	0.030	0.0278 0.50
1748:	66	6	60	1	66	65	7	59	1	65	217521.8354	0.0148	0.030	
1749:	83	19	64	1	83	83	18	65	1	83	217552.2131	-0.0005	0.030	-0.0014 0.50
1750:	83	19	65	1	83	83	18	66	1	83	217552.2131	-0.0023	0.030	-0.0014 0.50
1751:	66	5	61	1	66	65	6	60	1	65	217582.2128	0.0013	0.030	
1752:	65	22	43	1	65	64	22	42	1	64	217647.1029	0.0091	0.030	0.0092 0.50
1753:	65	22	44	1	65	64	22	43	1	64	217647.1029	0.0091	0.030	0.0092 0.50
1754:	65	23	42	1	65	64	23	41	1	64	217650.8370	0.0035	0.030	0.0036 0.50
1755:	65	23	43	1	65	64	23	42	1	64	217650.8370	0.0035	0.030	0.0036 0.50
1756:	65	21	44	1	65	64	21	43	1	64	217653.6337	-0.0001	0.030	-0.0002 0.50
1757:	65	21	45	1	65	64	21	44	1	64	217653.6337	-0.0001	0.030	-0.0002 0.50
1758:	65	24	41	1	65	64	24	40	1	64	217663.4935	-0.0075	0.030	-0.0076 0.50
1759:	65	24	42	1	65	64	24	41	1	64	217663.4935	-0.0075	0.030	-0.0076 0.50
1760:	65	20	45	1	65	64	20	44	1	64	217672.1630	-0.0037	0.030	-0.0038 0.50
1761:	65	20	46	1	65	64	20	45	1	64	217672.1630	-0.0037	0.030	-0.0038 0.50
1762:	65	25	40	1	65	64	25	39	1	64	217684.0225	0.0073	0.030	0.0073 0.50
1763:	65	25	41	1	65	64	25	40	1	64	217684.0225	0.0073	0.030	0.0073 0.50
1764:	82	19	63	1	82	82	18	64	1	82	217694.4664	-0.0096	0.030	-0.0102 0.50
1765:	82	19	64	1	82	82	18	65	1	82	217694.4664	-0.0107	0.030	-0.0102 0.50
1766:	65	19	46	1	65	64	19	45	1	64	217704.9048	0.0090	0.030	0.0091 0.50
1767:	65	19	47	1	65	64	19	46	1	64	217704.9048	0.0090	0.030	0.0091 0.50
1768:	65	26	39	1	65	64	26	38	1	64	217711.4976	-0.0025	0.030	-0.0026 0.50
1769:	65	26	40	1	65	64	26	39	1	64	217711.4976	-0.0025	0.030	-0.0026 0.50
1770:	65	27	38	1	65	64	27	37	1	64	217745.2323	-0.0068	0.030	-0.0069 0.50
1771:	65	27	39	1	65	64	27	38	1	64	217745.2323	-0.0068	0.030	-0.0069 0.50
1772:	65	18	47	1	65	64	18	46	1	64	217754.7093	0.0050	0.030	0.0050 0.50
1773:	65	18	48	1	65	64	18	47	1	64	217754.7093	0.0050	0.030	0.0050 0.50
1774:	42	7	35	1	42	41	6	36	1	41	217731.3369	0.0703	0.030	
1775:	65	28	37	1	65	64	28	36	1	64	217784.6494	0.0102	0.030	0.0102 0.50
1776:	65	28	38	1	65	64	28	37	1	64	217784.6494	0.0102	0.030	0.0102 0.50
1777:	65	17	48	1	65	64	17	47	1	64	217825.4298	-0.0135	0.030	-0.0135 0.50
1778:	65	17	49	1	65	64	17	48	1	64	217825.4298	-0.0135	0.030	-0.0135 0.50
1779:	65	29	36	1	65	64	29	35	1	64	217829.1920	-0.0131	0.030	-0.0132 0.50
1780:	65	29	37	1	65	64	29	36	1	64	217829.1920	-0.0131	0.030	-0.0132 0.50
1781:	81	19	62	1	81	81	18	63	1	81	217830.0406	-0.0191	0.030	-0.0195 0.50
1782:	81	19	63	1	81	81	18	64	1	81	217830.0406	-0.0198	0.030	-0.0195 0.50
1783:	65	30	35	1	65	64	30	34	1	64	217878.5520	0.0322	0.030	0.0322 0.50
1784:	65	30	36	1	65	64	30	35	1	64	217878.5520	0.0322	0.030	0.0322 0.50
1785:	66	6	61	1	66	65	6	60	1	65	217895.5789	-0.0222	0.030	
1786:	65	16	50	1	65	64	16	49	1	64	217922.3884	0.0068	0.030	0.0068 0.50
1787:	65	16	49	1	65	64	16	48	1	64	217922.3884	0.0067	0.030	0.0068 0.50
1788:	65	31	34	1	65	64	31	33	1	64	217932.2057	-0.0229	0.030	-0.0229 0.50
1789:	65	31	35	1	65	64	31	34	1	64	217932.2057	-0.0229	0.030	-0.0229 0.50
1790:	80	19	61	1	80	80	18	62	1	80	217959.1714	0.0001	0.030	-0.0001 0.50
1791:	80	19	62	1	80	80	18	63	1	80	217959.1714	-0.0003	0.030	-0.0001 0.50
1792:	66	5	61	1	66	65	5	60	1	65	217986.7693	-0.0257	0.030	
1793:	65	32	33	1	65	64	32	32	1	64	217990.0386	0.0107	0.030	0.0107 0.50
1794:	65	32	34	1	65	64	32	33	1	64	217990.0386	0.0107	0.030	0.0107 0.50
1795:	65	15	51	1	65	64	15	50	1	64	218052.9490	0.0029	0.030	0.0017 0.50
1796:	65	15	50	1	65	64	15	49	1	64	218052.9490	0.0005	0.030	0.0017 0.50
1797:	65	6	59	1	65	64	6	58	1	64	218062.7607	-0.0159	0.030	
1798:	79	19	60	1	79	79	18	61	1	79	218082.0335	0.0220	0.030	0.0219 0.50
1799:	79	19	61	1	79	79	18	62	1	79	218082.0335	0.0217	0.030	0.0219 0.50
1800:	65	34	31	1	65	64	34	30	1	64	218116.8524	-0.0303	0.030	-0.0304 0.50
1801:	65	34	32	1	65	64	34	31	1	64	218116.8524	-0.0303	0.030	-0.0304 0.50
1802:	78	19	59	1	78	78	18	60	1	78	218198.7561	-0.0199	0.030	-0.0201 0.50
1803:	78	19	60	1	78	78	18	61	1	78	218198.7561	-0.0201	0.030	-0.0201 0.50
1804:	70	0	70	1	70	69	0	69	1	69	218204.4680	-0.0071	0.030	-0.0071 0.20
1805:	70	0	70	1	70	69	1	69	1	69	218204.4680	-0.0071	0.030	-0.0071 0.30
1806:	70	1	70	1	70	69	0	69	1	69	218204.4680	-0.0071	0.030	-0.0071 0.30
1807:	70	1	70	1	70	69	1	69	1	69	218204.4680	-0.0071	0.030	-0.0071 0.20
1808:	65	14	52	1	65	64	14	51	1	64	218228.0279	0.0259	0.030	0.0049 0.50

1809:	65	14	51	1	65	64	14	50	1	64	218228.0279	-0.0162	0.030	0.0049	0.50
1810:	77	19	58	1	77	77	18	59	1	77	218309.6515	-0.0042	0.030	-0.0043	0.50
1811:	77	19	59	1	77	77	18	60	1	77	218309.6515	-0.0044	0.030	-0.0043	0.50
1812:	65	37	28	1	65	64	37	27	1	64	218332.2479	-0.0330	0.030	-0.0330	0.50
1813:	65	37	29	1	65	64	37	28	1	64	218332.2479	-0.0330	0.030	-0.0330	0.50
1814:	65	38	27	1	65	64	38	26	1	64	218410.0684	-0.0589	0.030	-0.0590	0.50
1815:	65	38	28	1	65	64	38	27	1	64	218410.0684	-0.0589	0.030	-0.0590	0.50
1816:	76	19	57	1	76	76	18	58	1	76	218414.8720	0.0354	0.030	0.0354	0.50
1817:	76	19	58	1	76	76	18	59	1	76	218414.8720	0.0353	0.030	0.0354	0.50
1818:	65	13	53	1	65	64	13	52	1	64	218464.1297	0.0445	0.030		
1819:	65	13	52	1	65	64	13	51	1	64	218464.7566	0.0305	0.030		
1820:	67	4	63	1	67	66	5	62	1	66	218468.5218	-0.0659	0.030		
1821:	67	5	63	1	67	66	5	62	1	66	218486.8806	0.0289	0.030		
1822:	65	39	26	1	65	64	39	25	1	64	218490.7688	-0.0067	0.030	-0.0067	0.50
1823:	65	39	27	1	65	64	39	26	1	64	218490.7688	-0.0067	0.030	-0.0067	0.50
1824:	67	4	63	1	67	66	4	62	1	66	218492.7747	-0.0867	0.030		
1825:	67	5	63	1	67	66	4	62	1	66	218511.1454	0.0199	0.030		
1826:	75	19	56	1	75	75	18	57	1	75	218514.5056	0.0059	0.030	0.0059	0.50
1827:	75	19	57	1	75	75	18	58	1	75	218514.5056	0.0058	0.030	0.0059	0.50
1828:	74	19	55	1	74	74	18	56	1	74	218608.8098	-0.0124	0.030	-0.0124	0.50
1829:	74	19	56	1	74	74	18	57	1	74	218608.8098	-0.0124	0.030	-0.0124	0.50
1830:	65	41	24	1	65	64	41	23	1	64	218660.0335	0.0009	0.030	0.0009	0.50
1831:	65	41	25	1	65	64	41	24	1	64	218660.0335	0.0009	0.030	0.0009	0.50
1832:	64	9	55	1	64	63	9	54	1	63	218660.9744	-0.0341	0.030		
1833:	28	11	18	1	28	27	10	17	1	27	218686.3251	-0.0039	0.030	-0.0040	0.50
1834:	28	11	17	1	28	27	10	18	1	27	218686.3251	-0.0040	0.030	-0.0040	0.50
1835:	73	19	54	1	73	73	18	55	1	73	218697.9839	0.0069	0.030	0.0069	0.50
1836:	73	19	55	1	73	73	18	56	1	73	218697.9839	0.0069	0.030	0.0069	0.50
1837:	72	19	53	1	72	72	18	54	1	72	218782.1802	0.0475	0.030	0.0475	0.50
1838:	72	19	54	1	72	72	18	55	1	72	218782.1802	0.0475	0.030	0.0475	0.50
1839:	65	12	54	1	65	64	12	53	1	64	218786.0766	-0.0050	0.030		
1840:	65	12	53	1	65	64	12	52	1	64	218794.0624	0.0025	0.030		
1841:	71	19	52	1	71	71	18	53	1	71	218861.4341	-0.0203	0.030	-0.0203	0.50
1842:	71	19	53	1	71	71	18	54	1	71	218861.4341	-0.0203	0.030	-0.0203	0.50
1843:	70	19	51	1	70	70	18	52	1	70	218936.1162	0.0128	0.030	0.0128	0.50
1844:	70	19	52	1	70	70	18	53	1	70	218936.1162	0.0128	0.030	0.0128	0.50
1845:	69	19	50	1	69	69	18	51	1	69	219006.2124	-0.0248	0.030	-0.0249	0.50
1846:	69	19	51	1	69	69	18	52	1	69	219006.2124	-0.0248	0.030	-0.0249	0.50
1847:	64	7	57	1	64	63	7	56	1	63	219033.0798	-0.0102	0.030		
1848:	21	13	8	1	21	20	12	9	1	20	219051.5161	-0.0282	0.030	-0.0283	0.50
1849:	21	13	9	1	21	20	12	8	1	20	219051.5161	-0.0282	0.030	-0.0283	0.50
1850:	68	19	49	1	68	68	18	50	1	68	219072.0013	-0.0091	0.030	-0.0091	0.50
1851:	68	19	50	1	68	68	18	51	1	68	219072.0013	-0.0091	0.030	-0.0091	0.50
1852:	65	8	58	1	65	64	8	57	1	64	219096.0271	-0.0014	0.030		
1853:	67	19	48	1	67	67	18	49	1	67	219133.5696	-0.0042	0.030	-0.0042	0.50
1854:	67	19	49	1	67	67	18	50	1	67	219133.5696	-0.0042	0.030	-0.0042	0.50
1855:	68	3	65	1	68	67	4	64	1	67	219140.7963	-0.0246	0.030		
1856:	68	4	65	1	68	67	4	64	1	67	219141.6801	0.1442	0.030	0.0155	0.50
1857:	68	3	65	1	68	67	3	64	1	67	219141.6801	-0.1132	0.030	0.0155	0.50
1858:	68	4	65	1	68	67	3	64	1	67	219142.5528	0.0444	0.030		
1859:	66	19	47	1	66	66	18	48	1	66	219191.0495	-0.0256	0.030	-0.0257	0.50
1860:	66	19	48	1	66	66	18	49	1	66	219191.0495	-0.0256	0.030	-0.0257	0.50
1861:	65	11	55	1	65	64	11	54	1	64	219218.5242	-0.0357	0.030		
1862:	65	19	46	1	65	65	18	47	1	65	219244.6395	-0.0197	0.030	-0.0198	0.50
1863:	65	19	47	1	65	65	18	48	1	65	219244.6395	-0.0197	0.030	-0.0198	0.50
1864:	64	19	45	1	64	64	18	46	1	64	219294.4627	-0.0051	0.030	-0.0051	0.50
1865:	64	19	46	1	64	64	18	47	1	64	219294.4627	-0.0051	0.030	-0.0051	0.50
1866:	65	11	54	1	65	64	11	53	1	64	219297.1478	0.0235	0.030		
1867:	63	19	44	1	63	63	18	45	1	63	219340.6370	-0.0026	0.030	-0.0027	0.50
1868:	63	19	45	1	63	63	18	46	1	63	219340.6370	-0.0026	0.030	-0.0027	0.50
1869:	62	19	44	1	62	62	18	45	1	62	219383.3069	-0.0039	0.030	-0.0039	0.50
1870:	62	19	43	1	62	62	18	44	1	62	219383.3069	-0.0039	0.030	-0.0039	0.50
1871:	61	19	42	1	61	61	18	43	1	61	219422.6018	-0.0127	0.030	-0.0128	0.50
1872:	61	19	43	1	61	61	18	44	1	61	219422.6018	-0.0127	0.030	-0.0128	0.50
1873:	64	7	58	1	64	63	6	57	1	63	219445.5458	-0.0043	0.030		
1874:	60	19	41	1	60	60	18	42	1	60	219458.6743	-0.0073	0.030	-0.0073	0.50
1875:	60	19	42	1	60	60	18	43	1	60	219458.6743	-0.0073	0.030	-0.0073	0.50
1876:	20	19	1	1	20	20	18	2	1	20	219467.9708	0.0317	0.030	0.0318	0.50
1877:	20	19	2	1	20	20	18	3	1	20	219467.9708	0.0317	0.030	0.0318	0.50
1878:	21	19	2	1	21	21	18	3	1	21	219479.7039	0.0397	0.030	0.0398	0.50
1879:	21	19	3	1	21	21	18	4	1	21	219479.7039	0.0397	0.030	0.0398	0.50
1880:	59	19	40	1	59	59	18	41	1	59	219491.6221	-0.0178	0.030	-0.0179	0.50
1881:	59	19	41	1	59	59	18	42	1	59	219491.6221	-0.0178	0.030	-0.0179	0.50
1882:	23	19	4	1	23	23	18	5	1	23	219503.7183	-0.0030	0.030	-0.0030	0.50
1883:	23	19	5	1	23	23	18	6	1	23	219503.7183	-0.0030	0.030	-0.0030	0.50
1884:	58	19	39	1	58	58	18	40	1	58	219521.6616	0.0463	0.030	0.0463	0.50
1885:	58	19	40	1	58	58	18	41	1	58	219521.6616	0.0463	0.030	0.0463	0.50
1886:	24	19	5	1	24	24	18	6	1	24	219516.0286	0.0714	0.030	0.0714	0.50
1887:	24	19	6	1	24	24	18	7	1	24	219516.0286	0.0714	0.030	0.0714	0.50
1888:	25	19	6	1	25	25	18	7	1	25	219528.2412	-0.0225	0.030	-0.0225	0.50
1889:	25	19	7	1	25	25	18	8	1	25	219528.2412	-0.0225	0.030	-0.0225	0.50
1890:	26	19	7	1	26	26	18	8	1	26	219540.5865	-0.0011	0.030	-0.0012	0.50
1891:	26	19	8	1	26	26	18	9	1	26	219540.5865	-0.0011	0.030	-0.0012	0.50

```
1892: 57 19 38  1 57   57 18 39  1 57       219548.7259  -0.0046  0.030  -0.0046 0.50
1893: 57 19 39  1 57   57 18 40  1 57       219548.7259  -0.0046  0.030  -0.0046 0.50
1894: 27 19  8  1 27   27 18  9  1 27       219552.8863   0.0124  0.030   0.0124 0.50
1895: 27 19  9  1 27   27 18 10  1 27       219552.8863   0.0124  0.030   0.0124 0.50
1896: 28 19  9  1 28   28 18 10  1 28       219565.0490  -0.0161  0.030  -0.0161 0.50
1897: 28 19 10  1 28   28 18 11  1 28       219565.0490  -0.0161  0.030  -0.0161 0.50
1898: 56 19 37  1 56   56 18 38  1 56       219573.1306   0.0240  0.030   0.0241 0.50
1899: 56 19 38  1 56   56 18 39  1 56       219573.1306   0.0240  0.030   0.0241 0.50
1900: 29 19 10  1 29   29 18 11  1 29       219577.1069   0.0048  0.030   0.0048 0.50
1901: 29 19 11  1 29   29 18 12  1 29       219577.1069   0.0048  0.030   0.0048 0.50
1902: 30 19 11  1 30   30 18 12  1 30       219588.9202  -0.0034  0.030  -0.0034 0.50
1903: 30 19 12  1 30   30 18 13  1 30       219588.9202  -0.0034  0.030  -0.0034 0.50
1904: 55 19 36  1 55   55 18 37  1 55       219594.8600  -0.0015  0.030  -0.0016 0.50
1905: 55 19 37  1 55   55 18 38  1 55       219594.8600  -0.0015  0.030  -0.0016 0.50
1906: 31 19 12  1 31   31 18 13  1 31       219600.4145  -0.0519  0.030  -0.0519 0.50
1907: 31 19 13  1 31   31 18 14  1 31       219600.4145  -0.0519  0.030  -0.0519 0.50
1908: 32 19 13  1 32   32 18 14  1 32       219611.6710   0.0056  0.030   0.0057 0.50
1909: 32 19 14  1 32   32 18 15  1 32       219611.6710   0.0056  0.030   0.0057 0.50
1910: 54 19 36  1 54   54 18 37  1 54       219614.1155   0.0037  0.030   0.0038 0.50
1911: 54 19 35  1 54   54 18 36  1 54       219614.1155   0.0037  0.030   0.0038 0.50
1912: 33 19 14  1 33   33 18 15  1 33       219622.4613   0.0082  0.030   0.0082 0.50
1913: 33 19 15  1 33   33 18 16  1 33       219622.4613   0.0082  0.030   0.0082 0.50
1914: 53 19 34  1 53   53 18 35  1 53       219630.9604  -0.0103  0.030  -0.0103 0.50
1915: 53 19 35  1 53   53 18 36  1 53       219630.9604  -0.0103  0.030  -0.0103 0.50
1916: 34 19 15  1 34   34 18 16  1 34       219632.7599  -0.0007  0.030  -0.0007 0.50
1917: 34 19 16  1 34   34 18 17  1 34       219632.7599  -0.0007  0.030  -0.0007 0.50
1918: 35 19 16  1 35   35 18 17  1 35       219642.5242   0.0075  0.030   0.0075 0.50
1919: 35 19 17  1 35   35 18 18  1 35       219642.5242   0.0075  0.030   0.0075 0.50
1920: 52 19 33  1 52   52 18 34  1 52       219645.5302  -0.0199  0.030  -0.0199 0.50
1921: 52 19 34  1 52   52 18 35  1 52       219645.5302  -0.0199  0.030  -0.0199 0.50
1922: 36 19 17  1 36   36 18 18  1 36       219651.6057  -0.0425  0.030  -0.0426 0.50
1923: 36 19 18  1 36   36 18 19  1 36       219651.6057  -0.0425  0.030  -0.0426 0.50
1924: 51 19 32  1 51   51 18 33  1 51       219657.9470  -0.0123  0.030  -0.0123 0.50
1925: 51 19 33  1 51   51 18 34  1 51       219657.9470  -0.0123  0.030  -0.0123 0.50
1926: 37 19 18  1 37   37 18 19  1 37       219660.0785  -0.0017  0.030  -0.0018 0.50
1927: 37 19 19  1 37   37 18 20  1 37       219660.0785  -0.0017  0.030  -0.0018 0.50
1928: 38 19 20  1 38   38 18 21  1 38       219667.7319  -0.0037  0.030  -0.0037 0.50
1929: 38 19 19  1 38   38 18 20  1 38       219667.7319  -0.0037  0.030  -0.0037 0.50
1930: 50 19 31  1 50   50 18 32  1 50       219668.3326   0.0270  0.030   0.0271 0.50
1931: 50 19 32  1 50   50 18 33  1 50       219668.3326   0.0270  0.030   0.0271 0.50
1932: 39 19 20  1 39   39 18 21  1 39       219674.5548   0.0193  0.030   0.0194 0.50
1933: 39 19 21  1 39   39 18 22  1 39       219674.5548   0.0193  0.030   0.0194 0.50
1934: 49 19 31  1 49   49 18 32  1 49       219676.6896  -0.0042  0.030  -0.0043 0.50
1935: 49 19 30  1 49   49 18 31  1 49       219676.6896  -0.0042  0.030  -0.0043 0.50
1936: 40 19 21  1 40   40 18 22  1 40       219680.4517   0.0529  0.030   0.0530 0.50
1937: 40 19 22  1 40   40 18 23  1 40       219680.4517   0.0529  0.030   0.0530 0.50
1938: 48 19 29  1 48   48 18 30  1 48       219683.2372   0.0099  0.030   0.0100 0.50
1939: 48 19 30  1 48   48 18 31  1 48       219683.2372   0.0099  0.030   0.0100 0.50
1940: 41 19 23  1 41   41 18 24  1 41       219685.2248  -0.0177  0.030  -0.0178 0.50
1941: 41 19 22  1 41   41 18 23  1 41       219685.2248  -0.0177  0.030  -0.0178 0.50
1942: 47 19 29  1 47   47 18 30  1 47       219687.9348  -0.0717  0.030  -0.0718 0.50
1943: 47 19 28  1 47   47 18 29  1 47       219687.9348  -0.0717  0.030  -0.0718 0.50
1944: 42 19 24  1 42   42 18 25  1 42       219688.9614  -0.0206  0.030  -0.0207 0.50
1945: 42 19 23  1 42   42 18 24  1 42       219688.9614  -0.0206  0.030  -0.0207 0.50
1946: 46 19 27  1 46   46 18 28  1 46       219691.1004  -0.0303  0.030  -0.0304 0.50
1947: 46 19 28  1 46   46 18 29  1 46       219691.1004  -0.0303  0.030  -0.0304 0.50
1948: 43 19 25  1 43   43 18 26  1 43       219691.5631   0.0326  0.030   0.0326 0.50
1949: 43 19 24  1 43   43 18 25  1 43       219691.5631   0.0326  0.030   0.0326 0.50
1950: 45 19 26  1 45   45 18 27  1 45       219692.7412   0.0445  0.030   0.0037 0.30
1951: 45 19 27  1 45   45 18 28  1 45       219692.7412   0.0445  0.030   0.0037 0.30
1952: 44 19 25  1 44   44 18 26  1 44       219692.7412  -0.0577  0.030   0.0037 0.20
1953: 44 19 26  1 44   44 18 27  1 44       219692.7412  -0.0577  0.030   0.0037 0.20
1954: 65 10 56  1 65   64 10 55  1 64       219702.6828   0.0021  0.030
1955: 65  9 57  1 65   64  9 56  1 64       219870.4003   0.0048  0.030
1956: 32 10 23  1 32   31  9 22  1 31       219964.9505   0.0399  0.030   0.0065 0.50
1957: 32 10 22  1 32   31  9 23  1 31       219964.9505  -0.0268  0.030   0.0065 0.50
1958: 40  8 33  1 40   39  7 32  1 39       220193.5129  -0.0231  0.030
1959: 65 10 55  1 65   64 10 54  1 64       220273.1131   0.0234  0.030
1960: 66  7 60  1 66   65  7 59  1 65       220338.6256   0.0131  0.030
1961: 64  8 56  1 64   63  8 55  1 63       220554.7011   0.0061  0.030
1962: 70  1 69  1 70   69  2 68  1 69       220559.9696   0.0008  0.030   0.0005 0.30
1963: 70  2 69  1 70   69  2 68  1 69       220559.9696   0.0005  0.030   0.0005 0.20
1964: 70  1 69  1 70   69  1 68  1 69       220559.9696   0.0004  0.030   0.0005 0.20
1965: 70  2 69  1 70   69  1 68  1 69       220559.9696   0.0001  0.030   0.0005 0.30
1966: 25 12 13  1 25   24 11 14  1 24       220578.4104   0.0027  0.030   0.0028 0.50
1967: 25 12 14  1 25   24 11 13  1 24       220578.4104   0.0027  0.030   0.0028 0.50
1968: 67  5 62  1 67   66  6 61  1 66       220727.4626   0.0685  0.030
1969: 18 14  4  1 18   17 13  5  1 17       220831.9877   0.0244  0.030   0.0244 0.50
1970: 18 14  5  1 18   17 13  4  1 17       220831.9877   0.0244  0.030   0.0244 0.50
1971: 36  9 28  1 36   35  8 27  1 35       220832.5191  -0.0180  0.030
1972: 67  6 62  1 67   66  6 61  1 66       220969.5415   0.0075  0.030
1973: 66 22 44  1 66   65 22 43  1 65       220984.2616   0.0365  0.030   0.0365 0.50
1974: 66 22 45  1 66   65 22 44  1 65       220984.2616   0.0365  0.030   0.0365 0.50
```

1975:	66	23	43	1	66	65	23	42	1	65	220986.4300	-0.0101	0.030	-0.0102	0.50
1976:	66	23	44	1	66	65	23	43	1	65	220986.4300	-0.0101	0.030	-0.0102	0.50
1977:	66	21	45	1	66	65	21	44	1	65	220992.6764	-0.0213	0.030	-0.0214	0.50
1978:	66	21	46	1	66	65	21	45	1	65	220992.6764	-0.0213	0.030	-0.0214	0.50
1979:	66	24	42	1	66	65	24	41	1	65	220997.9283	0.0020	0.030	0.0020	0.50
1980:	66	24	43	1	66	65	24	42	1	65	220997.9283	0.0020	0.030	0.0020	0.50
1981:	66	20	46	1	66	65	20	45	1	65	221013.6665	0.0116	0.030	0.0117	0.50
1982:	66	20	47	1	66	65	20	46	1	65	221013.6665	0.0116	0.030	0.0117	0.50
1983:	66	25	41	1	66	65	25	40	1	65	221017.5571	0.0062	0.030	0.0062	0.50
1984:	66	25	42	1	66	65	25	41	1	65	221017.5571	0.0062	0.030	0.0062	0.50
1985:	66	6	60	1	66	65	6	59	1	65	221023.2534	0.0009	0.030		
1986:	67	5	62	1	67	66	5	61	1	66	221040.7724	-0.0113	0.030		
1987:	66	26	41	1	66	65	26	40	1	65	221044.3779	-0.0196	0.030	-0.0196	0.50
1988:	66	26	40	1	66	65	26	39	1	65	221044.3779	-0.0196	0.030	-0.0196	0.50
1989:	66	19	47	1	66	65	19	46	1	65	221049.4079	-0.0017	0.030	-0.0018	0.50
1990:	66	19	48	1	66	65	19	47	1	65	221049.4079	-0.0017	0.030	-0.0018	0.50
1991:	40	8	32	1	40	39	7	33	1	39	221052.9565	-0.0339	0.030		
1992:	66	27	39	1	66	65	27	38	1	65	221077.7165	0.0000	0.030	0.0001	0.50
1993:	66	27	40	1	66	65	27	39	1	65	221077.7165	0.0000	0.030	0.0001	0.50
1994:	66	18	48	1	66	65	18	47	1	65	221102.9963	0.0025	0.030	0.0025	0.50
1995:	66	18	49	1	66	65	18	48	1	65	221102.9963	0.0025	0.030	0.0025	0.50
1996:	66	28	38	1	66	65	28	37	1	65	221116.9126	0.0248	0.030	0.0249	0.50
1997:	66	28	39	1	66	65	28	38	1	65	221116.9126	0.0248	0.030	0.0249	0.50
1998:	66	29	37	1	66	65	29	36	1	65	221161.3699	-0.0241	0.030	-0.0242	0.50
1999:	66	29	38	1	66	65	29	37	1	65	221161.3699	-0.0241	0.030	-0.0242	0.50
2000:	66	17	49	1	66	65	17	48	1	65	221178.4447	-0.0178	0.030	-0.0179	0.50
2001:	66	17	50	1	66	65	17	49	1	65	221178.4447	-0.0178	0.030	-0.0179	0.50
2002:	67	6	61	1	67	66	7	60	1	66	221179.8204	-0.0237	0.030		
2003:	66	30	36	1	66	65	30	35	1	65	221210.8130	0.0132	0.030	0.0133	0.50
2004:	66	30	37	1	66	65	30	36	1	65	221210.8130	0.0132	0.030	0.0133	0.50
2005:	66	31	35	1	66	65	31	34	1	65	221264.7961	0.0615	0.030	0.0615	0.50
2006:	66	31	36	1	66	65	31	35	1	65	221264.7961	0.0615	0.030	0.0615	0.50
2007:	66	16	51	1	66	65	16	50	1	65	221281.4054	0.0281	0.030	0.0281	0.50
2008:	66	16	50	1	66	65	16	49	1	65	221281.4054	0.0279	0.030	0.0281	0.50
2009:	67	6	62	1	67	66	5	61	1	66	221282.9050	-0.0186	0.030		
2010:	71	0	71	1	71	70	0	70	1	70	221287.9361	0.0000	0.030	-0.0001	0.20
2011:	71	0	71	1	71	70	1	70	1	70	221287.9361	0.0000	0.030	-0.0001	0.30
2012:	71	1	71	1	71	70	0	70	1	70	221287.9361	0.0000	0.030	-0.0001	0.30
2013:	71	1	71	1	71	70	1	70	1	70	221287.9361	0.0000	0.030	-0.0001	0.20
2014:	66	32	34	1	66	65	32	33	1	65	221322.9091	0.0273	0.030	0.0273	0.50
2015:	66	32	35	1	66	65	32	34	1	65	221322.9091	0.0273	0.030	0.0273	0.50
2016:	66	33	33	1	66	65	33	32	1	65	221384.9505	-0.0173	0.030	-0.0173	0.50
2017:	66	33	34	1	66	65	33	33	1	65	221384.9505	-0.0173	0.030	-0.0173	0.50
2018:	66	15	52	1	66	65	15	51	1	65	221419.6022	0.0010	0.030	-0.0007	0.50
2019:	66	15	51	1	66	65	15	50	1	65	221419.6022	-0.0025	0.030	-0.0007	0.50
2020:	66	34	32	1	66	65	34	31	1	65	221450.7928	0.0377	0.030	0.0378	0.50
2021:	66	34	33	1	66	65	34	32	1	65	221450.7928	0.0377	0.030	0.0378	0.50
2022:	66	35	31	1	66	65	35	30	1	65	221520.0188	-0.0164	0.030	-0.0164	0.50
2023:	66	35	32	1	66	65	35	31	1	65	221520.0188	-0.0164	0.030	-0.0164	0.50
2024:	68	4	64	1	68	67	5	63	1	67	221549.2617	-0.0120	0.030		
2025:	68	5	64	1	68	67	5	63	1	67	221563.0082	0.0127	0.030		
2026:	65	7	59	1	65	64	6	58	1	64	221564.2244	0.0158	0.030		
2027:	68	4	64	1	68	67	4	63	1	67	221567.5487	0.0110	0.030		
2028:	68	5	64	1	68	67	4	63	1	67	221581.2605	0.0011	0.030		
2029:	66	36	30	1	66	65	36	29	1	65	221592.6109	-0.0139	0.030	-0.0140	0.50
2030:	66	36	31	1	66	65	36	30	1	65	221592.6109	-0.0139	0.030	-0.0140	0.50
2031:	66	14	53	1	66	65	14	52	1	65	221604.7224	0.0400	0.030	0.0088	0.50
2032:	66	14	52	1	66	65	14	51	1	65	221604.7224	-0.0224	0.030	0.0088	0.50
2033:	66	37	29	1	66	65	37	28	1	65	221668.3498	-0.0113	0.030	-0.0114	0.50
2034:	66	37	30	1	66	65	37	29	1	65	221668.3498	-0.0113	0.030	-0.0114	0.50
2035:	66	38	28	1	66	65	38	27	1	65	221747.0422	-0.0565	0.030	-0.0565	0.50
2036:	66	38	29	1	66	65	38	28	1	65	221747.0422	-0.0565	0.030	-0.0565	0.50
2037:	66	39	27	1	66	65	39	26	1	65	221828.7035	-0.0033	0.030	-0.0033	0.50
2038:	66	39	28	1	66	65	39	27	1	65	221828.7035	-0.0033	0.030	-0.0033	0.50
2039:	66	13	54	1	66	65	13	53	1	65	221854.2609	0.0254	0.030		
2040:	66	13	53	1	66	65	13	52	1	65	221855.1082	-0.0451	0.030		
2041:	43	7	36	1	43	42	6	37	1	42	221869.2992	-0.0032	0.030		
2042:	66	40	26	1	66	65	40	25	1	65	221913.0413	-0.0261	0.030	-0.0262	0.50
2043:	66	40	27	1	66	65	40	26	1	65	221913.0413	-0.0261	0.030	-0.0262	0.50
2044:	29	11	19	1	29	28	10	18	1	28	222014.3558	-0.0237	0.030	-0.0239	0.50
2045:	29	11	18	1	29	28	10	19	1	28	222014.3558	-0.0240	0.030	-0.0239	0.50
2046:	65	7	58	1	65	64	7	57	1	64	222021.8898	-0.0257	0.030		
2047:	66	12	55	1	66	65	12	54	1	65	222194.3147	-0.0145	0.030		
2048:	66	12	54	1	66	65	12	53	1	65	222205.3411	-0.0001	0.030		
2049:	69	3	66	1	69	68	4	65	1	68	222220.7403	-0.0339	0.030		
2050:	69	4	66	1	69	68	4	65	1	68	222221.4014	0.1019	0.030	0.0071	0.50
2051:	69	3	66	1	69	68	3	65	1	68	222221.4014	-0.0878	0.030	0.0071	0.50
2052:	69	4	66	1	69	68	3	65	1	68	222222.0652	0.0507	0.030		
2053:	69	7	62	1	69	68	8	61	1	68	222291.9532	0.0854	0.030		
2054:	66	8	59	1	66	65	8	58	1	65	222303.5027	0.0289	0.030		
2055:	65	9	56	1	65	64	9	55	1	64	222374.5870	-0.0168	0.030		
2056:	30	26	4	1	30	30	25	5	1	30	301163.3899	-0.0108	0.050	-0.0109	0.50
2057:	30	26	5	1	30	30	25	6	1	30	301163.3899	-0.0108	0.050	-0.0109	0.50

```
2058: 97  0 97  1 97   96  0 96  1 96       301182.5508   0.1218  0.050   0.1219 0.20
2059: 97  0 97  1 97   96  1 96  1 96       301182.5508   0.1218  0.050   0.1219 0.30
2060: 97  1 97  1 97   96  0 96  1 96       301182.5508   0.1218  0.050   0.1219 0.30
2061: 97  1 97  1 97   96  1 96  1 96       301182.5508   0.1218  0.050   0.1219 0.20
2062: 32 26  6  1 32   32 25  7  1 32       301215.9746  -0.0549  0.100  -0.0549 0.50
2063: 32 26  7  1 32   32 25  8  1 32       301215.9746  -0.0549  0.100  -0.0549 0.50
2064: 33 26  7  1 33   33 25  8  1 33       301243.2026   0.0263  0.050   0.0264 0.50
2065: 33 26  8  1 33   33 25  9  1 33       301243.2026   0.0263  0.050   0.0264 0.50
2066: 34 26  8  1 34   34 25  9  1 34       301270.8455   0.0018  0.050   0.0019 0.50
2067: 34 26  9  1 34   34 25 10  1 34       301270.8455   0.0018  0.050   0.0019 0.50
2068: 35 26  9  1 35   35 25 10  1 35       301299.0111   0.0056  0.050   0.0057 0.50
2069: 35 26 10  1 35   35 25 11  1 35       301299.0111   0.0056  0.050   0.0057 0.50
2070: 94  4 90  1 94   93  4 89  1 93       301318.3680   0.0790  0.050   0.0801 0.20
2071: 94  4 90  1 94   93  5 89  1 93       301318.3680   0.0870  0.050   0.0801 0.30
2072: 94  5 90  1 94   93  4 89  1 93       301318.3680   0.0731  0.050   0.0801 0.30
2073: 94  5 90  1 94   93  5 89  1 93       301318.3680   0.0811  0.050   0.0801 0.20
2074: 36 26 10  1 36   36 25 11  1 36       301327.6248  -0.0096  0.050  -0.0097 0.50
2075: 36 26 11  1 36   36 25 12  1 36       301327.6248  -0.0096  0.050  -0.0097 0.50
2076: 91  8 83  1 91   90  9 82  1 90       301340.1141  -0.0691  0.050
2077: 37 26 11  1 37   37 25 12  1 37       301356.7292   0.0263  0.050   0.0263 0.50
2078: 37 26 12  1 37   37 25 13  1 37       301356.7292   0.0263  0.050   0.0263 0.50
2079:119 26 94  1119  119 25 95  1119       301380.3412  -0.0985  0.050  -0.0985 0.50
2080:119 26 93  1119  119 25 94  1119       301380.3412  -0.0985  0.050  -0.0985 0.50
2081: 38 26 12  1 38   38 25 13  1 38       301386.2341   0.0519  0.050   0.0519 0.50
2082: 38 26 13  1 38   38 25 14  1 38       301386.2341   0.0519  0.050   0.0519 0.50
2083: 90 18 73  1 90   89 18 72  1 89       301414.9506  -0.0122  0.050  -0.0167 0.50
2084: 90 18 72  1 90   89 18 71  1 89       301414.9506  -0.0211  0.050  -0.0167 0.50
2085: 39 26 13  1 39   39 25 14  1 39       301416.0533   0.0100  0.050   0.0101 0.50
2086: 39 26 14  1 39   39 25 15  1 39       301416.0533   0.0100  0.050   0.0101 0.50
2087: 60  9 51  1 60   59  8 52  1 59       301428.3761   0.1823  0.100
2088: 40 26 14  1 40   40 25 15  1 40       301446.2569   0.0007  0.050   0.0007 0.50
2089: 40 26 15  1 40   40 25 16  1 40       301446.2569   0.0007  0.050   0.0007 0.50
2090:118 26 92  1118  118 25 93  1118       301465.6340   0.0149  0.050   0.0149 0.50
2091:118 26 93  1118  118 25 94  1118       301465.6340   0.0149  0.050   0.0149 0.50
2092: 41 26 15  1 41   41 25 16  1 41       301476.7911   0.0006  0.050   0.0006 0.50
2093: 41 26 16  1 41   41 25 17  1 41       301476.7911   0.0006  0.050   0.0006 0.50
2094: 35 16 19  1 35   34 15 20  1 34       301489.8242   0.0472  0.050   0.0473 0.50
2095: 35 16 20  1 35   34 15 19  1 34       301489.8242   0.0472  0.050   0.0473 0.50
2096: 42 26 16  1 42   42 25 17  1 42       301507.6289   0.0138  0.050   0.0138 0.50
2097: 42 26 17  1 42   42 25 18  1 42       301507.6289   0.0138  0.050   0.0138 0.50
2098: 43 26 17  1 43   43 25 18  1 43       301538.7309   0.0328  0.050   0.0328 0.50
2099: 43 26 18  1 43   43 25 19  1 43       301538.7309   0.0328  0.050   0.0328 0.50
2100:117 26 91  1117  117 25 92  1117       301547.0135   0.0181  0.050   0.0181 0.50
2101:117 26 92  1117  117 25 93  1117       301547.0135   0.0181  0.050   0.0181 0.50
2102: 21 20  1  1 21   20 19  2  1 20       301566.3373   0.0303  0.050   0.0304 0.50
2103: 21 20  2  1 21   20 19  1  1 20       301566.3373   0.0303  0.050   0.0304 0.50
2104: 44 26 19  1 44   44 25 20  1 44       301570.0263   0.0193  0.050   0.0193 0.50
2105: 44 26 18  1 44   44 25 19  1 44       301570.0263   0.0193  0.050   0.0193 0.50
2106:102 46 56  1102  101 46 55  1101       342029.9368  -0.2023  0.100  -0.2024 0.50
2107:102 46 57  1102  101 46 56  1101       342029.9368  -0.2023  0.100  -0.2024 0.50
2108:101 11 91  1101  100 10 90  1100       342118.1290   0.0605  0.100
2109: 51  6 46  1 51   50  3 47  1 50       342174.5106  -0.0132  0.050
2110:102 47 55  1102  101 47 54  1101       342177.0185   0.0560  0.050   0.0560 0.50
2111:102 47 56  1102  101 47 55  1101       342177.0185   0.0560  0.050   0.0560 0.50
2112: 67 11 56  1 67   66 10 57  1 66       342392.3488  -0.0441  0.050
2113:109  2107  1109  108  2106  1108       342456.0120  -0.0061  0.050  -0.0062 0.20
2114:109  2107  1109  108  3106  1108       342456.0120  -0.0061  0.050  -0.0062 0.30
2115:109  3107  1109  108  2106  1108       342456.0120  -0.0061  0.050  -0.0062 0.30
2116:109  3107  1109  108  3106  1108       342456.0120  -0.0061  0.050  -0.0062 0.20
2117:102 16 87  1102  101 16 86  1101       342500.7695  -0.0998  0.050
2118:102 16 86  1102  101 16 85  1101       342543.6075  -0.0327  0.050
2119:102 12 91  1102  101 12 90  1101       342608.8045   0.0633  0.100
2120:106  6100  1106  105  6 99  1105       342610.1303  -0.0298  0.050  -0.0171 0.20
2121:106  6100  1106  105  7 99  1105       342610.1303   0.0734  0.050  -0.0171 0.30
2122:106  7100  1106  105  6 99  1105       342610.1303  -0.1077  0.050  -0.0171 0.30
2123:106  7100  1106  105  7 99  1105       342610.1303  -0.0045  0.050  -0.0171 0.20
2124: 69 11 59  1 69   68 10 58  1 68       342617.2631  -0.0553  0.050
2125:100 12 88  1100   99 12 87  1 99       342629.3445   0.0721  0.050
2126: 51 15 37  1 51   50 14 36  1 50       342773.6518  -0.0271  0.050  -0.0272 0.50
2127: 51 15 36  1 51   50 14 37  1 50       342773.6518  -0.0272  0.050  -0.0272 0.50
2128:110  1109  1110  109  1108  1109       343201.1937  -0.0299  0.050  -0.0299 0.20
2129:110  1109  1110  109  2108  1109       343201.1937  -0.0299  0.050  -0.0299 0.30
2130:110  2109  1110  109  1108  1109       343201.1937  -0.0299  0.050  -0.0299 0.30
2131:110  2109  1110  109  2108  1109       343201.1937  -0.0299  0.050  -0.0299 0.20
2132:107  5102  1107  106  5101  1106       343304.1205  -0.0081  0.050  -0.0076 0.20
2133:107  5102  1107  106  6101  1106       343304.1205  -0.0042  0.050  -0.0076 0.30
2134:107  6102  1107  106  5101  1106       343304.1205  -0.0109  0.050  -0.0076 0.30
2135:107  6102  1107  106  6101  1106       343304.1205  -0.0071  0.050  -0.0076 0.20
2136: 23 23  0  1 23   22 22  1  1 22       343348.5883  -0.0053  0.050  -0.0054 0.50
2137: 23 23  1  1 23   22 22  0  1 22       343348.5883  -0.0053  0.050  -0.0054 0.50
2138: 44 17 27  1 44   43 16 28  1 43       343435.0261   0.0029  0.050   0.0029 0.50
2139: 44 17 28  1 44   43 16 27  1 43       343435.0261   0.0029  0.050   0.0029 0.50
2140: 63 12 52  1 63   62 11 51  1 62       343454.7197  -0.0166  0.050
```

2141:103 11 93	1103	102 11 92	1102	343466.3490	-0.0914	0.050		
2142:102 15 87	1102	101 15 86	1101	343476.2839	0.0382	0.050		
2143: 30 21 9	1 30	29 20 10	1 29	343569.5547	0.0006	0.050	0.0007	0.50
2144: 30 21 10	1 30	29 20 9	1 29	343569.5547	0.0006	0.050	0.0007	0.50
2145: 63 12 51	1 63	62 11 52	1 62	343583.7355	-0.0477	0.050		
2146: 37 19 18	1 37	36 18 19	1 36	343625.6667	0.0048	0.050	0.0048	0.50
2147: 37 19 19	1 37	36 18 18	1 36	343625.6667	0.0048	0.050	0.0048	0.50
2148:104 9 95	1104	103 10 94	1103	343647.0228	-0.0922	0.050		
2149:103 27 76	1103	102 27 75	1102	343654.7560	0.0162	0.050	0.0162	0.50
2150:103 27 77	1103	102 27 76	1102	343654.7560	0.0162	0.050	0.0162	0.50
2151:103 28 76	1103	102 28 75	1102	343657.6840	0.0222	0.050	0.0223	0.50
2152:103 28 75	1103	102 28 74	1102	343657.6840	0.0222	0.050	0.0223	0.50
2153:103 26 77	1103	102 26 76	1102	343668.6192	-0.0121	0.050	-0.0122	0.50
2154:103 26 78	1103	102 26 77	1102	343668.6192	-0.0121	0.050	-0.0122	0.50
2155:103 29 74	1103	102 29 73	1102	343675.4461	0.0134	0.050	0.0134	0.50
2156:103 29 75	1103	102 29 74	1102	343675.4461	0.0134	0.050	0.0134	0.50
2157:103 25 78	1103	102 25 77	1102	343701.6883	-0.0630	0.050	-0.0630	0.50
2158:103 25 79	1103	102 25 78	1102	343701.6883	-0.0630	0.050	-0.0630	0.50
2159:103 30 74	1103	102 30 73	1102	343706.7129	0.2774	0.150	0.2774	0.50
2160:103 30 73	1103	102 30 72	1102	343706.7129	0.2774	0.150	0.2774	0.50
2161: 55 14 42	1 55	54 13 41	1 54	343715.3114	0.0055	0.050	-0.0049	0.50
2162: 55 14 41	1 55	54 13 42	1 54	343715.3114	-0.0152	0.050	-0.0049	0.50
2163:103 31 72	1103	102 31 71	1102	343749.3661	0.0414	0.050	0.0415	0.50
2164:103 31 73	1103	102 31 72	1102	343749.3661	0.0414	0.050	0.0415	0.50
2165:103 24 79	1103	102 24 78	1102	343757.1097	0.0012	0.050	0.0012	0.50
2166:103 24 80	1103	102 24 79	1102	343757.1097	0.0012	0.050	0.0012	0.50
2167:102 13 90	1102	101 13 89	1101	343779.7375	0.0252	0.100		
2168:102 14 89	1102	101 14 88	1101	343786.8274	-0.0284	0.100		
2169:103 32 71	1103	102 32 70	1102	343803.0348	0.0647	0.050	0.0647	0.50
2170:103 32 72	1103	102 32 71	1102	343803.0348	0.0647	0.050	0.0647	0.50
2171:103 23 80	1103	102 23 79	1102	343838.4997	-0.0110	0.050	-0.0110	0.50
2172:103 23 81	1103	102 23 80	1102	343838.4997	-0.0110	0.050	-0.0110	0.50
2173:103 34 69	1103	102 34 68	1102	343938.7792	-0.0598	0.050	-0.0599	0.50
2174:103 34 70	1103	102 34 69	1102	343938.7792	-0.0598	0.050	-0.0599	0.50
2175:111 0111	1111	110 0110	1110	343946.0247	0.0104	0.050	0.0104	0.20
2176:111 0111	1111	110 1110	1110	343946.0247	0.0104	0.050	0.0104	0.30
2177:111 1111	1111	110 0110	1110	343946.0247	0.0104	0.050	0.0104	0.30
2178:111 1111	1111	110 1110	1110	343946.0247	0.0104	0.050	0.0104	0.20
2179:103 22 81	1103	102 22 80	1102	343950.9322	0.0654	0.050	0.0655	0.50
2180:103 22 82	1103	102 22 81	1102	343950.9322	0.0655	0.050	0.0655	0.50
2181: 64 12 53	1 64	63 11 52	1 63	346422.5620	-0.0714	0.050		
2182:104 11 94	1104	103 11 93	1103	346494.5979	0.0720	0.050		
2183:103 15 89	1103	102 15 88	1102	346567.8257	-0.0377	0.050		
2184: 64 12 52	1 64	63 11 53	1 63	346601.2401	0.0002	0.050		
2185:103 11 93	1103	102 10 92	1102	346649.6617	0.2505	0.100		
2186: 24 23 1	1 24	23 22 2	1 23	346718.2084	0.0047	0.050	0.0048	0.50
2187: 24 23 2	1 24	23 22 1	1 23	346718.2084	0.0047	0.050	0.0048	0.50
2188:105 9 96	1105	104 10 95	1104	346721.3960	0.1414	0.100		
2189: 45 17 28	1 45	44 16 29	1 44	346772.1976	-0.0011	0.050	-0.0012	0.50
2190: 45 17 29	1 45	44 16 28	1 44	346772.1976	-0.0011	0.050	-0.0012	0.50
2191: 72 11 62	1 72	71 10 61	1 71	346881.7751	-0.0105	0.050		
2192:105 10 96	1105	104 10 95	1104	346934.2027	-0.0772	0.050		
2193: 31 21 10	1 31	30 20 11	1 30	346936.3207	0.0003	0.050	0.0004	0.50
2194: 31 21 11	1 31	30 20 10	1 30	346936.3207	0.0003	0.050	0.0004	0.50
2195:103 15 88	1103	102 15 87	1102	346942.3032	-0.0818	0.150		
2196:104 27 77	1104	103 27 76	1103	346943.8939	0.0439	0.050	0.0439	0.50
2197:104 27 78	1104	103 27 77	1103	346943.8939	0.0439	0.050	0.0439	0.50
2198:104 28 76	1104	103 28 75	1103	346944.9488	-0.0168	0.050	-0.0169	0.50
2199:104 28 77	1104	103 28 76	1103	346944.9488	-0.0168	0.050	-0.0169	0.50
2200: 56 14 43	1 56	55 13 42	1 55	346954.1826	0.0302	0.050	0.0137	0.50
2201: 56 14 42	1 56	55 13 43	1 55	346954.1826	-0.0029	0.050	0.0137	0.50
2202:104 26 79	1104	103 26 78	1103	346959.9199	-0.0517	0.050	-0.0517	0.50
2203:104 26 78	1104	103 26 77	1103	346959.9199	-0.0517	0.050	-0.0517	0.50
2204:104 29 75	1104	103 29 74	1103	346961.2716	-0.0205	0.050	-0.0206	0.50
2205:104 29 76	1104	103 29 75	1103	346961.2716	-0.0205	0.050	-0.0206	0.50
2206:104 10 94	1104	103 10 93	1103	346976.5549	0.0426	0.100		
2207: 38 19 20	1 38	37 18 19	1 37	346983.6916	0.0322	0.050	0.0322	0.50
2208: 38 19 19	1 38	37 18 20	1 37	346983.6916	0.0322	0.050	0.0322	0.50
2209:105 9 96	1105	104 9 95	1104	346989.7365	-0.0605	0.050		
2210:104 30 75	1104	103 30 74	1103	346991.1635	0.0006	0.050	0.0007	0.50
2211:104 30 74	1104	103 30 73	1103	346991.1635	0.0006	0.050	0.0007	0.50
2212:112 0112	1112	111 0111	1111	346992.6954	-0.0166	0.050	-0.0167	0.20
2213:112 0112	1112	111 1111	1111	346992.6954	-0.0166	0.050	-0.0167	0.30
2214:112 1112	1112	111 0111	1111	346992.6954	-0.0166	0.050	-0.0167	0.30
2215:112 1112	1112	111 1111	1111	346992.6954	-0.0166	0.050	-0.0167	0.20
2216:104 25 79	1104	103 25 78	1103	346995.8497	0.0255	0.050	0.0256	0.50
2217:104 25 80	1104	103 25 79	1103	346995.8497	0.0255	0.050	0.0256	0.50
2218:103 13 91	1103	102 13 90	1102	347027.2140	0.0270	0.050		
2219:104 31 73	1104	103 31 72	1103	347033.1864	-0.0056	0.050	-0.0056	0.50
2220:104 31 74	1104	103 31 73	1103	347033.1864	-0.0056	0.050	-0.0056	0.50
2221:104 24 80	1104	103 24 79	1103	347054.5031	-0.0152	0.050	-0.0152	0.50
2222:104 24 81	1104	103 24 80	1103	347054.5031	-0.0152	0.050	-0.0152	0.50
2223:109 4105	1109	108 4104	1108	347066.5601	-0.0371	0.050	-0.0372	0.20

```
2224:109  4105  1109  108  5104  1108       347066.5601  -0.0371  0.050  -0.0372 0.30
2225:109  5105  1109  108  4104  1108       347066.5601  -0.0372  0.050  -0.0372 0.30
2226:109  5105  1109  108  5104  1108       347066.5601  -0.0371  0.050  -0.0372 0.20
2227:104 32 72  1104  103 32 71  1103       347086.2613   0.0446  0.050   0.0446 0.50
2228:104 32 73  1104  103 32 72  1103       347086.2613   0.0446  0.050   0.0446 0.50
2229:104 23 82  1104  103 23 81  1103       347139.9714  -0.0258  0.050  -0.0259 0.50
2230:104 23 81  1104  103 23 80  1103       347139.9714  -0.0258  0.050  -0.0259 0.50
2231:103 14 90  1103  102 14 89  1102       347146.8069  -0.0502  0.050
2232:104 33 72  1104  103 33 71  1103       347149.2806   0.0287  0.050   0.0288 0.50
2233:104 33 71  1104  103 33 70  1103       347149.2806   0.0287  0.050   0.0288 0.50
2234: 31 30  1  1 31   31 29  2  1 31       347163.5360   0.1159  0.050   0.1160 0.50
2235: 31 30  2  1 31   31 29  3  1 31       347163.5360   0.1159  0.050   0.1160 0.50
2236: 32 30  2  1 32   32 29  3  1 32       347195.8794  -0.0961  0.050  -0.0961 0.50
2237: 32 30  3  1 32   32 29  4  1 32       347195.8794  -0.0961  0.050  -0.0961 0.50
2238:104 34 70  1104  103 34 69  1103       347221.2204  -0.2361  0.100  -0.2362 0.50
2239:104 34 71  1104  103 34 70  1103       347221.2204  -0.2361  0.100  -0.2362 0.50
2240: 33 30  3  1 33   33 29  4  1 33       347229.3398  -0.0275  0.050  -0.0275 0.50
2241: 33 30  4  1 33   33 29  5  1 33       347229.3398  -0.0275  0.050  -0.0275 0.50
2242:104 22 82  1104  103 22 81  1103       347257.3400  -0.0133  0.050  -0.0134 0.50
2243:104 22 83  1104  103 22 82  1103       347257.3400  -0.0133  0.050  -0.0134 0.50
2244: 34 30  5  1 34   34 29  6  1 34       347263.5361  -0.0429  0.050  -0.0430 0.50
2245: 34 30  4  1 34   34 29  5  1 34       347263.5361  -0.0429  0.050  -0.0430 0.50
2246: 60 13 48  1 60   59 12 47  1 59       347278.7631   0.0432  0.050
2247: 60 13 47  1 60   59 12 48  1 59       347282.0683   0.0055  0.050
2248: 35 30  5  1 35   35 29  6  1 35       347298.5352  -0.0587  0.050  -0.0587 0.50
2249: 35 30  6  1 35   35 29  7  1 35       347298.5352  -0.0587  0.050  -0.0587 0.50
2250:104 35 69  1104  103 35 68  1103       347302.0971  -0.0105  0.050  -0.0106 0.50
2251:104 35 70  1104  103 35 69  1103       347302.0971  -0.0105  0.050  -0.0106 0.50
2252: 36 30  6  1 36   36 29  7  1 36       347334.3662  -0.0285  0.050  -0.0285 0.50
2253: 36 30  7  1 36   36 29  8  1 36       347334.3662  -0.0285  0.050  -0.0285 0.50
2254: 37 30  7  1 37   37 29  8  1 37       347370.9363  -0.0274  0.050  -0.0274 0.50
2255: 37 30  8  1 37   37 29  9  1 37       347370.9363  -0.0274  0.050  -0.0274 0.50
2256:104 36 68  1104  103 36 67  1103       347390.5772  -0.0013  0.050  -0.0013 0.50
2257:104 36 69  1104  103 36 68  1103       347390.5772  -0.0013  0.050  -0.0013 0.50
2258: 38 30  8  1 38   38 29  9  1 38       347408.2723  -0.0106  0.050  -0.0106 0.50
2259: 38 30  9  1 38   38 29 10  1 38       347408.2723  -0.0106  0.050  -0.0106 0.50
2260:104 21 84  1104  103 21 83  1103       347413.3089  -0.0060  0.050  -0.0062 0.50
2261:104 21 83  1104  103 21 82  1103       347413.3089  -0.0064  0.050  -0.0062 0.50
2262:106  8 98  1106  105  9 97  1105       347416.0942   0.0651  0.050
2263:106  9 98  1106  105  9 97  1105       347432.2207  -0.0002  0.050
2264:106  8 98  1106  105  8 97  1105       347436.8699   0.0221  0.050
2265: 39 30  9  1 39   39 29 10  1 39       347446.3509   0.0170  0.050   0.0171 0.50
2266: 39 30 10  1 39   39 29 11  1 39       347446.3509   0.0170  0.050   0.0171 0.50
2267:106  9 98  1106  105  8 97  1105       347453.1030   0.0633  0.050
2268: 40 30 10  1 40   40 29 11  1 40       347485.0756  -0.0218  0.050  -0.0218 0.50
2269: 40 30 11  1 40   40 29 12  1 40       347485.0756  -0.0218  0.050  -0.0218 0.50
2270: 41 30 11  1 41   41 29 12  1 41       347524.5532  -0.0012  0.050  -0.0013 0.50
2271: 41 30 12  1 41   41 29 13  1 41       347524.5532  -0.0012  0.050  -0.0013 0.50
2272: 42 30 12  1 42   42 29 13  1 42       347564.6752  -0.0099  0.050  -0.0100 0.50
2273: 42 30 13  1 42   42 29 14  1 42       347564.6752  -0.0099  0.050  -0.0100 0.50
2274:104 38 66  1104  103 38 65  1103       347588.9216   0.0604  0.050   0.0604 0.50
2275:104 38 67  1104  103 38 66  1103       347588.9216   0.0604  0.050   0.0604 0.50
2276: 43 30 13  1 43   43 29 14  1 43       347605.5198   0.0504  0.050   0.0504 0.50
2277: 43 30 14  1 43   43 29 15  1 43       347605.5198   0.0504  0.050   0.0504 0.50
2278:104 20 85  1104  103 20 84  1103       347617.0547   0.0311  0.050   0.0283 0.50
2279:104 20 84  1104  103 20 83  1103       347617.0547   0.0254  0.050   0.0283 0.50
2280: 44 30 14  1 44   44 29 15  1 44       347646.8969   0.0103  0.050   0.0104 0.50
2281: 44 30 15  1 44   44 29 16  1 44       347646.8969   0.0103  0.050   0.0104 0.50
2282: 45 30 15  1 45   45 29 16  1 45       347688.9755   0.0597  0.050   0.0597 0.50
2283: 45 30 16  1 45   45 29 17  1 45       347688.9755   0.0597  0.050   0.0597 0.50
2284: 46 30 16  1 46   46 29 17  1 46       347731.5809   0.0452  0.050   0.0452 0.50
2285: 46 30 17  1 46   46 29 18  1 46       347731.5809   0.0452  0.050   0.0452 0.50
-------------------------------------------------------------------------------

PARAMETERS IN FIT (values truncated and Nlines statistics):

        10099            A  /MHz         7635.62481(22)                       1
        20099            B  /MHz         1788.636927(35)                      2
        30099            C  /MHz         1554.218399(39)                      3
          299           DJ  /kHz            0.878122(13)                      4
         1199          DJK  /kHz          -10.00175(14)                       5
         2099           DK  /kHz           52.4857(12)                        6
        40199       deltaJ  /kHz            0.2333427(69)                     7
        41099       deltaK  /kHz            1.72956(65)                       8
          399           HJ  /Hz             0.00058741(94)                    9
         1299          HJK  /Hz             0.01675(18)                      10
         2199          HKJ  /Hz            -0.36068(72)                      11
         3099           HK  /Hz             1.4592(26)                       12
        40299           hJ  /Hz             0.0002656(15)                    13
        41199          hJK  /Hz            -0.00469(22)                      14
        42099           hK  /Hz             0.1004(60)                       15
         1399         LJJK  /mHz           -0.0000199(19)                    16
         2299          LJK  /mHz           -0.001215(13)                     17
```

3199	LKKJ	/mHz	0.01862(31)	18
4099	LK	/mHz	-0.0646(15)	19
40399	lJ	/mHz	-0.000000629(78)	20
41299	lJK	/mHz	-0.000082(13)	21
4199	PKKJ	/mHz	-0.000000375(93)	22
110010000	X.aa*3/2	/MHz	-0.0621(32)	23
110040000	X.(b-c)/4	/MHz	-0.97787(67)	24

MICROWAVE AVG = 0.000391 MHz, IR AVG = 0.00000
MICROWAVE RMS = 0.030317 MHz, IR RMS = 0.00000
END OF ITERATION 1 OLD, NEW RMS ERROR= 0.93721 0.93721

distinct frequency lines in fit: 1464
distinct parameters of fit: 24

MICROWAVE	lines fitted total	dv=0	dv.ne.0	lines UNFITTD	lines e>900	RMS	RMS ERROR	J range	Ka range	freq. range
v"= 0	63	63	0	0	0	0.003060	1.01241	1 8	0 2	4381 19151
v"= 1	1401	1401	0	0	0	0.030969	0.93227	5 119	0 47	75021 347732
total:	1464	1464	0	0	0	0.030302	0.93586			

NOTE: the RMS values above are for Nlines statistics, but the 'total' values may differ slightly from those in the .FIT file since the o-c values for this evaluation are as rounded in the .FIT.

PARAMETERS IN FIT WITH STANDARD ERRORS ON THOSE THAT ARE FITTED:
(values rounded and degrees of freedom, Ndegf=Nlines-Nconst, statistics)

10099	A	/MHz	7635.62481(21)	1
20099	B	/MHz	1788.636927(33)	2
30099	C	/MHz	1554.218399(37)	3
299	DJ	/kHz	0.878123(12)	4
1199	DJK	/kHz	-10.00176(14)	5
2099	DK	/kHz	52.4857(12)	6
40199	deltaJ	/kHz	0.2333427(65)	7
41099	deltaK	/kHz	1.72956(61)	8
399	HJ	/Hz	0.00058741(89)	9
1299	HJK	/Hz	0.01675(17)	10
2199	HKJ	/Hz	-0.36068(68)	11
3099	HK	/Hz	1.4593(25)	12
40299	hJ	/Hz	0.0002657(14)	13
41199	hJK	/Hz	-0.00470(21)	14
42099	hK	/Hz	0.1004(57)	15
1399	LJJK	/mHz	-0.0000199(18)	16
2299	LJK	/mHz	-0.001216(12)	17
3199	LKKJ	/mHz	0.01863(30)	18
4099	LK	/mHz	-0.0647(14)	19
40399	lJ	/mHz	-0.000000629(74)	20
41299	lJK	/mHz	-0.000082(12)	21
4199	PKKJ	/mHz	-0.000000375(88)	22
110010000	X.aa*3/2	/MHz	-0.0621(30)	23
110040000	X.(b-c)/4	/MHz	-0.97787(63)	24

Worst fitted parameters, with greater than 20% uncertainty: %

4199	PKKJ	/mHz	-0.000000375(88)	22	23.4

CORRELATION COEFFICIENTS, C.ij:

	A	B	C	-DJ	-DJK	-DK	-deltaJ	-deltaK
A	1.0000							
B	0.1237	1.0000						
C	0.3364	0.6307	1.0000					
-DJ	-0.1908	-0.7116	-0.7940	1.0000				
-DJK	-0.5127	-0.2651	-0.3187	0.1572	1.0000			
-DK	-0.7302	0.0717	-0.1144	0.0621	0.0329	1.0000		
-deltaJ	0.2301	-0.2356	0.4561	-0.2420	0.0070	-0.1869	1.0000	
-deltaK	0.0920	0.3218	0.3370	-0.3262	-0.0310	-0.0962	-0.0759	1.0000
HJ	0.1816	0.5431	0.6622	-0.9032	-0.0853	-0.1004	0.3135	0.1654
HJK	0.0599	0.2548	0.2748	-0.4684	-0.0170	-0.0296	0.0455	0.0748
HKJ	0.1985	-0.1701	-0.1463	0.3631	-0.4131	-0.0561	-0.0253	-0.1171
HK	0.5489	-0.0061	0.1405	-0.1390	0.1080	-0.9290	0.1553	0.1263
hJ	-0.1716	0.2206	-0.3169	0.1146	-0.0253	0.1419	-0.9155	0.1521
hJK	-0.1438	-0.4551	-0.5235	0.6810	0.1136	0.0902	-0.0640	-0.8401
hK	0.0389	0.2022	0.2221	-0.4175	0.0336	-0.0377	0.0463	0.0270
LJJK	-0.1352	-0.4422	-0.4785	0.6778	0.1325	0.0383	-0.0914	-0.2587
LJK	-0.0579	0.0937	0.0972	-0.2160	0.2682	-0.0566	0.0578	0.0549

```
LKKJ       -0.4473  -0.0004  -0.0768  -0.0272   0.7116   0.2150  -0.0189   0.1532
LK         -0.4712   0.0305  -0.0969   0.0697  -0.1466   0.8852  -0.1340  -0.1209
lJ          0.1576  -0.1714   0.2926  -0.1386   0.0345  -0.1356   0.8503  -0.1889
lJK         0.1588   0.4711   0.5695  -0.7732  -0.1162  -0.0964   0.1449   0.7398
PKKJ        0.4171  -0.0157   0.0534   0.0589  -0.6684  -0.2192   0.0041  -0.1323
X.aa*3/2    0.0489   0.0121   0.0183  -0.0125  -0.0261  -0.0348   0.0079   0.0063
X.(b-c)/4  -0.0422  -0.0107  -0.0052   0.0047   0.0219   0.0308   0.0041  -0.0041

           HJ       HJK      HKJ      HK       hJ       hJK      hK       LJJK

HJ          1.0000
HJK         0.4787   1.0000
HKJ        -0.3851  -0.8607   1.0000
HK          0.1740   0.2055  -0.2094   1.0000
hJ         -0.2511   0.1197  -0.0981  -0.0932   1.0000
hJK        -0.4878  -0.3358   0.2642  -0.1375  -0.1096   1.0000
hK          0.4484   0.9929  -0.8531   0.2041   0.1118  -0.2855   1.0000
LJJK       -0.6809  -0.7665   0.7060  -0.2151  -0.0403   0.4836  -0.7061   1.0000
LJK         0.3391   0.1306  -0.2864   0.1451  -0.0750  -0.0643   0.1287  -0.5191
LKKJ        0.0233   0.0836  -0.5606   0.0246  -0.0027  -0.0396   0.0652  -0.1806
LK         -0.0957  -0.0529   0.1173  -0.9760   0.1003   0.0860  -0.0482   0.1083
lJ          0.3224  -0.0337   0.0192   0.1058  -0.9768   0.1458  -0.0216  -0.0365
lJK         0.6452   0.3987  -0.3061   0.1488   0.0221  -0.9719   0.3518  -0.5388
PKKJ       -0.0870  -0.0630   0.5207   0.0013   0.0308   0.0314  -0.0493   0.2358
X.aa*3/2    0.0111   0.0041   0.0088   0.0263  -0.0056  -0.0092   0.0028  -0.0087
X.(b-c)/4  -0.0039  -0.0014  -0.0093  -0.0229  -0.0035   0.0046  -0.0006   0.0041

           LJK      LKKJ     LK       lJ       lJK      PKKJ     X.aa*3/2 X.(b-c)/

LJK         1.0000
LKKJ        0.2594   1.0000
LK         -0.1228  -0.0998   1.0000
lJ          0.1438   0.0137  -0.0993   1.0000
lJK         0.0785   0.0208  -0.0859  -0.0410   1.0000
PKKJ       -0.4448  -0.9622   0.0671  -0.0559  -0.0186   1.0000
X.aa*3/2   -0.0024  -0.0216  -0.0225   0.0054   0.0099   0.0200   1.0000
X.(b-c)/4   0.0031   0.0191   0.0197   0.0027  -0.0044  -0.0179  -0.0976   1.0000

 Mean value of |C.ij|, i.ne.j =   0.2141
 Mean value of  C.ij,  i.ne.j =  -0.0257


 No correlations with absolute value greater than 0.9950


 Worst fitted lines (obs-calc/error):

   203:   -4.1         73:   -4.0        500:    3.7        171:   -3.6
   156:   -3.4       1088:    3.0        897:   -2.9        401:   -2.9
  1824:   -2.9        975:    2.9        707:   -2.9       1602:    2.9
  1453:   -2.9       2053:    2.8       1431:    2.8        918:    2.8
  1497:    2.7        501:    2.7        481:    2.7        328:   -2.7
   207:   -2.7       1124:    2.6        479:    2.6        475:   -2.6
  1530:   -2.6        181:    2.6         97:    2.6         96:    2.5
  1706:   -2.5       2185:    2.5        407:   -2.5       1601:   -2.5
   636:   -2.4       2058:    2.4        202:    2.4       1942:   -2.4
   556:    2.4       1886:    2.4       2238:   -2.4       1051:    2.4
  1491:    2.3       1774:    2.3        169:   -2.3       2234:    2.3
  1599:   -2.3          5:   -2.3        634:   -2.3       1668:   -2.3
  1968:    2.3        410:   -2.3

   203/ 24  7 17  1 24   23  7 16  1 23        80563.2752  -0.1014  0.025
    73/ 16  3 14  1 16   15  2 13  1 15        75715.7683  -0.1010  0.025
   500/ 30  9 21  1 30   30  8 22  1 30       100558.2053   0.0931  0.025
   171/ 18  3 16  1 18   17  2 15  1 17        79324.5139  -0.0907  0.025
   156/ 38  3 36  1 38   38  2 37  1 38        77499.0446  -0.0845  0.025
  1088: 61  5 57  1 61   60  4 56  1 60       200147.0388   0.0887  0.030
   897: 35  9 26  1 35   34  9 25  1 34       117562.1056  -0.0732  0.025
   401: 32  3 29  1 32   31  4 28  1 31        96661.2653  -0.0730  0.025
  1824: 67  4 63  1 67   66  4 62  1 66       218492.7747  -0.0867  0.030
   975: 50 11 39  1 50   50 10 40  1 50       122105.7600   0.0721  0.025
                                               _____________________________________
_________________________________________/ SPFIT output reformatted with PIFORM


//////////////////////////////////////////////////////////////////////////////////////////
//////////////////////////////////////////////////////////////////////////////////////////
//////////////////////////////////////////////////////////////////////////////////////////
```
